# Anatomy and systematics of the sauropodomorph *Sarahsaurus aurifontanalis* from the Early Jurassic Kayenta Formation

**DOI:** 10.1371/journal.pone.0204007

**Published:** 2018-10-10

**Authors:** Adam D. Marsh, Timothy B. Rowe

**Affiliations:** 1 The Jackson School of Geosciences, University of Texas at Austin, Austin, Texas, United States of America; 2 Division of Science and Resource Management, Petrified Forest National Park, Arizona, United States of America; Indiana University Bloomington, UNITED STATES

## Abstract

*Sarahsaurus aurifontanalis*, from the Kayenta Formation of Arizona, is one of only three sauropodomorph dinosaurs known from the Early Jurassic of North America. It joins *Anchisaurus polyzelus*, from the older Portland Formation of the Hartford Basin, and *Seitaad reussi*, from the younger Navajo Sandstone of Utah, in representing the oldest North American sauropodomorphs. If it is true that sauropodomorphs were absent from North America during the Late Triassic, the relationship among these three dinosaurs offers a test of the mechanisms that drove recovery in North American biodiversity following the end-Triassic extinction event. Here we provide the first thorough description of *Sarahsaurus aurifontanalis* based on completed preparation and computed tomographic imaging of the holotype and referred specimens. With new anatomical data, our phylogenetic analysis supports the conclusion that *Sarahsaurus aurifontanalis* is nested within the primarily Gondwanan clade Massospondylidae, while agreeing with previous analyses that the three North American sauropodomorphs do not themselves form an exclusive clade. A revised diagnosis and more thorough understanding of the anatomy of *Sarahsaurus aurifontanalis* support the view that independent dispersal events were at least partly responsible for the recovery in North American vertebrate diversity following a major extinction event.

## Introduction

In the late 19th Century O. C. Marsh of the Yale Peabody Museum described the first remains of North American early sauropodomorph dinosaurs, from the Early Jurassic Portland Formation of Massachusetts and Connecticut [1–6]. Several names were coined for these specimens, but most recent authors consider all this material to represent a single taxon, and most (but not all) recognize *Anchisaurus* (*Ammosaurus*) *polyzelus* as its valid name [7–10]. We follow this convention below.

Several decades would pass before additional early sauropodomorph specimens were found in North America, and nearly a century before informative new specimens were recovered. The next sauropodmorph to be described was a fragmentary postcranial skeleton collected by Lionel F. Brady of the Museum of Northern Arizona. It was found in northern Arizona in the uppermost formation of the Glen Canyon Group, the Early Jurassic Navajo Sandstone. Brady referred this material to *Anchisaurus* (*Ammosaurus*) [11, 12], believing this taxon to be a theropod dinosaur. Galton [7, 13] later correctly interpreted *Anchisaurus* (*Ammosaurus*) as an early sauropodomorph, to which he referred Brady’s material plus a second fragmentary specimen from the Navajo Sandstone of Arizona that was collected by Charles L. Camp for the University of California Museum of Paleontology. Yates [8] subsequently argued that the two Navajo Sandstone specimens represented a single taxon and that it was distinct from *Anchisaurus* (*Ammosaurus*). He thought it was possibly related to *Massospondylus carinatus* [14] from southern Africa, but he deemed the material too incomplete for a definitive referral.

Between 1976 and 1978, a joint expedition from the Museum of Northern Arizona and Harvard’s Museum of Comparative Zoology (MCZ) explored another part of the Glen Canyon Group in northern Arizona, the so-called ‘Silty Facies’ of the Early Jurassic Kayenta Formation, which underlies and interfingers with the Navajo Sandstone. They collected a single broken sauropodomorph skull and associated postcranial fragments from the base of a geographic feature known as ‘Rock Head.’ Owing to the questionable taxonomic identity (below), we informaly designate this as the ‘Rock Head specimen.’ The specimen was reposited in the MCZ (MCZ 8893).

In the initial description [15], the Rock Head specimen was referred to *Massospondylus* sp., a well-known taxon represented by numerous relatively complete skeletons from multiple localities in southern Africa [14]. Its identification as *Massospondylus* sp. was taken as evidence further supporting the concept that a cosmopolitan dinosaur fauna of low taxonomic diversity occupied Pangaea in the Late Triassic and Early Jurassic [9, 10, 16–18]. Subsequent workers expressed doubts about its referral to *Massospondylus* sp., and it became known as ‘the unnamed Kayenta prosauropod’ (e.g., [19] p. 27, [20]). Uncertainty about its identity also raised some of the first doubts to be cast over the prevailing view of cosmopolitan dinosaur faunas of low taxonomic diversity across Pangaea in the Late Triassic and Early Jurassic.

In 2006, the sauropodomorph “*Fendusaurus eldoni*” from the Early Jurassic McCoy Brook Formation of Nova Scotia was named on a very poorly preserved specimen and given preliminary description in an unpublished dissertation [21]. A formal description and diagnosis has yet to be published.

In 2010, preliminary descriptions of two new early sauropodomorphs from the Glen Canyon Group of the Colorado Plateau were published. The first was *Seitaad ruessi*, described by Sertich and Loewen [22] based on a single, partial skeleton lacking the skull. It was the third sauropodomorph specimen reported from the Navajo Sandstone; it was collected in southern Utah by the Utah Museum of Natural History [22, 23]. *Seitaad ruessi* was postulated to have affinities either to plateosaurid or to massospondylid sauropodomorphs, but its incompleteness left a measure of phylogenetic uncertainty that was compounded by more general uncertainty and poor phylogenetic resolution for this early history of the sauropodomorph lineage [24, 25]. The two specimens previously reported from the Navajo Sandstone were found to compare favorably to *Seitaad ruessi*, but they were considered too fragmentary for a confident referral [22].

Next to be described was *Sarahsaurus aurifontanalis*, based on two partial skeletons [25]. Both individuals were collected from a single quarry in the Silty Facies of the Kayenta Formation during a collaborative survey by The University of Texas Vertebrate Paleontology Laboratory and the Navajo Nation EcoScouts that took place between 1997–2000. In the initial diagnosis and description of *Sarahsaurus aurifontanalis*, the holotype was designated as the more mature and complete of the two skeletons and the only one that included parts of a disarticuated skull; the less-mature and less-complete skeleton was designated the paratype [25]. In this initial description, the Rock Head skull was provisionally referred to *Sarahsaurus aurifontanalis*.

Below, we present a more detailed description of the holotype and paratype specimens of *Sarahsaurus aurifontanalis* and a revised description of the Rock Head specimen. These are based on more fully prepared specimens and on high-resolution X-ray computed tomographic (CT) and micro-computed tomography (μCT) data from cranial elements of both the holotype and the Rock Head specimens. Additionally, we discuss aspects of the referral of the Rock Head specimen to *Sarahsaurus aurifontanalis* that are typical problems that paleontologists commonly confront when using multiple specimens to score a single operational taxonomic unit (OTU). We explored this issue by performing a series of tests designed to evaluate the phylogenetic effects of restricting the matrix scores for *Sarahsaurus* to the holotype and compared those results to a composite score that includes the holotype, paratype, and Rock Head specimens.

We also note that since the initial description of *Sarahsaurus aurifontanalis*, a number of new Late Triassic and Jurassic sauropodomorphs have been described or re-described (e.g., [26–41]). Hence, a richer comparative context invites a reassessment of its initial diagnosis and systematic position.

Lastly, based on these new data and enriched comparative basis, we reexamine the question of whether the Early Jurassic North American sauropodomorphs comprise a unique clade unto themselves, or if they represent independent dispersals of early sauropodomorphs into North America. The answer to this question offers insights into general patterns and mechanisms that previous authors have postulated as mechanisms driving the early diversification of dinosaurs and Mesozoic terrestrial faunas in general, from a time spanning when Pangaea was intact through its early break-up in the Jurassic [9, 10, 16, 17].

## Systematic paleontology

Dinosauria Owen, 1842 [42] sensu Sereno, 2005 [43]

Saurischia Seeley, 1887 [44] sensu Sereno, 2005 [43]

Sauropodomorpha Huene, 1932 [45] sensu Sereno, 2005 [43]

*Sarahsaurus aurifontanalis* Rowe, Sues, and Reisz, 2010 [25]

### Etymology

Named in honor of Mrs. Sarah and Dr. Ernest Butler, whose vision, generosity, and broad interests in the arts, science, and medicine have enriched Texas in many ways; specific epithet from ‘aurum’ (L., gold) and ‘fontanalis’ (L., of the spring), in reference to Gold Spring, Arizona, where the holotype was discovered [25].

### Holotype

TMM 43646–2 partially articulated skeleton including a basicranium, quadrate, frontal, prefrontal, and maxilla, articulated pre-caudal vertebral column, partially articulated and nearly complete tail, complete left pectoral girdle, both humeri, articulated left forelimb including articulated manus, complete sacrum, both femora, articulated left tibia, fibula, tarsus, and pes.

### Referred specimens

Paratype, TMM 43646–3, partial skeleton including separated centra and neural arches, an uncrushed scapulae and right coracoid, left ilium and ischium, both pubes, right femur, left tibia and fibula, and right tarsus and pes; MCZ 8893, articulated skull with fragmentary postcranial elements, including atlantal neural arches, partial cervical neural arch, three partial caudal centra, partial distal humerus, incomplete femoral shaft, and fragments of gastralia. The two referred specimens differ in skeletal maturity at their times of death from the holotype (detailed below), and these differences introduce a measure of uncertainty regarding their systematic identity. As explained below, we performed a series of tests to identify and constrain this uncertainty with respect to their taxonomic identity and effects on phylogenetic analyses.

### Horizon and locality

Silty Facies of the Kayenta Formation, Glen Canyon Group, Pliensbachian [46]; TMM 43646 (holotype locality), Gold Spring, Coconino County, Navajo Nation, Arizona; MCZ field number 20/78AR (referred locality), Rock Head, Coconino County, Navajo Nation, Arizona. Detailed locality information is on file at the Vertebrate Paleontology Laboratory in Austin, TX as well as the Navajo Nation Minerals Department in Window Rock, AZ and is available to qualified researchers. All necessary permits were obtained for the described study, which complied with all relevant regulations.

## Materials and methods

### Geological setting of the specimens

**Fig 1:**
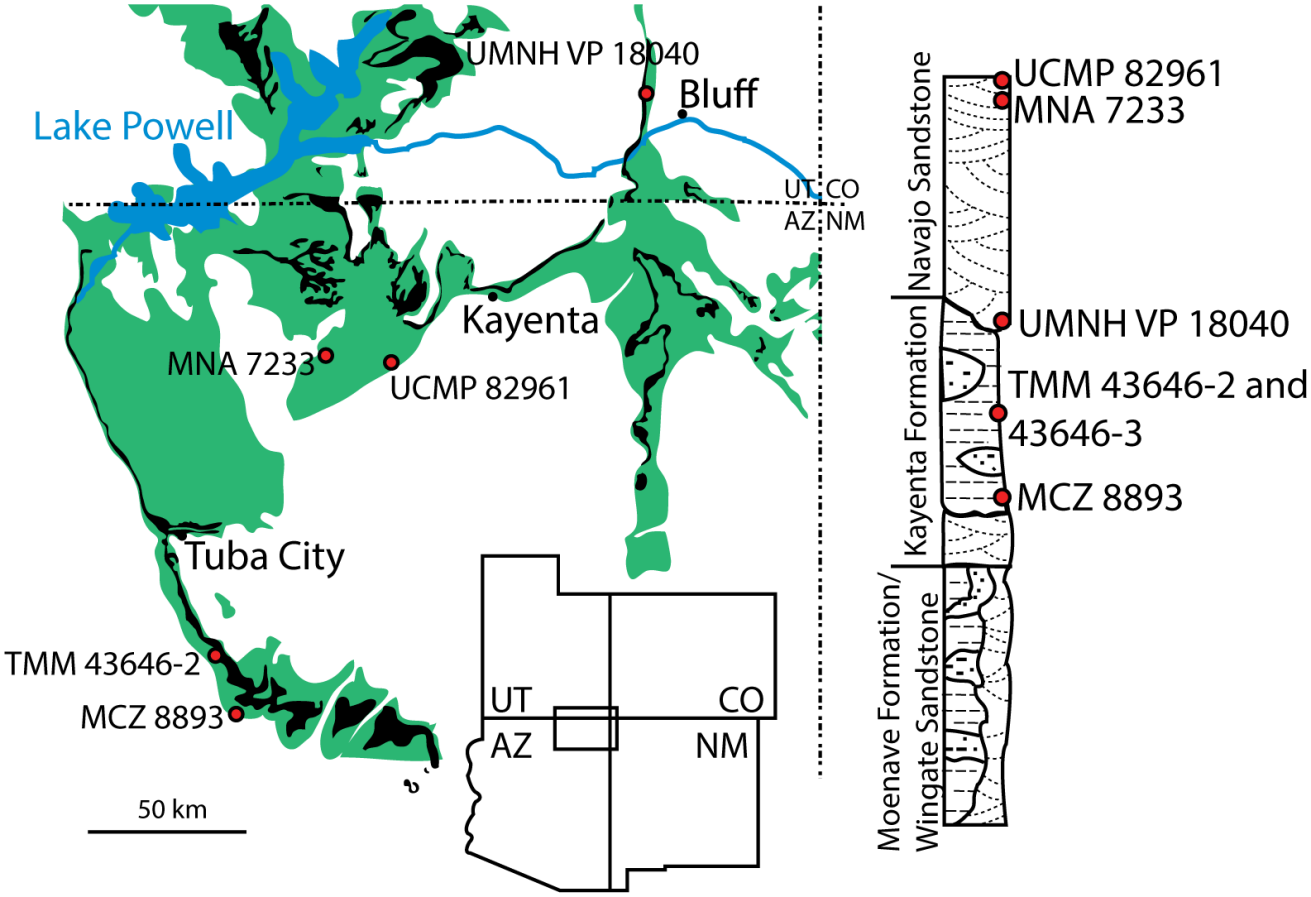
Geographic distribution of the holotype (TMM 43646–2), paratype (TMM 43646–3), and referred specimen (MCZ 8893) of *Sarahsaurus aurifontanalis* within the Glen Canyon Group (green) and Kayenta Formation (black) of northern Arizona and southern Utah. The stratigraphic column to the right is an idealized representation of the units from which each specimen was recovered (modified from [22]).

All three of the specimens in question were collected from the Silty Facies of the Kayenta Formation near its southwestern-most exposure (Fig 1). The Silty Facies is broadly exposed in Moenkopi Wash and to the south over a distance of roughly 60 kilometers. North of Moenkopi Wash, the Silty Facies grades into the sandier Typical Facies of the Kayenta Formation, which forms the towering Vermillion Cliffs that extend northwards into Utah. The sandier sediments of the Typical Facies are poorly fossiliferous, with the exception of the Comb Ridge locality, near the type section of the Kayenta Formation at the town of Kayenta, Arizona [47, 48].

Productive exposures of the Silty Facies are mostly confined to Moenkopi Wash, and to a line of cliffs extending to the south that is formed by the Navajo Sandstone and Kayenta Formation. This escarpment bears various names, including ‘Adeii Eechii Cliffs’ [49, 50], the ‘Tloi Eechii Cliffs’ (e.g., [51]), and the Echo Cliffs (e.g., [52]), names alluding to the red escarpment formed by the prominent cliff-forming Dinosaur Canyon Member of the Moenave Formation along this portion of Ward’s Terrace. South of Moenkopi Wash, the Adeii Eechii escarpment delineates both the eastern flank of the Little Colorado River valley and the western edge of the Moenkopi Plateau. Below the Adeii Eechii Cliffs is Ward Terrace, a physiographic feature made famous for extensive deposits of Triassic vertebrates recovered from the Moenkopi Formation, Chinle Formation, and Moenave Formation (e.g., [53]). From Moenkopi Wash southwards, exposure of the Silty Facies of the Kayenta is rather steep and offers relatively little surface area for prospecting, although a few fossils have been recovered from these exposures (e.g., [54]). However, expansive, and heavily-dissected badlands of the Silty Facies of the Kayenta Formation are exposed along the southern-most extent of the Adeii Eechii Cliffs on a broad bench formed by the thickening of the underlying Dinosaur Canyon Member of the Moenave Formation, which broadens Ward’s Terrace considerably to the west. Since the time of the joint MNA-MCZ expeditions in the 1970s, this has been the most productive part of the Kayenta Formation [18, 55, 56, 57].

This study is based on three specimens collected from lands of the Navajo Nation in northeastern Arizona, under permits granted by the Navajo Nation Minerals Division to the MNA and to the Vertebrate Paleontology Laboratory of the University of Texas (VPL) in collaboration with the Navajo EcoScouts program. Of primary importance herein are the holotype and paratype specimens of *Sarahsaurus aurifontanalis* that were collected from a single quarry (locality TMM 43646; Fig 1) that also included a partial skeleton of an immature individual of *Dilophosaurus wetherilli* [58, 59]. The stratigraphy of the quarry and partial disarticulation of each individual complicated identification and association of the individual elements. The *Dilophosaurus* bones were mostly situated above the holotype skeleton of *Sarahsaurus aurifontanalis*, which in turn lay above the paratype. However, it became evident during the excavation that some mixing of the elements had occurred post-mortem, perhaps as a result of scavenging as evidenced by bite marks on many of the bones (see below). Further mixing of the elements may have occurred during post-mortem transport, although partial articulation of the *Dilophosaurus* and holotype skeleton of *Sarahsaurus* suggest that the transport distance was short. There is no unequivocal evidence that the holotype quarry contained more than these three individuals, however we acknowledge that about two-dozen of the recovered bones remain unidentified, and we cannot exclude the possibility that one or more additional individuals and/or taxa were buried at this site. Appendix A (S1 Text) itemizes all the individual bones recognized as the holotype and paratype of *Sarahsaurus aurifontanalis*.

A third specimen potentially referable to *Sarahsaurus aurifontanalis* (MCZ 8893) [8, 15] was found near the base of a physiographic feature known as Rock Head. The Rock Head specimen was discovered in 1978 by TBR and collected by Farish A. Jenkins, Jr. and William Amaral. The skull was found in association with a smalll number of postcranial fragments that had weathered onto the surface. They include possible atlantal neural arch fragments, a partial cervical neural arch with small epipophysis that extends to back (but not beyond) the post zygapophyseal facets. This neural arch resembles that of the holotype of *Sarahsaurus aurifontanalis*. Also present is a neural spine possibly from the second cervical vertebra; an elongate cervical prezygapophysis that also resembles the holotype cervicals of *Sarahsaurus aurifontanalis*; and two or possibly three partial distal caudal centra. A heavily weathered distal end of a humerus and a partial femoral shaft were also recovered, along with a number of fragmented gastralia. These were all that could be located of the postcranium during excavation of the Rock Head specimen in 1978; no other fragments were found when the site was revisited in 2007.

The Rock Head skull was found less than a kilometer from the holotype locality of the ornithischian *Scutellosaurus lawleri* [55], 1.5 kilometers from the type locality of the theropod *Syntarsus kayentakatae* [56, 60], and 8.5 kilometers to the south of the type locality of *Sarahsaurus aurifontanalis* (TMM 43646). The Rock Head specimen was preserved lower in section than the holotypes of *Sarahsaurus aurifontanalis*, *Scutellosaurus lawleri*, and *Syntarsus kayentakatae*.

**Fig 2:**
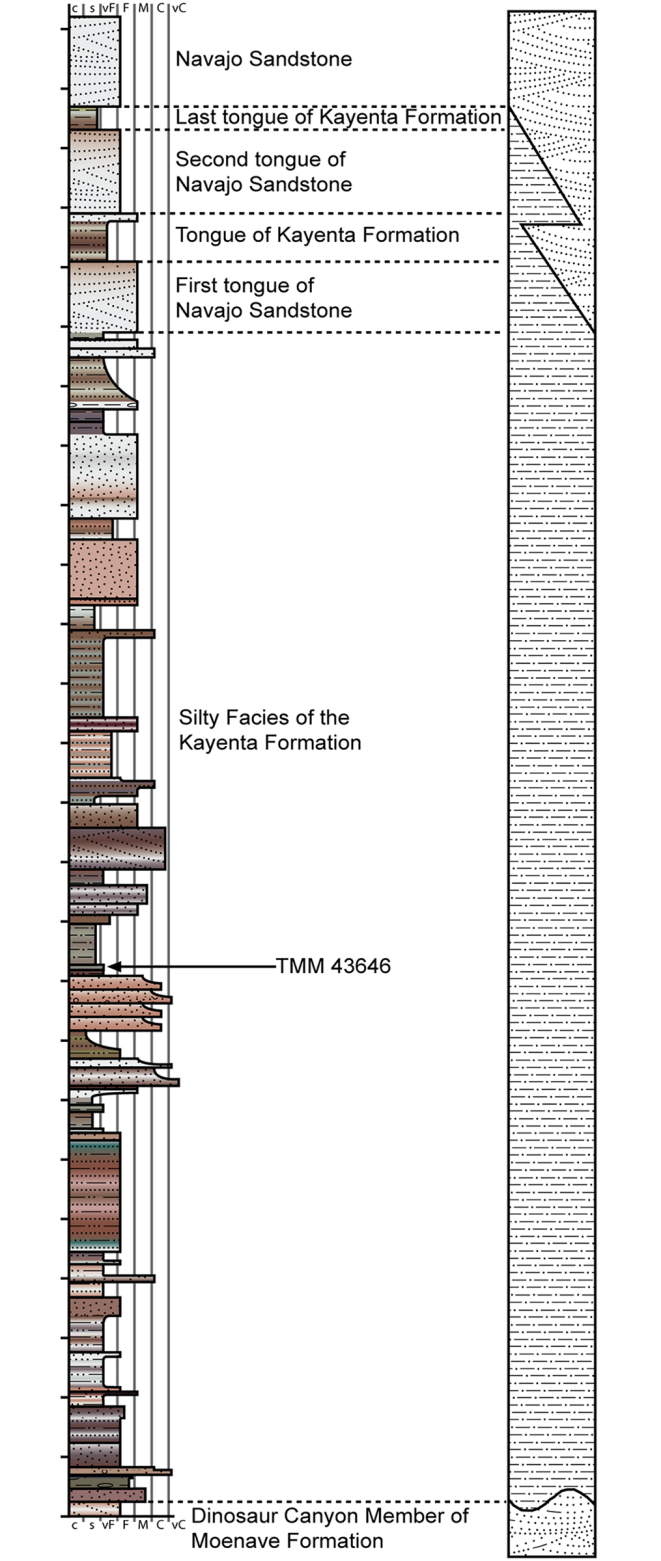
Stratigraphic section of the Kayenta Formation at Gold Spring, AZ. Vertical increments are 5 meters.

All three of the sauropodomorph specimens discussed herein were collected from the broad bench of the silty Kayenta Formation along the Adeii Eechii Cliffs. The *Sarahsaurus aurifontanalis* holotype quarry is located in the stratigraphic middle-third of the Kayenta Formation, along the northern-most flank of the Gold Spring drainage basin (Fig 2). Detrital zircon crystals were recovered from the matrix of the holotype and paratype during preparation. Unpublished U-Pb dates based on these detrital zircon crystals corroborate a late Pliensbachian age for the holotype quarry [46]. Figs 1 and 2 depict the geographic and stratigraphic relationships of the localities with respect to the other sauropodomorph remains discovered in the Glen Canyon Group of the Colorado Plateau.

### Specimen excavation and preparation

All three specimens were collected with hand tools and consolidated using Glyptol (MCZ 8893), Butvar, and cyanoacrylate (TMM 43636–2 and 43646–3). They were manually prepared using both hand tools and pneumatic tools. Many of the bones were encrusted with a resilient, red-purple-black oxide coating that we informally referred to as ‘hematite’ although we did not analyze its composition (but see [61]). This secondary crust, possibly biogenic in it origin [62], was harder than the bone, and to some degree it was responsible for the fine surficial detail preserved on many of the elements. However, removal of this crust was necessary to study the material and this was an exceedingly slow and laborious procedure. In many cases, elements were removed from field jackets and then prepared with delicate pneumatic percussion hammers beneath a microscope.

**Fig 3:**
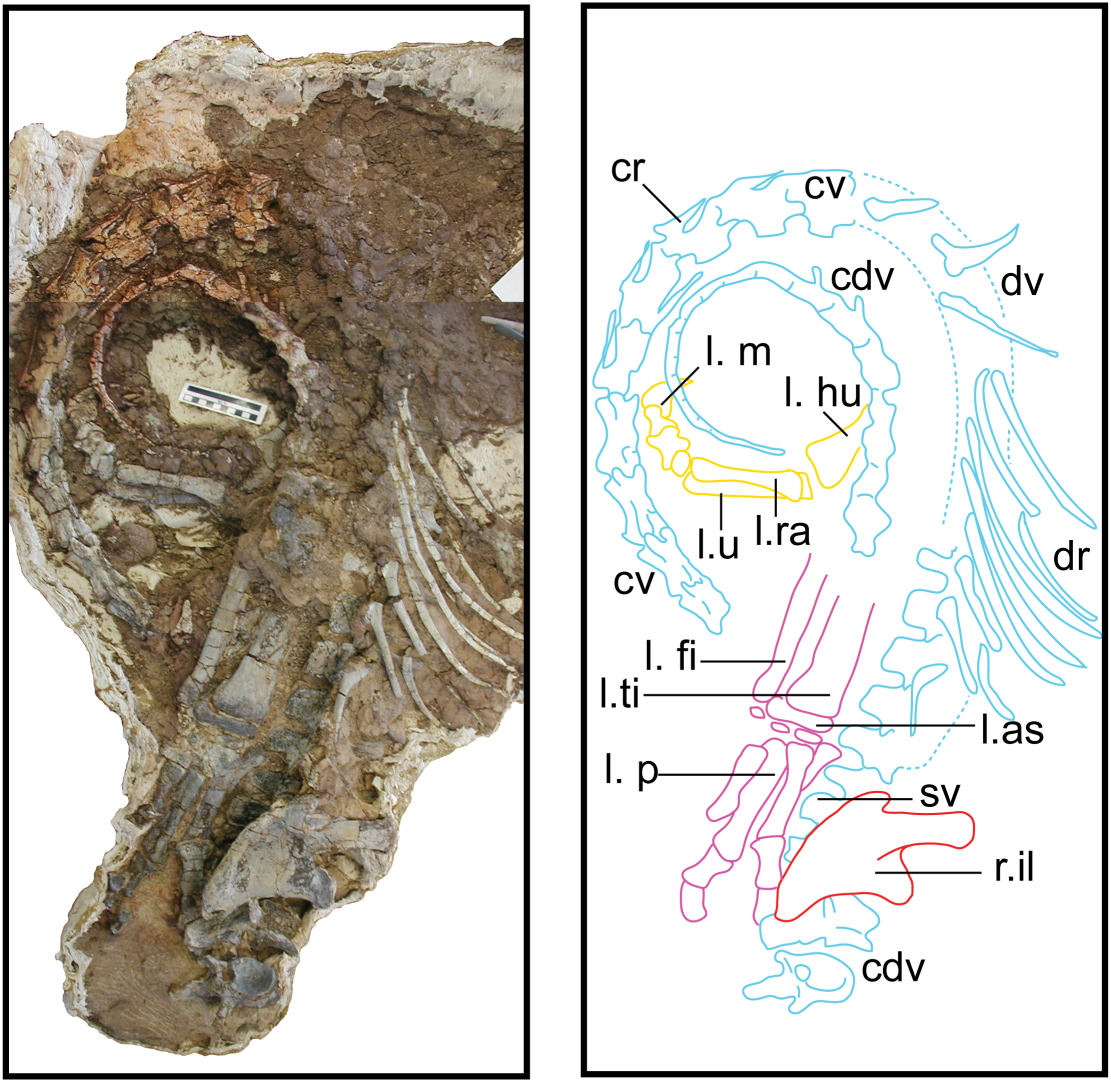
Main block from TMM 43646 containing much of the holotype specimen of *Sarahsaurus aurifontanalis*. Abbreviations: caudal vertebra (cdv), cervical rib (cr), cervical vertebra (cv), dorsal rib (dr), dorsal vertebra (dv), left astragalus (l. as), left fibula (l. fi), left humerus (l. hu), left pes (l. p), left radius (l. ra), left tibia (l. ti), left ulna (l. u), right ilium (r. il), sacral vertebra (sv). Scale bar in photograph is 10 cm.

Fig 3 displays the main field jacket surrounding most of the semi-articulated postcranial skeleton of the holotype specimen of *Sarahsaurus aurifontanalis*. Possible tooth marks scattered throughout the skeleton suggest that the effects of post-mortem scavenging were exacerbated by post-burial crushing. The anterior half of the presacral vertebral column is lateromedially flattened, and the femora are also flattened. Details regarding the deformation of specific skeletal elements are provided in the following description.

### Computed tomography

High-resolution X-ray computed tomographic (HRXCT) datasets were generated for the hand and braincase of the holotype as part of its initial description [25]. More recently, we scanned the Rock Head skull and three possible palatal teeth to augment the present analyses. All datasets were generrated at The University of Texas High-Resolution X-ray Computed Tomography Facility (UTCT) and are archived there. Scanning specifications for each dataset are presented in Appendix B (S2 Text), and full-resolution datasets in 8bit JPEG format are available for download at: http://digimorph.org/specimens/Sarahsaurus_aurifontanalis/.

In addition, movies made from these datasets are included here as supplemental data (S1 to S21 Animations, see Appendix B, S2 Text). They include three movies of the holotype braincase and inner ear, seven movies of the holotype hand and wrist, five movies of the referred Rock Head skull and jaws, and six movies of the possible palatal teeth that were recpovered from the Rock Head skull. All were generated using VG StudioMax, version 2.1.

### Comparative framework

In the following description, we compare the anatomy of *Sarahsaurus aurifontanalis* with that of many archosauriform taxa. We observed first-hand fossils of *Anchisaurus polyzelus*, *Buriolestes schultzi*, *Coelophysis bauri*, *Dilophosaurus wetherilli*, *Eoraptor lunensis*, *Euparkeria capensis*, *Herrerasaurus ischigualastensis*, *Massospondylus carinatus*, *Plateosaurus engelhardti*, *Psittacosaurus mongoliensis*, *Saturnalia tupiniquim*, *Staurikosaurus pricei*, and *Syntarsus kayentakatae*. We also consulted photographs of *Adeopapposaurus mognai*, *Coloradisaurus brevis*, *Guaibasaurus candalarensis*, *Mussaurus patagonicus*, *Pampadromaeus barbarenai*, and *Seitaad ruessi*. All other comparisons and character scorings were made from the literature. Certain elements of the holotype and paratype specimens of *Sarahsaurus aurifontanalis* were determined to be relatively uncrushed and were used to take linear measurements using Pittsburg digital calipers (Appendix C, S3 Text).

### Phylogenetic analyses

We rescored the holotype specimen of *Sarahsaurus aurifontanalis* from the CT data and more fully-prepared holotype skeleton (TMM 43646–2) using versions of the Yates [63] and Upchurch et al. [64] taxon-character matrices for early sauropodomorphs, as further modified by Yates et al. [33] and Apaldetti et al. [27, 28]. We did not change character scores for any taxon other than *Sarahsaurus aurifontanalis*. One character was added to both matrices: Number of foramina in proximal portion of pubis; one (0), two (1). The final matrices derived from Yates [63] and Upchurch et al. [64] have 52 taxa and 363 characters, and 39 taxa and 302 characters, respectively. The matrices were constructed in Mesquite [65] and the phylogenetic analyses were conducted in TNT [66, 67]. We also scored the holotype specimen of *Sarahsaurus aurifontanalis* in a more recent phylogenetic matrix constructed by McPhee et al. [37] and modified by McPhee and Choiniere [38]; the same character was added to this matrix as the other matrices described above. That matrix contains 61 taxa and 365 characters.

Heuristic searches in TNT utilized 1000 replications, tree bisection and reconnection, and random sequence additions while keeping ten trees per replication and collapsing zero-length branches. Bremer support and bootstrap resampling of 1000 replications were also conducted in TNT. Outgroup constraints were not enforced but the trees were rooted on *Euperkeria capensis* (Yates matrix and McPhee and Choiniere matrix) and *Marasuchus lilloensis* (Upchurch et al. matrix). All characters were treated as unordered in the Yates and Upchurch et al. matrices to closely replicate the initial analyses [25]. Characters 8, 13, 19, 23, 40, 57, 69, 92, 102, 117, 121, 131, 134, 145, 148, 150, 151, 158, 163, 168, 171, 178, 185, 208, 211, 218, 226, 231, 238, 246, 254, 257, 270, 282, 303, 309, 317, 337, 350, 353, 355, 360, and 364 were ordered in the McPhee and Choiniere matrix [38]. Complete character descriptions are found in Appendix D (S4 Text) and matrices are available as TNT files in Appendix E (S1 Files).

Separate sensitivity analyses were conducted using the Yates and Upchurch et al. matrices [63, 64] as well as the McPhee and Choiniere matrix [38]. One analysis followed a long-standing but little-tested practice in paleontology of combining multiple specimens into a single operational taxonomic unit (OTU), in order to gain the most complete characte-state scores for each taxon in an analysis. In generating composite OTUs, the basis for referral of specimens is rarely described. Gauthier et al. [68] acknowledged the potential of mistaken association. They also cited problematic instances where tree toppology changes depending on whether or not potentially separate OTUs are combined. Details of each sensitivity analysis are described below.

#### Testing referral of the Rock Head specimen (MCZ 8893) to *Sarahsaurus aurifontanalis*

The initial publication on the Rock Head specimen described only the skull and identified it as *Massospondylus* sp. [15]. Subsequent researchers questioned this identification and the specimen came to be known as the ‘undescribed Kayenta prosauropod’ [19, 20]. This specimen was provisionally referred to *Sarahsaurus aurifontanalis* in its initial description [25], and it was used to substantially augment the cranial description and matrix score for *Sarahsaurus aurifontanalis* in the initial phylogenetic analyses.

We note that the Rock Head specimen represents an individual skeletally less mature at time of death than the holotype specimen. In its terminal maturity, the Rock Head specimen is probably closer to the paratype than to the holotype, but because the papratype completely lacks cranial remains a direct comparison between the two cannot be made.

Compared to the holotype, immaturity at time of death in the Rock Head skull is indicated by separation of the exoccipitals from the basioccipital along their sutural contacts. In the holotype braincase these elements externally seem co-ossified to one another, however CT imagery shows that complete internal fusion had yet to occur. The Rock Head braincase is also slightly smaller than the holotype. Additional features pointing to immaturity of the Rock Head specimen include separation of distinct right and left centers of ossification of the supraoccipital; the presence of an open fontanelle on the dorsal midline between the supraoccipitals and parietals; and lack of fusion of between the parietals. CT imagery also shows the right and left frontals to abut on the midline in a flat plane, and that the interdigitating sutural relations observed between more mature frontals had not yet begun at time of death. In skeletally mature specimens, all of these elements suture tightly and can coossify in a familiar pattern reported in other dinosaurs [69].

Circumstantial evidence supporting referral of the Rock Head specimen to *Sarahsaurus aurifontanalis* includes the narrow stratigraphic and geographic ranges where they occur in the southern exposures of the Silty Facies of the Kayenta Formation. Moreover, insofar as they can be compared, every element of the local faunas from the Rock Head and Gold Spring collecting fields are taxonomically identical, although the fine-grained mudstones of the latter preserve a greater diversity of taxa.

In referring the Rock Head specimen to *Sarahsaurus aurifontanalis*, the initial study [25] used the lists of character-states compiled by Yates [63] and by Upchurch et al. [64] as a basis for comparison with the holotype. The two specimens compared favorably in all observable character states, apart from bone-to-bone fusions attributable to their differential maturities at time of death. However, the initial description also found that all but one of these many points of favorable comparison represented plesiomorphic character states. The one autapomorphy found at that time in common between the Rock Head specimen and the holotype was a unique configuration in the low wall between the basitubera in which there is a central anterior fossa. The analyses presented below suggest that this feature is more widely distributed and, considering what is now known of systematically significant variation among early sauropodomorphs, there there are no preserved autapomorphies linking the Rock Head specimen to the holotype. However, it is equally significant that none of these analsyes have found character discordances which might suggest that they are different taxa.

For these reasons, and for others discused below, in the present study we chose to test the effects of referal of the Rock Head specimen to *Sarahsaurus aurifontanalis* on tree topology. We performed separate analyses in which we 1) scored only the holotype; 2) treated the Rock Head skull and the holotype specimen as separate OTUs; and 3) combined the scorings such that the Rock Head skull was used to augment the holotype. The results are discussed below.

## Description of TMM 43646–2

### Skull

Isolated cranial elements recovered from the type quarry of *Sarahsaurus aurifontanalis* were assigned to the holotype specimen of *Sarahsaurus aurifontanalis* based on their size, shape, and association with other elements from the holotype. Most notably, a basicranium was recovered next to the articulated cervical vertebrae of the holotype specimen (Fig 3). A single isolated tooth was recovered from the quarry is probably part of the holotpye. It is subconical, recurved, and resembles those of other early saurischians such as *Eoraptor lunensis* [70], *Buriolestes shultzi* [71], and *Pampadromaeus barberenai* [72]; however, it is unlike the subsymmetrical, leaf-shaped teeth of sauropodomorphs found in *Arcusaurus pereirabdalorum* [73], *Pulanesaurus eocollum* [37, 38], and *Xingxiulong chengi* [36].

#### Maxilla

**Fig 4:**
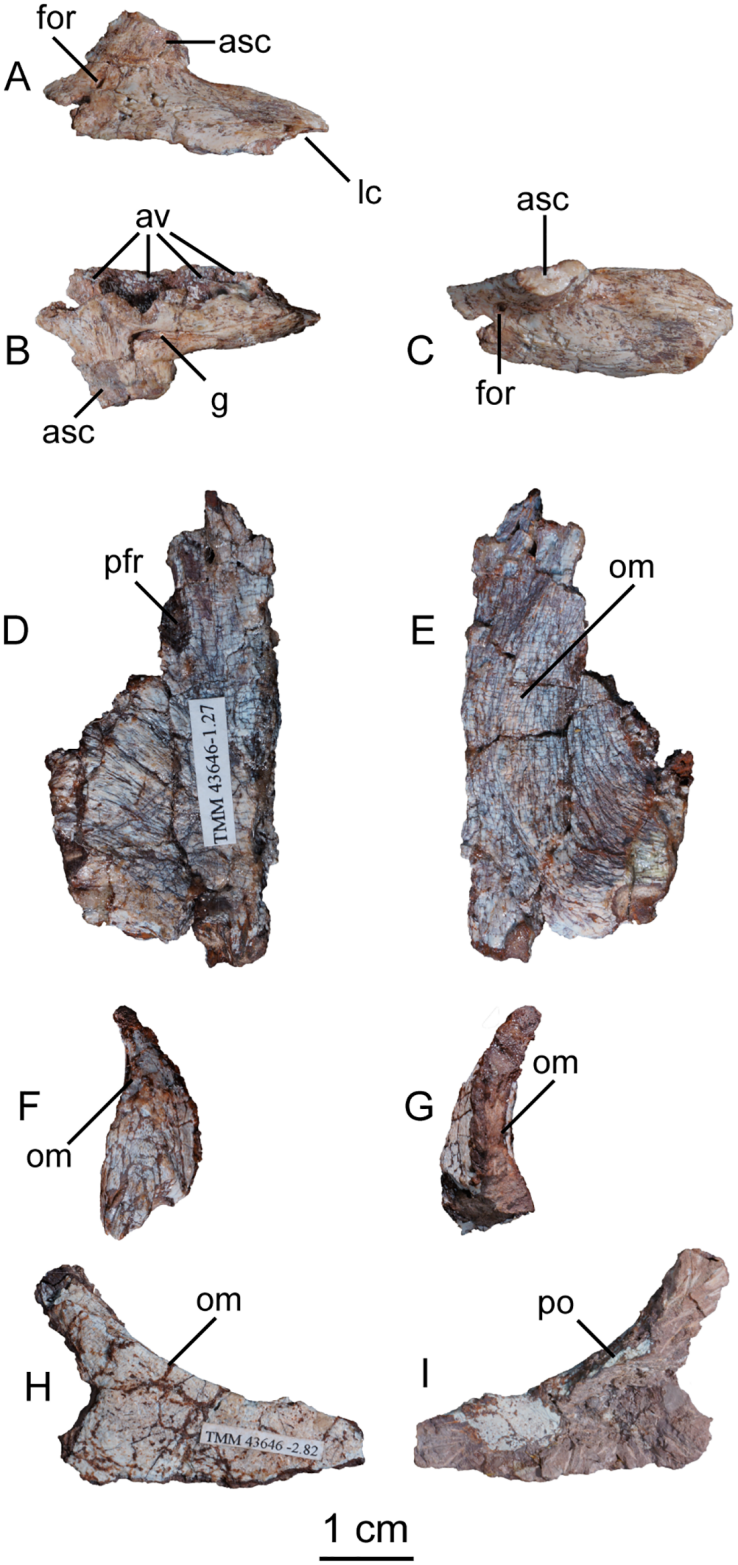
Holotype left maxilla (A-C), left frontal (D-E), left prefrontal (F-G), and right jugal (H-I) of *Sarahsaurus aurifontanalis*. A, F, H- lateral view; B, G, I- medial view; C, E- ventral view; D- dorsal view. Abbreviations: ascending process (asc), alveolus (av), foramen (for), groove (g), lateral condyle (lc), orbital margin (om), articulation with postorbital (po), articulation with prefrontal (prf).

A single left maxilla was found associated with the holotype specimen of *Sarahsaurus aurifontanalis* (Fig 4A–4C). This small bone is missing its anterior and posterior margins as well as the top of its ascending process. Viewed from above, the main body of the bone is thickest posteriorly near its articulation with the jugal. A foramen perforates the anterolateral surface of the maxilla where the ascending process meets the maxillary body. Only four maxillary alveoli are preserved; owing to its incompleteness the total number of maxillary teeth is unknown. The medial surface is flat. A rounded, inverted L-shaped groove extends along the medial surface from the base of the ascending process to just above the posterior margin of the second alveolus. A longer, straight strip of thin bone was found associated with the maxilla and may represent part of the vomer, but the incompleteness of the bone prevents conclusive identification.

#### Frontal

The left holotype frontal is incomplete along its posterior, posterolateral, and anterior margins (Fig 4D and 4E). The midline articular surface is serrated along the broken interdigitating suture with the other frontal. The supratemporal fossa occupies the dorsal surface of the frontal in saurischian dinosaurs [74] and its absence here is probably due to incompleteness of the posterior margin of the frontal [74, 75]. The supratemporal fossa extends onto the frontal in the Rock Head specimen. Anterolaterally, a shallow round notch accommodates the posterior articular surface of the prefrontal. Ventrally, a sharply defined semicircular ridge curves inward and approaches the midline along the length of the frontal, where it circumscribes the dorsomedial margins of the orbit.

#### Prefrontal

Only the bulbous anterior end of the left prefrontal is preserved in the holotype specimen (Fig 4F and 4G). The curved bone is arched slightly dorsally where it participated extensively in the anterior margin of the orbit. A short rostral process is directed anteroventrally.

#### Jugal

The only cranial element known from the right side of the holotype specimen of *Sarahsaurus aurifontanalis* is a fragmentary right jugal (Fig 4H and 4I). This thin, subtriangular bone preserves a postorbital ramus that is oriented posterodorsally, where it contributes to the anteroventral margin of the infratemporal fenestra, and the posteroventral margin of the orbit. The preserved portion of the jugal is lateromedially flat and is thickest medially at its articulation with the postorbital.

#### Quadrate

**Fig 5:**
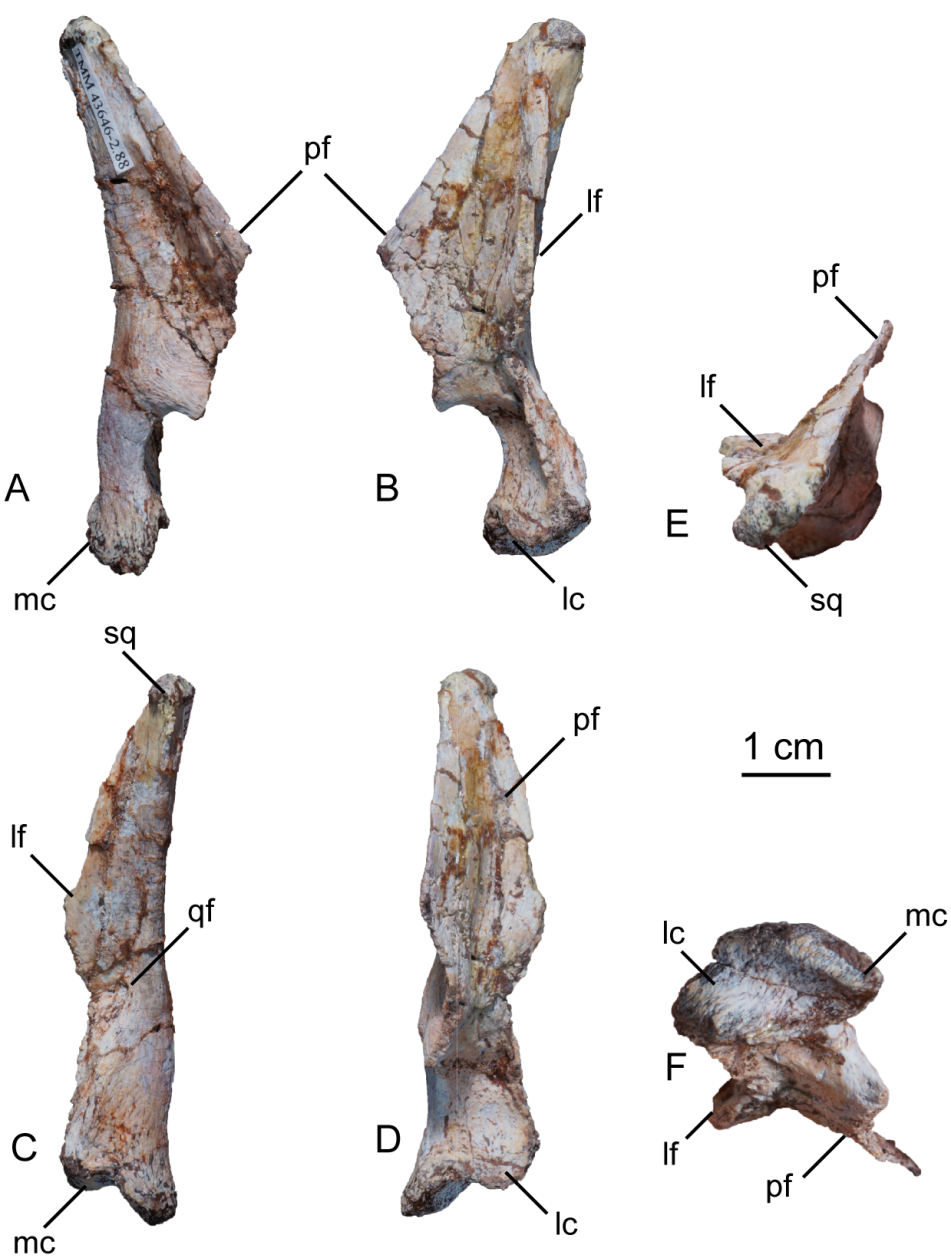
Holotype left quadrate of *Sarahsaurus aurifontanalis*. A- medial view; B- lateral view; C- posterior view; D- anterior view; E- dorsal view; F- ventral view. Abbreviations: lateral condyle (lc), lateral flange (lf), medial condyle (mc), pterygoid flange (pf), quadrate foramen (qf), articulation with squamosal (sq).

Only the left quadrate was preserved in the holotype specimen (Fig 5). It was buried close to the basicranium and anterior cervical vertebrae. The quadrate is dorsoventrally long and curves anteriorly near the ventral constriction of the shaft above its two condyles. The pterygoid and lateral flanges are visible in dorsal view. The two flanges come together at the top of the bone in a rugose articular surface for the squamosal. The lateral flange is broken along most of its length, but judging from its thickness at its base, it was not as prominent as the anteromedially-projecting pterygoid flange. The pterygoid flange is subtriangular in lateral view but is broken anteriorly. The pterygoid flange meets the ventral portion of the quadrate 80% down the length of the quadrate, where its margin is subcircular and hook-shaped. The quadrate foramen enters the main body of the quadrate near the ventral margin of the lateral flange. Distally, the quadrate of *Sarahsaurus aurifontanalis* expands mediolaterally into two distinct condyles that articulate with the lower jaw. The medial condyle is larger than the lateral condyle and projects further ventrally.

#### Basicranium and bony labyrinth

**Fig 6:**
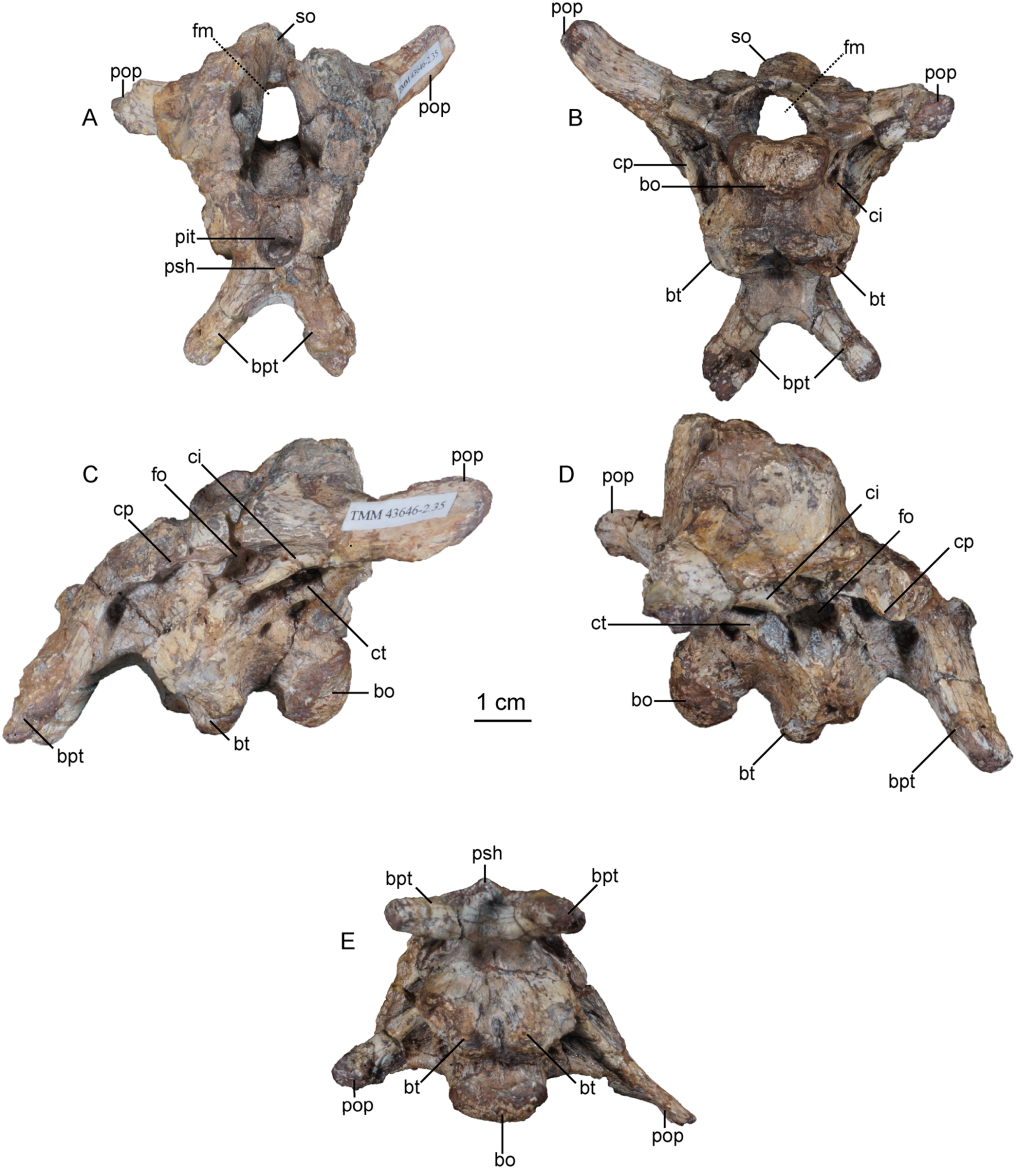
Color photographs of holotype braincase of *Sarahsaurus aurifontanalis*. A- anterior view; B- posterior view; C- left lateral view; D- right lateral view; E- ventral view. Abbreviations: basioccipital (bo), basipterygoid process (bpt), basal tuber (bt), crista interfenestralis (ci), crista prootica (cp), foramen magnum (fm), foramen ovale (fo), pituitary fossa (pit), paroccipital process (pop), parasphenoid rostrum (psh), supraoccipital (so).

**Fig 7:**
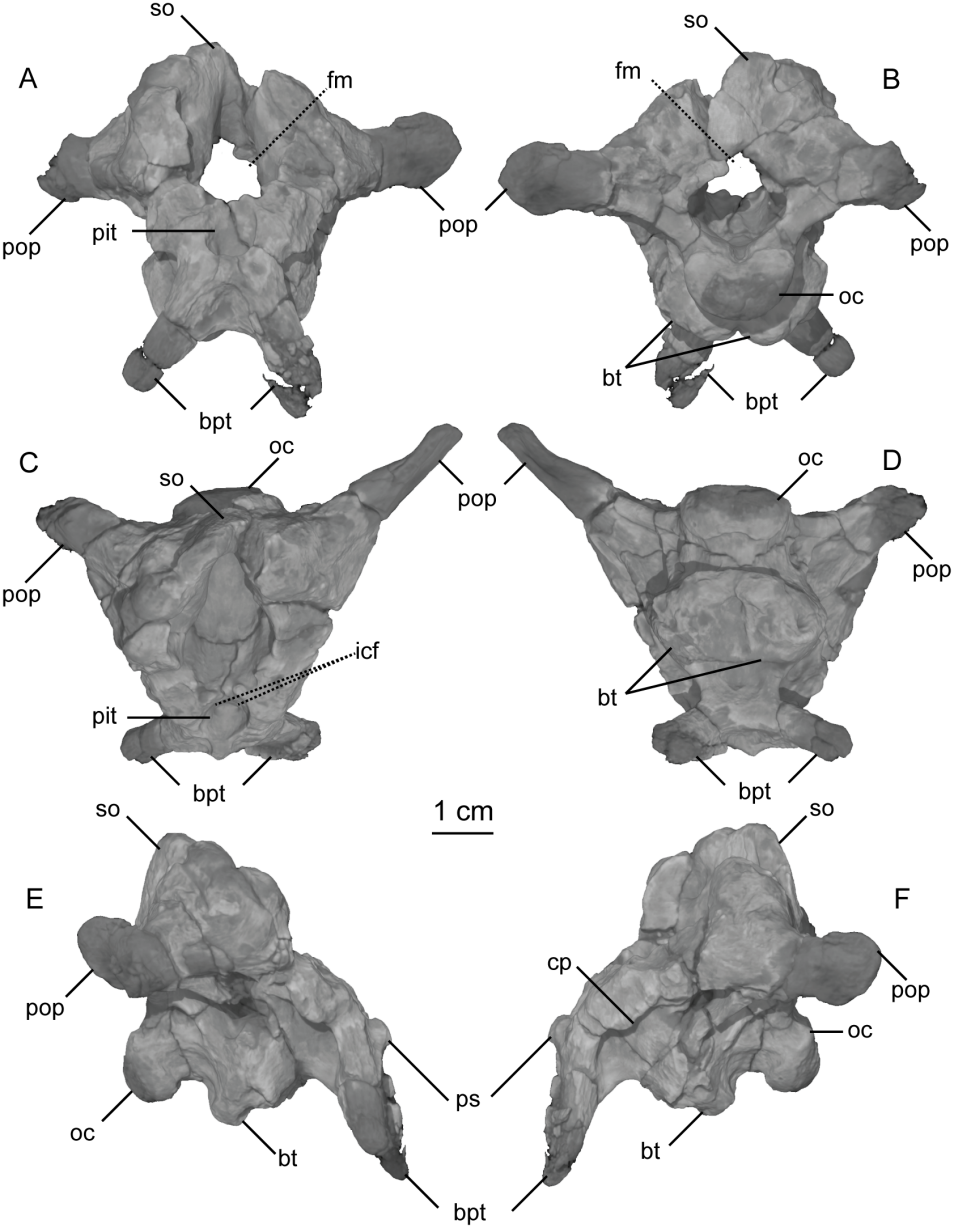
CT-volume-rendered images of the major structural features of holotype braincase of *Sarahsaurus aurifontanalis*. A- anterior view; B- posterior view; C- dorsal view; D- ventral view; E- right lateral view; F- left lateral view. Abbreviations: basipterygoid process (bpt), basal tuber (bt), crista prootica (cp), passage for internal carotid (icf), foramen magnum (fm), foramen ovale (fo), occipital condyle (oc), pituitary fossa (pit), paroccipital process (pop), parasphenoid rostrum (psh), supraoccipital (so).

**Fig 8:**
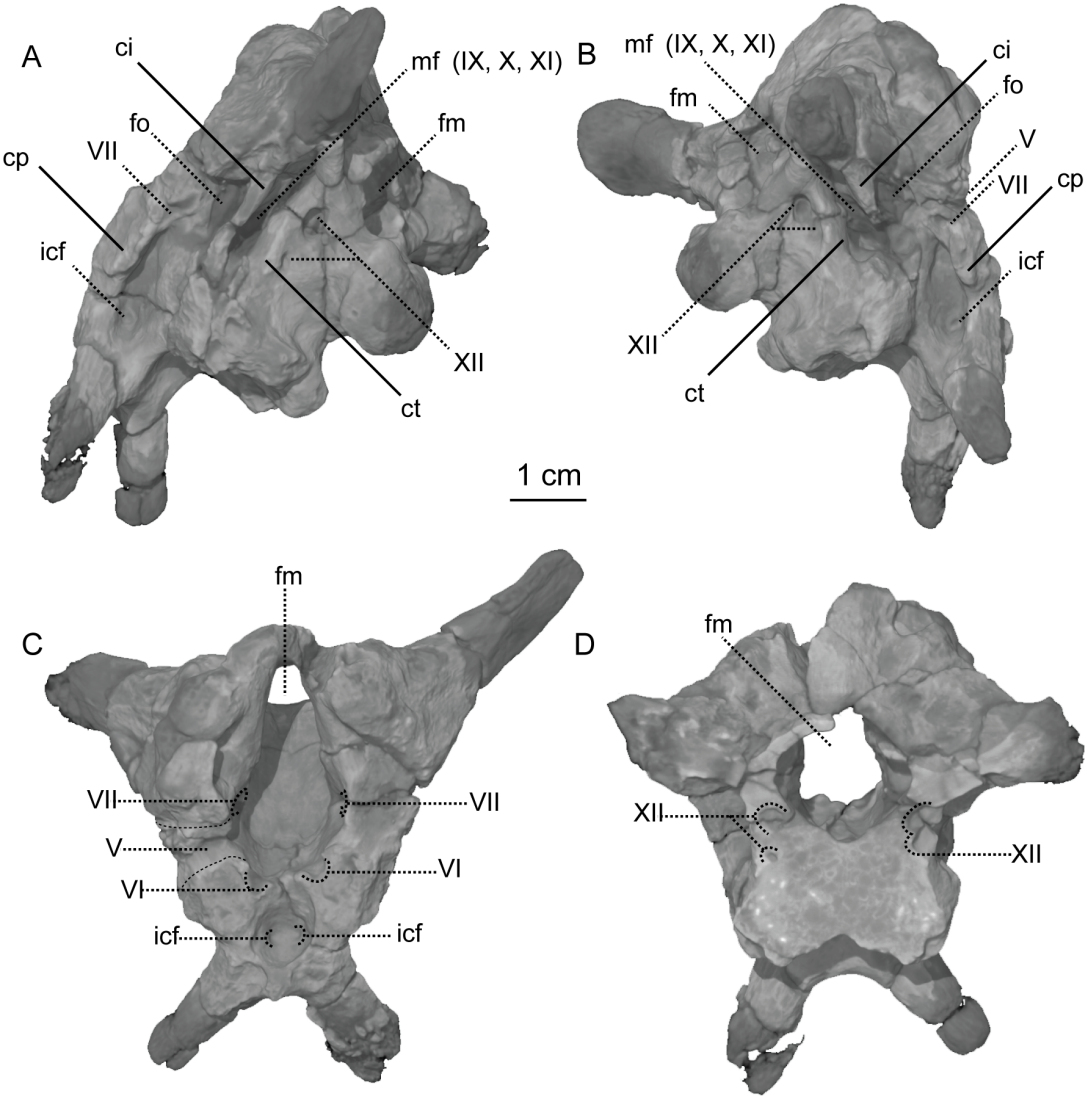
CT-volume-rendered images of the major openings for the cranial nerves and blood vessels of the holotype braincase of *Sarahsaurus aurifontanalis*. A- left posterolateral view; B- right posterolateral view; C- anterodorsal view; D- posterodorsal view (with occipital condyle, basal tubera, and roof of the foramen magnum removed to show the asymmetry in cranial nerve XII). Abbreviations: crista interfenestralis (ci), crista prootica (cp), crista tuberalis (ct), foramen magnum (fm), foramen ovale (fo), passage for internal carotid (icf), metotic fissure (mf), cranial nerves (V, VII, IX, X, XI, XII).

The braincase of the holotype specimen of *Sarahsaurus aurifontanalis* preserves the parasphenoid, basisphenoid, basioccipital, supraoccipital, prootics, opisthotics, and exoccipitals. Externally, with the exception of the exoccipital-basioccipital contact, these elements appear to be completely fused. However, sutures between most these elements can be distinguished in CT cross-sections. The braincase is missing the anterior rostrum of the parasphenoid, both laterosphenoids, the anterior half of the supraoccipital, and the anterior margin of the left prootic. Figs 6, 7 and 8 display external features of the braincase in color and CT images.

**Fig 9:**
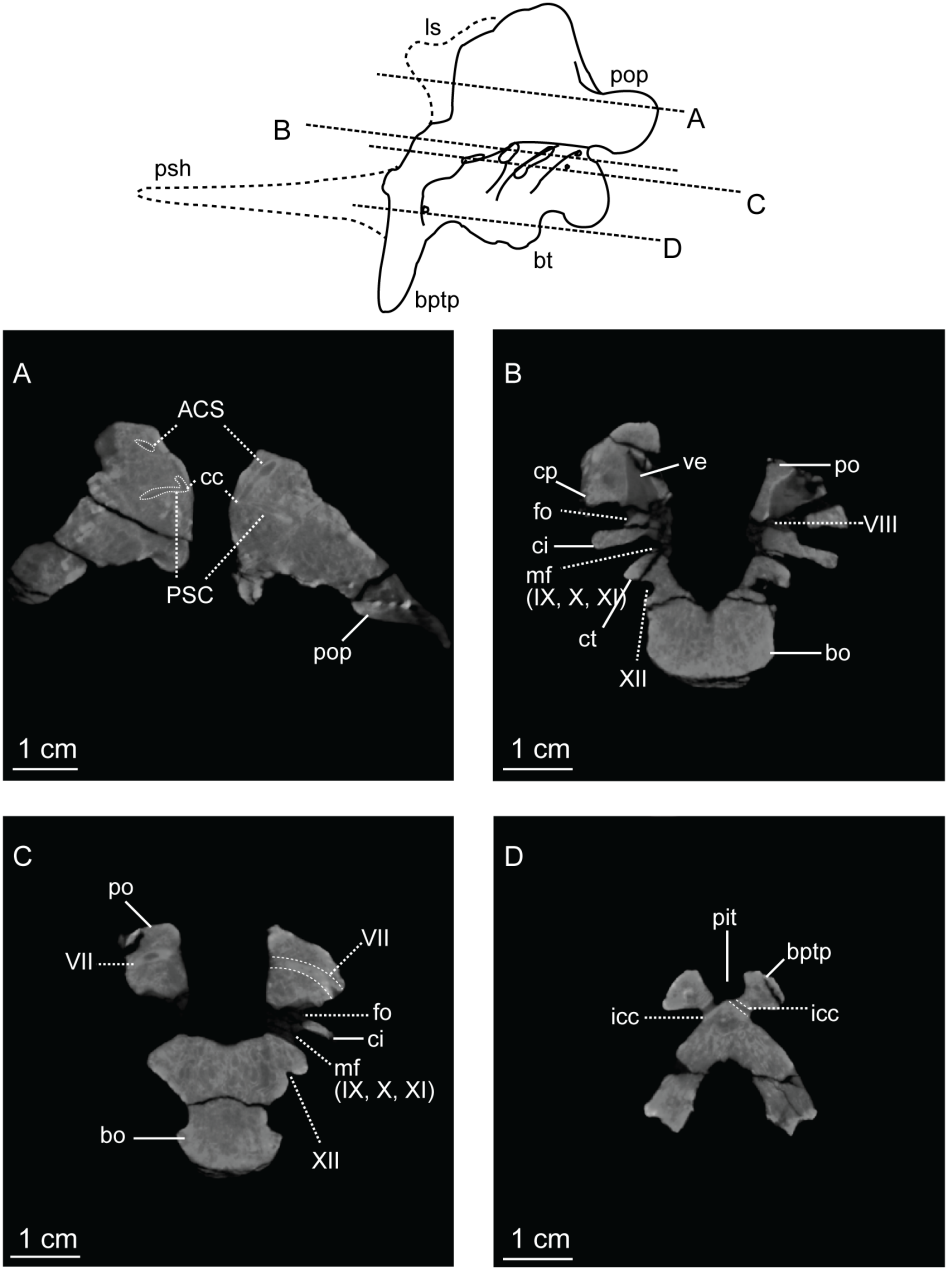
CT-cross-sections of four horizontal planes through the holotype braincase of *Sarahsaurus aurifontanalis*. A- Through the paroccipital processes; B- Through the major cristae and foramina of the braincase; C- Through the prootics and basioccipital; D- Through the basipterygoid processes. The top line drawing reconstructs the parasphenoid rostrum and laterosphenoid. Abbreviations: anterior semicircular canal (ASC), basioccipital (bo), basipterygoid process (bpt), basal tuber (bt), common crus (cc), crista interfenestralis (ci), crista prootica (cp), crista tuberalis (ct), passage for internal carotid (icf), foramen ovale (fo), laterosphenoid (ls), metotic fissure (mf), pituitary fossa (pit), prootic (po), paroccipital process (pop), posterior semicircular canal (PSC), parasphenoid rostrum (psh), vestibule (ve), cranial nerves (VII, VIII, IX, X, XI, XII).

The supraoccipital lies dorsal to the paroccipital processes where it is arched dorsally and inclined at approximately 45° to the horizontal plane. With the lateral semicircular canal oriented horizontally (Fig 9), the paroccipital processes have a slight ventral deflection in occipital view, similar to the dinosauromorph *Silesaurus opolensis* [76]. Lateral to the foramen magnum, the paroccipital processes become constricted and thier distal ends expand into terminal flat paddles. At the dorsal rim of the foramen magnum, the supraoccipital is thin, but the lateral rim of the foramen thickens laterally in the exoccipitals and opisthotics. A U-shaped midline channel for the spinal cord incises the dorsal surface of the basioccipital. It is bordered on either side by the exoccipitals, and all three bones contribute to the occipital condyle.

In lateral view, the basal tubera and the basipterygoid processes are aligned horizontally and the posterior portion of the parasphenoid rostrum is only slightly dorsal to that alignment. This condition is more like that of *Anchisaurus polyzelus* [7,8, 32], *Adeopapposaurus mognai* [77], *Lufengosaurus hueni* [78, 79], and *Massospondylus carinatus* [20, 80], than to *Saturnalia tupiniquim* [81], *Plateosaurus engelhardti* [31] and *Coloradisaurus brevis* [28] where the basal tubera are in a more dorsal position relative to the basipterygoid processes. The basipterygoid processes are not as long as the height of the braincase from the parasphenoid to the supraoccipital. The basipterygoid processes are long and thin in *Sarahsaurus aurifontanalis* and do not expand distally as in *Adeopapposaurus mognai* [77].

A subcylindrical groove separates the occipital condyle from the basal tubera. The basal tubera are well-developed and form a broad, transverse ridge on the ventral margin of the braincase. These tubera are subrectangular and are separated on the midline by a thin groove that opens anteriorly into a subtriangular rostral fossa. The ventral surface of the braincase thins medially behind this fossa at the inter-basipterygoidal space, houses a smooth, subelliptical depression before expanding into the paired basipterygoid processes. A thin web of bone connects the basipterygoid processes at their bases. This web of bone is roofed by the posterior portion of the parasphenoid rostrum anteriorly. A subelliptical pituitary fossa indents the dorsal surface of the basisphenoid. The internal carotid foramina penetrate the floor of the pituitary fossa on either side of the midline. The carotid canal extends through the basipterygoid process and exits laterally behind the crista prootica and extends up half the length of the paraoccipital process (Figs 8 and 9D).

A smooth rostral notch on the right prootic marks the more posterior opening for the trigeminal nerve (CN V). The abducens nerve (CN VI) exits the braincase near the floor of the braincase anterolateral to the pituitary fossa (Fig 8). Cranial nerve VII exits through an oblong foramen dorsal to the posterior internal carotid foramen, and behind the crista prootica on the lateral surface of the braincase (Fig 9C). The foramen ovale is subelliptical and is sandwiched between the crista prootica and the more posterior crista interfenestralis. Behind this is the elongate, rounded metotic fissure, and opening that is plesiomorphic for dinosaurs [75, 76, 82] and provides the exits for cranial nerves IX, X, and XI (Figs 8 and 9B). A low crista tuberalis separates the metotic fissure from the two hypoglossal foramina. The two branches of cranial nerve XII exit the interior wall of the braincase through these foramina. The larger of the two hypoglossal foramina is dorsal and posterior to the smaller foramen. The right hypoglossal foramina are much closer together (Fig 8D). This appears to be a natural case of bilateral asymmetry. Distortion of the right crista interfenestralis, crista prootica, and paroccipital process, appears to represent post-mortem deformation (Fig 8A and 8B).

**Fig 10:**
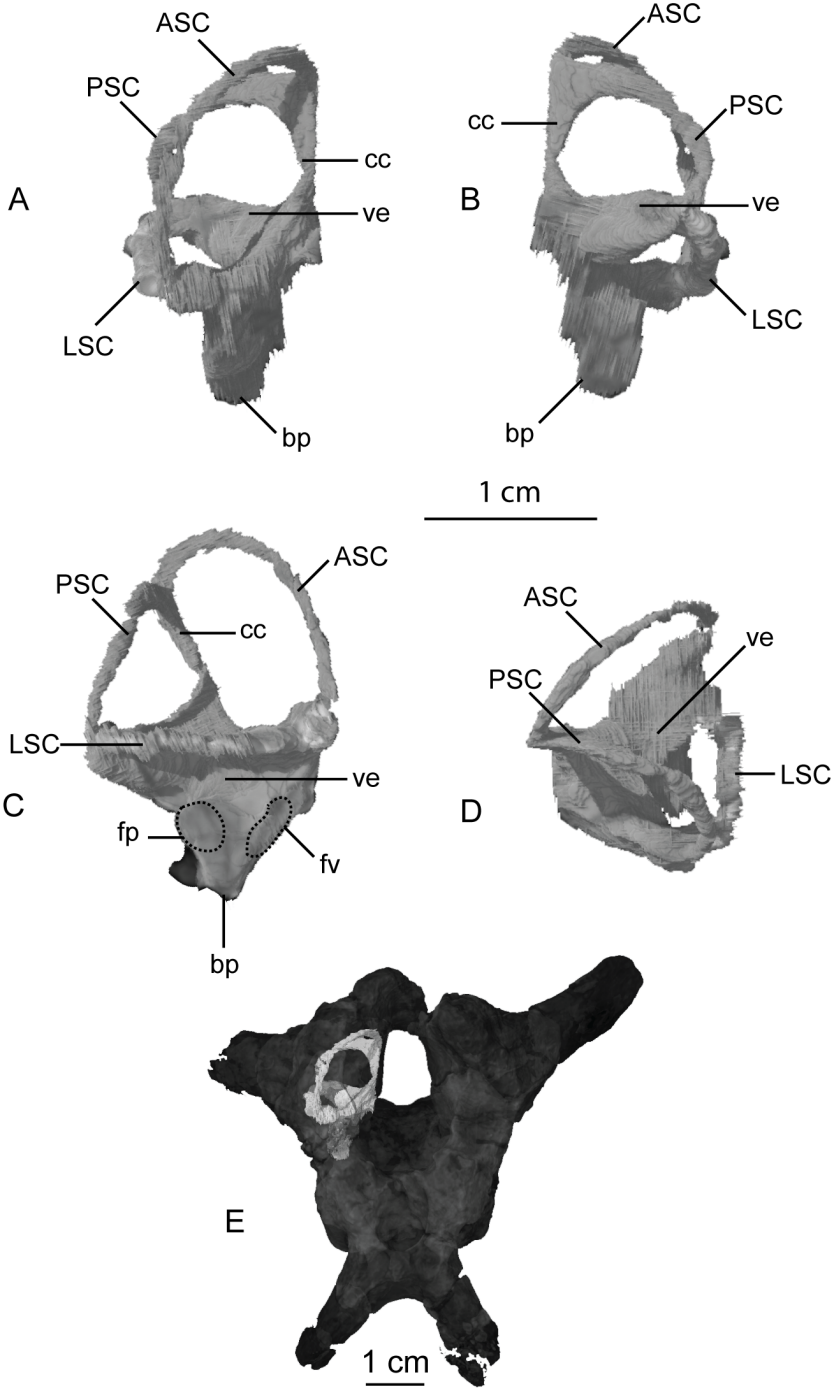
CT-volume-rendered images of the right inner ear labyrinth within the holotype braincase of *Sarahsaurus aurifontanalis*. A- anterior view; B- posterior view; C- right lateral view; D- dorsal view; E- Inner ear labyrinth in-situ within the braincase. Abbreviations: anterior semicircular canal (ASC), basilar papilla (bp), common crus (cc), fenestra pseudorotunda (fp), fenestra ovalis (fv), lateral semicircular canal (LSC), posterior semicircular canal (PSC), vestibule (ve).

The bony inner ear labyrinths are preserved in both sides of the braincase, although the left labyrinth is incomplete where the prootic is broken (Fig 9B). The internal auditory meatus (for CN VIII) connects the vestibule with the endocranial cavity (Fig 9C). Additionally, the bony labyrinth opens laterally through the side of the braincase via the foramen ovale. Of the three semicircular canals, the lateral canal is the shortest and curves the least (Fig 10). The lateral semicircular canal of *Sarahsaurus aurifontanalis* does not create a straight tube like that of *Spinophorosaurus nigerensis* [83], but curves laterally as in *Massospondylus carinatus* [80] and *Saturnalia tupiniquim* [81]. The anterior semicircular canal is the tallest and bows strongly anterolaterally. The posterior semicircular canal meets the anterior canal dorsally, and only the anterior canal continues dorsally beyond this point before turning ventrally. The base of the common crus forms a triangle where it enters the vestibule. The basilar papilla is small and subconical like that of *Massospondylus carinatus* [80, 83] and *Saturnalia tupiniquim* [81] unlike the longer basillar papilla of sauropods [29].

### Vertebral column

The holotype skeleton preserves every vertebra between the axis and the first caudal vertebra in articulation. A few other anterior caudal vertebrae were found associated with the posterior region of the sacrum, but most of the tail is articulated and separated from the rest of the vertebral column. We estimate that 10–12 caudal vertebrae were not preserved with the rest of the holotype owing to perimortem scavenging. The cervical and anterior dorsal vertebrae have undergone plastic deformation post-mortem. Pathological spondyloarthropy affected several of the vertebrae [25]. In the holotype, every centrum is fused to its neural arch, and the sutures between these elements are not visible externally. Mostly complete cervical and dorsal ribs were found in articulation on both sides of the body and in some cases are severely distorted. A few disarticulated anterior haemal arches were recovered, but most of the middle and posterior haemal arches remain in articulation with the caudal vertebrae. Finally, gastralia were found in nearly every block recovered from the quarry, but none seem to have been in articulation. The paratype individual also preserves disarticulated vertebral elements, but centra, neural arches, and sacral ribs are not fused to one another. Elements of the atlas either are fused to the anterior part of the axis or were found in association with the basicranium of the holotype individual. Including the atlas, *Sarahsaurus aurifontanalis* is estimated to have ten cervical, 14 dorsal, three sacral, and approximately 50 caudal vertebrae. The total length of the vertebral column exceeds 3.1 m.

#### Atlas-axis

**Fig 11:**
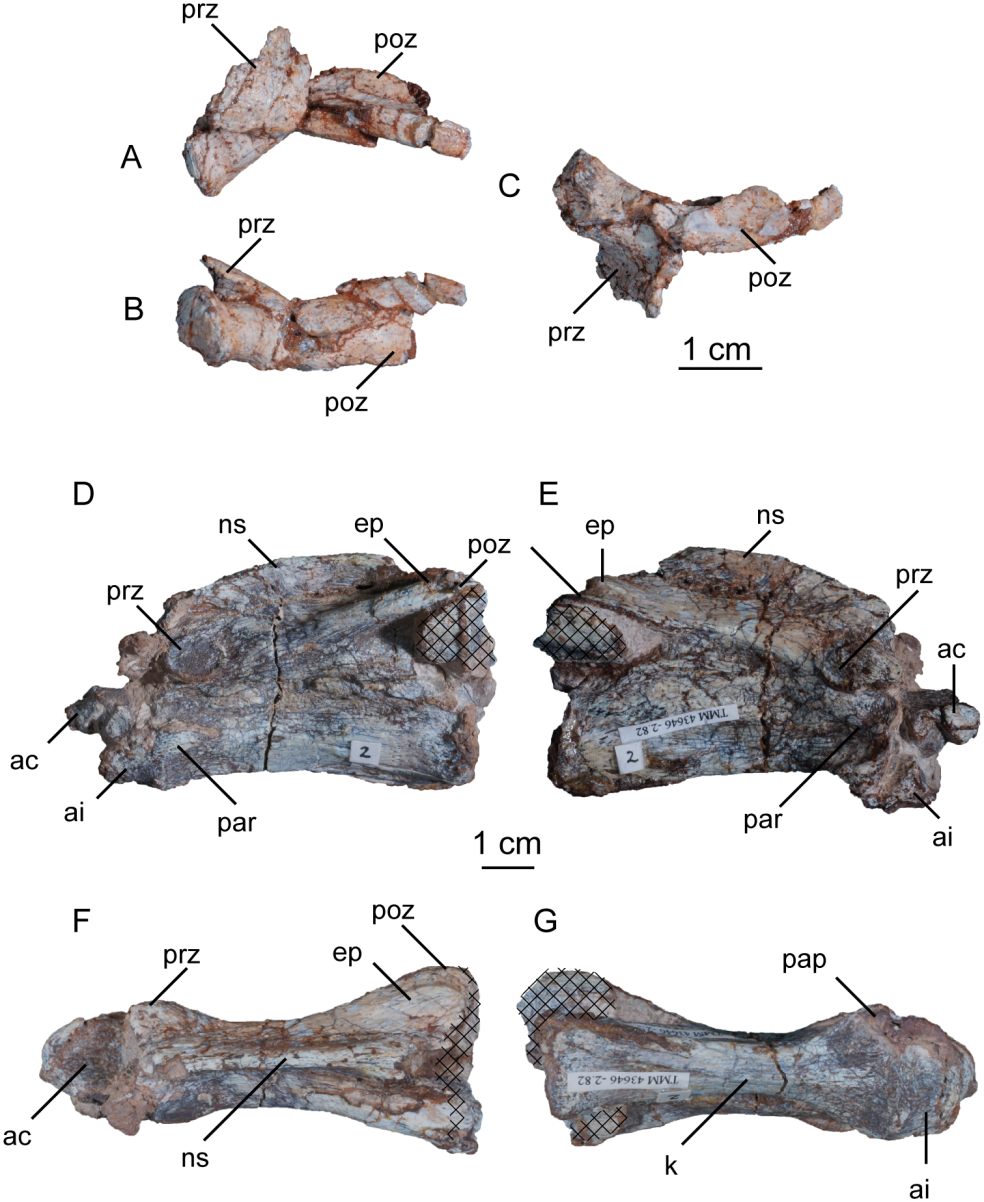
Holotype left atlantal neural arch (A-C) and atlantal centrum, axial intercentrum, and axial centrum (D-G) of *Sarahsaurus aurifontanalis*. A- lateral view; B- ventral view; C- medial view; D- left lateral view; E- right lateral view; F- ventral view; G- dorsal view. Hatched areas represent overlapping bone from preceding and/or succeeding elements. Abbreviations: atlantal centrum (ac), anterodorsal flange (adf), anteroventral process (avp), axial intercentrum (ai), epipophysis (ep), keel (k), neural spine (ns), parapophysis (par), postzygapophysis (poz), posterior process (pp), prezygapophysis (prz).

The proatlas of *Sarahsaurus aurifontanalis* is unknown. A single left atlantal neural arch is preserved in the holotype specimen (Fig 11A–11C). The atlantal intercentrum is missing. The atlantal centrum, or odontoid process, is articulated to the anterodorsal surface of the axial centrum, whose constituemnt parts are thoroughly fused (Fig 11D and 11E). Cervical ribs are missing from the first four vertebrae, but parapophyses on these centra indicate that ribs were present in life.

The atlantal neural arch closely resembles that of *Adeopapposaurus mognai* [77] and *Leyesaurus marayensis* [26] (Fig 11A–11C). Laterally, the neural arch clearly is made up of three major features: a posterior process, an anteroventral process, and a medially-directed anterodorsal flange. The subcylindrical posterior process tapers posteriorly and is fairly straight. The ventral margin of the posterior process becomes increasingly concave anteriorly, forming a broad subrectangular process that projects posteroventrally and articulates with the atlantal intercentrum. The lateral surface of this process is broad and convex and points anterolaterally. The thickest portion of the atlantal neural arch is its anteroventral process, which thins towards the dorsomedial flange and the posterior process.

The atlantal centrum is a subcircular bone with a slightly concave dorsal surface (Fig 11D). Ventrally, a transverse M-shaped groove divides the atlantal centrum into an anterior ridge and a wider bulbous posterior portion. This ventral groove travels dorsally slightly as it extends posterolaterally but does not reach the dorsal surface.

The axis comprises the fused axial centrum and neural arch, as well as a coossified axial intercentrum. That element is fused to the bottom of the front edge of the axial centrum and is lateromedially narrower than the axial centrum (Fig 11G). The intercentrum is wedge-shaped laterally with a concave ventral surface. The axial centrum is transversely concave ventrally and is dorsoventrally thinnest just posterior to the parapophysis at a level one-third along the length of the bone (Fig 11D–11G). A low keel extends longitudinally along the ventral midline. The axial centrum is at least three times as long as it is tall. The posterior margin of the centrum extends farther posteriorly than the postzygapophyses. The parapophyses are anteroposteriorly elongate tuberosities on the anteroventral margin of the centrum that abut the posterior rim of the axial intercentrum.

The synostosis between the axial centrum and intercentrum is a sharp, transverse ridge. The prezygapophyses are subelliptical and face dorsolaterally. In dorsal view, they expand laterally, and the neural arch thins just behind each of them. The neural arch expands gradually dorsolaterally, terminating in broad posterolaterally-facing postzygapophyses. Epipophyses projecting from the postzygapophyses form low, round processes. The axial neural spine extends from the anterior margins of the prezygapophysis and terminates anterior to the posterior end of the postzygapophysis. Posteriorly, the neural spine bifurcates as it transitions into the epipophyses. In lateral view, the neural spine is low, subrectangular, and slightly convex.

#### Post-axial cervical vertebrae

**Fig 12:**
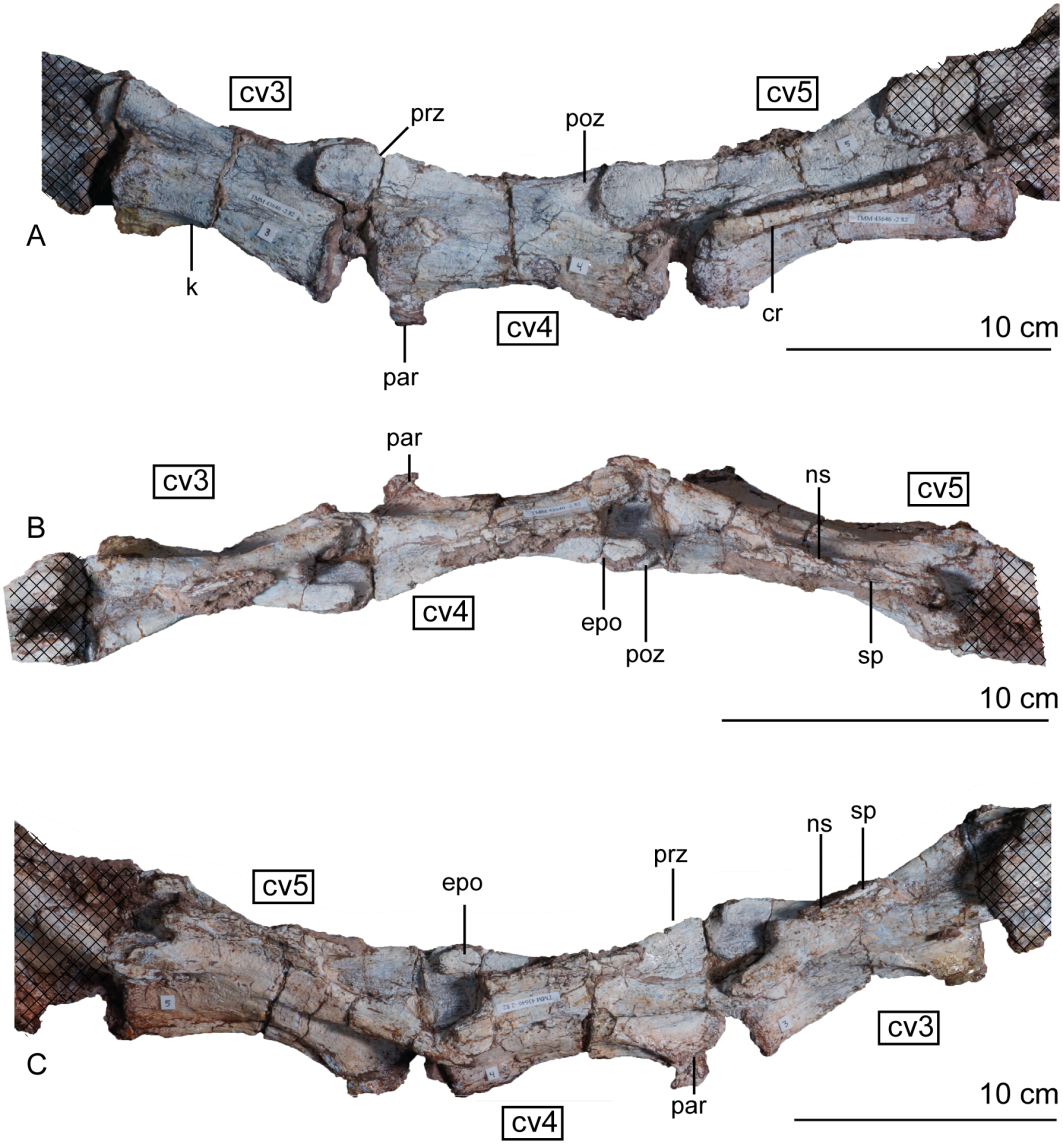
Articulated holotype cervical vertebrae 3, 4, and 5 of *Sarahsaurus aurifontanalis* in left lateral (A), dorsal (B), and right lateral (C) views. Stippled areas represent overlapping bone from preceding and/or succeeding elements. Abbreviations: cervical rib (cr), cervical vertebra (cv), epipophysis (epo), keel (k), neural spine (ns), parapophysis (par), postzygapophysis (poz), prezygapophysis (prz), spine table (sp).

The cervical vertebrae are largely unbroken but were crushed mediolaterally, with the right and left sides offset from one another in dorsal view. The prezygapophyses and postzygapophyses are tightly articulated (Fig 12). Every post-axial cervical vertebra is longer than the axis and every centrum is slightly amphicoelous, with the articular faces becoming more concave down the neck. The anterior face of the centrum is always dorsoventrally shorter than the posterior face. Aside from the crushing, the cervical vertebrae are compressed laterally with anteroposteriorly concave ventral margins that are outlined by a strong ventral keel. The concavity forming the ventral surface in altearl view is deepest in the anterior third of the vertebrae but becomes shallower moving posteriorly. The anterior cervical vertebrae are approximately 125% higher than they are wide, a feature found in the sauropods *Shunosaurus lii* [84, 85] and *Mamenchisaurus* [86–89]. This resemblance may be an artifact of the oblique post-mortem crushing in this region of the skeleton. Laterally, the anterior cervical vertebrae are subrectangular with an anterior margin that faces anteroventrally. This angle of the articular face disappears posteriorly down the neck. The third vertebra is 1.4 times longer than the axis, but not twice as long like as it is in *Adeopapposaurus mognai* [77]. The cervical centra lengthen from second to sixth; reaching a length of almost 100 mm. The longest cervical vertebra (vertebra six) is twice as anteroposteriorly long as it is dorsoventrally high. Centra seven through ten decrease in length, a trend maintained until the sixth dorsal vertebra. Similarly, the distance between the prezygapophyses and postzygapophses increases down the cervical series and is longest at the sixth vertebra.

**Fig 13:**
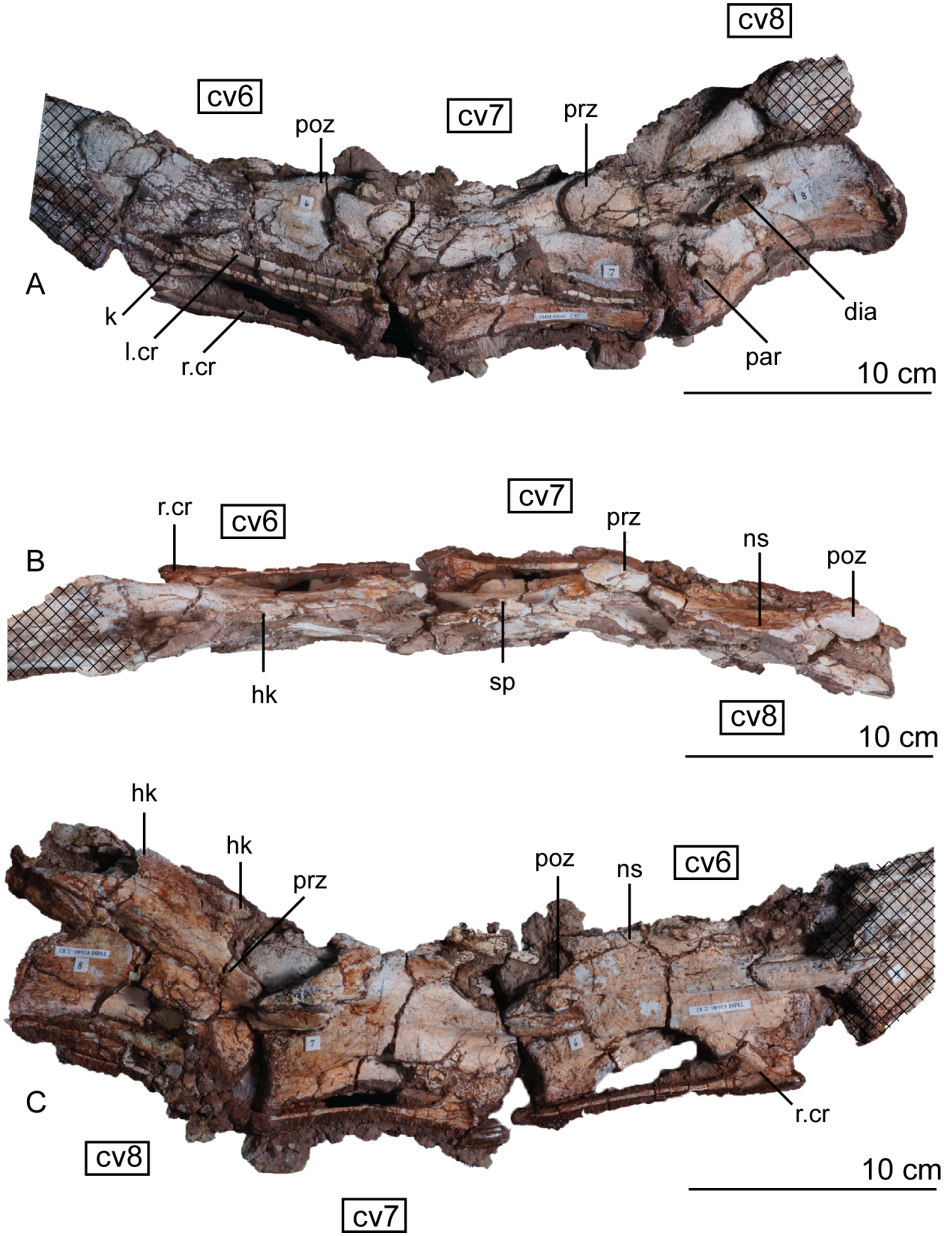
Articulated holotype cervical vertebrae 6, 7, and 8 of *Sarahsaurus aurifontanalis* in left lateral (A), dorsal (B), and right lateral (C) views. Hatched areas represent overlapping bone from preceding and/or succeeding elements. Abbreviations: cervical vertebra (cv), left and right cervical rib (l. cv, r. cv), diapophysis (dia), hook (hk), keel (k), neural spine (ns), parapophysis (par), postzygapophysis (poz), prezygapophysis (prz), spine table (sp).

**Fig 14:**
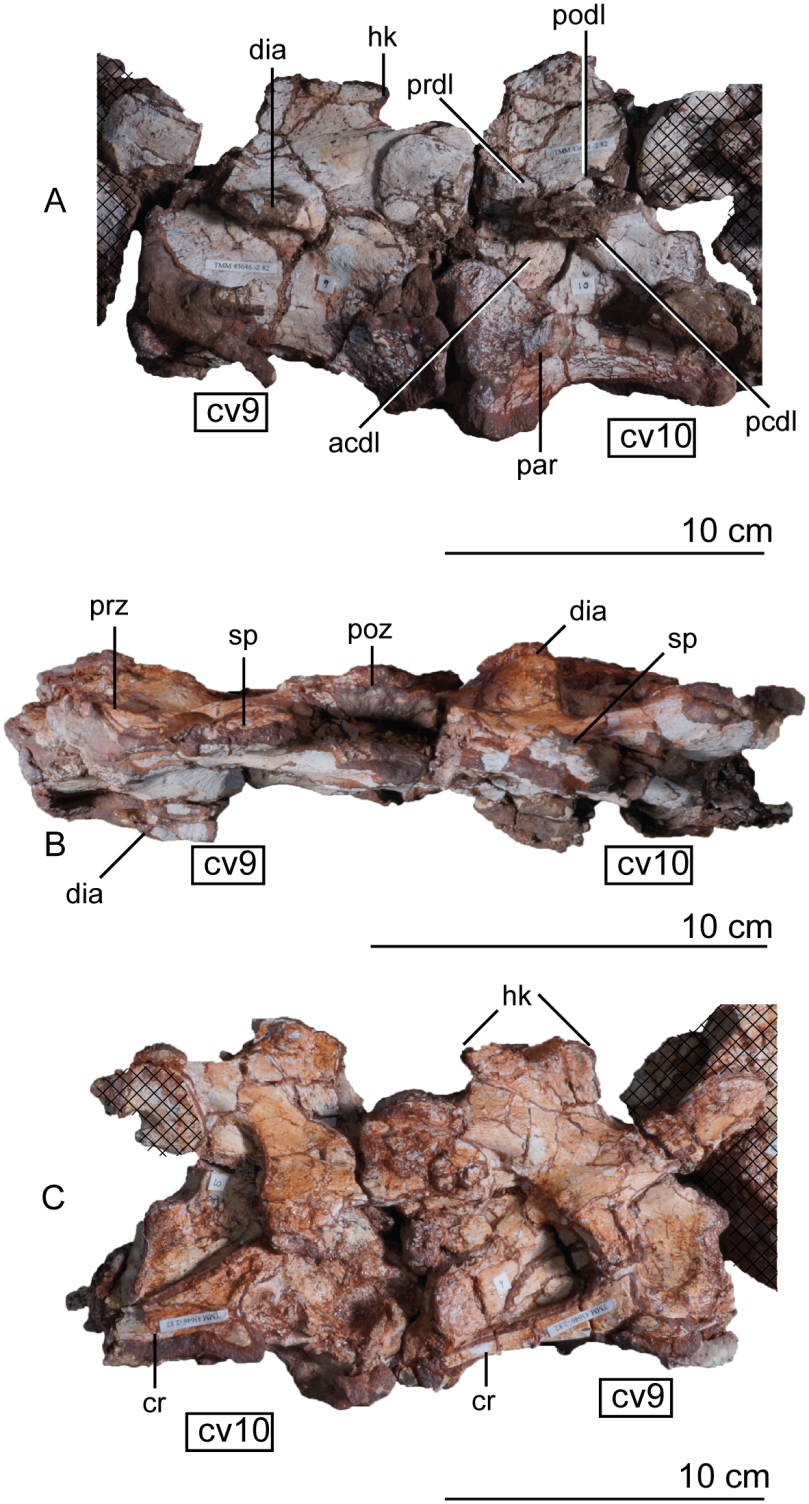
Articulated holotype cervical vertebrae 9 and 10 of *Sarahsaurus aurifontanalis* in left lateral (A), dorsal (B), and right lateral (C) views. Hatched areas represent overlapping bone from preceding and/or succeeding elements. Abbreviations: anterior centrodiapophyseal lamina (acdl), cervical rib (cr), cervical vertebra (cv), diapophysis (dia), hook (hk), parapophysis (par), posterior centrodiapophyseal lamina (pcdl), postzygadiapophyseal lamina (podl), postzygapophysis (poz), prezygadiapophyseal lamina (prdl), prezygapophysis (prz), spine table (sp).

In every cervical vertebra, the prezygapophyses overhang the centrum by a considerable margin, but the postzygapophyses remain flush with the rear face of the centrum (Figs 12A, 13A and 14A). The cervical prezygapophyses are spoon-shaped and broad, projecting anteriorly from the neural arch on laterally-directed stalks. In dorsal view, the prezygapophyses expand farther laterally than the postzygapophyses on the same vertebra. On each cervical vertebra, a table-like shelf connects the prezygapophyses and postzygapophyses to form the ventral margin of the neural arch. No laminae are found beneath this table in the anterior and mid-cervical vertebrae. The postzygapophyses are rounded and project posterolaterally on short stalks. All the epipophyses are low and are tightly joined to the postzygapophyses (Fig 12B), instead of the plesiomorphic condition of terminating in a free, pointed tip. *Lufengosaurus hueni* [78, 79], *Shunosaurus lii* [84, 85], and *Omeisaurus* [90, 91] share these low epipophyses with *Sarahsaurus aurifontanalis*.

The structure of the cervical neural spines of *Sarahsaurus aurifontanalis* is unique among early sauropodomorphs. The height of the neural spine of the third vertebra is shorter than that of the axis, but its anteroposterior length is subequal. The neural spines increase in height throughout the cervical series, but not as much as those of *Leonerasaurus taquetrensis* [30] and *Pulanesaura eocollum* [37, 38]. After the eighth cervical vertebra, the combined height of the neural arch and spine is more than the total height of the centrum, constituting at least half of the total height of the vertebra (Appendix C, S3 Text). This condition is similar to other early sauropodomorphs that have relatively low neural spines on the cervical vertebrae such as *Adeopapposaurus mognai* [77] and *Mussaurus patagonicus* [41]. The dorsoventrally longest neural spine occurs on the fifth vertebra. The convexity of the dorsal margin of the neural spine also reduces posteriorly. Beginning at vertebra four, short hooks are present on the anterior and posterior dorsal margins of the neural spines. In lateral view, these hooks form pinched corners create deep concavities on the anterior and posterior margins of the neural spine (Fig 14C). The fourth neural spine marks the beginning of a series of laterally-projecting table-like spurs in the extreme dorsal margin of the spine. On vertebra four, this is manifested as a lateral bulge one-third of the way down the spine. Posterior to that, each sequential neural spine has a more expanded table, so that by vertebra seven, the table is subrectangular in dorsal view and has small accessory lateral bumps. The spine table becomes thickens dorsally, as well. The hooks and laterally-expanded spine tables are most recognizable in the mid-cervical vertebrae (Fig 13B and 13C).

On every cervical vertebra, the parapophysis is represented by a subelliptical protuberance on the lateral surface of the centrum, located near the anteroventral margin. Posterior to the fourth vertebra, a clear distinction between the diapophyses and parapophyses can be made; where the parapophyses remain in the anteroventral margin of the centrum and the diapophyses shift posterodorsally up the side of the centrum (Figs 12A, 13A and 14A). When present, the diapophyseal facet is situated at the anterior portion of a short longitudinal ridge extending from the centrum body. In more posterior cervical vertebrae that ridge transitions into ventrolaterally-extending transverse processes. Starting at cervical eight, distinct transverse processes are formed by low, concave laminae extending from the prezygapophyses and postzygapophyses to the diapophyses. On the tenth vertebra, the transverse process is subtriangular and overhangs the prezygadiapophyseal, postzygadiapophyseal, anterior centrodiapophyseal, and posterior centrodiapophyseal laminae (Fig 14A) [92].

#### Cervical ribs

Many of the post-axial cervical ribs were articulated to the holotype cervical series. At least one rib is preserved in articulation with cervical vertebrae four through ten. All of the ribs are elongate, formed by a triradiate head and a long, tapering, subcylindrical shaft. The rib shafts are long and overlap two succeeding vertebrae (Fig 13A). Additionally, as preserved the cervical rib shafts are all positioned above the ventral margin of their respective centrum, and they overlap one another moving down the neck. The shape of the head of these ribs is transitional through the cervical series. The anterior process, capitulum, and tuberculum are weakly developed. In more posterior ribs, the rib head expands distally and the anterior process forms a hook-like projection that is larger than that of *Massospondylus carinatus* [14]. The dorsal margin of the anterior process is concave and transitions into the subcircular tuberculum. The ventral margin is straight but directed medially, forming the capitular process and a round capitulum. The two articular processes of the rib head are longer in the posterior cervical vertebrae, and they move apart from one another as the parapophysis and diapophysis separate along the vertebral column. A broadly concave web of bone connects the capitulum and tuberculum of the tenth cervical rib, and there is no trace of the anterior process.

#### Dorsal vertebrae

**Fig 15:**
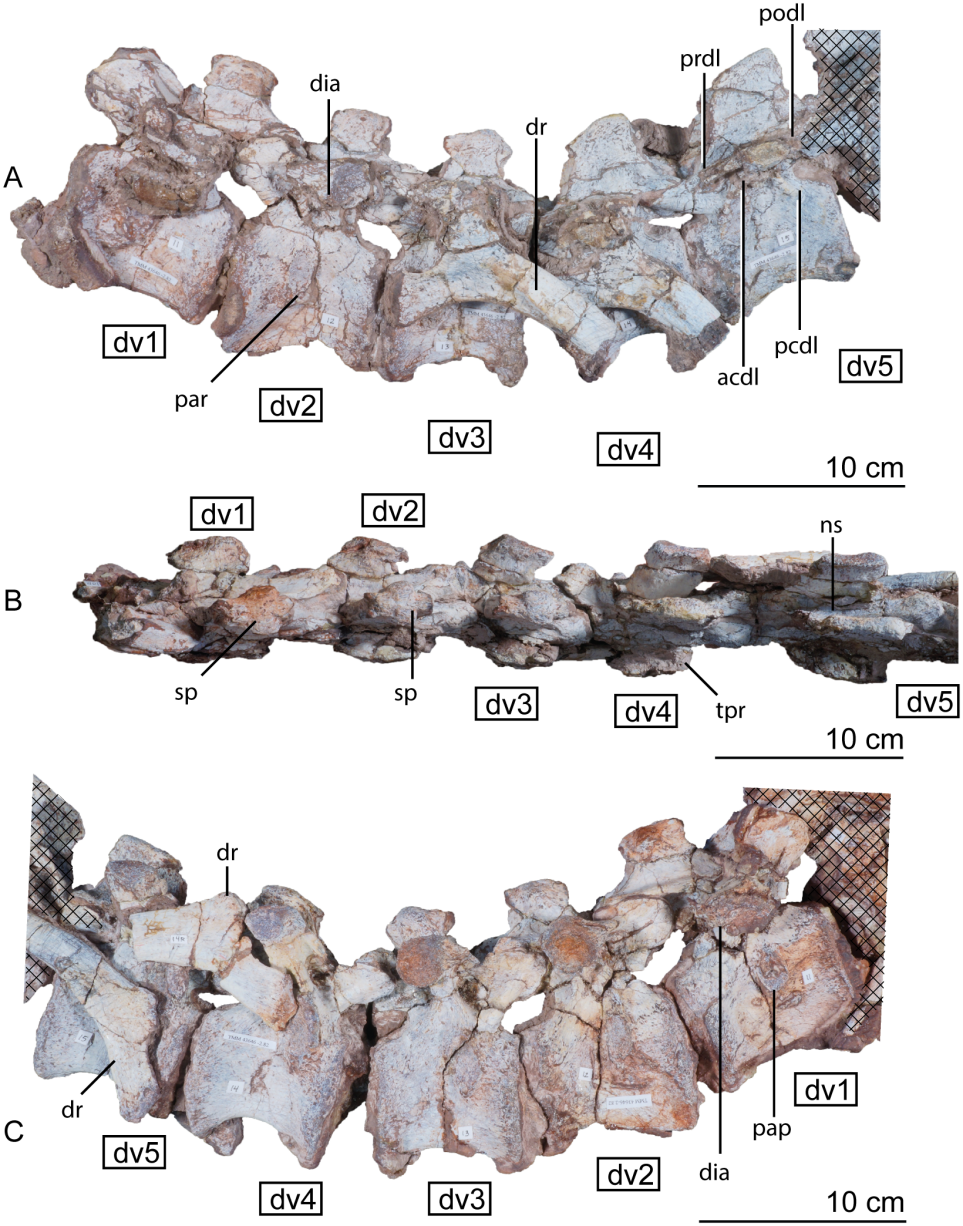
Articulated holotype dorsal vertebrae 1–5 of *Sarahsaurus aurifontanalis* in left lateral (A), dorsal (B), and right lateral (C) views. Stippled areas represent overlapping bone from preceding and/or succeeding elements. Abbreviations: anterior centrodiapophyseal lamina (acdl), diapophysis (dia), dorsal rib (dr), dorsal vertebra (dv), parapophysis (pap), neural spine (ns), posterior centrodiapophyseal lamina (pcdl), postzygadiapophyseal lamina (podl), prezygadiapophyseal lamina (prdl), spine table (sp), transverse process (tpr).

**Fig 16:**
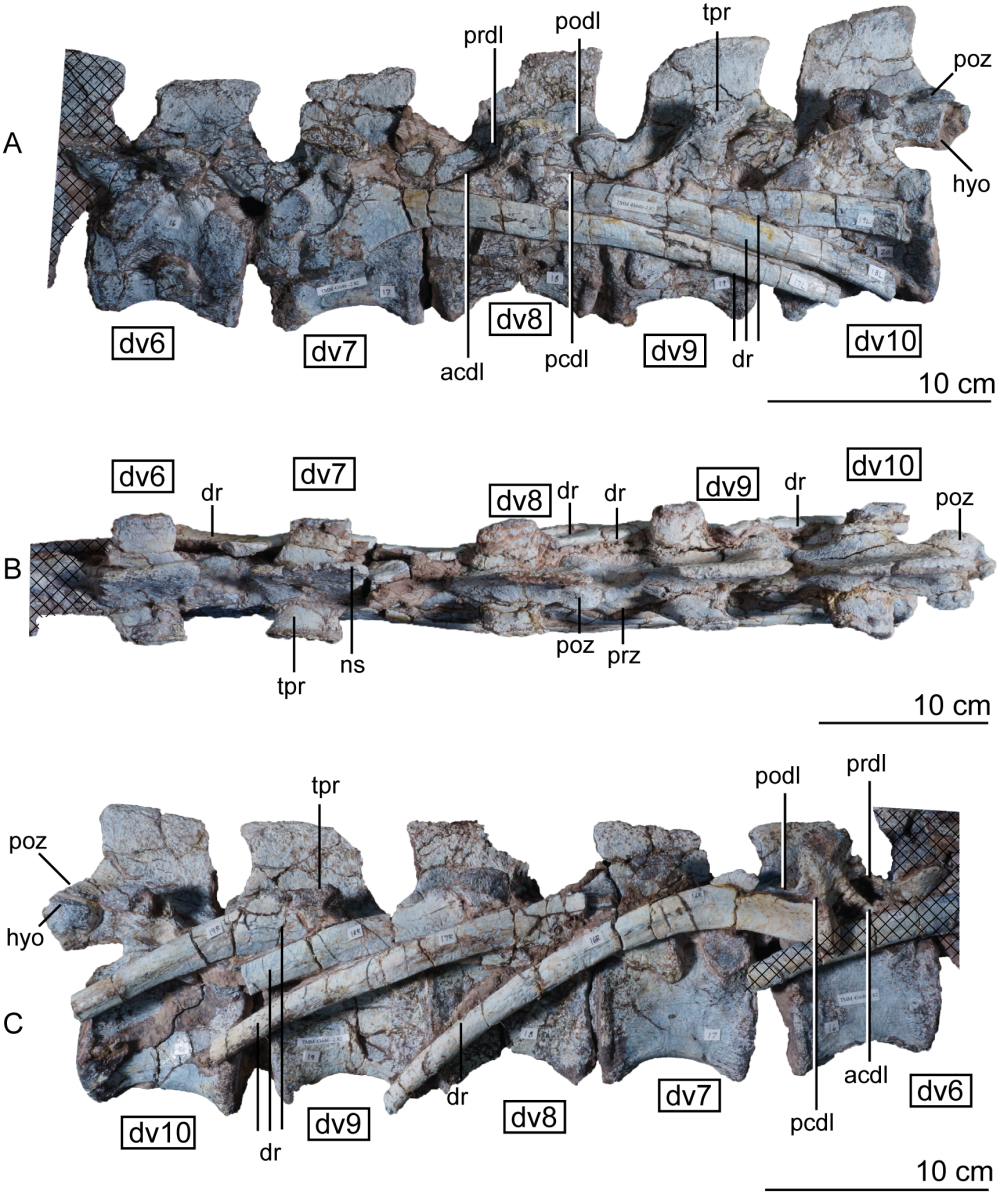
Articulated holotype dorsal vertebrae 6–10 of *Sarahsaurus aurifontanalis* in left lateral (A), dorsal (B), and right lateral (C) views. Stippled areas represent overlapping bone from preceding and/or succeeding elements. Abbreviations: anterior centrodiapophyseal lamina (acdl), dorsal rib (dr), dorsal vertebra (dv), hyposphene (hyo), neural spine (ns), posterior centrodiapophyseal lamina (pcdl), postzygadiapophyseal lamina (podl), postzygapophysis (poz), prezygadiapophyseal lamina (prdl), transverse process (tpr).

**Fig 17:**
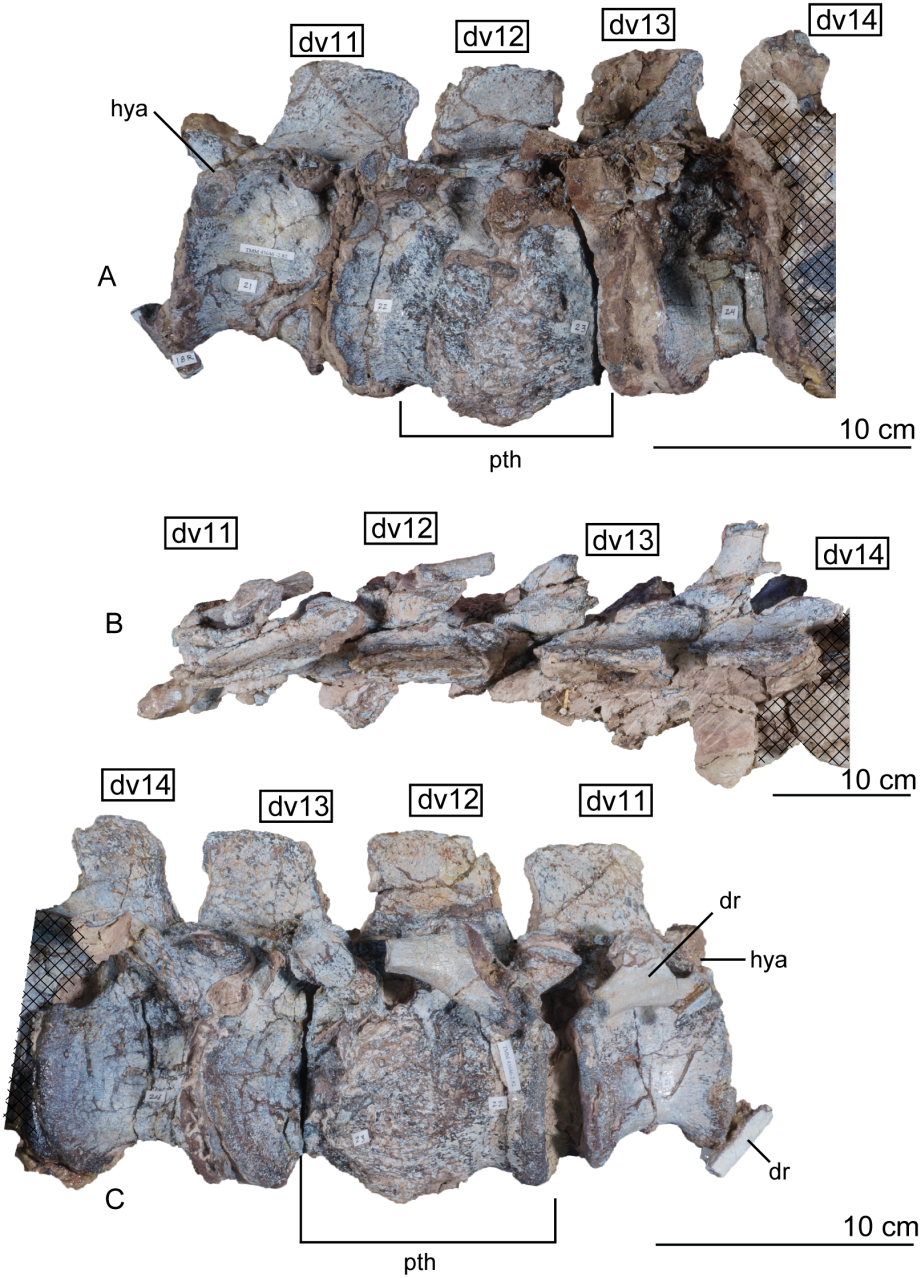
Articulated holotype dorsal vertebrae 11–14 of *Sarahsaurus aurifontanalis* in left lateral (A), dorsal (B), and right lateral (C) views. Not the pathological co-ossified centra of dorsals 12, 13, and 14. Stippled areas represent overlapping bone from preceding and/or succeeding elements. Abbreviations: dorsal rib (dr), dorsal vertebra (dv), hypantrum (hya).

The fourteen dorsal vertebrae are similar in structure to one another, differing mostly in the shape of the neural spines, the positions of the parapophyses and diapophyses, and in overall dimensions (Figs 15, 16 and 17). Again, these are tightly articulated with one another in the holotype specimen of *Sarahsaurus aurifontanalis*.

The anterior dorsal centra are slightly shorter than the posterior centra, and the inter-zygapophyseal distance and total vertebral height increase posteriorly. Overall, the centra are shorter and taller in the dorsal series than the cervical series, and the neural spines and arches contribute to much more of the total height of the vertebrae. The anterior and posterior articular surfaces of the dorsal centra are not inclined. The ventral margins of the centra are strongly concave and resemble a half-ellipse in lateral view. The first two centra of the dorsal series, vertebrae 11 and 12, also have a ventral keel, but the keel is much more reduced than in the cervical series.

The subelliptical prezygapophyses also assume a smaller, less rounded shape posterior to dorsal vertebra 4 (Fig 15A). Notably, it is at dorsal vertebra 4 that the prezygapophyses and postzygapophyses enlarge ventrally to form articular surfaces for secondary hyposphene-hypantrum articulations that persist at least as far as the last pre-sacral vertebra. Because the vertebral column is articulated, it is difficult to interpret details of their anatomy (dv10 in Fig 16). However, in comparing isolated neural arches of the paratype individual, the hyposphene-hypantrum articular surfaces resemble the condition found in the early saurischian *Herrerasaurus ischigualastensis* [93–96] as well as the early sauropodomorphs *Saturnalia tupiniquim* [97], *Massospondylus carinatus* [14], and *Adeopapposaurus mognai* [77], in that the hyposphenes are dorsoventrally shorter than the neural canal.

The elaborate laterally-projecting neural spine tables found along the cervical series persist up to the second dorsal vertebra (Fig 15B). This feature also is present in *Plateosaurus engelhardti* [98, 99], *Adeopapposaurus mognai* [77], *Massosponylus carinuatus* [14], and *Lufengosaurus hueni* [78, 79] but not in *Thecodontosaurus antiquus* [19, 100, 101] and *Saturnalia tupiniquim* [97]. The sauropodomorphs *Xingxiulong chengi* [36] and *Buriolestes schultzi* [71] possess laterally-projecting tables on the tops of the neural spines on the most posterior dorsal vertebrae. Such spine tables are lost in later-diverging sauropodomorphs like *Barapasaurus tagorei* [102], *Cetiosaurus oxoniensis* [103, 104], *Omeisaurus* [90, 91], *Mamenchisaurus* [86–89]. The anterior hook of the anterodorsal edge of the neural spine also disappears posterior to the second dorsal vertebra in *Sarahsaurus aurifontanalis*, but the posterior hook and the concave posterior margin of the neural spine is maintained on all through dorsal vertebra 12. The neural spines of dorsal vertebrae 5 to 11 are subrectangular and inclined posteriorly (Figs 15A and 16A). Throughout the dorsal series, the length of the neural spine gradually increases posteriorly until dorsal vertebra 12, which transitions into a shorter spine more closely resembling those of the sacral vertebrae from anterior to posterior trunk vertebrae along the remaining of the dorsal series (Fig 17A)(Appendix C, S2 Text). The dorsoventral height:anteroposterior length ratio of the 12th neural spine is 0.74 and that of the last dorsal neural spine is 1.3.

The parapophyses of the dorsal vertebrae shift from the anteroventral margin of the centrum to the ventral margin of the neural arch behind the prezygapophysis by dorsal vertebra 3. This condition is unlike some sauropodomorphs such as *Sefapanosaurus zastroensis* [40] and *Antetonitrus ingipes* [39], in which the parapophysis of the anterior dorsal vertebrae is found in the middle of the lateral side of the neural arch. Along this course, the parapophyses transition from subcircular to subelliptical in shape; they are only raised facets on the side of the centrum and do not project laterally. Posterior to vertebra 3, the parapophyses transition from the neural arch to the lateral extent of the transverse process. The diapophyses, however, mostly remain towards the center of the vertebrae just dorsal to the junction of the neural arch and the centrum (Figs 15B, 16A and 17A). The diapophyseal facets are subcircular in the anterior dorsals, but are modified into roughly triangular shapes by the laminae that join them. The transverse processes project outwards and slightly back but not upwards like in sauropods such as *Shunosaurus lii* [84, 85], *Cetiosaurus oxoniensis* [103, 104], and *Omeisaurus* [90, 91].

Four major diapophyseal laminae are found on dorsal vertebra 1 and demarcate three sharp triangular fossae around the diapophysis and transverse process (observed best in dv5 of Fig 15A). The associated fossae do not excavate extensively into the bone. The prezygadiapophyseal and postzygadiapophyseal laminae are present on the dorsal series, but become successively lower, moving posteriorly after the midpoint of the trunk. This feature is highly variable in early sauropodomorphs; it is difficult to determine where the prezygadiapophyseal lamina disappears in *Sarahsaurus aurifontanalis* because it is obscured by the articulated dorsal ribs. Spinodiapophyseal and suprapostzygapophyseal laminae are absent in *Sarahsaurus* but are found in some sauropods (e.g., *Barapasaurus tagorei* [102] and *Omeisaurus* [90, 91]).

No excavations that may represent pleurocoels are found on the lateral wall of any of the vertebrae of *Sarahsaurus aurifontanalis*. The centra of dorsal vertebrae 12 and 13 are pathological, being joined by a spondylarthropy, or overgrowth of rugose bone on both sides of the holotype individual (Fig 17A and 17B). This kind of pathology is not uncommon in fossil dinosaur bones, especially vertebrae [105–108]. This appears to have resulted from an injury sustained during life that the animal survived. The zygapophyseal articulation between the vertebrae does not appear to be aberrant, so the pathology is restricted to the two centra. In the last two pre-sacral vertebrae, the parapophyses shift posteriorly and the diapophyses move anteriorly enough that they share an articular facet for the ribs.

#### Dorsal ribs

The rib structure of *Sarahsaurus aurifontanalis* is unremarkable and similar to that of many other early sauropodomorphs. Many of the proximal portions of the dorsal ribs remain affixed to their respective vertebra in the holotype specimen (Figs 15A and 15B, 16A and 16B and 17B). The first dorsal rib is considerably longer than the last cervical rib. The last two-thirds of the rib shafts are fairly straight and subelliptical in cross-section. Proximally, the shaft bows outward before reaching the rib head. The capitular processes of the dorsal ribs are never long and terminate in a flat capitular surface. The tubercular processes become more prominant until the mid-dorsal vertebrae, rising from the rib head at an angle to accommodate the changing position of the diapophysis compared to the parapophysis. Just distal to the split between these two processes, the rib is subtriangular in cross-section. The dorsal surface is flattened somewhat as it bows outwards, and a shallow groove extends from the base of the tubercular process down the posterior side of the rib shaft. The capitulum and tuberculum approach one another in the posterior dorsal ribs until they merge at dorsal vertebrae 13 and 14 (Fig 17B). Depending on the skeletal maturity of the individual at the time of death, these last two ribs may have been fused to their vertebrae [106].

#### Sacral vertebrae

**Fig 18:**
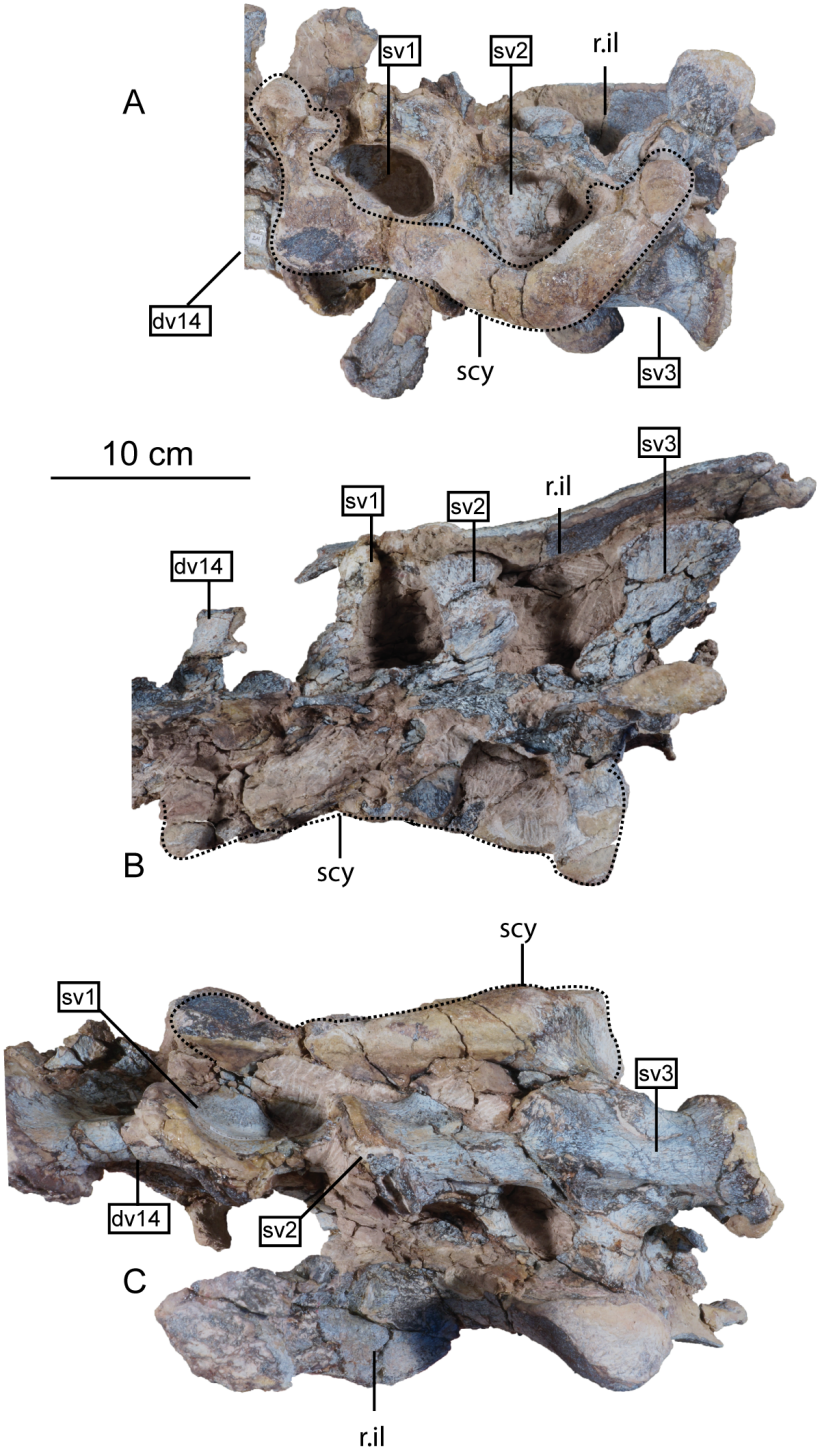
Articulated holotype sacrum of *Sarahsaurus aurifontanalis* with left ilium removed in left lateral (A), dorsal (B), and ventral (C) views. Abbreviations: accessory articulation (aa), dorsal vertebra (dv), right ilium (r. il), sacricostal yolk (scy), sacral vertebra (sv).

The sacrum is a solid block of three vertebrae and their fused transverse processes and ribs that articulate tightly with the medial walls of the paired ilia (Fig 18). The lateral profile of these fused transverse processes forms a longitudinal sacricostal yoke which articulates with the inside surface of each corresponding ilium. The holotype specimen of *Sarahsaurus aurifontanalis* preserves the sacrum in articulation, and the left ilium was removed to see the lateral surfaces of the sacral ribs. The sacral block is anteroposteriorly distorted post-mortem; the left side was pushed anteriorly and the right side moved posteriorly. The anterior two neural spines are broken but remain attached by matrix to the small block containing the left crus, tarsus, metatarsus, and a few caudal vertebrae. The neural spine of the third sacral vertebra is unbroken. The sacral elements of the paratype specimen include the isolated centra of the first and third sacral vertebrae, as well as two isolated sacral ribs, one from the first and the other dubiously assigned to the second vertebra.

As discussed by Nesbitt [75], numbering the sacral vertebrae has been conducted in one of two ways. Under one system, each vertebra can be numbered sequentially, starting with the first vertebra to attach laterally to the ilium via transverse processes or sacral ribs [74, 109]. Under the second system, the vertebrae can be numbered according to their relationship to hypothesized primordial sacral vertebrae [110, 111]. The plesiomorphic state for dinosaurs (and all archosauriforms) is the presence of two primordial sacral vertebrae, to which theropods, sauropodomorphs, and ornithischians add additional vertebrae homoplastically [111–113]. We follow Nesbitt’s [75] diagnosis of the two primordial sacral vertebrae based upon shape and orientation of the sacral ribs of these elements. To clarify terminology, we refer to the sacral vertebrae in series as sacral vertebrae one, two, and three, while acknowledging that primordial sacral vertebrae one and two are sacrals one and three, respectively.

The first vertebra in the sacrum of *Sarahsaurus aurifontanalis* is interpreted here as the first primordial sacral vertebra, not as a dorsosacral addition as originally described [25]. It lies behind the 24th pre-sacral vertebrae and their centra are not co-ossified. The first sacral rib projects dorsolaterally from the anterodorsal quarter of the centrum. Laterally, the rib is made up of two parts, a ventral subcircular articular surface and a smaller anterodorsal articular (Fig 18A). The main ventral articular surface of the rib is connected to this anterodorsal articulation by a thin sheet of bone that does not touch the ilium laterally. In lateral view, these features create a C-shape that is posteriorly concave. The first sacral rib is broad and subrectangular in anterior view excepting the dorsolateral concavity of bone that does not reach the ilium. The ventral margin of the anterior surface is also concave and slopes upward. The neural arch of the first sacral vertebra is obscured by matrix. Its neural spine is more rounded in lateral view than the subrectangular spine of dorsal vertebra 14, and thickens posteriorly. The ilium bears attachment scars on the anterior margin of the medial surface of the bone, just beneath the preacetabular process where the pubic peduncle begins its anteroventral descent. The anterodorsal margin of the inner surface of the blade also is roughened for the anterodorsal articulation of the first sacral rib.

The second vertebra in the sacral series is hypothesized to have been ‘inserted’ between the two primordial sacral vertebrae [75], although developmental experiments are needed to support the possibility of this occurring. Matrix covers the dorsal surface of the neural arch. The sacral rib of the second sacral vertebra is fused to the posterior margin of the first sacral rib and to the anterior margin of the third sacral rib. This forms a longitudinal sacricostal yoke that resembles an ‘L’ rotated 90° counterclockwise (Fig 18A) spanning all three sacral vertebrae. This co-ossification only occurs along the dorsal articular margin with the ilium; in ventral view, subelliptical spaces separate each sacral rib and their respective centra medially. The inserted second vertebra also articulates with the ilium at a T-shaped articular surface of the sacral rib that lies above the sacricostal yoke (Fig 18A). These articular surfaces are separated by a non-articulating gap like those of the first sacral rib. Because the ventral margin of the sacral verterbrae lies above the dorsal margin of the acetabulum, no portion of the sacral vertebrae contribute to the dorsal margin of the acetabulum. The exclusion of the sacral ribs from the acetabular margin is found in early sauropodomorphs but not in sauropods, in which the sacral ribs make a contribution to the dorsal margin of the acetabulum.

The second primordial sacral vertebra is interpreted to be the third vertebra in the sacrum and closely matches the anatomy described for the third sacral in *Massospondylus carinatus* [14, 75], *Adeopapposaurus mognai* [77], *Leonerosaurus taquetrensis* [30] and *Mussaurus patagonicus* [41]. The sacral rib projects posterolaterally as a broad, transversely-wide platform. The rib sits along the anterior half of the centrum, starting at the anteroventral margin and sloping posterodorsally. There is one long, subelliptical articular surface that tapers upwards but is quite robust along its entire length. Dorsally, the third sacral rib projects farther laterally than the first, and it is approximately twice as wide anteroposteriorly at its articulation with the ilium. In posterior view, the third sacral rib is strongly flared dorsoventrally extending laterally. A roughened strip of bone on the medial surface of the ilium also inclines posterodorsally and corresponds to the lateral expansion of the third sacral rib. This articular surface extends parallel and adjacent to the posterior margin of the dorsal third of the ilium, just ventral to the small brevis fossa. The prezygapophyses and postzygapophyses of this vertebra are relatively short, and accessory hyposphene-hypantrum articulations are absent. The last neural spine in the sacrum is slightly taller than the first and is more rounded dorsally in lateral view and thicker dorsally.

The three sacral centra are not completely co-ossified to one another. The lipped margins of the first and second sacral centra are clearly separate along the dorsal half of their articulation. However, those margins come together and may be co-ossified above this demarcation. The second and third sacral centra are completely co-ossified, but the bodies of the centra are distinguishable from one another (Fig 18C).

Based upon the two specimens of *Sarahsaurus aurifontanalis*, it seems that the sacrum fuses in stages. The first elements to fuse are the centra to the neural arches and the sacral ribs to the centra. Next, the sacral ribs fuse to one another forming the sacricostal yoke. The sacral centra fuse to one another, starting posteriorly. It is not clear when the sacral ribs begin to co-ossify with the ilium. The paratype specimen hardly exhibits any bone-on-bone rugose scars on the medial surface of the ilium, and the holotype ilium was only marginally co-ossified to the ribs and it was easily removed during preparation. In addition to the lack of co-ossification between the scapula and coracoid, this may suggest that the holotype specimen represents an individual that closely approached but did not reach terminal skeletal maturity at the time of death.

#### Caudal vertebrae

**Fig 19:**
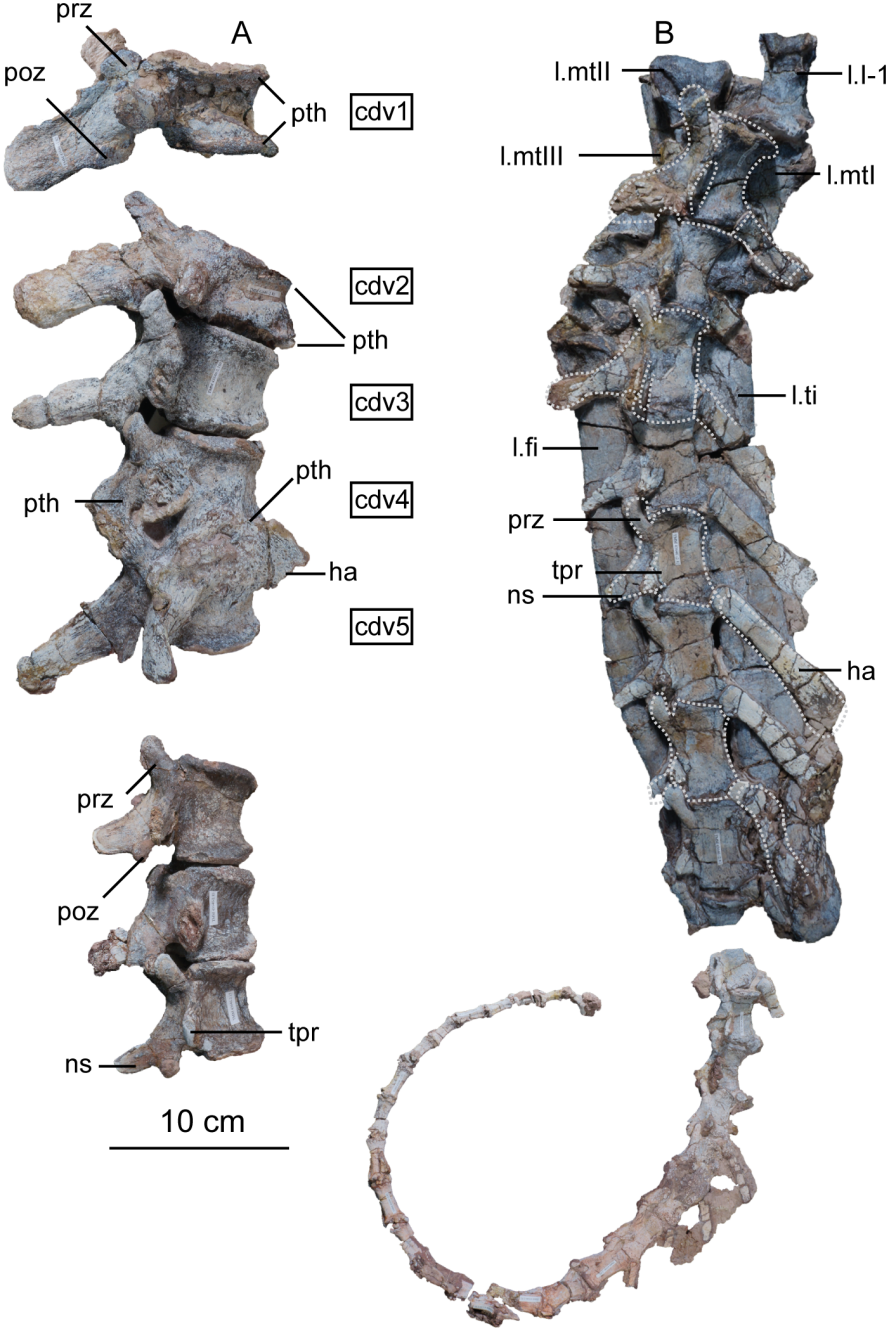
Holotype caudal vertebrae of *Sarahsaurus aurifontanalis* in right lateral view. A- caudal vertebrae 1–5 with three more distal vertebrae; B- 31 most distal caudal vertebrae. Note the pathological bone growth covering much of the proximal caudal centra. Abbreviations: caudal vertebra (cdv), left fibula (fi), haemal arch (ha), left metatarsal (mt), neural spine (ns), pathology (pth), postzygapophysis (poz), prezygapophysis (prz), left tibia (ti), transverse process (tpr).

We estimate that *Sarahsaurus aurifontanalis* had approximately 50 caudal vertebrae. The holotype specimen preserves 40 of these vertebrae; a few may be missing from the distal end of the tail, and two to four are missing from behind the fifth caudal vertebra. The proximal caudal vertebrae are distorted like the sacrum, being compressed mediolaterally post-mortem. Additionally, those centra exhibit pathological rims along their outer margins (Fig 19A). Caudal vertebrae 4 and 5 are co-ossified, especially on their left side (Fig 19A). The two centra are highly overgrown by bone, and bony tissue also spreads over the right prezygapophyseal and postzygapophyseal articulations and the haemal arch between the two vertebrae. This paleopathology is not unlike that which was described by Butler et al. [105] for articulated distal caudal vertebrae of *Massospondylus carinatus* [14]. The posterior two-thirds of the tail remain in articulation and have been mediolaterally flattened post-mortem in the middle caudal region. Little material from the tail was recovered from the paratype specimen, which only preserves a few caudal centra.

The anterior and posterior ventral corners of cauda centra contain facets that articulate with haemal arches. This results in a lateral outline that is sloped upwards at the anterior and posterior ventral margins of the centra. The anterior caudal centra are subelliptical in anterior outline, with the long axis of the ellipses oriented dorsoventrally. Mid-caudal centra are subcircular in anterior view, and the distal caudal vertebrae also are rounded but are slightly concave ventrally. A longitudinal sulcus, paralleled on each side by round ridges, is present on the ventral surface of every centrum of the caudal vertebrae except the proximal two. The presence of the sulcus is highly variable among early sauropodomorphs. It is present at least as posterior as the middle caudal vertebrae in *Anchisaurus polyzelus* [7,8, 32], *Adeopapposaurus mognai* [77], *Massospondylus carinatus* [14], and *Plateosaurus engelhardti* [98, 99], but it is not present in *Thecodontosaurus antiquus* [19, 100, 101] or *Riojasaurus incertus* [114, 115].

The transverse process of the caudal vertebrae are subtrapezoidal in cross-section towards the base of the process, and thin considerably into horizontal plate-like spines laterally. The base of the process lies solely on the neural arch and does not extend onto the centrum. The transverse processes of the most proximal tail vertebrae of *Sarahsaurus aurifontanalis* are inclined almost 30° dorsally. Those processes point ventrally in *Adeopapposaurus mognai* [77]. The lengths of the transverse processes diminish in the posterior vertebrae and their shape transitions from a flat process into a subtriangular, pointed knob. Along the same vertebrae, their inclination decreases until the small transverse processes of the third quarter of the tail point directly laterally. Transverse processes are absent on the last 20 vertebrae (Fig 19B). The most distal vertebrae are subrectangular in posterior view and have flattened lateral and ventral surfaces; these are similar in shape to those of *Anchisaurus polyzelus* [7,8, 32], *Aardonyx celestae* [33], and *Camelotia borealis* [116].

All of the neural spines on the caudal vertebrae are inclined posterodorsally. In anterior view, these are subrectangular in outline. The shape changes in more posterior caudal vertebrae into a subtriangular spine that decreases in relative height. Neural spines are absent on the distal quarter of the tail (Fig 19B). Both the prezygapophyses and postzygapophyses overhang the centrum for the entire caudal series. The prezygapophyses project anterolaterally along small stalks that originate behind the anterodorsal margin of the centrum. The postzygapophyses are situated on the posteroventral margin of the neural spines in the anterior- and mid-caudal vertebrae, but they migrate off the spine and form their own short posterodorsal projections originating on the distal half of the tail. In more proximal vertebrae the postzygapophyses are separated by a small notch that can be seen in dorsal view. The prezygapophyses and postzygapophyses of more distal vertebrae form short two-pronged processes in dorsal view. There are no hyposphene-hypantrum articulations along the caudal series.

#### Haemal arches

Isolated haemal arches (chevrons) were recovered from the type locality but cannot always be assigned with confidence to one or the other specimen of *Sarahsaurus aurifontanalis*. A few of these arches are preserved in articulation at the midpoint of the tail of the holotype individual. Regardless, enough of the elements are known that they can be described as closely resembling haemal arches of other early saurischians. They are Y-shaped in anterior view and individual haemal arches diminish in size posteriorly down the tail. Any given chevron is less than twice the length of the preceding chevron. The dorsal arms of the arches appear to meet, forming a half-circle articular surface along the entire series. That dorsal expansion is dorsoventrally convex but is divided proximally by a transverse ridge. The anterior articular surface is slightly anteroposteriorly wider than the posterior surface, and the articular surfaces fit snugly into the ventral notch between the caudal vertebrae.

Mediolaterally, the chevrons taper considerably distal to the junction of the proximal arms. This results in a long, flat strip of bone jutting posteroventrally which supported the hypaxial musculature of the tail (Fig 19B). The haemal canal is almost a perfect oval. Underneath the canal is a long median groove on the anterior and posterior surfaces of the bone. Rounded ridges frame this groove for its length down the haemal spine. In lateral view, the spine has subparallel anterior and posterior margins and is round at its termination. There is no marked anterodorsal expansion at the distal end of the haemal spines, and none of the chevrons contain a ventral slit. Small triangular chips of bone may represent the remains of haemal arches and are present as far down the tail as the 35th preserved caudal vertebra.

### Pectoral girdle

The left scapula and coracoid are preserved and undistorted in the holotype, as are the right scapula and coracoid of the paratype specimen. The scapulocoracoid is not co-ossified in either individual. The co-ossification of these elements may occur late in ontogeny, but their sutures may never be entirely erased [106]. That lack of fusion can be seen in most early sauropodomorph taxa, such as *Saturnalia* (MCP 3844-PV), *Plateosaurus engelhardti* (SMNS 13200 and BSP 1962 I 153), and *Jingshanosaurus xinwaensis* (LV003) [117]. The scapula and coracoid are described as if they are articulated and oriented such that the long axis is vertical. These elements would be slanted backwards in life such that the glenoid fossa faced posteroventrally. Only the left sternal plate was recovered from the holotype specimen of *Sarahsaurus aurifontanalis*, and it was found in association with the distal tips of the anterior dorsal ribs, along with the left clavicle. Left clavicles were found in both specimens of *Sarahsaurus aurifontanalis*. The paratype clavicle is incomplete on both ends and is smaller than that of the holotype. The holotype clavicle is complete.

#### Scapula

**Fig 20:**
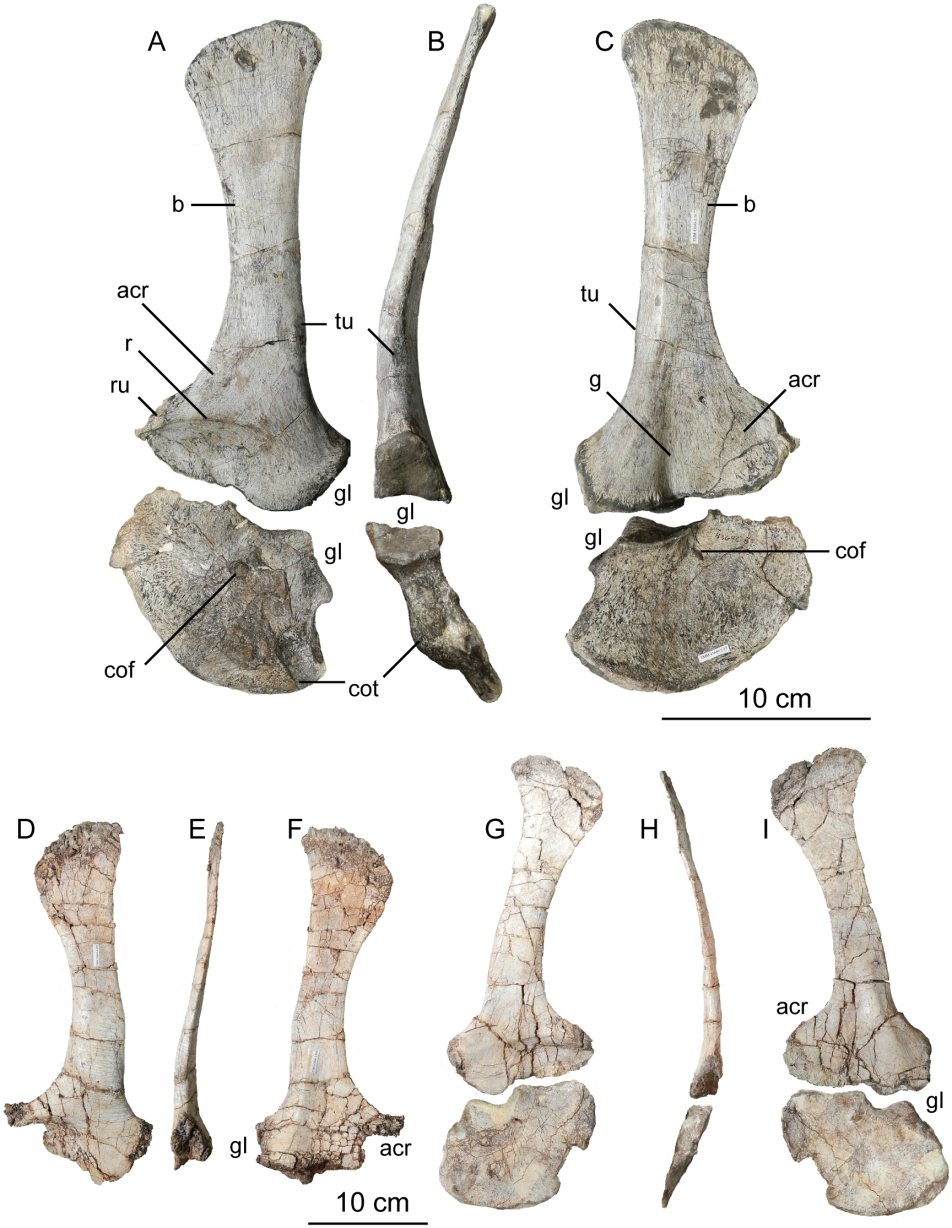
Holotype left scapula and coracoid (A-C), paratype left scapula (D-F), and paratype right scapula and coracoid (G-I) of *Sarahsaurus aurifontanalis*. A, D, G- lateral view; B, E, H- anterior view; C, F, I- medial view. Abbreviations: acromion (acr), blade (b), coracoid foramen (cof), coracoid tubercle (cot), depression (d), groove (g), glenoid (gl), ridge (r), rugosity (ru), tuberosity (tu).

**Fig 21:**
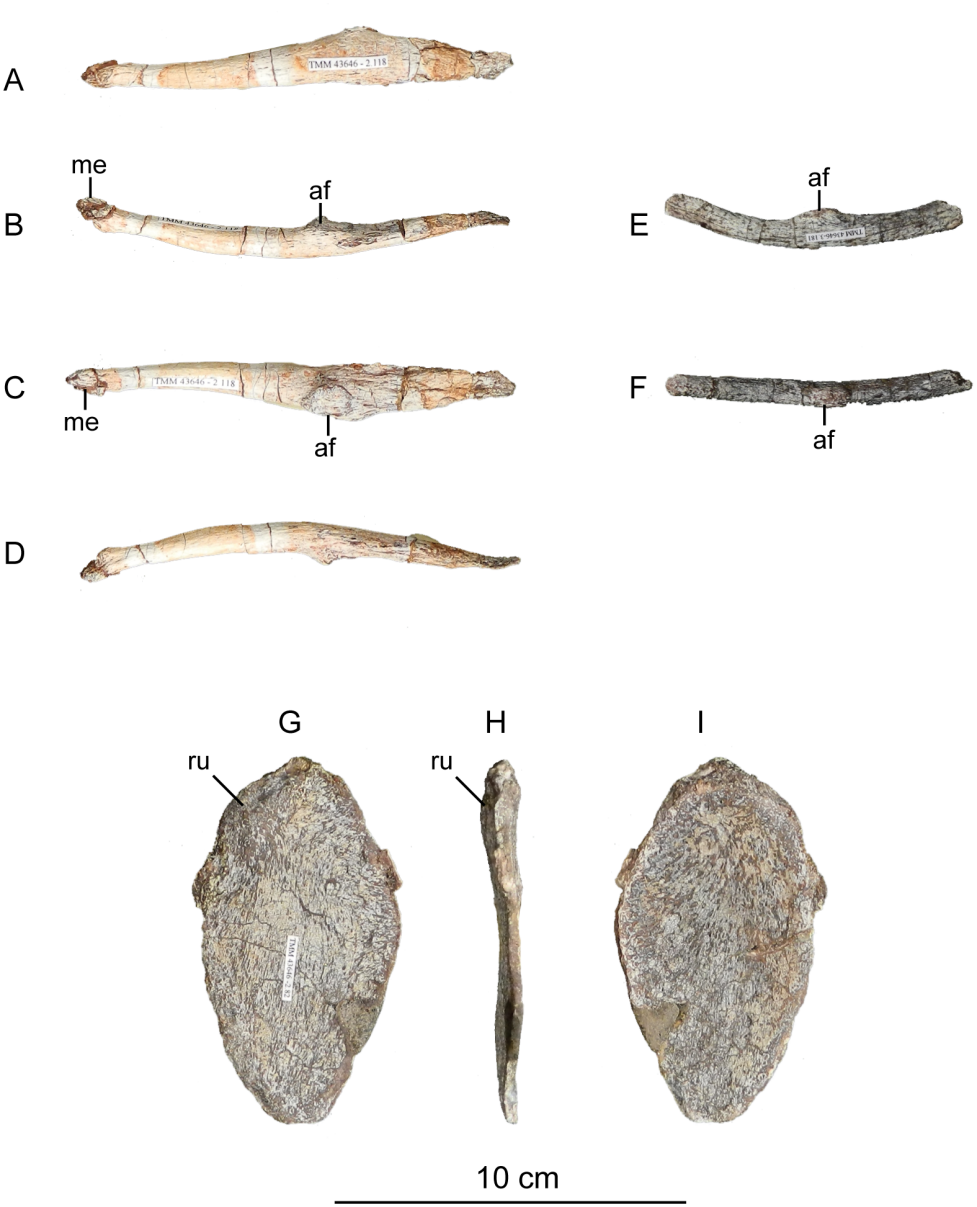
Holotype left clavicle (A-D), paratype left clavicle (E-F), and holotype left sternal plate (G-I) of *Sarahsaurus aurifontanalis*. A, G- external view; B, E- dorsal view; C, F, I- internal view; D- ventral view; H- lateral view. Abbreviations: articular facet (af), midline expansion (me), prongs (pr), rugosity (ru).

The scapula is shaped like an hourglass that arches laterally, conforming to the shape of the ribcage (Fig 20). The dorsal and ventral expansions of the scapula are separated by a narrowed strap-like shaft that is subelliptical in cross-section. The ventral expansion, comprising the acromion process anteriorly and the glenoid region posteriorly, is broader anteroposteriorly than the dorsal expansion. This feature is plesiomorphic for sauropodomorphs. The posterodorsal corner of the blade extends further dorsally relative to the anterodorsal corner. There is an oval depression on the dorsolateral surface of the left holotype scapula that may represent a tooth mark of a scavenging theropod such as *Dilophosaurus*, but it is too large to have been produced by the contemporaneous coelophysoid ‘*Syntarsus*’ *kayentakatae*. The dorsal rim of the scapula is formed by a squared edge that is mediolaterally thicker posteriorly than anteriorly. The shaft is constricted and forms a blade, the smallest width of which is found halfway down the bone. The greatest dorsoventral length of the scapula is seven times the thinniest anterioposterior width of the blade. The posterior margin is long and slightly concave, extending from the posterodorsal edge to the posteroventral glenoid facet. Ventrally, the shaft meets the glenoid region at a steeper angle than it meets the posterodorsal corner of the blade. Just below mid-shaft, there is a pronounced tuberosity on the posterolateral surface of the scapula (Fig 20A–20C). That feature is more prominent in the holotype specimen, and only exists as a small, low convexity in the paratype specimen. Additionally, a small, round depression lies just dorsal to the glenoid on the posterolateral surface of the scapula (Fig 21A and 21B). The anterior margin is much more concave and is shorter than the posterior margin, terminating ventrally at the anteroventral margin of the acromion, which is a thin margin of bone.

The ventral margin of the acromion portion of the scapula makes a 60° angle with the long axis of the scapular blade like that seen in other early sauropodomorphs (e.g., *Plateosaurus engelhardti* [98, 99], *Seitaad ruessi* [22], and *Thecodontosaurus antiquus* [19, 100, 101). A ridge extends along the lateral surface of the scapula where the blade expands anteroposteriorly to delimit the acromion region from the glenoid region. That ridge traverses a circular path circumscribing a pre-glenoid fossa that is deepest anteriorly. Medially, a longitudinal ridge extends from the posteroventral edge of the glenoid facet to the pinched midshaft of the blade. Anterior to the ridge is a triangular depression that makes up most of the acromion region of the proximal portion of the scapula (Fig 20C). A small foramen exits the medial surface of the scapula in this depression, where the scapular blade meets the acromion.

The glenoid region is the mediolaterally thickest portion of the ventral region of the scapula. The scapular contribution of the glenoid fossa is subtrapezoidal in posterior outline, is taller than it is wide, and has a dorsomedial margin that is higher than the dorsolateral margin. The ventral margin of the glenoid facet is concave. Viewed ventrally, the base of the scapula is curved. Small striations extend perpendicular to the ventral articular surface on both the lateral and medial margins. The glenoid region is clearly divided into the glenoid facet of the scapula and the larger articular surface for the coracoid, which is pitted and rugose. In ventral view, the acromion tapers and articulates with a corresponding thin margin of the coracoid. The segments of the articular surface for the coracoid are convex in lateral and medial views and are separated by a small concave slot that articulates with a knob on the dorsal margin of the coracoid. This knob and slot is not as apparent in the paratype specimen (Fig 20G and 20I). This may either represent differential growth between the individuals or may represent the beginning of the suturing of these two elements as the individual approached skeletal maturity.

#### Coracoid

The coracoid is subelliptical in lateral view. The coracoid is more concave medially than it is convex laterally owing to the thicker posterior glenoid portion of the coracoid. The dorsal margin articulating with the scapula is separated into two concave portions delimited by the dorsal knob (see the description of the scapula). In dorsal outline, the curvature of the bone is evident, and the thicker articular surface is rugose. The coracoid portion of the glenoid is immediately adjacent to this surface posteriorly and is separated from it by a ridge. The glenoid facet of the coracoid is suboval in posterior outline, wherein the slightly convex dorsal margin articulated into the slightly concave ventral margin of the scapular facet of the glenoid. A small, flat, subrectangular area lies immediately ventral to the glenoid in posterior view, which forms to straight margins between the glenoid and coracoid tubercle in lateral and medial view. This rectangular area is slightly depressed in the holotype, but is flat in the paratype. Posteriorly, the medial edge of the squared surface is more pronounced and juts farther posteriorly, allowing the surface to be viewed obliquely in lateral view. Posteroventral to the squared area on the lateral surface is the coracoid (= biceps) tubercle, which is quite prominent in the holotype specimen, but seems to be incomplete in the paratype (Fig 20B). The presence of a coracoid tubercle is plesiomorphic for Dinosauria and is found in the non-dinosaur dinosauriforms *Silesaurus opolensis* [118] and *Marasuchus lilloensis* [119], but the tubercle is lost in sauropods. The coracoid tubercle is subtriangular in posterior view and is separated into a larger lateral and smaller medial surface by a low ridge terminating in a point that merges with the prominent posteroventral margin of the coracoid. Laterally, the coracoid tubercle is subelliptical and is inclined, extending subparallel to the glenoid facet of the coracoid.

The posterior margin of the coracoid begins ventral to the coracoid tubercle and proceeds from the prominent, thick posteroventral edge of the bone to the anterior tip of the thin articulation with the scapula. The posterior two-thirds of the posterior margin is thick and forms a hard, squared edge. Anteriorly, the margin thins into sheet-like bone. The large, round coracoid foramen enters the lateral surface of the coracoid almost halfway between the anterodorsal margin and posteroventral margin of the bone (Fig 20A and 20C). The coracoid foramen passes obliquely through the bone, traversing a posteroventral path. Medially, the coracoid foramen is not nearly as round, but it may have been crushed post-mortem. The medial aperture of the canal also does not lie as close to the middle of the dorsal surface of the coracoid as it does laterally, and instead is more anterior and is closer to the articular surface of the scapula. Similar striations to those found along the articular surface of the scapula punctuate the corresponding lateral and medial dorsal margins of the coracoid.

#### Clavicle

This element is elongate and separated into a longer medial arm and lateral shorter arm by an oval, flat articular facet on the internal or dorsal surface (Fig 21A–21D). This bone is thickest around the region of this facet. The medial arm is more dorsoventrally compressed than the lateral arm, which is subcylindrical in cross-section. Both ends taper distally and the entire element is arched ventrally. The lateral end of the clavicle is rugose surrounding a flattened internal surface. The medial arm of the clavicle taper medially and end in an expanded region that probably articulates with the clavicle from the opposite side. Posteroventrally, the medial expanded tip forms two small prongs (Fig 21D). The most dorsal of those prongs is slightly larger and projects farther medially.

#### Sternal plate

The sternal plate is subrhombohedral in ventral outline, and the posterior half is more elongate and pointed than the anterior half (Fig 21G and 21I). The overall shape of the sternal plate in *Sarahsaurus aurifontanalis* is very similar to that of *Yunnanosaurus huangi* [120], but not those of *Lufengosaurus hueni* [78, 79] or *Massospondylus carinatus* [14], whose sternal plate is more squared in outline. The internal surface of the sternal plate of *Sarahsaurus* is flat and featureless. The external surface is only weakly convex and does not have a longitudinal ridge extending down its length like that found in the sauropods *Cetiosaurus oxoniensis* [103, 104], *Shunosaurus lii* [84, 85], and *Mamenchisaurus* [86–89]. The anterior apex of the sternal plate is rugose and thickened near its association with the coracoid. The medial margin of the sternal plate also is thickened slightly, presumably where the paired sternal plates meet at the midline. Most of the bone is thin, especially approaching the lateral margin.

### Reconstruction of the pectoral girdle

There is some disagreement regarding the orientation and articulation of elements within the pectoral girdle in early sauropodomorph dinosaurs. Most of that confusion is owing to the absence of specimens that preserve articulated scapulocoracoids, sternal plates, and clavicles. Often, those elements are known from later-diverging taxa within major dinosaurian groups, but early dinosaur skeletons historically have not contained those elements.

Ossified sternal elements are plesiomorphic for dinosaurs [75], and sternal plates are known in ornithischians (stegosaurs, ceratopsians, and ornithopods) and saurischians, including many early sauropodomorphs and sauropods [121]. Clavicles are rarer, in part because they are small, thin, and superficially resemble gastralia (Fig 21A–21F). Clavicles are present in non-archosaurian archosauriforms and early crocodylian-line archosaurs, lost in multiple lineages, incorporated into a heavily ossified sternum, or even fused with one another within Archosauria [75] Among dinosaurs, clavicles are known primarily from saurischians, but they are reported in the ornithischian *Psittacosaurus mongoliensis* [122, 123]. The presence of a furcula formed by the co-ossification of paired clavicles at the midline may be plesiomorphic for theropods [124].

The interclavicle is plesiomorphically present in archosauriforms [125] but this element is lost in dinosaurs. Because fossils of early dinosauriforms like *Silesaurus opolensis* [118] and *Marasuchus lilloensis* [119] do not preserve this region of the pectoral girdle, it cannot be determined if the absence of the interclavicle in dinosaurs is plesiomorphic [75]. An interclavicle was reported from *Massospondylus carinatus* [14], but was later re-identified as a clavicle [125]. More recently, putative interclavicles were reported from a sauropod quarry in the Morrison Formation, but these elements were not found in articulation and could just as easily represent sternal ribs or gastralia that were also found in great abundance in that locality [126].

**Fig 22:**
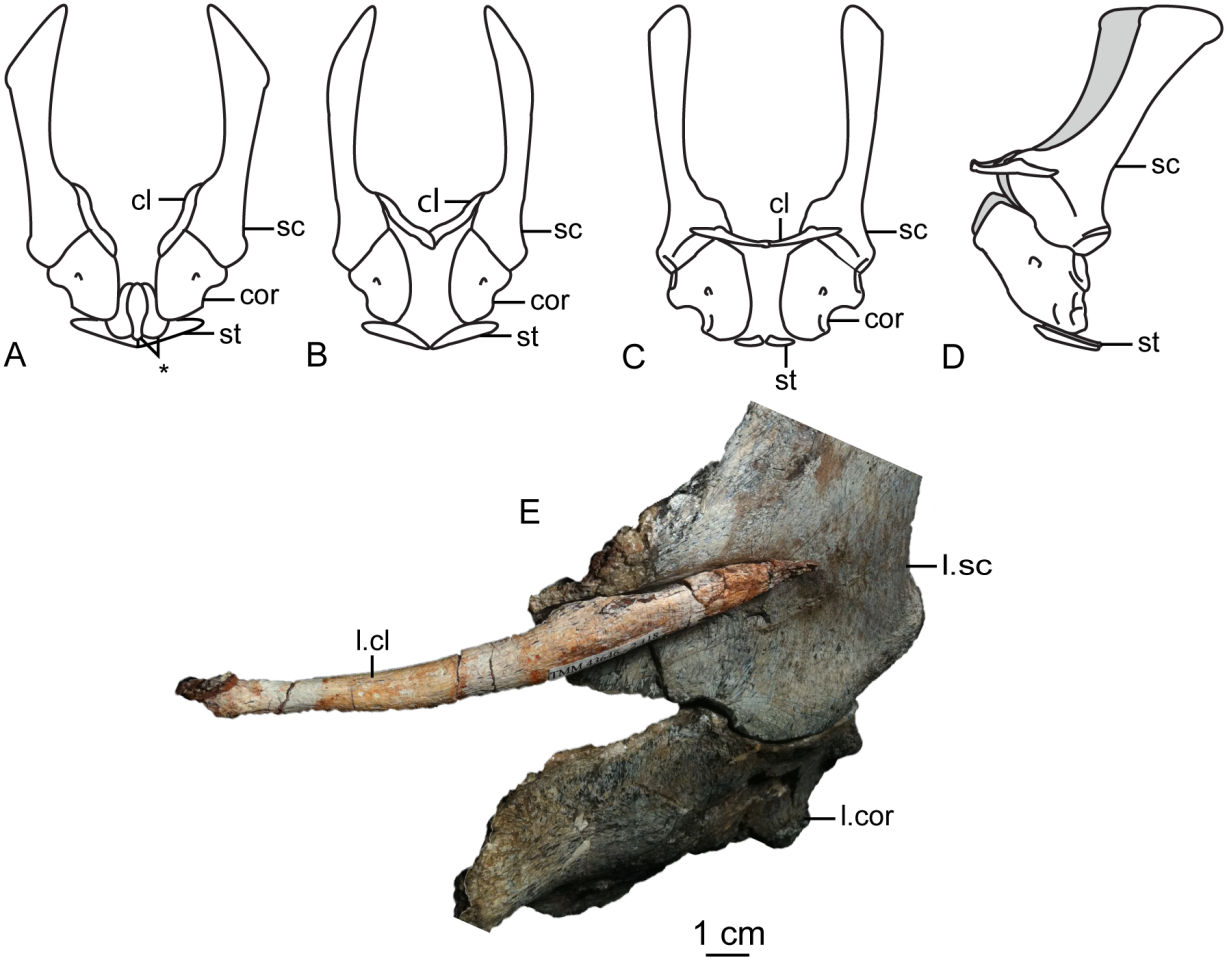
Past and recent reconstructions of the pectoral girdle of sauropodomorph dinosaurs. A- Non-bracing model of *Plateosaurus engelhardti* in anterior view. Asterisk denotes episternal bones for which no evidence is known; B- Bracing model of *Massospondylus carinatus* (BP/1/5241) in anterior view; C- Proposed bracing model of holotype specimen of *Sarahsaurus aurifontanalis* (TMM 43646–2) in anterior view; D; Proposed bracing model of *Sarahsaurus* in left lateral view; E- Color photograph of articulated holotype left clavicle, scapula, and coracoid of *Sarahsaurus aurifontanalis*. Modified from [127] and [128]. A-D not to scale. Abbreviations: clavicle (cl), coracoid (cor), scapula (sc), sternal plate (st).

Some well-preserved specimens of *Plateosaurus engelhardti* [98, 99] and *Massospondylus carinatus* [14] include clavicles closely associated with the pectoral girdle. Previous reconstructions place the clavicles on the anterodorsal margin of the acromion process of the scapulae in either a bracing or non-bracing model (Fig 22). In the non-bracing model, the clavicles are positioned parallel to the main body of the scapula and do not meet at the midline, contacting instead the coracoid only (Fig 22A) [127]. However, later authors re-evaluated well-preserved and articulated specimens of *Plateosaurus engelhardti* (SMNS 58958) and *Massospondylus carinatus* (BP/1/5241), and concluded that the bracing model of clavicular articulation was more plausible [128]. In that model, the proximal tips of the clavicles contact the acromion process of the corresponding scapula, but then both clavicles proceed towards the midline where their spatulate ends overlap medially (Fig 22B). The overlapped, V-shaped arrangement (also found in theropods with furculae) braced the pectoral girdle and kept it functionally immobile [128].

The pectoral girdle of *Sarahsaurus aurifontanalis* is well-preserved. Although the holotype elements were not articulated, the entire left side of the girdle was found in association. Obviously, the scapula and coracoid articulate along a tight, immobile joint at the glenoid region. The subelliptical facet on the clavicle corresponds to a smooth, thickened articular surface on the acromion, whereas the rest of the flattened, internal lateral surface of the clavicle extends transversely across the scapula just above the subcircular ridge near the acromion. This arrangement differs from previous reconstructions in placing more of the lateral half of the clavicle along the acromion of the scapula (Fig 22E).

In this case, the fact that these elements were disarticulated was fortunate because this seemingly seamless articulation cannot be discounted as an artifact of the clavicle being crushed against the scapula. The expanded medial tips of the clavicles probably overlap one another as shown in Fig 22B and 22C [128]. As exhibited by mounted and digitally reconstructed skeletons of *Plateosaurus engelhardti* [98, 99], the scapulae and coracoids were not held subparallel to the trunk vertebrae, but instead wrapped around the rib cage and are angled between 45° and 65° with respect to the horizontal [129, 130]. The orientation does not push the pectoral girdle too far forward and also agrees with the plesiomorphic bauplan of archosaurs, as exemplified by *Euparkeria capensis* [131–132], in which the coracoids almost touch at the midline restricting the position of the sternal plates to immediately posterior to the coracoids [133, 134]. The thickened anterior apex of the sternal plate of *Sarahsaurus aurifontanalis* most likely touches or is closely associated with the coracoid, and the linear medial margins of each sternal plate meets one another along the midline (Fig 22).

### Forelimb

Both humeri are preserved in the holotype of *Sarahsaurus aurifontanalis*, although owing to post-mortem alteration, various structures are compressed or elongated depending on their orientation in the skeleton. The right humerus was not crushed, but the proximal end of the bone, including the head, appears to be pathologically thickened by secondary bone growth. The left humerus is elongated and compressed anteroposteriorly at both ends, and the deltopectoral crest is flattened closer to the humeral shaft. The left radius and ulna were found associated with the distal end of the left humerus, and are part of a complete and articulated left antebrachium and manus. The left radius was rotated over the ulna post-mortem, and no longer represents the in-vivo arrangement of the forearm elements between the elbow and the wrist. Only the holotype specimen preserves an ulna. Three radii were found; the right from the paratype and the right and left pair from the holotype. The humerus, radius, and ulna are described with their long axes oriented vertically. Carpals, metacarpals, and phalanges are oriented such that the palmar surface is ventral.

#### Humerus

**Fig 23:**
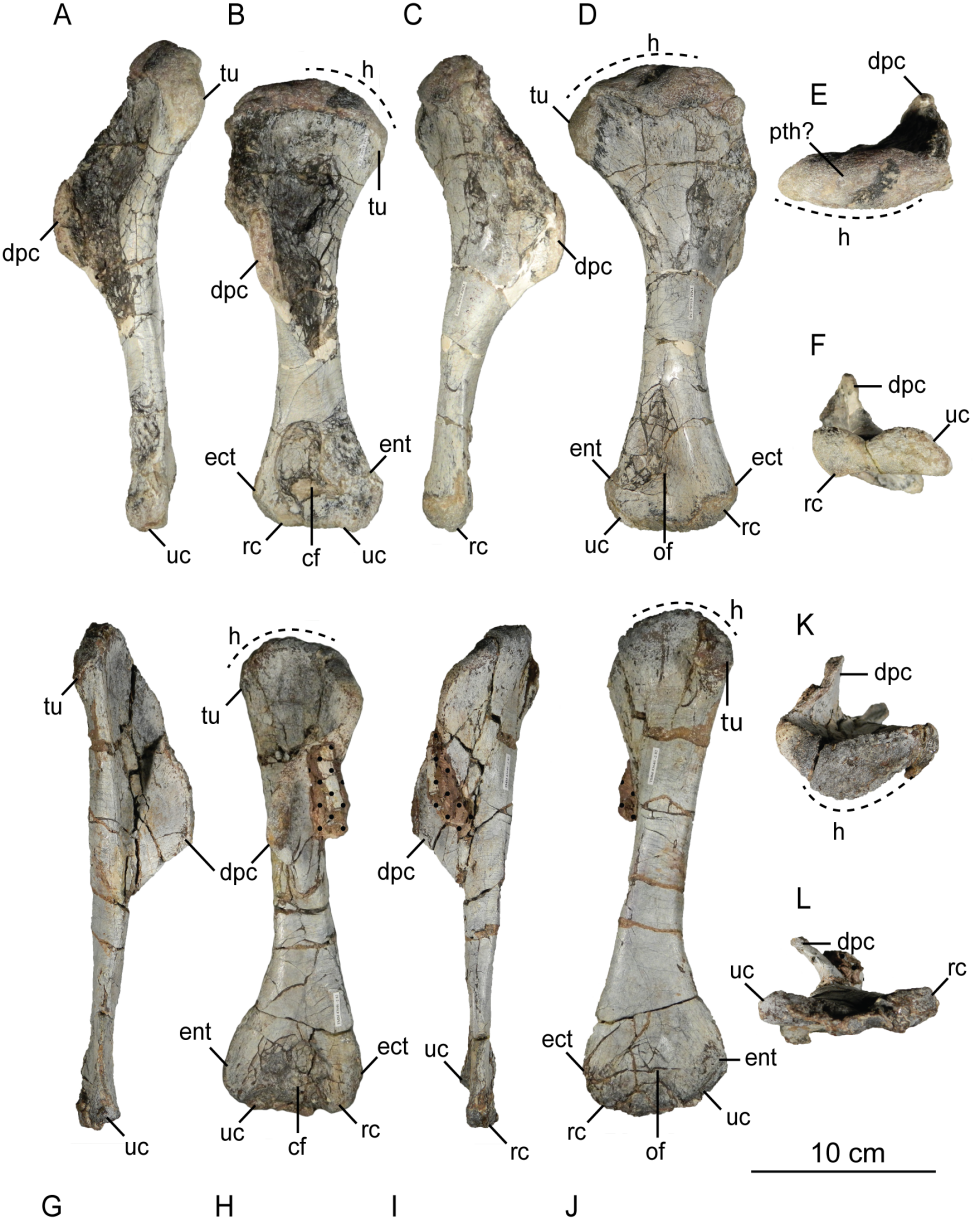
Holotype right humerus (A-F) and left humerus (G-L) of *Sarahsaurus aurifontanalis*. A, G- medial view; B, H- anterior view; C, I- lateral view; D, J- posterior view; E, K- proximal view; F, L- distal view. Stippled areas indicate matrix and bone not belonging to the humerus. Abbreviations: cuboid fossa (cf), deltopectoral crest (dpc), ectepicondyle (ect), entepicondyle (ent), head (h), olecranon fossa (of), pathology (pth), radial condyle (rc), tuberosity (tu), ulnar condyle (uc).

The length of the humerus is 61% of the length of the femur. The proximal outline of the humerus resembles an asymmetrical chevron with its apex pointing posteriorly, and the lateral limb of the chevron is longer and more robust than the medial limb (Fig 23K). The humeral head makes up much of the central and medial portions of the proximal margin of the humerus, forming a subtrapezoidal structure in anterior view. The elongate deltopectoral crest is not continuous with the proximal surface of the bone, but instead first rises a few centimeters distally down the shaft, continuing for approximately 57% the length of the humerus. The deltopectoral crest is inclined 40° away from the long axis of the shaft proximally before becoming parallel with the shaft halfway through its length. It then inclines toward the shaft making another angle of approximately 50°. In lateral outline, the deltopectoral crest is more subrectangular than subtriangular, and more resembles other sauropodomorphs like *Massospondylus carinatus* [14], *Adeopapposaurus mognai* [77], and *Plateosaurus engelhardti* [98, 99] than the archosauriform *Euparkeria capensis* [131, 132] (Fig 23C and 23G). Proximally, the deltopectoral crest leans slightly posterolaterally and is slightly sigmoidal in anterior profile, but it transitions to being more or less straight and perpendicular to the humeral shaft along its length. The anteroproximal margin of the deltopectoral crest also is rugose, which probably represents the insertion point of the supracoracoideus muscle [135]. Unlike the proximal notch of *Seitaad ruessi* [22], the distal ‘hook’ of *Saturnalia tupiniquim* [97, 136], and the paramarginal sulcus of *Antetonitrus ingenipes* [39], the deltopectoral crest of the humerus of *Sarahsaurus aurifontanalis* does not have any embayment crest in lateral view.

The humeral shaft is thinnest at the distal termination of the deltopectoral crest. The entire shaft is roughly sigmoidal in lateral profile, with the proximal half bowing anteriorly and the distal half bowing posteriorly (Fig 23A). A shallow and wide triangular groove (= cuboid fossa [136]) occurs on the anterior surface of the distal end of the bone that deepens and flares out between the distal condyles. Anteroposterior crushing exaggerated this feature in both humeri of *Sarahsaurus aurifontanalis*. The olecranon fossa on the distal posterior surface is shallow, but little detail is preserved.

The distal end of the humerus is expanded and the condyles and epicondyles are pronounced (Fig 23). The distal and proximal articular surfaces are twisted 30–40° from one another. The ulnar condyle has a pronounced process on the posteromedial margin in distal view. The entepicondyle extends farther than the ectepicondyle in anterior view, but both epicondyles are formed by a roughly triangular expansion of bone that widens anteroposteriorly in lateral and medial views. Like that of *Sarahsaurus aurifontanalis*, the entepicondyle is round in *Euparkeria capensis* [131, 132], the dinosauriforms *Marasuchus lilloensis* [119] and *Silesaurus opolensis* [113], the saurischian *Herrerasaurus ischigualastensis* [93–96], and early sauropodomorphs like *Saturnalia tupiniquim* [97, 136] and *Thecodontosaurus antiquus* [19, 100, 101], suggesting that this is plesiomorphic for sauropodomorphs and dinosaurs in general. In both humeri, the margins of the proximal articular surface and the distal condyles are rough and resemble the rugose patterning found between sauropod joint surfaces that suggest the presence of cartilaginous caps. The mediolateral width of the distal end of the humerus is 30% of the total length of the element.

#### Radius

**Fig 24:**
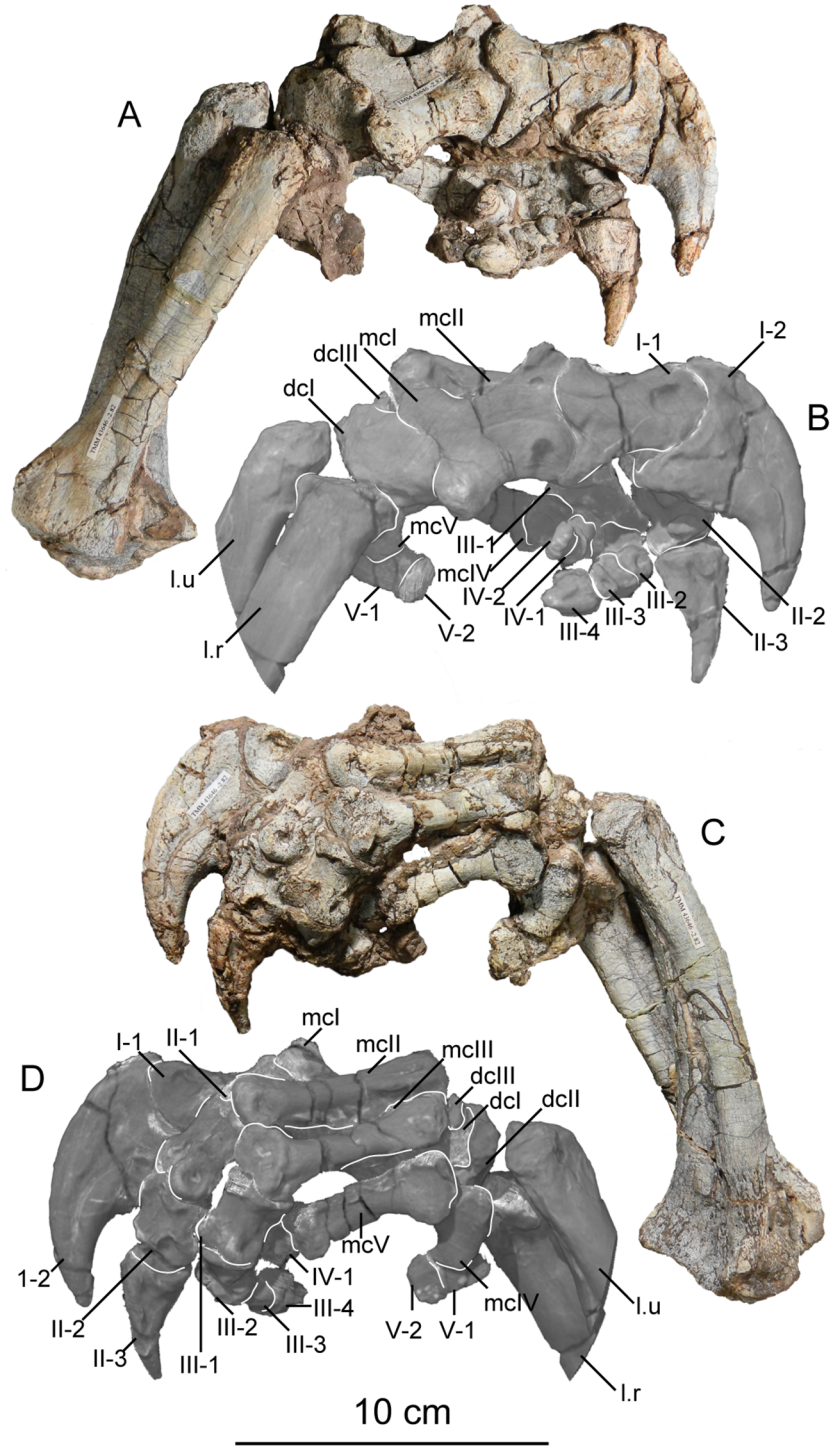
Holotype articulated left antebrachium and manus of *Sarahsaurus aurifontanalis* in color photographs (A and C) and CT-volume-rendered images (B and D). A, B- medial view; C, D- lateral view. Abbreviations: distal carpal (dc), metacarpal (mc), left radius (ra), left ulna (u), digits one to five (I-V).

**Fig 25:**
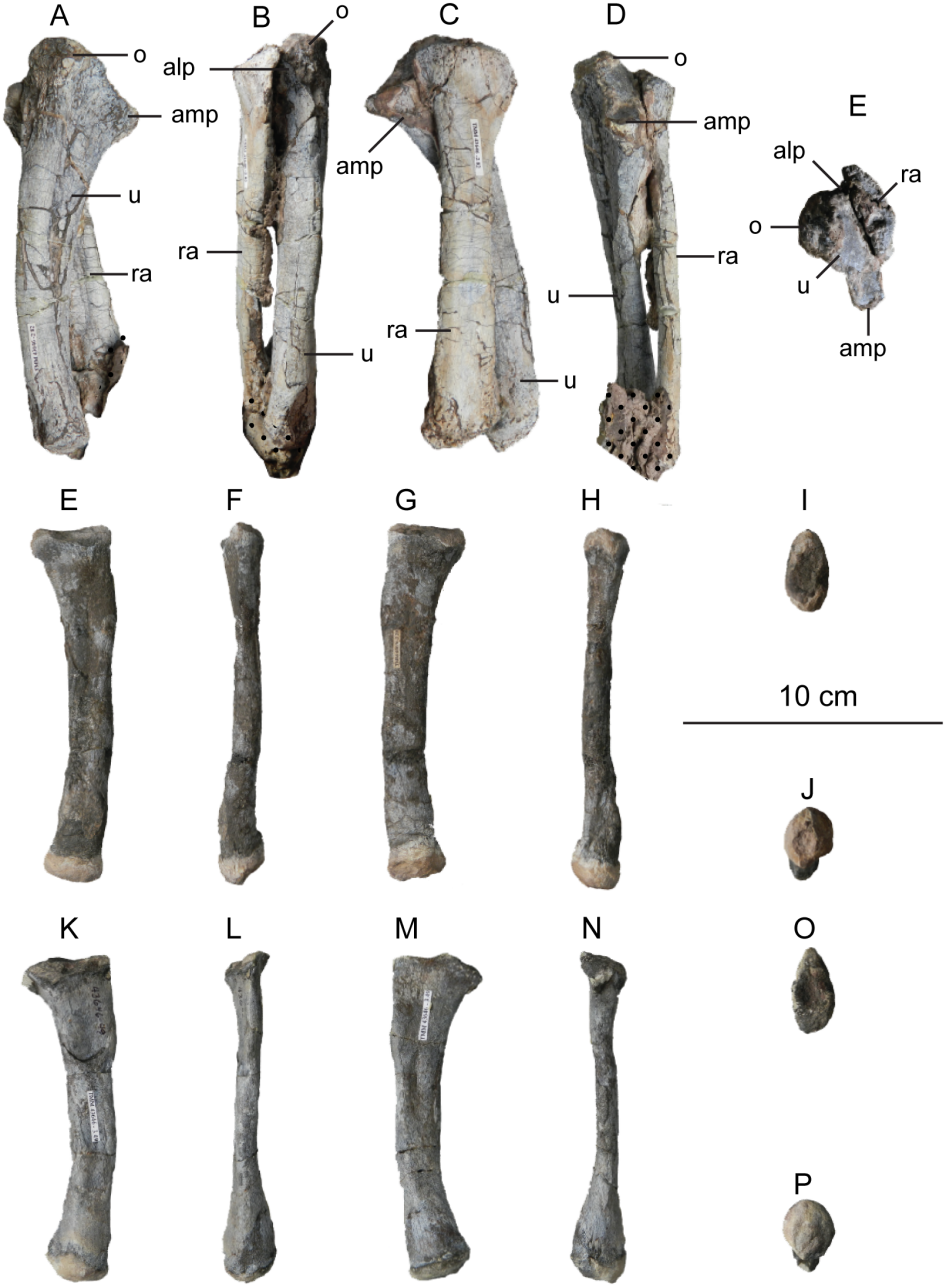
Holotype left radius and ulna (A-E), holotype right radius (E-J), and paratype right radius (K-P) of *Sarahsaurus aurifontanalis*. A, E, K- anterior view; B, F, L- lateral view; C, G, M- posterior view; D, H, N- medial view; E, I, O- proximal view; J, P- distal view. Stippled areas indicate matrix and bone not belonging to the radius or ulna. Abbreviations: anterolateral process (alp), anteromedial process (amc), olecranon (o), radius (ra), ulna (u).

The radius is relatively short and structurally simple (Figs 24 and 25). Its length is 60% of the length of the humerus. Proximally, the articular surface is subtrapezoidal in outline and the shaft transitions into an elliptical cross-section along its length, terminating in an oval distal articular surface. The proximal head is expanded anteroposteriorly with the anterior margin jutting out prominently. The lateral ulnar articulation surface is slightly convex and rests in the medial concavity on the proximal end of the ulna. The shaft does not twist relative to either end of the bone. The distal end is bulbous at its lateral articulation with the ulna, which may represent the attachment site of ligamentous tissue or may even represent a co-ossified radiale, because this region is also in close association with distal carpal 1 (Fig 24). The distal end of the radius also is expanded, but not to the extent of the proximal end. There is a slight kink at the distal end like that found in *Saturnalia tupiniquim* [136], but the overall shape of the radius is most similar to that of *Massospondylus carinatus* [14] and *Adeopapposaurus mognai* [77].

#### Ulna

The ulna also is relatively short; it is 67% of the humeral length. In outline, the proximal end is subtriangular (Figs 24 and 25). The proximal end of the ulna comprises two processes and a smaller ridge. The first is the anteromedial process, a mediolaterally-compressed feature found in saurischians and theropods such as *Herrerasaurus ischigualastensis* [93–96] and *Dilophosaurus wetherilli* [58, 59]. *Sarahsaurus aurifontanalis* also has a ridge extending up the lateral surfaceof the proximal end of the ulna that can be seen as a low anterolateral process proximally. The proximal medial surface of the ulna is in close articulation with the radius; in proximal view, a very shallow fossa lies between the anteromedial process and the short anterolateral ridge, into which the radius articulates. Sauropods and later-diverging sauropodomorphs like *Sefapanosaurus zastroensis* [40], *Pulanesaurus eocollum* [37, 38], and *Melanorosaurus readi* [38, 137] have well-developed anterolateral processes delimiting an associated radial fossa, but the early sauropodomorphs *Plateosaurus engelhardti* [98, 99] and *Massospondylus carinatus* [14] do not have that feature. The olecranon process is expanded posteriorly but does not extend far proximally past the humeral articulation surface on the ulna. This may represent the attachment site of a largely cartilaginous olecranon process like that described in some specimens of *Plateosaurus engelhardti* [98]. Presence of the olecranon process as in *Sarahsaurus aurifontanalis* is plesiomorphic in sauropodomorphs and its absence in sauropods is apomorphic for that group. A broad subtriangular depression makes up the posterolateral surface of the proximal end of the bone and is delimited by the olecranon process, the anterolateral ridge, and a short posterior ridge that extends along the lateral surface from the olecranon process distally to halfway down the shaft.

The ulnar shaft itself is bowed anteriorly, unlike the radius which bows slightly posteriorly along its length. The shaft twists distally so that the mediolateral axes of each end are almost 90° from one another. The distal end of the ulna is subrectangular and rugose, especially along the medial articulation surface with the radius. The rugosity may represent ligament attachment sites or may be the result of the co-ossification of the ulnare to the distal end of the ulna. The ulna is most closely associated with the second and third distal carpals, but also shares an association with distal carpal 1 medially.

#### Carpus

**Fig 26:**
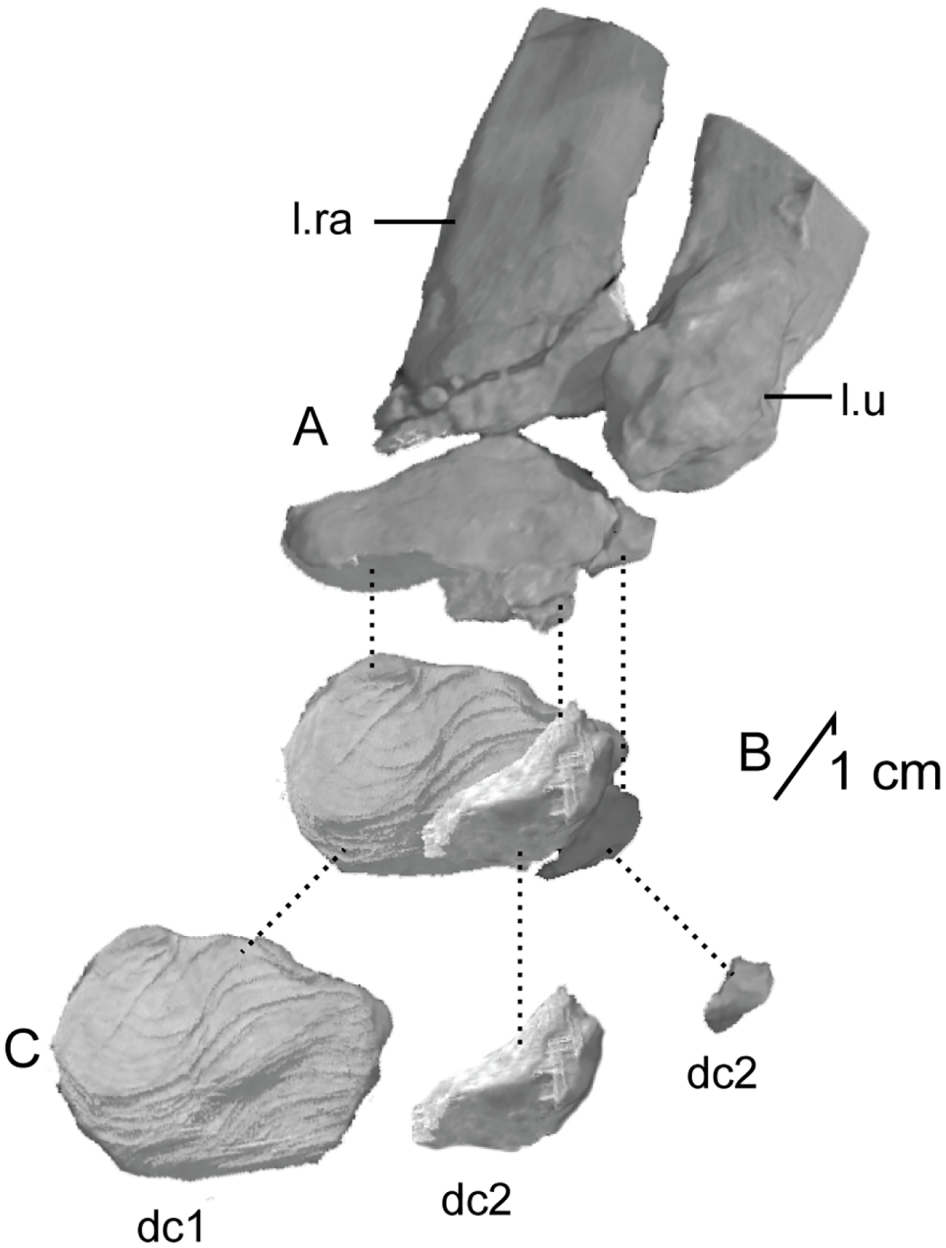
CT-volume-rendered images of left holotype carpus of *Sarahsaurus aurifontanalis* shown articulated with the distal radius and ulna in dorsal view (A) and in articulated distal view (B). Distal carpals 1–3 were digitally disarticulated and are shown in distal view (C). The arrow in B and C points dorsally. Abbreviations: distal carpal (dc), left radius (ra), left ulna (u).

The carpus of *Sarahsaurus aurifontanalis* was preserved in close articulation with the distal end of the radius and ulna and the proximal metacarpus (Fig 26). Three distal carpals are ossified. The radiale and ulnare are absent in *Sarahsaurus aurifontanalis*, but are present in early theropods like *Herrerasaurus ischigualastensis* [93–96]. They either failed to ossify or are co-ossified with the distal margin of the radius and ulna, respectively. The distal ends of the radius and ulna are extensively crushed, making interpretations difficult. Because of the extremely close articulation of the ulna, radius, and three distal carpals, and the metacarpus, it seems unlikely that any proximal carpals that may have ossified were lost post-mortem (Fig 26B and 26D). A fair degree of overlap is present in the three carpal elements and this is almost identical to the arrangement of the distal carpals of *Massospondylus carinatus* [14].

Distal carpal 1 is the largest of the three carpal elements and is a robust bone with a subtrapezoidal distal outline. It is thickest at its ventral and medial margins and thins towards its articulations with the distal carpals 2 and 3. The proximal surface is gently convex but it flattened at its association with the radius on its proximoventral surface. The medial half of its distal surface is smooth and slightly convex for the articulation with metacarpal I (Fig 26B). Unlike *Riojasaurus incertus* [114, 115], no sulcus is observed along the medial half of distal carpal 1 in *Sarahsaurus aurifontanalis*. The lateral half of the distal articular surface transitions into a concave surface to articulate with distal carpal 2, and thins at its most lateral margin to articulate with distal carpal 3.

Distal carpal II articulates with the distal surface of distal carpal 1 (Fig 26A and 26B). The second distal carpal is significantly smaller than the first. Its distal outline is subrhombohedral and the rounded proximal surface fits into the distolateral concavity of distal carpal 1. The dorsal surface of distal carpal 2 thickens proximodistally and mediolaterally into a knob-like distal projection that articulates with the proximoventral margin of metacarpal II as well as the proximomedial surface of metacarpal III. Only the most lateral and ventral edge of the second distal carpal is associated with distal carpal 3 and metacarpal IV.

The third distal carpal is significantly smaller than distal carpal 2. This wedge-shaped bone is thickest dorsally and laterally, and thins significantly laterally and towards its ventral edge (Fig 26B). The medial surface is slightly concave where it articulates with distal carpal 1. Only the most distal edge of this surface articulates with distal carpal 2. Some amount of post-mortem displacement of these elements may have occurred, resulting in the extreme lateral position of distal carpal 3 within the carpus such that it only weakly associates with the fifth metacarpal along its most lateral surface.

#### Metacarpus

**Fig 27:**
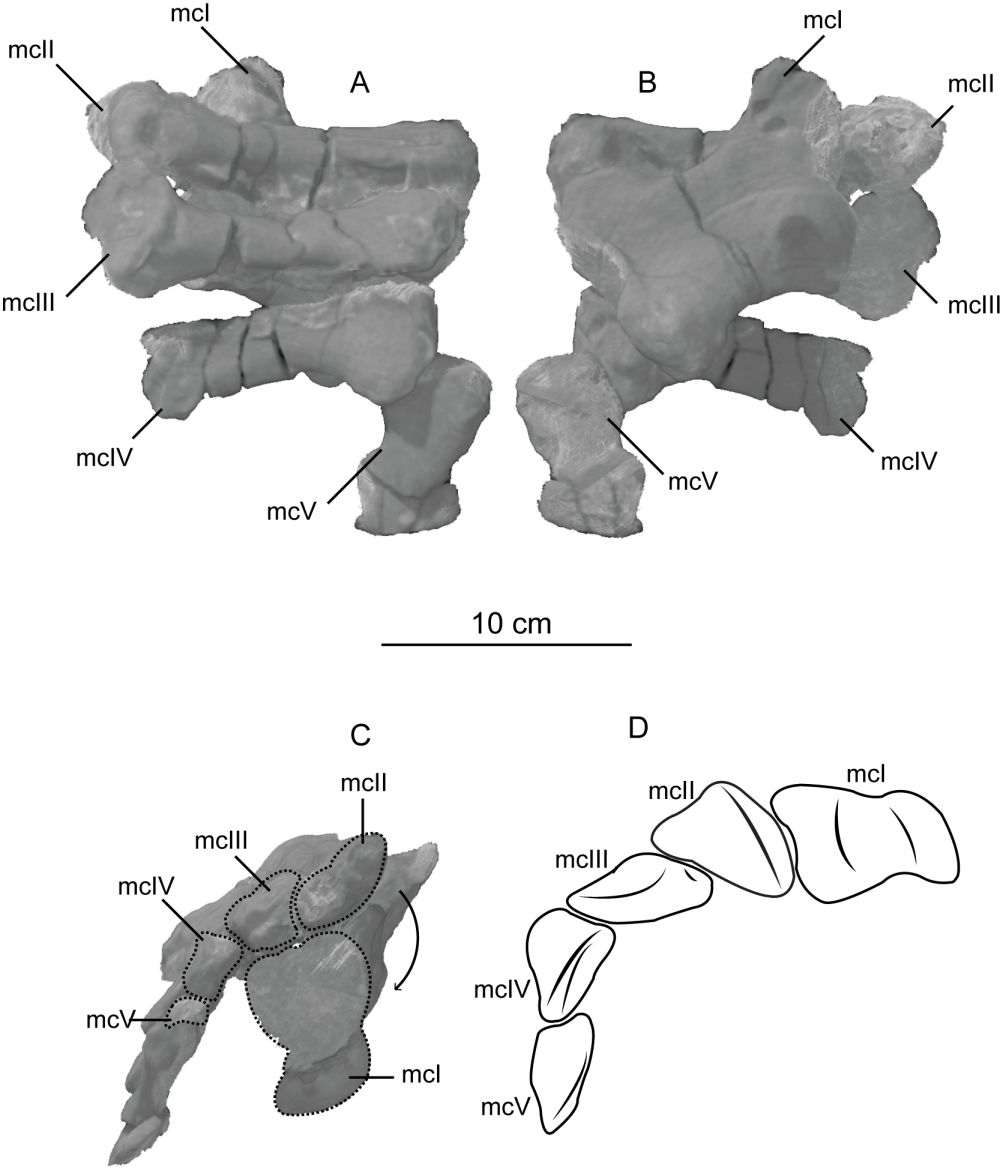
CT-volume-rendered images of the articulated holotype left metacarpus of *Sarahsaurus aurifontanalis* in lateral (A), proximal (B), and medial (C) view. Metacarpal I (along with the ulna and the rest of digit I) was crushed medially toward the ventral surface post-mortem (see arrow in C). D displays the proximal outlines of the left metacarpus reconstructed from the elements from the left and right manus. Abbreviations: metacarpal (mc).

**Fig 28:**
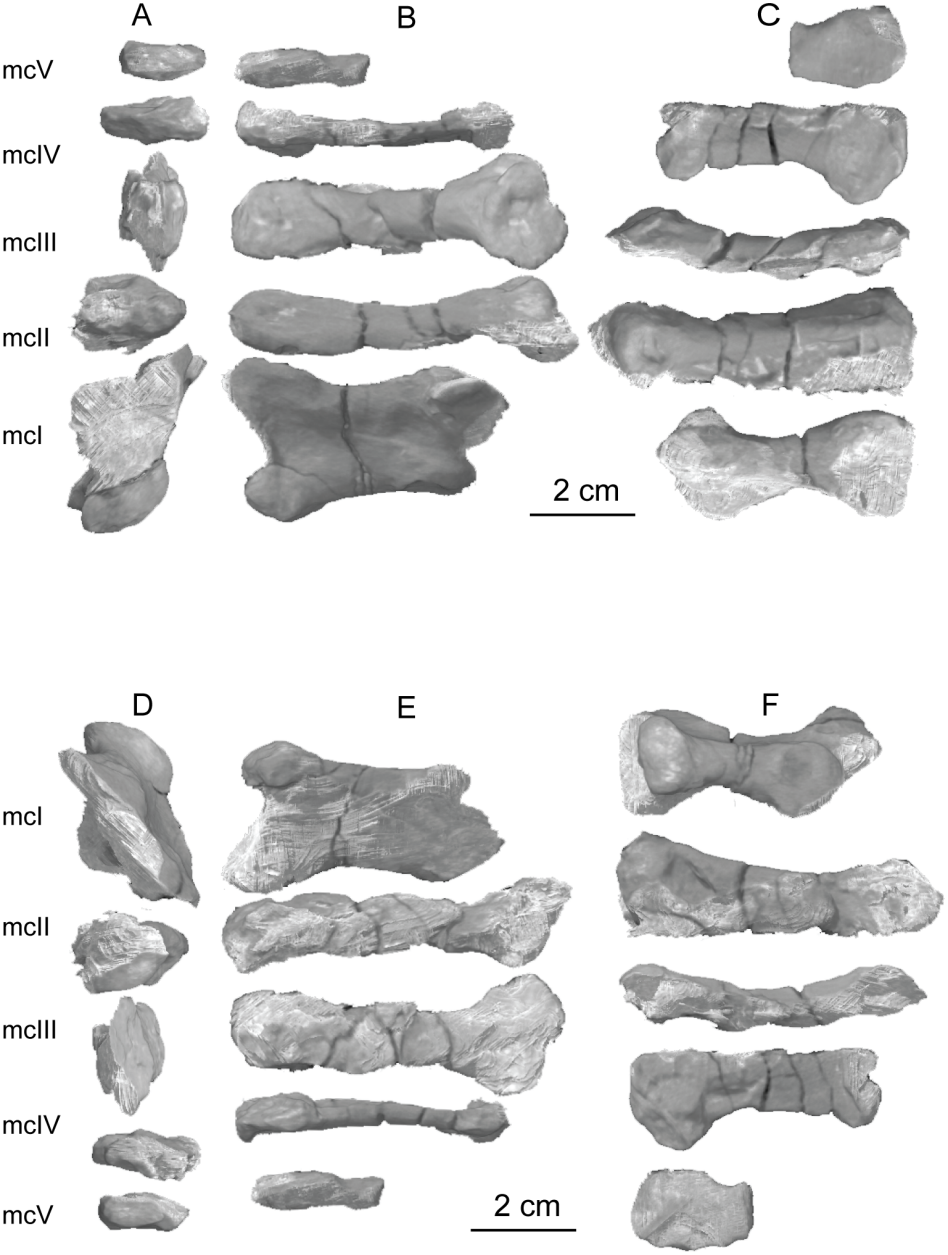
Digitally disarticulated CT-volume-rendered images of the holotype left metacarpals of *Sarahsaurus aurifontanalis*. A- proximal view; B- dorsal view; C- lateral view; D- distal view; E- ventral view; F- medial view. Abbreviations: metacarpal (mc).

The left metacarpus of the holotype specimen is preserved in close articulation, but the elements were shifted in relation to one another during or after burial. The articulated left manus of the holotype was crushed and distorted post-mortem. CT imaging greatly enhanced our ability to interpret the position and articular surfaces of these elements (Figs 27 and 28). The position of metacarpal I was altered most dramatically, crushing all of digit I medially and ventrally beneath the other metacarpals. The holotype specimen also includes manual elements from the right side, including partial digits II and III and complete, articulated digits IV and V. Appendix C (S3 Text) displays the lengths of the metacarpals from the paratype and holotype specimens.

Reconstructions based upon CT images of the left metacarpus and elements preserved from the right metacarpus show that the metacarpus curves gently outwards, and the metacarpals of *Sarahsaurus aurifontanalis* were spread much more than the metacarpals of sauropod dinosaurs (Fig 24D). *Sarahsaurus aurifontanalis* also held the first three metacarpals in line with one another, but the fourth and fifth metacarpals were angled downwards. This overall shape is identical to that of the manus found in well-preserved, articulated specimens of *Massospondylus carinatus* [14], *Plateosaurus engelhardti* [98, 99], and *Anchisaurus polyzelus* [7, 8, 32].

Metacarpal I is distinctive and is similar in structure to that of most early sauropodomorphs (Fig 28). Its greatest transverse width is only slightly less than the length of metacarpal II, which is the longest element in the metacarpus. Metacarpal I and the rest of digit I are robust. The first metacarpal is aligned with the rest of the metacarpals proximally, and is not inset into the carpus as in other early sauropodomorphs like *Adeopapposaurus mognai* [77], *Anchisaurus polyzelus* [7,8, 32], *Lufengosaurus hueni* [78, 79], *Plateosaurus engelhardti* [98, 99], and *Massospondylus carinatus* [14]. However, in *Sarahsaurus aurifontanalis* that is probably a result of post-mortem reorientation of digit I with respect to the rest of the manus. Digit I was rotated ventromedially, causing the digit to become displaced towards the ventral surface (Fig 27C). Metacarpal I articulates along its concave proximal surface closely with distal carpal 1. The proximal outline is subtrapezoidal and tapers medially (Fig 28A). The proximolateral process of metacarpal I is expanded broadly along its lateral margin, creating a subtriangular surface that is inset by a shallow fossa into which metacarpal II articulates.

The proximal width of the first metacarpal is 60% of the total length of the element. The width of the shaft of metacarpal I is thinnest at its mid-length and then expands distally into asymmetrical distal condyles, which are separated by a shallow groove on the dorsal surface. The medial distal condyle houses a broad medial ligament fossa and is shorter and rounder than the lateral distal condyle, which extends more distally and is larger than the medial distal condyle. No such fossa is preserved on the lateral surface of the lateral distal condyle, but that may be an artifact of digital preparation and the lack of contrast in density in the CT data between matrix and bone in that region. The strong asymmetry of the distal condyles of metacarpal I in *Sarahsaurus aurifontanalis* is plesiomorphically present in Sauropodomorpha and Sauropoda, but is lost in neosauropods like *Giraffatitan brancai* [138] and *Apatosaurus excelsus* [139] (Fig 28D). The axis through the distal condyles is twisted 25° medially relative to the proximal lateral and medial processes.

**Fig 29:**
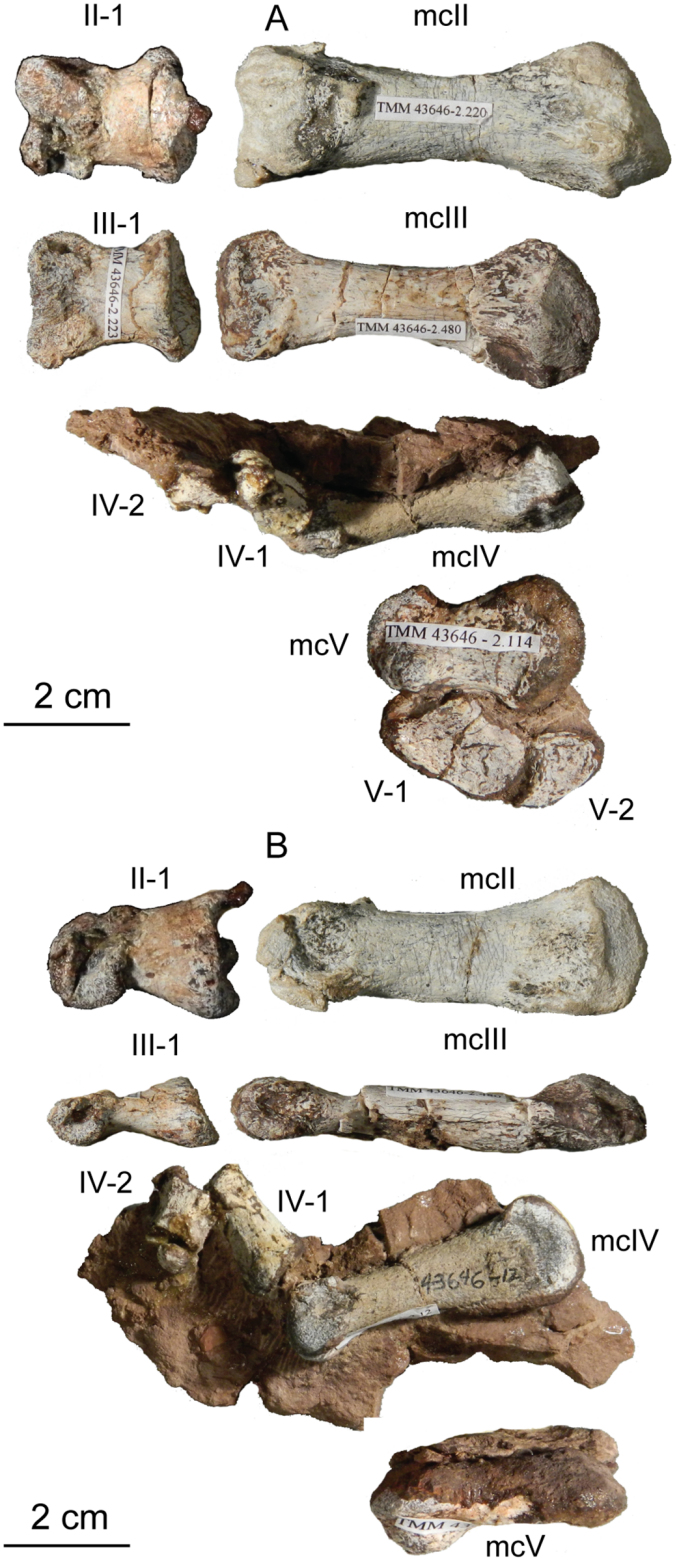
Holotype right metacarpals and manual phalanges of *Sarahsaurus aurifontanalis*. A- dorsal view; B- medial view. Abbreviations: metacarpal (mc), digits one through five (I = V).

Metacarpal II is the longest element in the metacarpus (Fig 28B). The bone is subtriangular in proximal outline, with its apex pointed laterally. The ventral surface is divided unequally by a ridge extending distally from the proximal articulation. This creates a recessed platform or fossa on the proximolateral margin of the ventral surface of metacarpal II that can be seen in lateral view. The dorsal surface also has such a ridge that creates a similar slope lower on its proximolateral margin (Fig 29A). In lateral view, metacarpal II is fairly robust, and slims only slightly towards its distal end. The shaft is subelliptical in cross-section. The distal condyles are both excavated by collateral ligament pits, the medial of which is more pronounced. The second metacarpal has a larger medial distal condyle than its lateral counterpart. The distal condyles are separated along their dorsal and ventral surfaces by a shallow groove. The distal condyles are twisted approximately 50° relative to the proximal end of the bone.

The third metacarpal is slightly shorter than metacarpal II, and is slightly longer than the longest transverse length of metacarpal I (Fig 28B). The distal end of the element is badly compressed and flattened in the left side of the holotype, but is less compressed on the right side. Metacarpal III is not as tall dorsoventrally as metacarpal II, although its proximal end is slightly taller than its distal end and has a subtrapezoidal proximal outline that is pinched medially. In dorsal view, the proximal end of metacarpal III is broad and flat and thins distally until it expands at the distal condyles. The ventral surface of metacarpal III is fairly flat, except for a very shallow fossa directly underneath the raised knob on the proximal dorsal surface. The dorsal surface of the third metacarpal is divided on its proximal half by a longitudinal ridge that forms a raised medial border to a small inclined lower shelf on the lateral edge of the bone (Fig 29A). The distal condyles are subymmetrical and lack the dividing groove between them seen in metacarpal II, which also results in a subtrapezoidal distal outline. Both distal condyles have shallow collateral ligament pits that are wider than they are tall, suggesting that this element underwent mild compression. The axis through both distal condyles is twisted 15° from the main proximal articular axis.

The length of metacarpal IV is approximately 65% of the length of metacarpal II (Fig 28). The fourth metacarpal is severely compressed dorsoventrally in the left holotype, but is unaltered on the right side. In proximal view, metacarpal IV is subtrapezoidal, with a medial edge that is taller than the lateral edge. Viewing the ventral surface, the medial margin of the proximal end of metacarpal IV expands medially and proximally beyond the proximal extent of the lateral margin. Moving distally, the bone thins only slightly before expanding again at the distal condyles. Viewed laterally, the bone is pinched at a quarter of its length after a broad, flat surface and continues to thin slightly before reaching the distal condyles (Fig 29B). The distal condyles themselves are inclined slightly medially and host a shallow dividing groove. The distal outline of metacarpal IV is very similar to that of metacarpal II, except for the inclined distal condyles. A lateral ligament fossa is present, but a medial fossa can only be presumed as it is not preserved or exposed in either specimen.

The fifth metacarpal is the smallest in the manus, being roughly half the size of metacarpal IV (Figs 28 and 29). It closely resembles the fifth metacarpal of *Plateosaurus engelhardti* [98, 99] and *Massospondylus carinatus* [14], but is not as long and gracile as that of *Seitaad ruessi* [22] and *Adeopapposaurus mognai* [77]. The distal end of metacarpal V is crushed in the left side of the holotype but is preserved on the right side. In dorsal view, the proximal end of the element is wider than the distal end, but the bone flares again mediolaterally at the distal end. The proximal end is broadly convex and subtriangular in proximal outline, associated with distal carpal 1 along its ventral and medial margin and the third distal carpal on its extreme proximal edge. The distal end is more round and highly convex where the asymmetric distal condyles articulate with the first phalanx of digit V. Only a trace of a collateral ligament fossa is discernible on the larger medial distal condyle.

#### Manual phalanges

**Fig 30:**
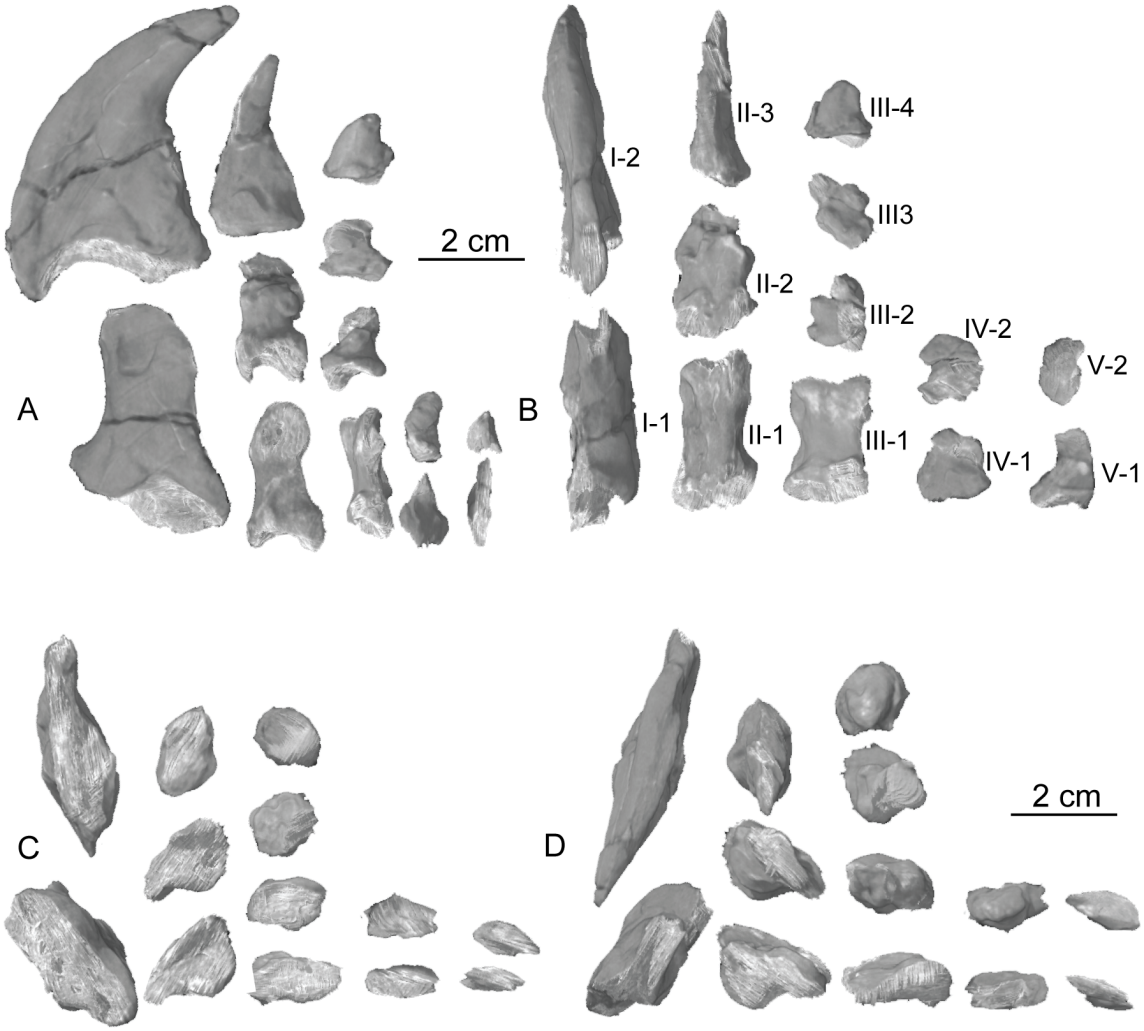
Digitally disarticulated CT-volume-rendered images of the holotype left manual phalanges of *Sarahsaurus aurifontanalis*. A- medial view; B- dorsal view; C- proximal view; D- distal view. Abbreviations: digits one through five (I-V).

All the manual phalanges are present in the left manus of the holotype specimen, however the distal phalanges of digits IV and V are difficult to interpret. Fortunately, those elements are preserved in the right manus of the holotype (Fig 24). Thus, every element in the manus is known for *Sarahsaurus aurifontanalis*. The phalangeal count is 2-3-4-2-2, and having two phalanges on digit V is unique among early sauropodomorphs (Figs 24, 29 and 30). Fig 30 displays digitally disarticulated manual phalanges from the left manus of the holotype and Fig 29 displays elements from the right holotype manus.

The first phalanx of digit I (I-1) is a prominent bone in the manus. Proximally, phalanx I-1 is subrectangular in outline, and a small ridge divides the proximal surface into medial and lateral concave facets (Fig 30C). Those two regions are approximately equal in area. The ridge corresponds to the groove between the distal condyles of metacarpal I, which articulate into the concave regions of the proximal surface of the first phalanx. The distal condyles of that phalanx are twisted almost 80° relative to the proximal surface (Fig 30D) similar to massospondylids and unlike the relatively more robust and less-twisted first phalanx of *Antetonitrus ingenipes* [39] and *Lessemsaurus sauropoides* [140]. This arrangement of metacarpal I and the first phalanx of digit I is common in early sauropodomorphs, where the entire digit is reflected medially. A broad depression makes up a significant portion of the lateral side of I-1, but this is due to its being crushed against the articulation of metacarpal II and the first phalanx of digit II. The distal condyles of I-1 flare out along their ventral margins, and the medial condyle extends slightly further distally and ventrally than the lateral condyle. Collateral ligament fossae are well established on the lateral and medial surfaces of the distal condyles and while they are not very deep, both are broad and oblong proximodistally (Fig 30A). A deep groove divides the distal and ventral ends of the distal condyles of I-1, articulating with a ridge on phalanx I-2.

Phalanx 2 of digit I is a large, trenchant ungual claw and the largest ungual in the manus (Fig 30A); it is 150% longer than the first metacarpal. A ridge separates the proximal articulation surface into medial and lateral facets, of which the medial is larger and makes up more of the articular surface. The ridge and the articular surfaces slot into the distal condyles of I-1. A strong flexor tubercle is present on the ventral edge of the proximal surface of I-2 (Fig 30C). A longitudinal groove extends proximodistally from the distal tip of the claw along its medial and lateral surfaces, terminating their arcs at the base of the flexor tubercle. On the medial side, the groove bifurcates, and a shallower dorsal portion extends for a short length dorsally towards the extensor tubercle. Again, a shallow depression is found on the lateral side distal to the flexor tubercle, but that results from compression against the articulation of II-2 and II-3 during burial.

Phalanges 1 and 2 of digit II are similar in form, with II-2 being slightly shorter in than II-1 (Fig 30). The proximal outlines of these phalanges are subtriangular, with the dorsal apex of the triangle leaning medially and extending proximally (Fig 30C). This surface itself is divided into two regions that articulate with the distal condyles of the preceding element. In dorsal view, the medial lean of the proximally-extending dorsal process exposes the lateral articular facet. The shaft remains subtriangular in II-1 and II-2 until the distal condyles broaden the distal end of the bones. Round distal condyles form the distal ends of both elements and the condyles are inclined medially in distal view (Fig 30D). Well-developed collateral ligament fossae are found on the lateral and medial distal condyles of II-1. However, owing to post-mortem cracks in the bone, only the lateral distal condyle of II-2 exhibits such a fossa.

As in digit I, digit II terminates in an ungual claw but the length of II-3 is less than half that of I-2, and the claw is not nearly as recurved along its length (Fig 30A). II-3 is subconical in shape and differs greatly from I-2 in overall morphology. There is a trace of a ridge dividing the proximal surface into two articular facets that articulate with the distal condyles of II-2. A shallow groove divides the lateral and medial sides of phalanx 3 of digit II into dorsal and ventral regions. A strong flexor tubercle is absent in this ungual, but is weakly established along the extreme distal and ventral margin.

The first three phalanges of digit III are similar, only decreasing in size sequentially (Fig 30). Unlike digit II, phalanges III-1, III-2, and III-3 have a suboval proximal articular outline, and the ventral edge of that surface slightly extends proximally past the dorsal edge. The ridge dividing the proximal medial and lateral articular facets, if present, is extremely low. These three phalanges pinch slightly at mid-shaft, but do exhibit prominent distal condyles, separated dorsally by a shallow groove that articulates into a doubly-concave proximal surface of the next bone. Collateral ligament fossae are found in the medial and lateral distal condyles of III-1, III-2, and III-3. As is the case for II-2, any absence of a fossa in the distal condyles of those elements is probably an artifact of poor preservation.

Phalanx III-4 caps the fourth digit and is a blunt, slightly curved ungual that is roughly one-third of the length of the ungual of digit II (Fig 30A). This element is quite different in shape than the larger ungual of digit III of *Plateosaurus engelhardti* [98, 99], *Adeopapposaurus mognai* [77], and *Massospondylus carinatus* [14], but is most similar to the small, blunt ungual of *Melanorosaurus readi* (NMQR 3314) [137]. Its proximal articular surface is like that of I-2 and II-3, and a trace of a longitudinal groove on the medial surface is preserved.

The phalanges of digit IV are poorly preserved in the left holotype manus, but are complete and in articulation from the right side (Fig 29). Phalanx 1 of digit IV is similar in form to the second phalanx of digit III, but is slightly larger. The distal condyles do not seem to be separated by a depression, but are excavated by circular collateral ligament fossae. Phalanx IV-2 is a claw slightly smaller than that of the third digit and resembles a rounded thumb-tack in overall shape, like that of *Plateosaurus engelhardti* [98, 99]. The proximal articular surface of IV-2 is slightly concave, but is not divided into two sloping surfaces.

The two phalanges of digit V are preserved in articulation in the right side of the holotype (Fig 29). Phalanx V-1 is subrectangular in lateral view, with a dorsal process that articulates with the small groove in between the distal condyles of metacarpal V. In dorsal view, the proximal end of V-1 is the widest region of the bone. The distal end of the first phalanx of digit V is semicircular in lateral view, and articulates with a small, round ungual phalanx comparable in size to that of digit IV.

### Pelvic girdle

Every element of the pelvic girdle is well represented in the two specimens of *Sarahsaurus aurifontanalis*. The holotype comprises complete pairs of ilia, pubes, and ischia. Only the pubic aprons and ischial obturator plates are incomplete. The ilia were articulated with the sacralvertebrae in the quarry. Although the sacral ribs are fused to one another, they were not completely fused to the medial surface of the ilia. The right ilium remains in its original position within the sacrum, but the other elements of the holotype are disarticulated. The paratype specimen includes the complete left side, except for the missing proximoventral corner of the pubis. A partial right pubis of the paratype is also preserved. There is no major difference in relative length or shape between the elements of the two individuals.

#### Ilium

**Fig 31:**
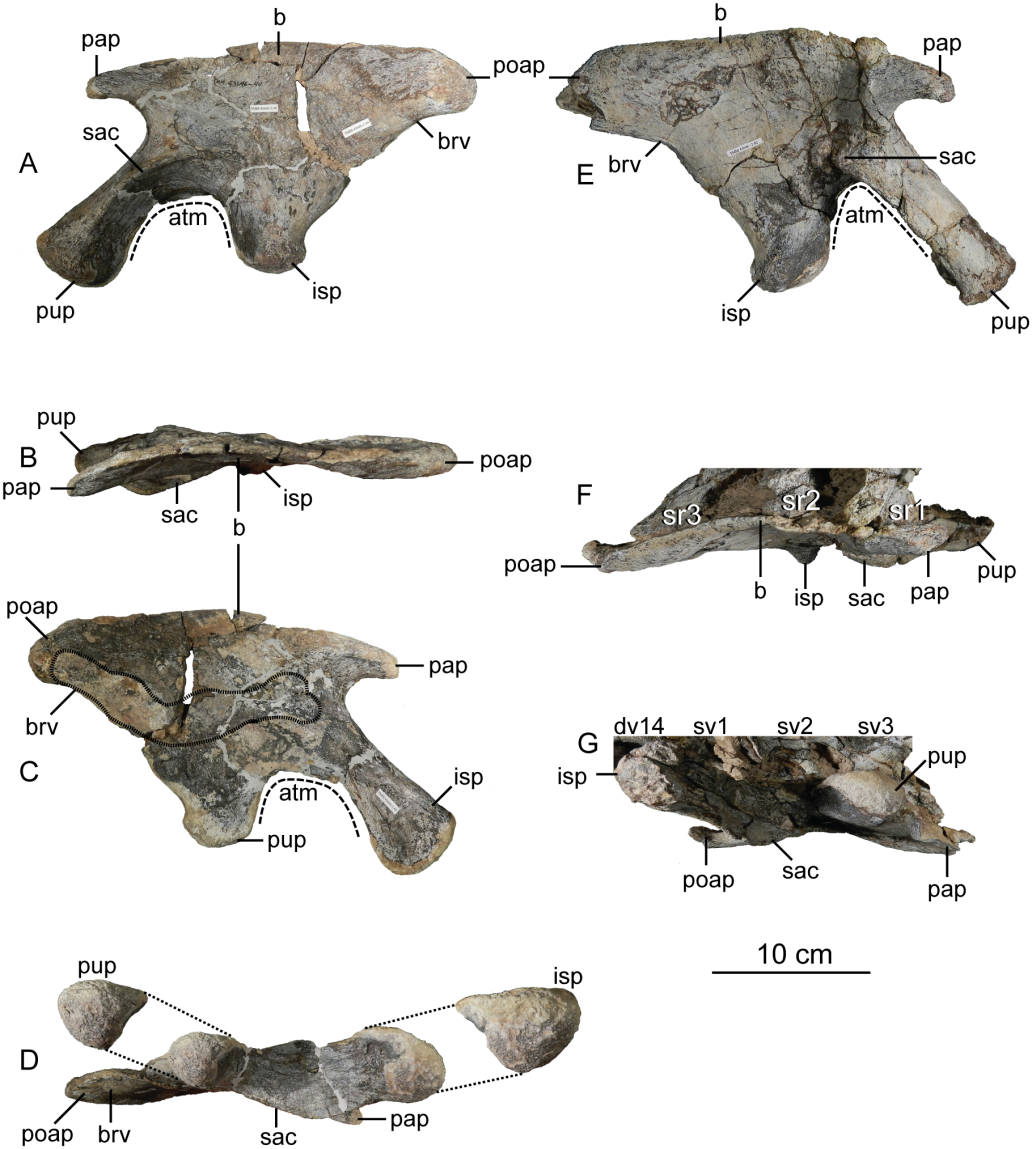
Left (A-D) and right (E-G) holotype ilia of *Sarahsaurus aurifontanalis*. A, E- lateral view; B, F- dorsal view; C- medial view; D, G- ventral view. Dashed line in C surrounds medial scars made by the sacricostal yoke. The distal outlines of the pubic and ishial peduncles are provided in D. Abbreviations: acetabular margin (atm), blade (b), brevis fossa (brv), dorsal vertebra (dv), ischiac peduncle (isp), preacetabular process (pap), postacetabular process (poap), pubic peduncle (pup), supraacetabular crest (sac), sacral rib (sr), sacral vertebra (sv).

**Fig 32:**
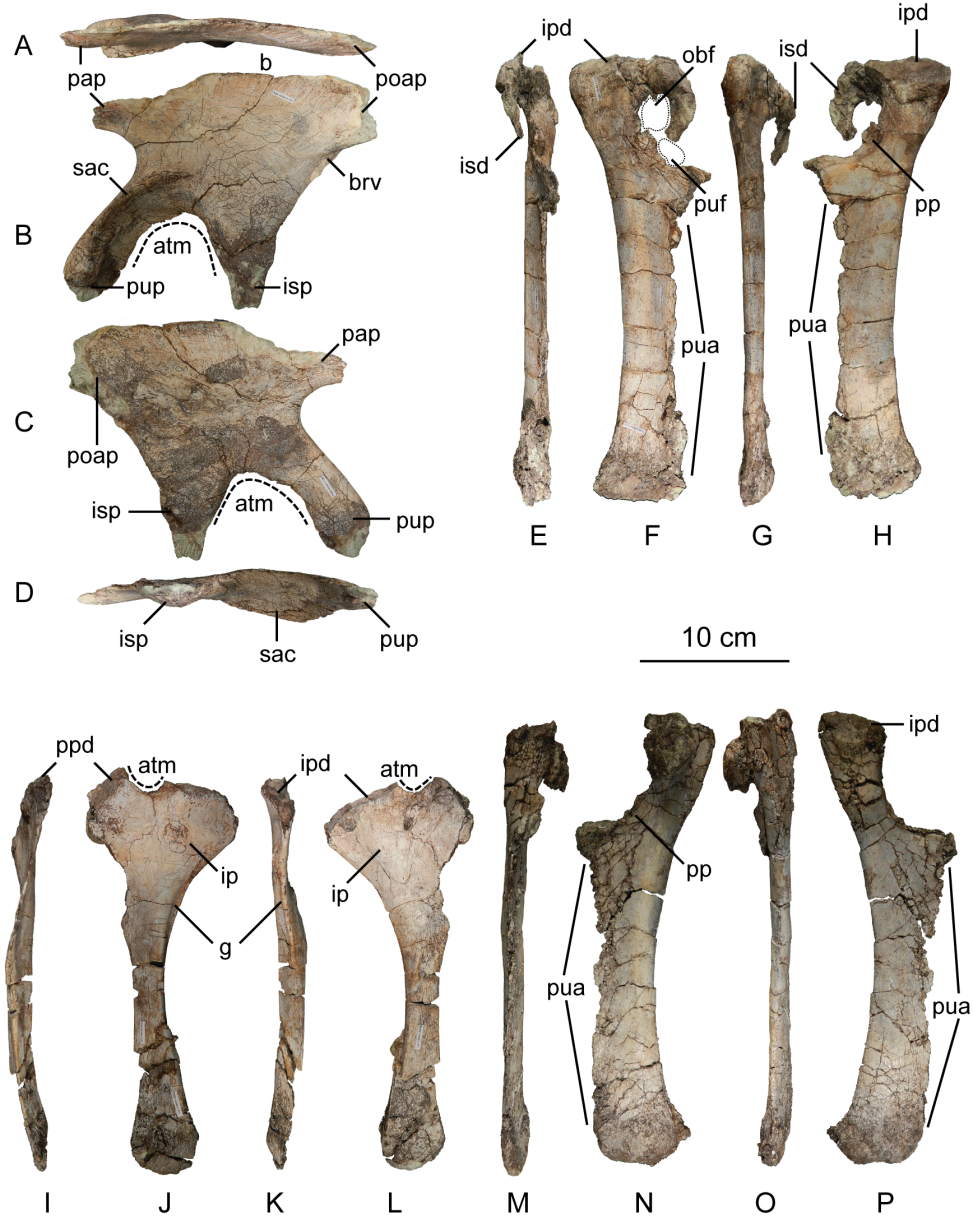
Left paratype ilium (A-D), left paratype pubis (E-H), left paratype ischium (I-L), and right paratype pubis (M-P) of *Sarahsaurus aurifontanalis*. A- dorsal view; D- ventral view; B, E, L, M- medial view; C, G, J, O- lateral view; F, K, N- posterior view; H, I, P- anterior view. Abbreviations: acetabular margin (atm), blade (b), brevis fossa (brv), groove (g), ischiac plate (ip), iliac pedicel (ipd), ischiac peduncle (isp), obturator foramen (obf), preacetabular process (pap), postacetabular process (poap), pubic plate (pp), pubic pedicel (ppd), pubic apron (pua), pubic foramen (puf), pubic peduncle (pup), supraacetabular crest (sac).

The ilium resembles that of most early plateosaurian sauropodomorphs and comprises elongate pubic and ischiadic peduncles, a short preacetabular process of the iliac blade, and a longer, more robust postacetabular process (Figs 31 and 32A–32D). The long dorsal edge of the blade is thin and only weakly convex in lateral view, unlike the strongly curved ilium found in later-diverging sauropodomorphs such as *Meroktenos thabanensis* [24] and *Antetonitrus ingenipes* [39], and sauropods [84, 85]. In dorsal view, the blade is S-shaped; the anterior half bows inward and the posterior half arches laterally (Fig 31B and 31F). The dorsal margin of the ilium thickens towards the posterior end. A shallow, semielliptical depression is visible on the lateral surface of the anterior two-thirds of the blade of the ilium, but it does not project below the ventral margin of the preacetabular process. The preacetabular process is mediolaterally thin and tapers to a round point anterolaterally. The process is relatively short and does not extend as far anteriorly as the pubic peduncle. The preacetabular process of the ilium of *Mussaurus patagonicus* [41], *Leonerasaurus taquetrensis* [30], and other later-diverging sauropodomorphs such as *Tazoudasaurus naimi* [141] is much longer and extends as far or farther anteriorly as the pubic peduncle of the ilium. The preacetabular process joins the main body of the ilium dorsal to the acetabulum (Fig 31A and 31E). The postacetabular process is not blunt and rectangular in lateral view, but instead comes to a rounded point in the posteroventral corner below its convex dorsal margin. The postacetabular process does not project very far backwards unlike that of *Leonerosaurus taquetrensis* [30].

In the left holotype ilium of *Sarahsaurus aurifontanalis*, a rough circular patch of bone lies on the lateral surface of the postacetabular process and is probably pathological. A small depression beneath the posteroventral corner of this process faces medioventrally and represents the area occupied by the brevis fossa, which serves as the insertion of the muscle *caudofemoralis brevis* that originates at the front end of the tail [75]. In *Sarahsaurus aurifontanalis*, the flat area that might be homologous to the brevis fossa only extends for a few centimeters anteroposteriorly along the ventral margin of the postacetabular process and is bounded laterally by a short ventrolateral ridge. The ventral margin of the postacetabular process slopes seamlessly into the posteroventral margin of the ilium and the dorsal margin of the posterior edge of the ischiadic peduncle.

The ventral half of the ilium is much thicker, owing to the mediolateral expansions of the pubic and ischiadic peduncles and roof of the acetabulum (Fig 31D and 31G). The pubic peduncle is longer than the ischiadic peduncle. It extends anteroventrally at an angle of 45° from the body of the ilium, whereas the ischiadic peduncle points straight downwards. A prominent supra-acetabular crest projects laterally from the pubic peduncle (Fig 31A). It begins 2 cm from the base of the peduncle and arches over the acetabulum posteriorly onto the body of the ilium, receding before it reaches the ischiadic peduncle. In all, the supra-acetabular crest delimits one-quarter of a circle. It does not connect to the ventrolateral ridge bounding the brevis fossa. The tallest point along the crest is at its midpoint, where the pubic peduncle meets the body of the ilium.

The acetabulum of *Sarahsaurus aurifontanalis* is semicircular and completely open like most early sauropodomorphs and all sauropods (Fig 31A). The medial and lateral margins of the acetabulum approximate one another and are confluent with the ventral margin of the supra-acetabular crest, forming a broad, concave flat shelf that faces posterolaterally. A subrectangular depression can be observed on the posterior surface of the distal pubic peduncle bounded laterally and medially by short ridges. In distal view, the pubic peduncle is subtriangular in outline and contains a sharp projection on the posteromedial corner, which marks the ventral extent of the sharp ridge forming the medial margin of the acetabulum (Fig 31D). A similar projection can be found on the ischiadic peduncle in *Coloradisaurus brevis* [27, 142], *Lufengosaurus hueni* [78, 79], *Plateosaurus engelhardti* [98, 99], and *Riojasaurus incertus* [114, 115]. The ischiadic peduncle of *Sarahsaurus aurifontanalis* is subcircular in outline and is approximately as wide mediolaterally as it is anteroposteriorly (Fig 31D). In lateral view there is a round, concave notch between the posteroventral corner of the ischiadic peduncle and the posterior margin of postacetabular process of the ilium.

Medially, the ilium is fairly simple in structure. The most obvious features are found along the anteroposterior midline. That region bears rugose scars where the ilium made contact with the three ribs of the sacrum. The holotype left ilium is especially rugose, indicating a greater degree of contact and possible beginning of co-ossification of those elements (Fig 31C). The sacral rib scars are a winding shallow groove of rough bone that mirrors the distal shape of the sacral ribs. Above that long groove lies the thinner section of the iliac blade, which is covered in long striations spanning the transverse distance between the dorsal margin of the ilium and the dorsal extent of the sacral rib scars. The medial surface of the pubic peduncle is slightly concave dorsally and flattens ventrally. The entire ischiadic peduncle has a flat medial surface.

#### Pubis

**Fig 33:**
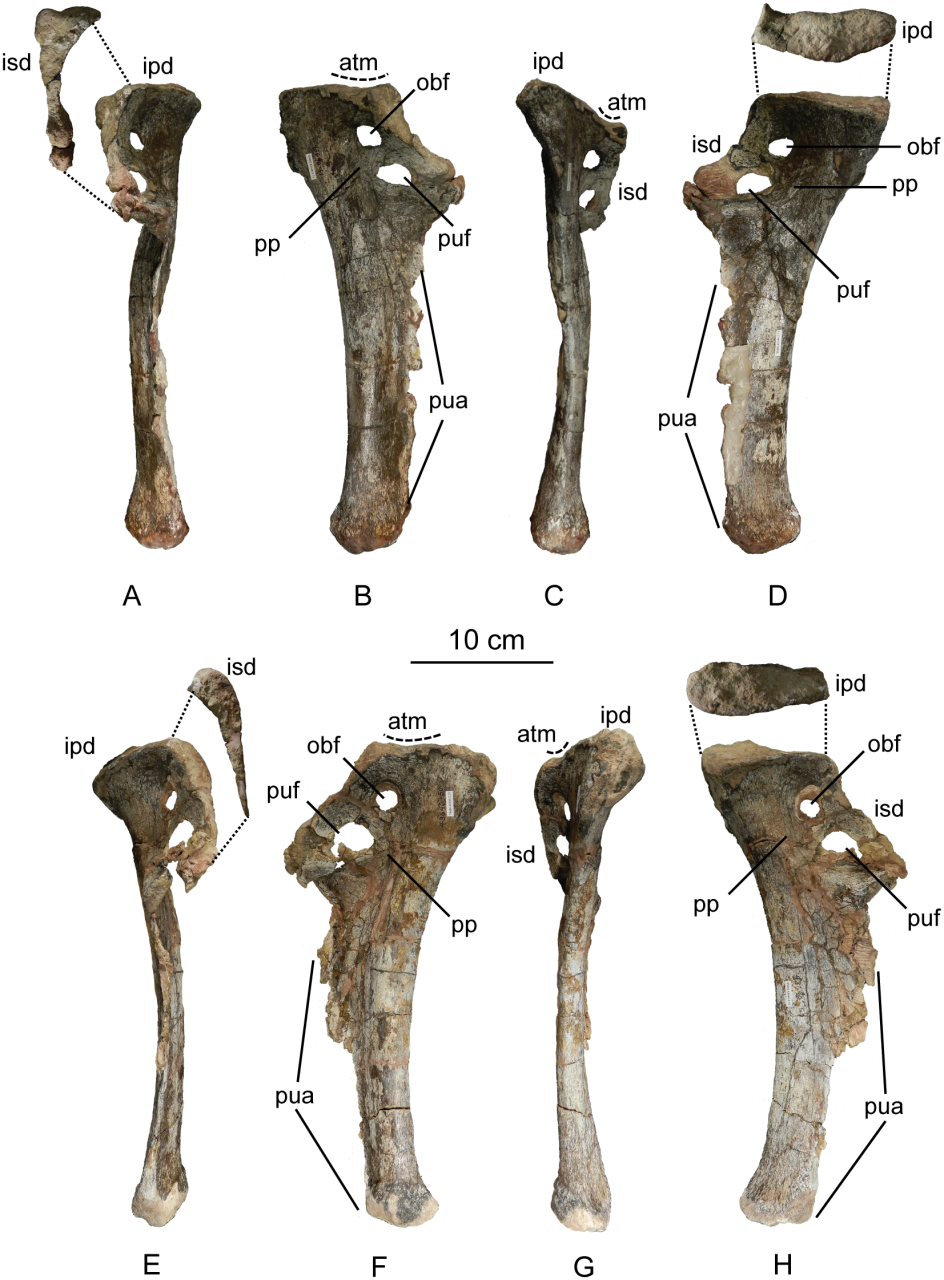
Left (A-D) and right (E-H) holotype pubes of *Sarahsaurus aurifontanalis*. A, E- medial view; B, F- posterior view; C, G- lateral view; D, H- anterior view. The proximal outlines of the ischiadic and iliac pedicels are provided in A and E, and C and G, respectively. Abbreviations: acetabular margin (atm), depression (d), groove (g), iliac pedicel (ipd), ischiac pedicel (isd), obturator foramen (obf), pubic plate (pp), pubic apron (pua), pubic foramen (puf), ridge (r).

The pubis is an elongate bone composed of a triangular proximal acetabular region, a long shaft tapering medioventrally into a thin apron of bone, and a distal expansion (Figs 32E–32H and 32M–32P and 33). Proximally, the iliac and ischiadic pedicels are subequal in length. The iliac pedicel is anteroposteriorly thicker and is reniform in outline, forming a concave margin (Fig 33D and 33H). The medial margin of the iliac pedicel and lateral margin of the ischiadic pedicel share a sharp transverse ridge that distinctly connects those two areas. The ischiadic pedicel is subtriangular and thins as it proceeds distally, eventually transitioning into the pubic plate and pubic apron (Fig 33A and 33E). The ischiadic pedicel is curved into an anteriorly-facing concave surface. The front edge of the iliac pedicel almost forms a right angle between the proximal surface of the pedicel and the lateral surface of the main body of the pubis. The lateral surface of the iliac pedicel is convex anteriorly before transitioning to concave posteriorly. That anterior concavity extends posteriorly into the semicircular bowl-like depression of the pubic plate and is bounded proximally by the two pedicels (Fig 33). The curvature of the proximal lateral margin of the pubis is found in sauropodomorphs and sauropods, but not in dinosauriforms and early dinosaurs, which have a straight margin [75].

In *Sarahsaurus aurifontanalis*, the pubic plate surrounds the obturator foramen, which is subcircular and forms the exit of a subcylindrical groove extending up the anterolateral surface of the iliac pedicel (Fig 33D and 33H). A subelliptical “pubic foramen” is found posteroventral to the obturator foramen (e.g., [25] p. 1049]. The pubic foramen is unique to *Sarahsaurus aurifontanalis* among sauropodomorphs. This accessory foramen appears to be a natural feature, as it is found on both pubes of the holotype. The proximal paratype pubes are too incomplete to observe those foramina (Fig 32F and 32N). Additionally, bone growth around the foramina follows their contour, indicating that the foramina are natural openings and not artifacts of preparation or preservation. Both foramina can be seen in lateral view (Fig 33C and 33G). The rest of the bone is concave down-shaft from to the cupped pubic plate, and also twists so that anterior margin of the proximal part turns laterally as it extends distally.

The cross-section of the pubis is subelliptical and tapered at its midshaft. The pubic apron is planar lengthwise but was broken post-mortem in both specimens of *Sarahsaurus aurifontanalis* (Figs 32E–32H and 33). The pubic apron is more complete in the paratype specimen (Fig 32H and 32P). The lateral surface of the pubic shaft is smooth and featureless, unlike the posteromedial surface which contains a low, rounded ridge just beneath the pubic plate (Fig 33B and 33E). Distally, the pubis is expanded mediolaterally. In distal outline, the pubis is subtriangular and it only has as slight anteroposterior expansion at its distal end.

#### Ischium

**Fig 34:**
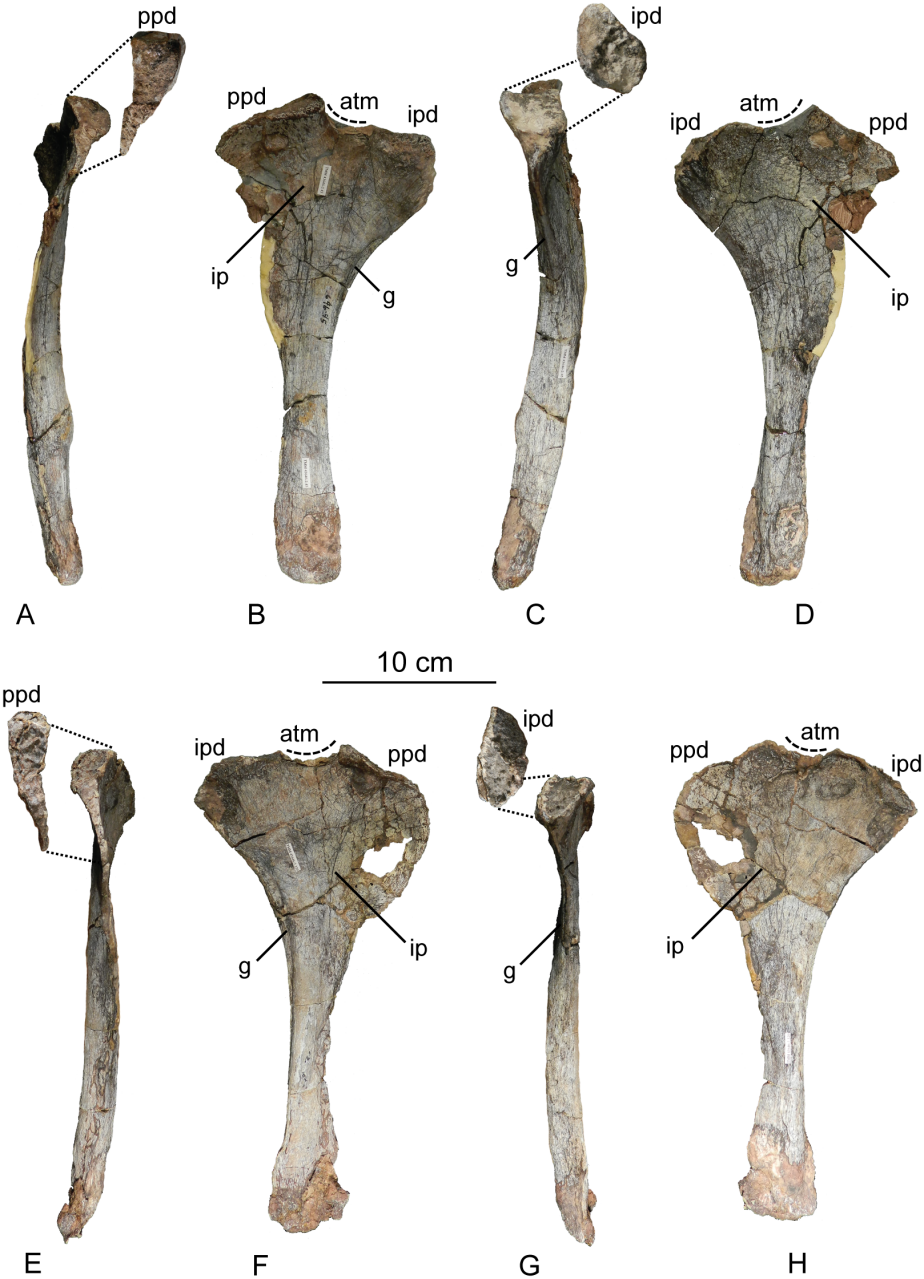
Left (A-D) and right (E-H) holotype ischia of *Sarahsaurus aurifontanalis*. A, E- anterior view; B, F- lateral view; C, G- posterior view; D, H- medial view. The proximal outlines of the pubic and iliac pedicels are provided in A and E, and C and G, respectively. Abbreviations: acetabular margin (atm), groove (g), ischiac plate (ip), iliac pedicel (ipd), pubic pedicel (ppd).

The ischium does not exhibit the same bowl-shaped proximal region as the pubis (Figs 32I–32L and 34). The pubic pedicel of the ischium has the same proximal outline as the corresponding ischiadic pedicel of the pubis (Fig 34A and 34E). The iliac pedicel of the ischium is subrhombohedral in outline and is divided into two regions by a short mediolateral ridge (Fig 34C and 34G). The posterior of those two regions is larger. In lateral view, the pubic pedicel and ischiadic plate project anterolaterally.

The pubic and iliac pedicels of the ischium are separated along the proximal margin of the ischiadic plate by a semicircular concavity that contributes to the acetabulum (Fig 34). The plate is slightly concave medially and laterally convex. In lateral view, the thin sheet of bone that forms the ischiadic plate circumscribes half of an ellipse along the posterior margin of the bone (Fig 34B and 34F). That thin sheet transitions after the midpoint of the shaft into a flatter triangular medial surface that articulates with the opposite ischium along the midline. The previous interpretation of a gap between the ischia was probably a misinterpretation of the missing portions of the ischiadic plate on the left holotype ischium [25]. Similarly, there is no notch separating the margin of the ischiadic plate from the shaft [143]. Instead, the plate and shaft transition smoothly into one another (Fig 34B and 34D). A longitudinal groove extends along the dorsolateral surface of the ischium, originating at the ischiadic plate and expanding at its distal end to transition posteriorly into the smooth dorsal surface of the bone (Fig 34B and 34C). This groove is also present in most early sauropodomorphs, but not sauropods. The ischial shaft of *Sarahsaurus aurifontanalis* is strongly triangular in cross-section owing to the smooth dorsal surface and flat medial articular surface. The distal ischium is expanded slightly anteroposteriorly and terminates in a bulbous, rounded end that is subtriangular in outline.

### Reconstruction of the pelvic girdle

**Fig 35:**
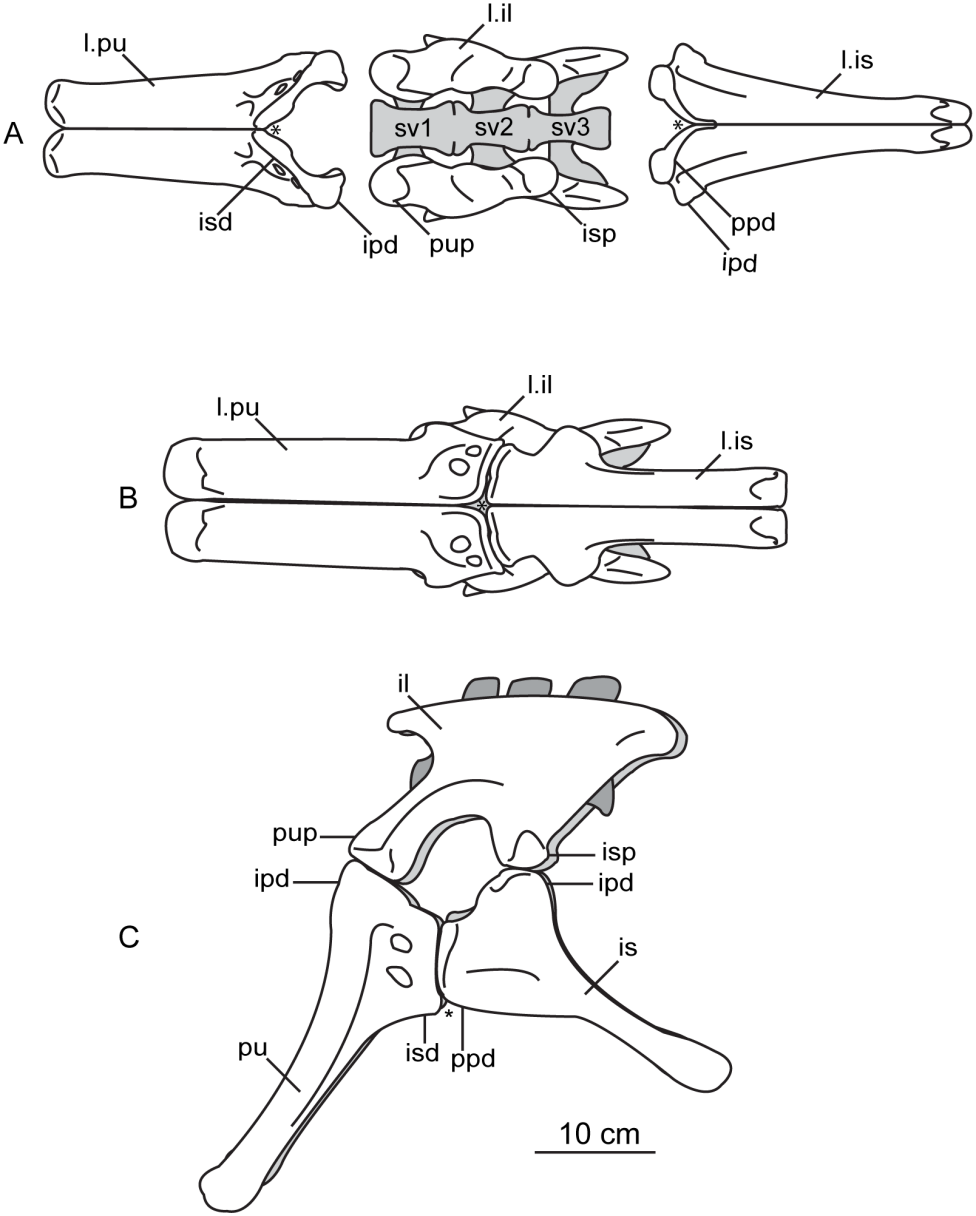
Line drawing reconstruction of pelvic girdle of *Sarahsaurus aurifontanalis* based upon the complete pelvic girdle of the holotype specimen. A- Articulated pairs of ischia, ilia, and pubes in ventral view; B- Ventral view of articulated pelvic girdle; C- Left lateral view of articulated pelvic girdle and sacral vertebrae. Asterisks in A, B, and C indicate articulation along the midline between the paired ischia and pubes. No evidence of a fontanelle is present in this region. Abbreviations: ilium (il), iliac pedicel (ipd), ischium (is), ischiac pedicel (isd), ischiac peduncle (isp), pubic pedicel (ppd), pubis (pu), pubic peduncle (pup), sacral vertebra (sv).

Many elements that make up the pelvic girdle of *Sarahsaurus aurifontanalis* are preserved and re-articulate well with one another. The most incomplete elements are the distal pubes, which do not preserve much of the aprons that would articulate with one another along the midline as in other related sauropodomorphs. However, the paired holotype pubes and ischia are complete enough that come together when articulated, and form a complete pubo-ischiadic plate that appears to be continuous and lacking a midline fontanelle (Fig 35). Because the bone is so thin in that region, the midline articulation is probably not preserved in many sauropodomorphs.

### Hindlimb

The holotype specimen of *Sarahsaurus aurifontanalis* preserves a complete, semi-articulated left hindlimb that includes every tarsal, metatarsal, and phalanx. The tibia, fibula, and pes were compressed against a series of caudal vertebrae and were not removed during preparation. Associated with the left hindlimb were the right femur, fibula, partial distal tibia, astragalus and calcaneum, metatarsals II and IV, and approximately half of the pedal phalanges. The holotype preserves an undistorted right tibia of which the proximal quarter is missing. Pedal elements from the right side are largely undistorted. The paratype specimen includes a complete right femur, left tibia and fibula, right astragalus and calcaneum, complete and disarticulated right metatarsals I-V, and numerous pedal phalanges. The femur, tibia, and fibula are described with their long axes oriented vertically. The astragalus and calcaneum are described as if in articulation with the tibia and fibula. Metatarsals and phalanges are oriented such that each plantar surface faces ventrally.

#### Femur

**Fig 36:**
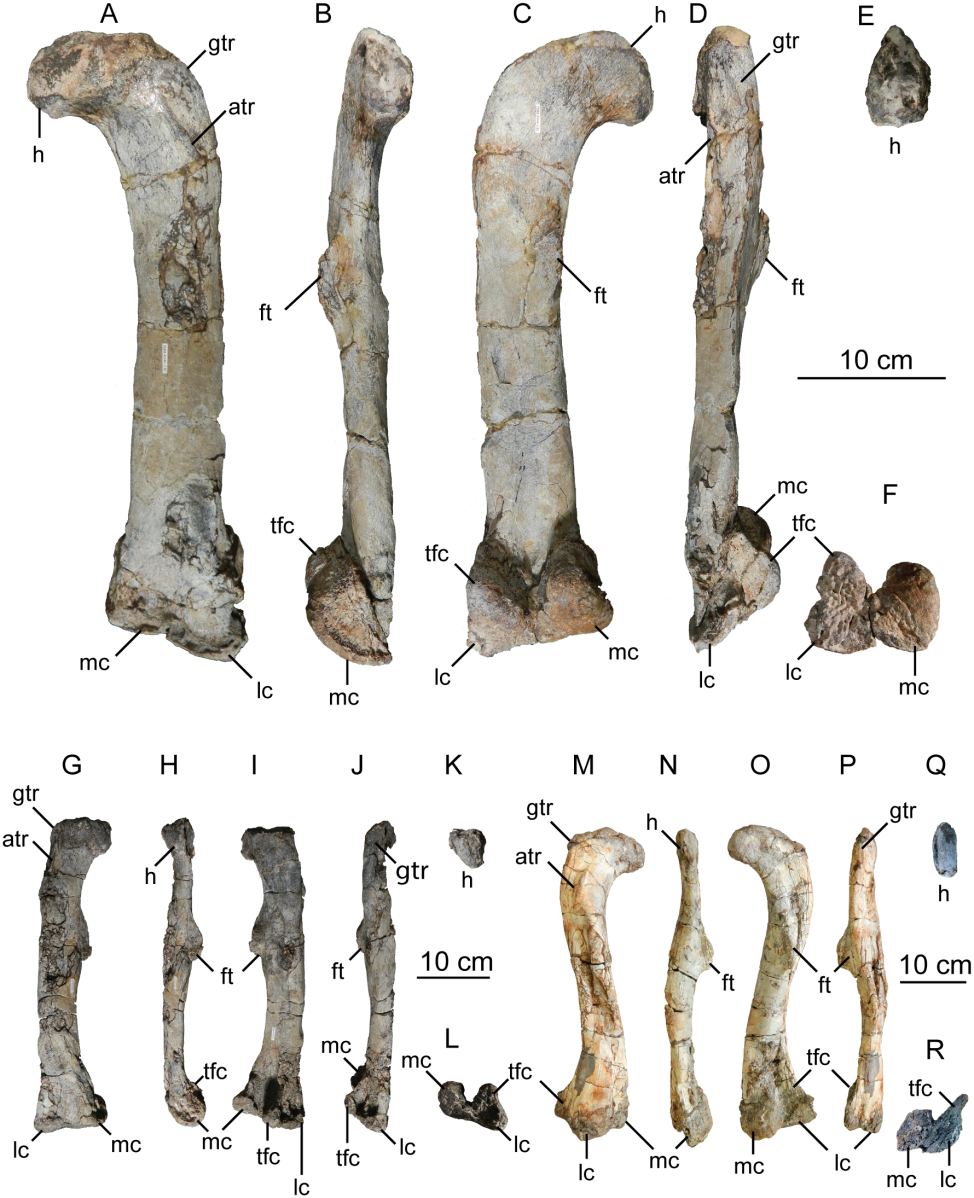
Holotype left (A-F), holotype right (G-L), and paratype right (M-R) femur of *Sarahsaurus aurifontanalis*. A,G, M- anterior view; B, H, N- medial view; C, I, O- posterior view; D, J, P- lateral view; E, K, Q- proximal view; F, L, R- distal view. Abbreviations: anterior trochanter (atr), crista tibiofibularis (ctf), fourth trochanter (ft), greater trochanter (gtr), head (h), lateral condyle (lc), medial condyle (mc), ridge (r).

Both of the holotype femora were flattened anteroposteriorly post-mortem owing to their position in the quarry, which makes it difficult to assess the degree of curvature of the femoral shaft in lateral view (Fig 36C and 36I). There is a slight sigmoidal curve in the femur; the proximal half of the femur bows posteriorly and the distal half arches anteriorly. The exact angle of total curvature is impossible to recreate from the crushed elements, but the femora are definitely not straight like those found in sauropod dinosaurs. The right paratype femur has been crushed obliquely such that the lateral curvature is manifested mostly in anterior view (Fig 36O and 36P). In anterior view, the femoral shaft is slightly curved (Fig 36A, 36G and 36M). There is a slight degree of offset between the transverse axis of the distal condyles and the longitudinal axis of the femoral head that doesn’t exceed 30° in any of the femora available for study. Both of these observations differ from the features described previously [25].

The proximal part of the femoral head is reniform (Fig 36E, 36K and 36Q). The surface of the head is round and rugose, especially in the holotype. This surface would have been covered by articular cartilage in life. Proximally, the posterior surface of the femoral head and neck are anteroposteriorly flat. All three femora are crushed inwards halfway down the shaft on the anterior surface. Because it is in multiple pieces, the paratype femur shows that this crushing is the result of the hollow medullary cavity being pushed inward post-mortem. The femoral shaft is subelliptical in cross-section above and below the crushed midsection.

The dorsolateral trochanter (= ‘greater trochanter’) forms a thin, low ridge projecting laterally from the proximal anterolateral surface of the shaft adjacent to the femoral head (Fig 36D). That ridge follows the subtle curvature of the lateral margin of the top of the femur and terminates distally before the transition into the femoral shaft. As the dorsolateral trochanter diminishes distolaterally, the anterior trochanter begins to rise on the anterior surface of the proximal part of the femoral shaft. The anterior trochanter is a tall, elongate rugosity that is undistorted in the paratype but pinched mediolaterally and crushed against the anterior surface of the holotype specimen (Fig 36A and 36M). The anterior trochanter is oriented on the anterior surface of the femur such that the distal end lies close to the anterolateral margin of the bone and the proximal end is just lateral to the midpoint of the anterior surface. That trochanter bows slightly proximolaterally, and becomes thicker distally. The anterior trochanter does not form a transverse shelf unlike that of *Pampadromaeus barberenai* [72], *Buriolestes schultzi* [71, 144], and some early theropods [56, 58, 145]. The anterior trochanter is not visible in posterior view.

The fourth trochanter is a ridge that lies just proximal to the midpoint of the femoral shaft, situated close to the medial margin on the posterior surface of the femur (Fig 36C and 36O). It is roughly parallel to the long axis of the femur, unlike the obliquely-oriented fourth trochanter found on the femora of *Eucnemosaurus entaxonis* [37] and *Morektonus thabanensis* [35]. The fourth trochanter of *Sarahsaurus aurifontanalis* forms a slanted trapezoid in lateral view, with distinct proximal and distal slopes and a flat apex that is subparallel to the femoral shaft. The proximal slope is longer and low, rising at 20° from the proximal femur, approximately level with the distal termination of the anterior trochanter. The distal slope of the fourth trochanter is shorter and steeper, making a 45° angle with the shaft. The asymmetrical lateral profile of that structure is plesiomorphic for sauropodomorphs, but it becomes symmetrical in sauropods. In *Sarahsaurus aurifontanalis*, the crest of the trochanter is slightly concave medially, roughly paralleling the longitudinal contour of the femoral shaft.

The lateral and medial margins of the femur expand slightly distal to the fourth trochanter until they flare out at the distal condyles. The mediolateral width exceeds the anteroposterior width. Medial and lateral condyles are apparent in distal view, but those features are differentially crushed in the three femora (Fig 36F, 36L and 36R). Posteriorly, a slanting ridge extends along the distal quarter of the length of the femur, towards the condyles.

The medial condyle is subrectangular and is more rounded on the posterior corners (Fig 36F and 36L). Viewed distally, the anteromedial corner approximates a 90° angle. The round articular surface of the medial condyle slopes significantly towards the posterolateral corner in distal view. A very shallow depression is found on the anterior surface of the femur in between the condyles, which is found in most early sauropodomorphs (except *Saturnalia tupiniquim* [146, 147], and *Thecodontosaurus antiquus* [19, 100, 101]). A triangular concavity separates the medial condyle from the lateral condyle plus the crista tibiofibularis in posterior view. The lateral condyle and crista tibiofibularis form a larger complex that is roughly triradiate in distal outline (Fig 36F and 36L). The posteromedial arm of that triangle is composed of the lateral condyle and is inclined facing posteriorly and only slightly laterally. The crista tibiofibularis forms the posterolateral corner of the triangle. Viewed distally, the lateral condyle faces laterally and very slightly anteriorly and projects slightly more distally than the rest of the distal condyle complex in posterior view. The crista tibiofibularis and lateral condyle are demarcated by a shallow depression on the distal surface of the femur, which can only be observed on the holotype right femur. The medial condyle is smaller than the entire region incorporated by the lateral condyle and the crista tibiofibularis. In sauropods, the medial condyle is larger than the lateral condyle plus the crista tibiofibularis. The crista tibiofibularis is greatly diminished proximally in the holotype femora, but is more pronounced (and crushed anterolaterally) in the right paratype femur (Fig 36M and 36O). In *Sarahsaurus aurifontanalis*, the entire articular surface is rugose and contains areas with rather large pits that would be covered by articular cartilage (Fig 36F).

#### Tibia

**Fig 37:**
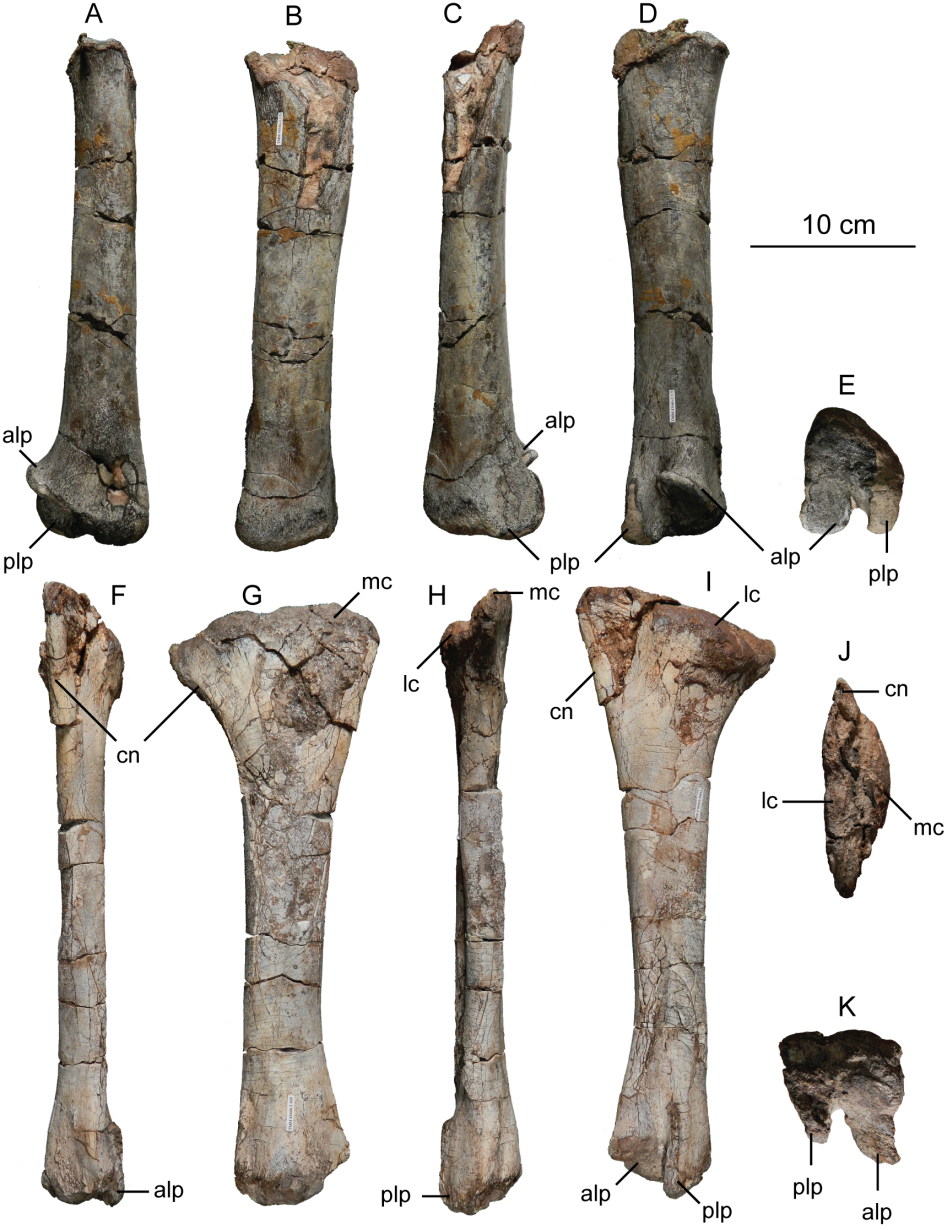
Holotype right (A-E) and paratype left (F-K) tibia of *Sarahsaurus aurifontanalis*. A,F- anterior view; B, G- medial view; C, H- posterior view; D, I- lateral view; E, K- distal view; J- proximal view. Abbreviations: anterolateral process (alp), cnemial crest (cn), hole (h), lateral condyle (lc), medial condyle (mc), posterolateral process (plp), rugosity (ru), triangular concavity (tc).

The only bone that preserves the entire proximal head of the tibia is the left paratype tibia, although that region has been compressed both mediolaterally and anteroposteriorly (Fig 37). The total length of the tibia is 61% of that of the femur. Proximally, the anteroposterior width is greater than the mediolateral width. The proximal outline exhibits medial and lateral articular condyles that are separated by a shallow, broad groove extending anteriorly from the posterior margin of the proximal articular surfaces to the beginning of the cnemial crest (Fig 37J). The proximal medial surface displays short striations extending parallel to the long axis of the bone. The medial surface of the bone curves anterolaterally as its proximalb part, merging seamlessly with the medial surface of the cnemial crest.

Viewed laterally, the cnemial crest is triangular (Fig 37I). This crest emerges from the bone pointing anteriorly and slightly laterally. The anterolateral orientation of the cnemial crest is exaggerated in the distorted paratype tibia. However, the distal-most origination of the crest is observable in proximal view of the incomplete holotype right tibia, in which the crest projects more anteriorly than laterally. The anterolateral orientation of the cnemial crest in *Sarahsaurus aurifontanalis* is plesiomorphic for dinosaurs, and the crest only projects strictly laterally within Sauropodomorpha among the sauropods. Similarly, the tallest point of the cnemial crest of *Sarahsaurus aurifontanalis* lies near the proximal end of the tibia and not halfway down the bone as in sauropods (Fig 37G and 37I).

Distally, the tibia of *Sarahsaurus aurifontanalis* becomes subcircular towards the distal end. The distal half of the bone contains a low but distinct anterolateral ridge. The ridge recedes distally before reaching the distal anterolateral process (Fig 37D and 37I). Viewed laterally, the distal margin of the anterolateral process ends abruptly in a smooth, sloping surface that faces posteriorly and slightly medially (Fig 37C, 37D and 37E). That surface corresponds to the flat, sloping surface of the ascending process of the astragalus. A triangular cavity receives more of the ventral portion of the ascending process of the astragalus. The posterolateral process extends laterally to obscure the distal anterolateral process and the concavity for the astragalus in posterior view (Fig 37C and 37H). The posterolateral process does not extend laterally and contact the fibula, similar to that of *Herrerasaurus ischigualastensis* [93–96], *Staurikosaurus pricei* [147–149], and most sauropodomorphs.

In distal view, the outline of the tibia of *Sarahsaurus aurifontanalis* is similar to that of early sauropodomorphs (Fig 37E and 37K). The anteromedial corner of the distal end of the tibia forms a right angle in *Marasuchus lilloensis* [119], *Herrerasaurus ischigualastensis* [93–96], *Staurikosaurus pricei* [147–149], *Eoraptor lunensis* [70, 150, 151], and *Saturnalia tupiniquim* [97, 146], but most early sauropodomorphs share the acute angle found in *Sarahsaurus aurifontanalis*. The distal-most end of the holotype right tibia contains a large hole anteriorly that is not present in the paratype. It is probably the result of scavenging or some other post-mortem alteration rather than a natural foramen (Fig 37A). However, an elliptical area of rough, raised bone lies just proximal to that hole on the distal anterior surface and is present in both individuals.

#### Fibula

**Fig 38:**
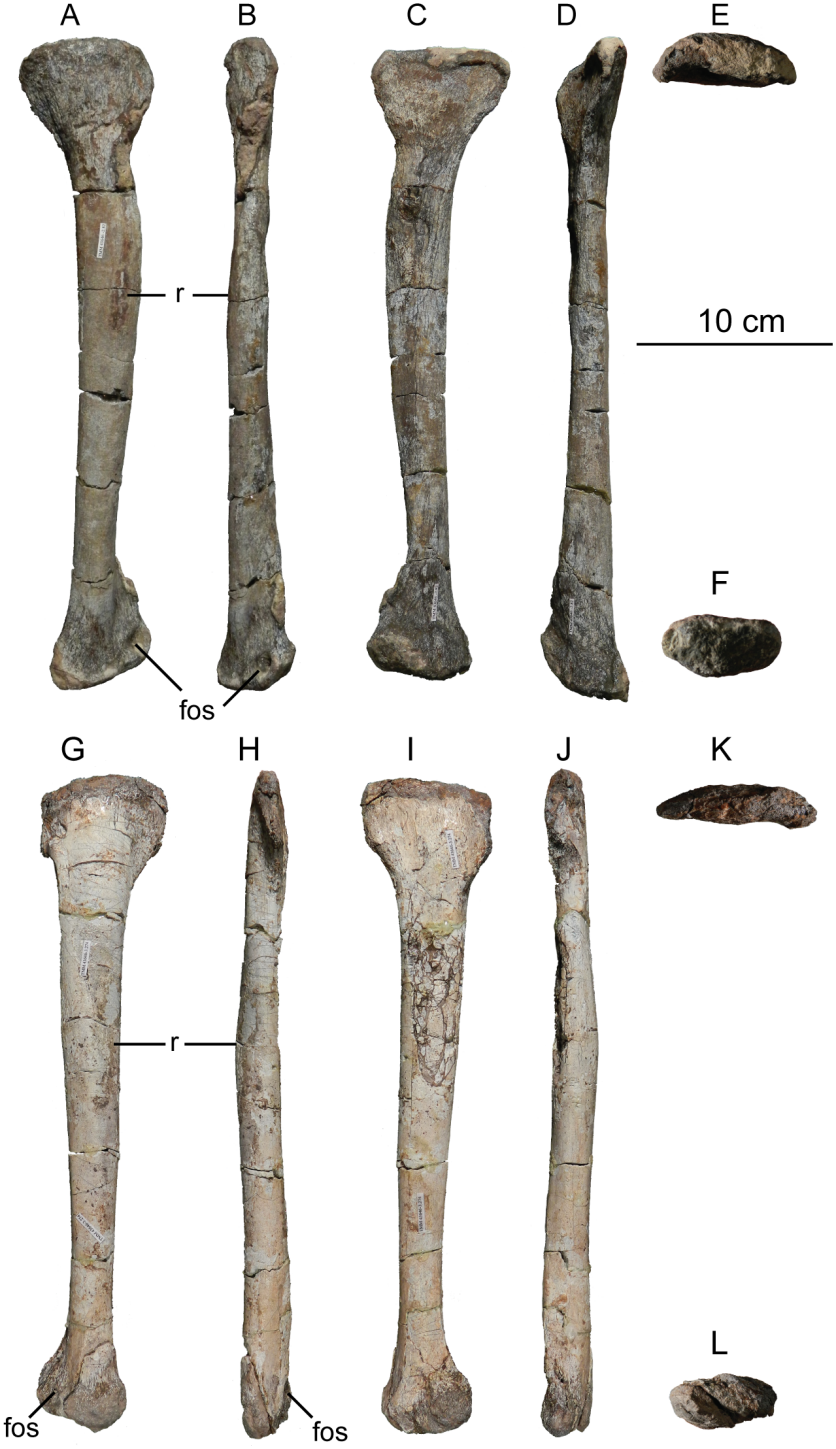
Holotype right (A-F) and paratype left (G-L) fibula of *Sarahsaurus aurifontanalis*. A,G- lateral view; B, H- anterior view; C, I- medial view; D, J- posterior view; E, K- proximal view; F, L- distal view. Abbreviations: foramen (f), fossa (fos), ridge (r).

The fibula is long and thin, bowing slightly laterally in anterior view and anteriorly in lateral view (Fig 38). The proximal end is expanded anteroposteriorly more than the distal end, and both are connected by a subcircular shaft that thins distally. The proximal and distal long axes are twisted by approximately 35°. The proximal expansion is flat on its medial surface and marked with small longitudinal striations. The articular surface for the tibia is slightly convex and smooth. The proximal lateral surface is convex before tapering to follow the fibular shaft. One-third down the anterolateral length of the fibula is a low longitudinal ridge (= fibular trochanter) onto which the muscle *iliofibularis* inserted (Fig 38A, 38B, 38G and 38H) [137].

A very small foramen is present on the midpoint of the shaft of the fibula. The distal end of the fibula is inclined posteromedially. The incline contains a small keel along its anterior corner. The holotype right fibula preserves a small, circular fossa at that keel which resembles that found in *Massospondylus carinatus* [14] (Fig 38A and 38B). The paratype left fibula does not preserve that area very well. Because the tibia is poorly preserved, it is impossible to determine the exact articular position of the fibula or its relationship to the astragalus. More than likely, the calcaneum was located just beneath the fibula, but it was not found in articulation.

#### Astragalus

**Fig 39:**
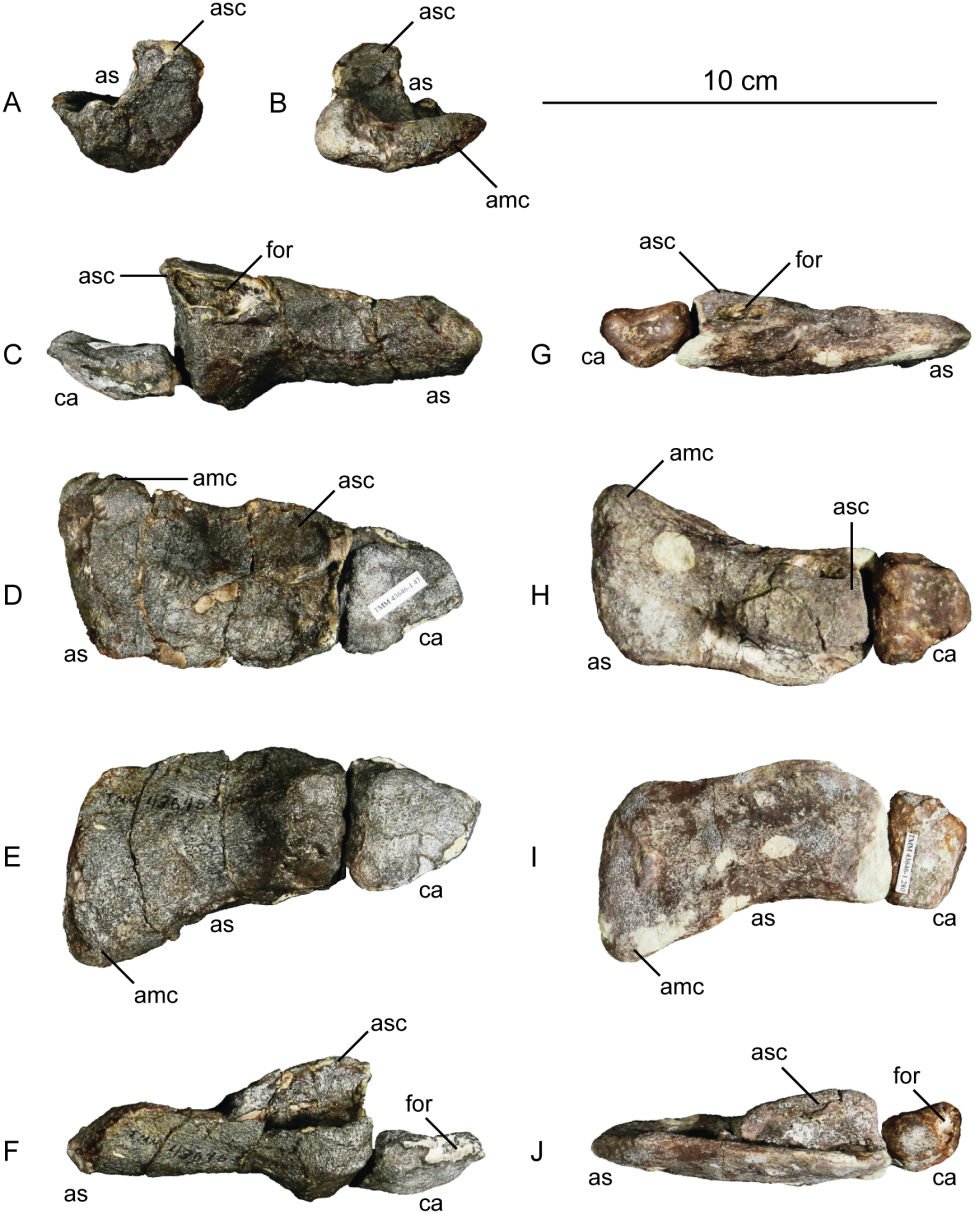
Holotype right (A-F) and paratype right (G-J) astragalus and calcaneum of *Sarahsaurus aurifontanalis*. A- lateral view (astragalus only); B- medial view (astragalus only); C, G- anterior view; D, H- proxima view l; E, I- distal view; F, J- posterior view. Abbreviations: astragalus (as), anteromedial corner (amc), ascending process (asc), calcaneum (ca), foramen (for).

The basic shape of the astragalus is plesiomorphic for early sauropodomorphs (Fig 39). In proximal outline, the astragalus is subtrapezoidal, in which the lateral margin is shorter than the medial margin. Both of those margins are straight and slanted posterolaterally. The anterior margin is wider than the posterior margin. The front of the bone is fairly straight laterally but is convex medially and the back is straight and inclined anterolaterally. The anteromedial corner is the most acute corner of the bone in proximal view. Most of the distal surface is broadly convex in medial view, resembling half of a cylinder (Fig 39E and 39I). However, the lateral third of the distal surface is more pronounced and is strongly convex, forming a low subrectangular process directly underneath the ascending process of the astragalus.

Proximally, the medial edge is proximodistally thick, especially at the anteromedial corner (Fig 39B). A pyramidal process is found on the dorsal surface of the anteromedial corner here a pyramidal process rises proximally, but it is not nearly as high as that of *Adeopapposaurus mognai* [77]. Laterally, the articular surface for the calcaneum is slightly convex. In lateral outline, the astragalus is roughly triangular, and the ascending process forms a tall apex (Fig 39A). A sharp ridge extends along the proximolateral margin of the astragalus from the ascending process to the posterolateral corner of the element.

The ascending process of the astragalus is subrectangular in proximal outline, but is more rounded laterally. The subelliptical articular surface is flat and inclined anteromedially, slotting onto the complementary surface of the tibia (Fig 39D and 39H). The base of the ascending process of the astragalus is slightly concave anteriorly and flares posterolaterally onto the main body of the astragalus near the middle of the bone. Because of this, the ascending process takes up the anterolateral quarter of the astragalus in proximal view. Plesiomorphic for early sauropodomorphs, the posterior extent of the ascending process in *Sarahsaurus aurifontanalis* does not get anywhere near the posterior margin of the main body of the astragalus as it does in sauropods.

The anteromedial margin of the articular facet of the ascending process is a sharp lip, underneath which is a concave area that wraps around to the posterior margin of the bone. A transverse groove lies behind the lip that accepts the distal edge of the posterolateral process of the tibia. Underneath the anterior lip of the articular facet is a subtrapezoidal fossa that is inclined proximolaterally. A foramen penetrates the mediodistal corner of that fossa (Fig 39C and 39G).

#### Calcaneum

The subtriangular calcaneum articulates tightly with the lateral face of the astragalus in a simple curved joint (Fig 39). The articulation with the astragalus is facilitated by a slight concavity on the calcaneum that slots over a corresponding convexity on the astragalus. The anteromedial corner of the calcaneum is somewhat taller proximally than the rest of the bone, which maintains a slight concavity for much of the proximal surface that articulates with the distal fibula. The anterolateral margin of the calcaneum houses a depression on the anterior surface. Similarly, a groove extends along the posterolateral margin, possibly perforated by a few small foramina (Fig 39F and 39J). The calcaneum is proximodistally thickenened in the middle of the bone, forming a thick middle section that slopes towards all three corners of the bone.

#### Distal tarsals

**Fig 40:**
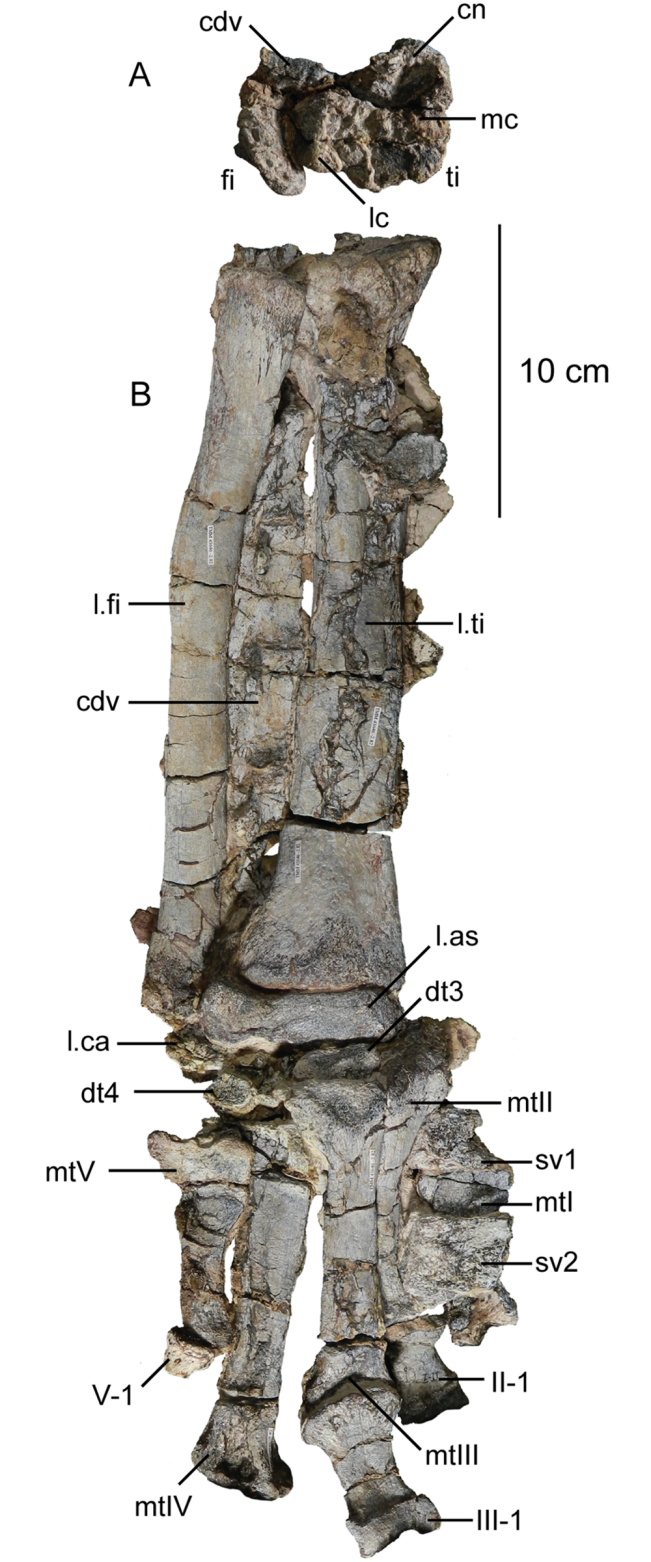
Articulated holotype left tibia, fibula, tarsus, and pes of *Sarahsaurus aurifontanalis*. A- proximal view of tibia and fibula; B- posterior view. Anterior view of the tibia, fibula, and astragalus is obstructed by articulated distal caudal vertebrae (see Fig 19B). Abbreviations: left astragalus (as), left calcaneum (ca), caudal vertebra (cdv), cnemial crest (cn), distal tarsal (dt), left fibula (fi), lateral condyle (lc), medial condyle (mc), metatarsal (mt), sacral vertebra (sv), left tibia (ti), digits one through five (I-V).

The only two identifiable distal tarsals known for *Sarahsaurus aurifontanalis* are preserved in articulation with the left tibia and fibula, proximal tarsals, and pes of the holotype individual (Fig 40B). Those two elements are most closely associated with metatarsals III and IV, and probably represent distal tarsals 3 and 4, which are present plesiomorphically in dinosaurs (Fig 40B) [75]. Distal tarsal 3 does not appear to have been displaced, but distal tarsal 4 was rotated slightly to the posterolateral surface of the pes. Those elements are obstructed by the proximal tarsals, metatarsals, and vertebrae articulated in that region, but distal tarsal 3 can be seen in anterior view and distal tarsal 4 can be seen in posterior view.

Distal tarsal 3 is subrhombohedral in anterior outline. Its medial margin is wider than the pointed lateral margin. The medial margin is convex. The proximal margin is straight for most of its length and curves upwards at the proximomedial corner. The proximal and medial surfaces are flat and meet the anterior surface almost at a right angle, forming a hard edge. A single subelliptical foramen lies on the extreme distal surface, lying on the medial third of the straight margin that abuts metatarsal III (Fig 40B). The entire distal margin of the bone is in close articulation with metatarsal III. The bulging medial margin articulates with the lateral concavity of metatarsal II, which was displaced proximally post-mortem. Distal tarsal 3 articulates closely with the distal surface of the astragalus.

The fourth distal tarsal is subrectangular in posterior view (Fig 40B). Most of its margins are subequal in length, except for the margin articulating with metatarsals IV and V. That margin is ‘stepped’ such that the distolateral half is thinner than the proximolateral half. The bone thickens distally. A small distal concavity can be seen in posterior view, and houses a small foramen that lies closer to the medial margin of the bone. Proximally, distal tarsal 4 is associated, but not closely articulated with the calcaneum.

#### Metatarsus

**Fig 41:**
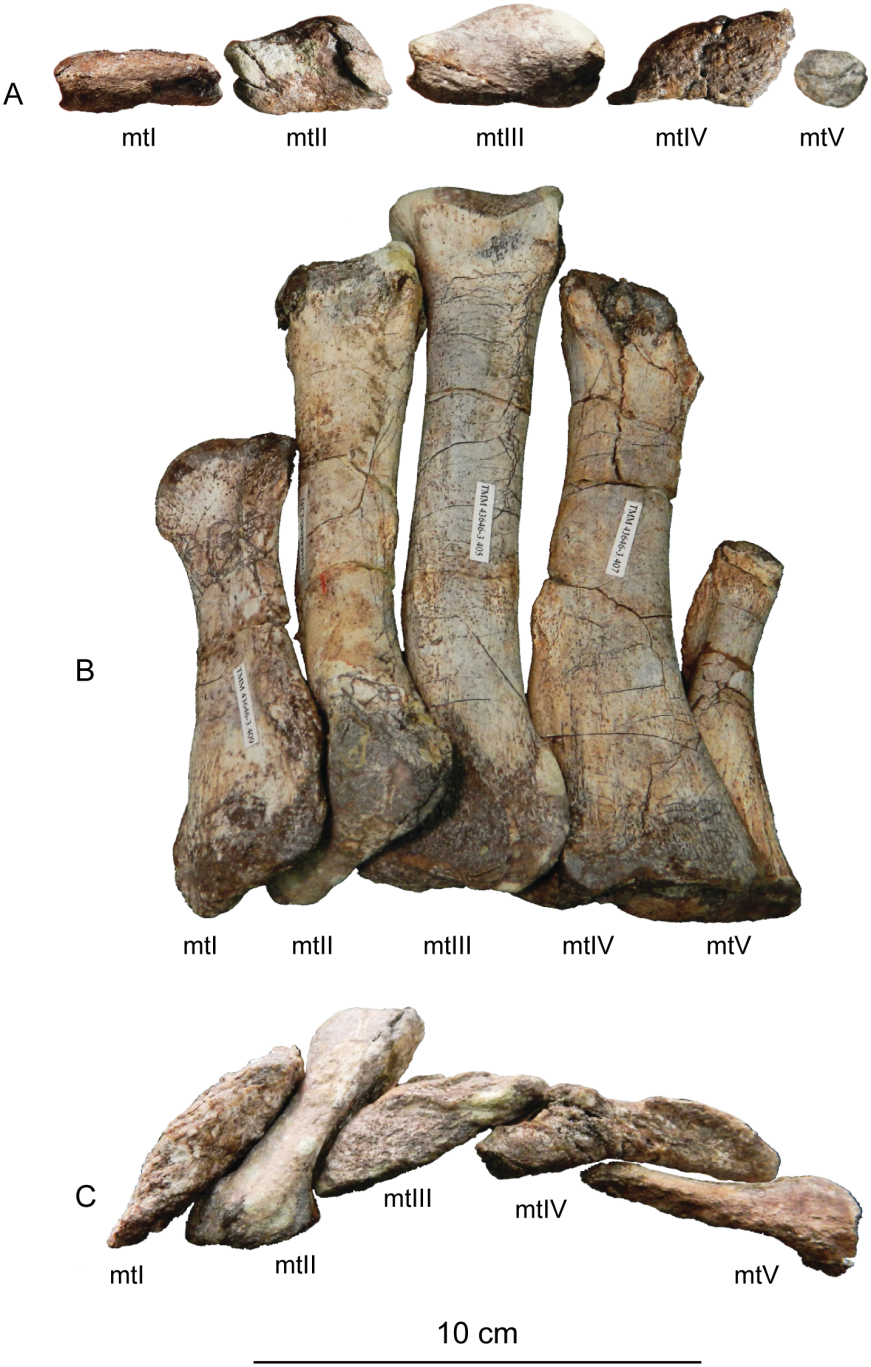
Articulated paratype right metacarpus of *Sarahsaurus aurifontanalis*. A- distal view; B- dorsal view; C- proximal view. Abbreviations: metatarsal (mt).

The metatarsus is arranged in a semi-columnar fashion in which the proximal surfaces of the metatarsals are aligned, but the distal ends are only slightly spread (Figs 40B and 41). Viewed proximally, the metatarsals form a gentle arch that bows outward (Fig 41C). The ventrolateral surface of each bone overlaps the dorsomedial surface of its lateral neighbor. The third metatarsal is the longest, followed by the fourth, second, first, and fifth. Most of the elements known from the metatarsus are slightly crushed.

**Fig 42:**
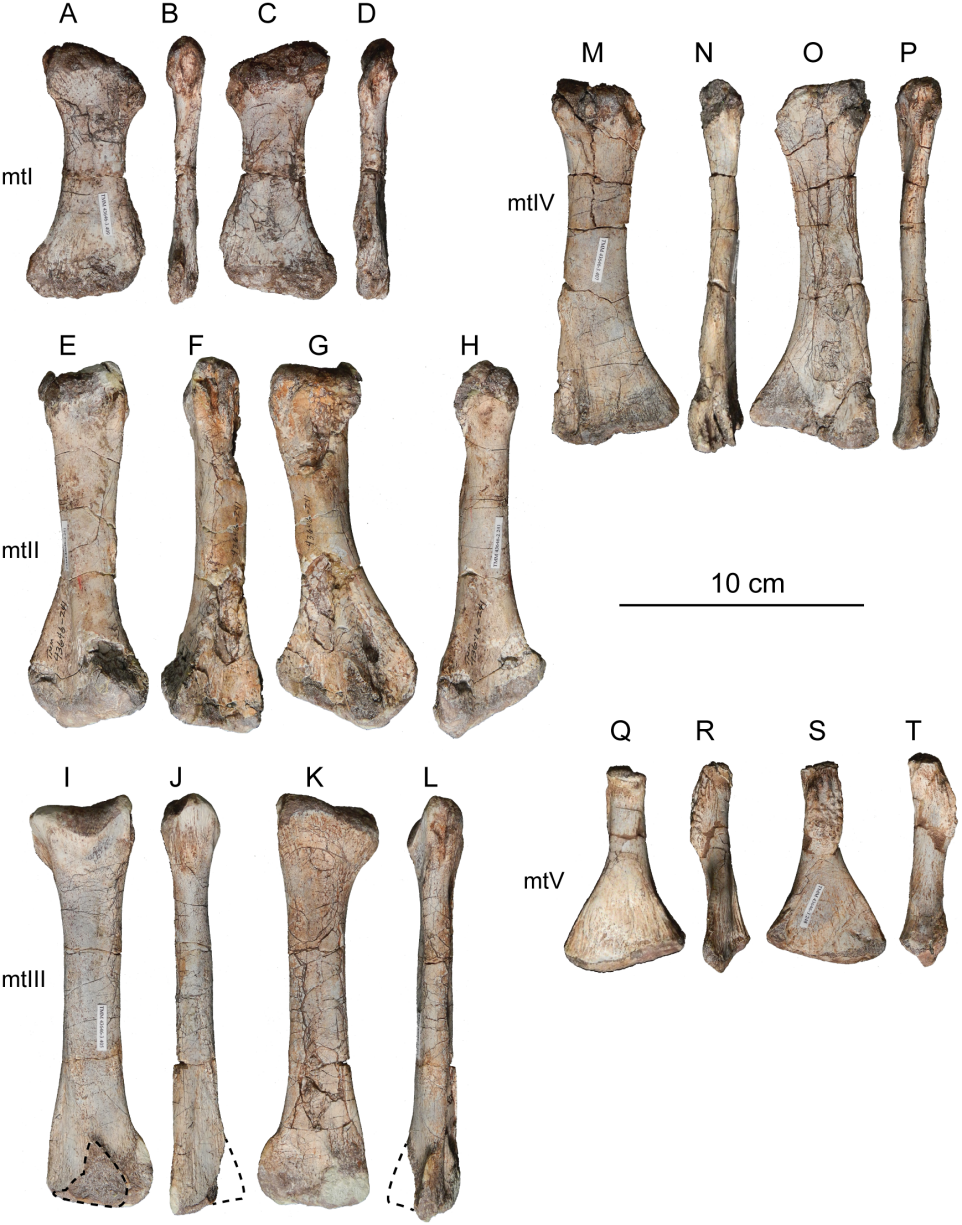
Paratype right metatarsals of *Sarahsaurus aurifontanalis*. A, E, I, M, Q- dorsal view; B, F, J, N, R- medial view; C, G, K, O, S- ventral view; D, H, L, P, T- lateral view. Dashed lines indicate missing areas. Abbreviations: metatarsal (mt), ridge (r).

Metatarsal I is a flat bone with expanded proximal and distal ends, and is mediolaterally thinnest just before the distal expansion (Fig 42A–42D). The maximum proximal width is 55% of the proximodistal length of the bone. In proximal outline, the bone is subelliptical, but it bulges more ventrally and thins to a pinched point laterally. This point corresponds to a broad, but short process on the proximolateral corner projecting just past the lateral extent of the distal end. Raised striations extend perpendicular to the articular surface on the proximal dorsal and ventral surfaces. Ventrally, a short ridge extends along the medial edge of the proximal quarter of the first metatarsal. The distal end expands mediolaterally and dorsoventrally. The lateral distal condyle is larger than the medial condyle, and projects further distally. The dorsal margin of the distal end is convex, but the two distal condyles are separated by a shallow groove on the ventral margin. Both condyles house oblong collateral ligament fossae. The proximal and distal transverse axes of metatarsal I are not twisted relative to one another.

**Fig 43:**
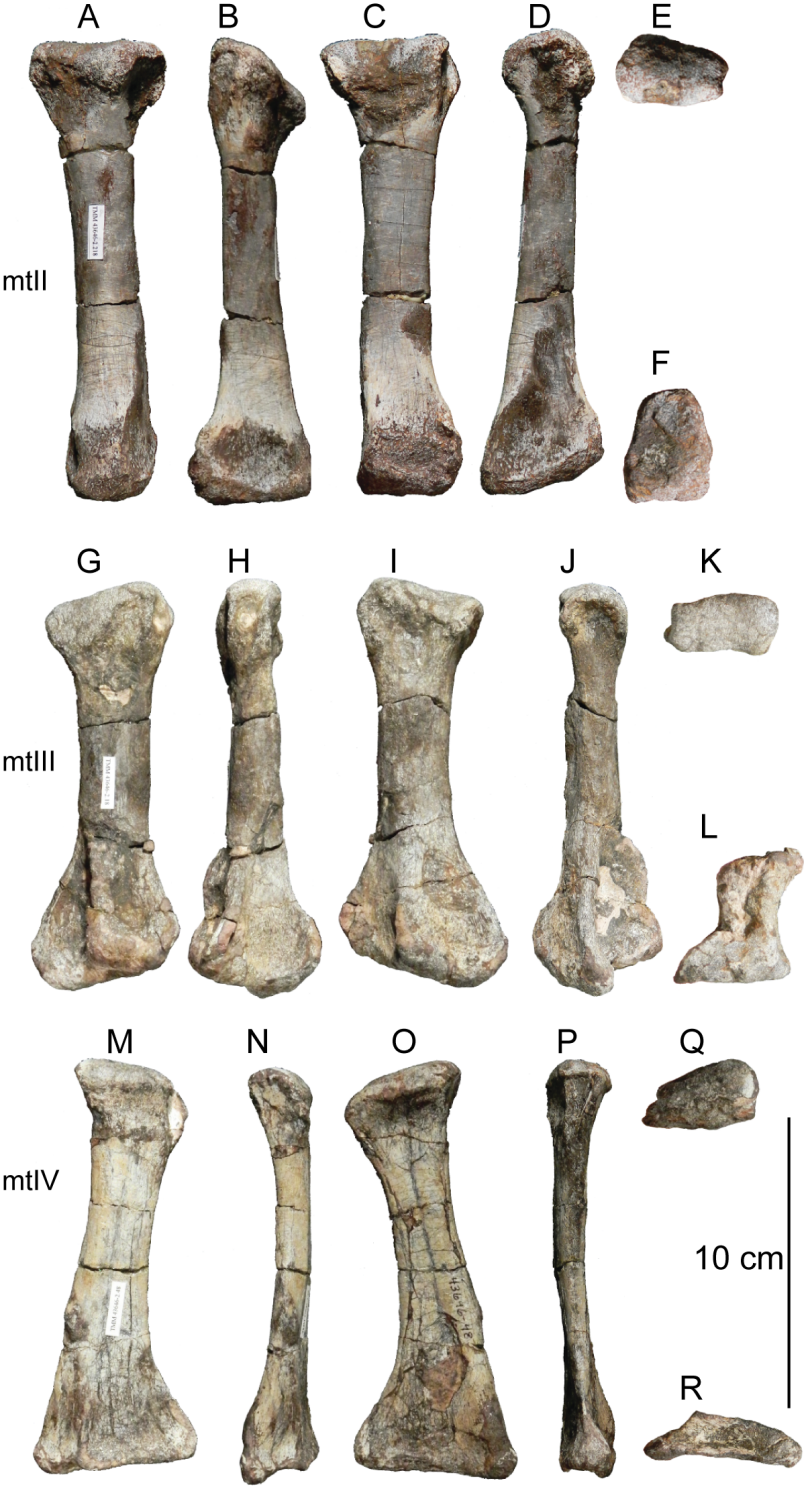
Holotype right metatarsals of *Sarahsaurus aurifontanalis*. A, G, M- dorsal view; B, H, N- medial view; C, I, O- ventral view; D, J, P- lateral view; E, K, Q- distal view; F, L, R- proximal view.

The proximal mediolateral width of metatarsal II exceeds that of metatarsal I (Figs 42E–42H and 43A–43F). The proximal end of the second metatarsal is slightly less than two times the maximum width of the distal end. The long axes of each of these ends are twisted 33° from one another. Proximally, the outline of metatarsal II is hourglass-shaped. The width of the ventral margin of the proximal outline is slightly larger than the width of the dorsal margin. The proximal articular surface for metatarsal III is covered by longitudinal raised ridges. Sharp-rimmed subelliptical fossae can be seen on the proximal dorsal and ventral surfaces of the metatarsal II, but those fossae are artifacts of crushing. Viewed dorsally, the ventromedial proximal corner is larger and more rounded than the dorsolateral corner.

Unlike metatarsal I, the thinnest portion of the shaft of metatarsal II is at its midsection. A triangular tuberosity lies on the lateral edge of the bone approximately halfway down its length. After that point, the bone expands mediolaterally to form the distal end. The medial distal condyle is larger and more pronounced than the lateral condyle. However, the lateral ligament fossa is much more shallow and round than the flattened, deep medial fossae.

Metatarsal III is the longest element in the pes, and is greater than 40% of the length of the tibia (Figs 42I–42L and 43G–43L). This bone is straight, and the mediolaterally-expanded proximal and distal ends are twisted 25° from one another. The proximal outline is subtriangular, with the ventral margin longer than the two other margins. Ventrally, the bone is flat. However, the apex of the proximal triangular outline forms a ridge that extends medially from the transverse midpoint of the proximal articular surface to the longitudinal midpoint of the shaft of the bone. The ridge also contains two smaller parallel bumps that are associated with the lateral triangular bump of metatarsal II when in articulation. More longitudinal ridges extend parallel to the long axis of the bone along the outer surface of the proximal part of the bone. Proximally, the shaft is subtriangular in cross-section, but it transitions into a subelliptical shape moving distally. The distal outline is also subtriangular due to the distal end expanding more dorsomedially. Similar to the second metatarsal, the medial distal condyle is larger and projects farther distally than the lateral condyle, but the lateral collateral ligament fossa is more flattened and deeper.

The fourth metatarsal is flat (Figs 42M–42P and 43M–43R). In dorsal view, the entire bone bows medially, but is more concave along its lateral surface. The element has a sublenticular proximal outline and is much wider mediolaterally than dorsoventrally. Proximally, the ventral margin is concave. Laterally, the bone pinches to a point. A dorsal ridge of bone is seen along the medial third of the bone. Medial to that ridge, there is a triangular fossa that articulates with metatarsal III. Proximally, the lateral two-thirds of the dorsal margin is convex, but flattens more distally along that surface. A pronounced tuberosity is present along the medial edge of the dorsal surface of metatarsal IV. The transverse width of the shaft is the thinnest at the midpoint of the bone. At the distal expansion, the lateral and medial distal condyles are pronounced. The medial condyle is the larger of the two, and both house collateral ligament fossae. The lateral edge of the shaft is rugose just proximal to the lateral distal condyle. In dorsal view, the lateral ligament fossa can be seen, but the medial fossa is obscured by a medial expansion of the distal end.

The paddle shape of metatarsal V is plesiomorphic for early sauropodomorphs (Fig 42Q–42T). The proximal half of this bone is subtriangular and covered in longitudinal raised ridges. The bone is thin medially but thickens to form a flat lateral surface. The proximal half is convex medially and concave laterally, and its ventral surface is entirely concave. The distal half of metatarsal V is subcylindrical and terminates in a subcircular outline. The ventral surface of this cylinder is rugose and expanded and almost makes a flat surface on the most distal ventral margin. Viewed medially, the dorsal margin of metatarsal V is concave. Digit V was probably not weight-bearing in *Sarahsaurus aurifontanalis* or in other early sauropodomorph taxa like *Anchisaurus polyzelus* [7,8, 32], *Seitaad ruessi* [22], *Massospondylus carinatus* [14], *Plateosaurus engelhardti* [98, 99], *Lufengosaurus hueni* [78, 79], *Jingshanosaurus xinwaensis* [117], and *Coloradisaurus brevis* [27, 142]. These taxa also have a similar structure but were incorrectly scored in the modified Yates matrix of Rowe et al. [25, 63]. Because the metatarsus was not splayed in these dinosaurs, it is doubtful that digit V ever touched the ground [129].

#### Pedal phalanges

**Fig 44:**
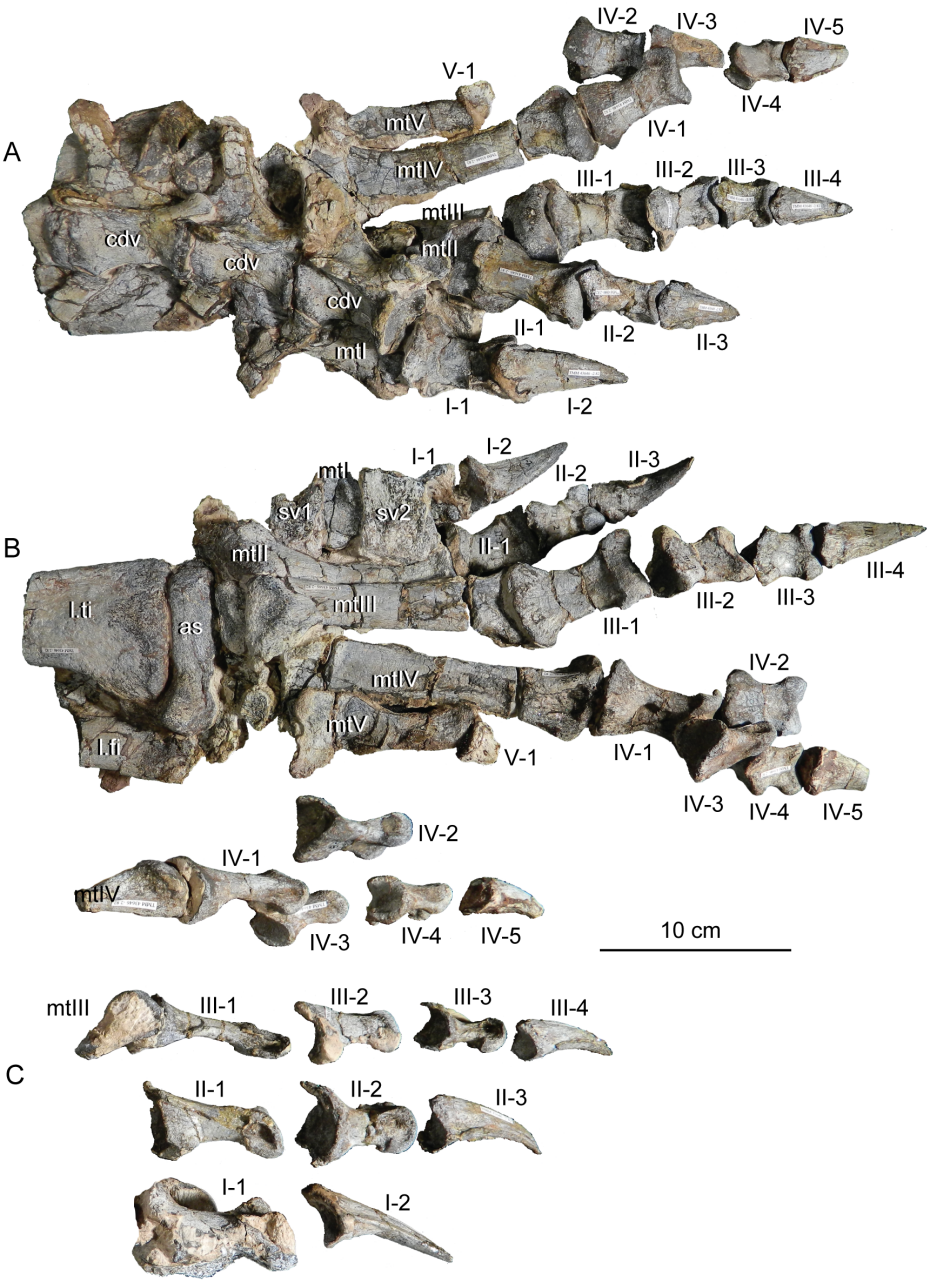
Articulated holotype left pes of *Sarahsaurus aurifontanalis*. A- dorsal view; B- ventral view; C- lateral view. Abbreviations: astragalus (as), caudal vertebra (cdv), left fibula (fi), metatarsal (mt), sacral vertebra (sv), left tibia (ti), digits one through five (I-V).

The complete left pes of the holotype specimen was mostly found in articulation and it was disarticulated during preparation (Fig 44). The complete pedal phalangeal count is 2-3-4-5-1, which is also found in many early sauropodomorphs including *Anchisaurus polyzelus* [7,8, 32], *Adeopapposaurus mognai* [77], *Plateosaurus engelhardti* [98, 99], and *Massospondylus carinatus* [14]. All non-terminal phalanges are longer than they are wide. Similarly, the proximal articulation surfaces of these phalanges are all wider than they are tall. The first four digits of the pes include at least one non-terminal phalanx and a long, pointed ungual. Phalanges from digits II and III are all wider proximally than they are deep. When articulated, the unguals were not directed medially like those of sauropods.

**Fig 45:**
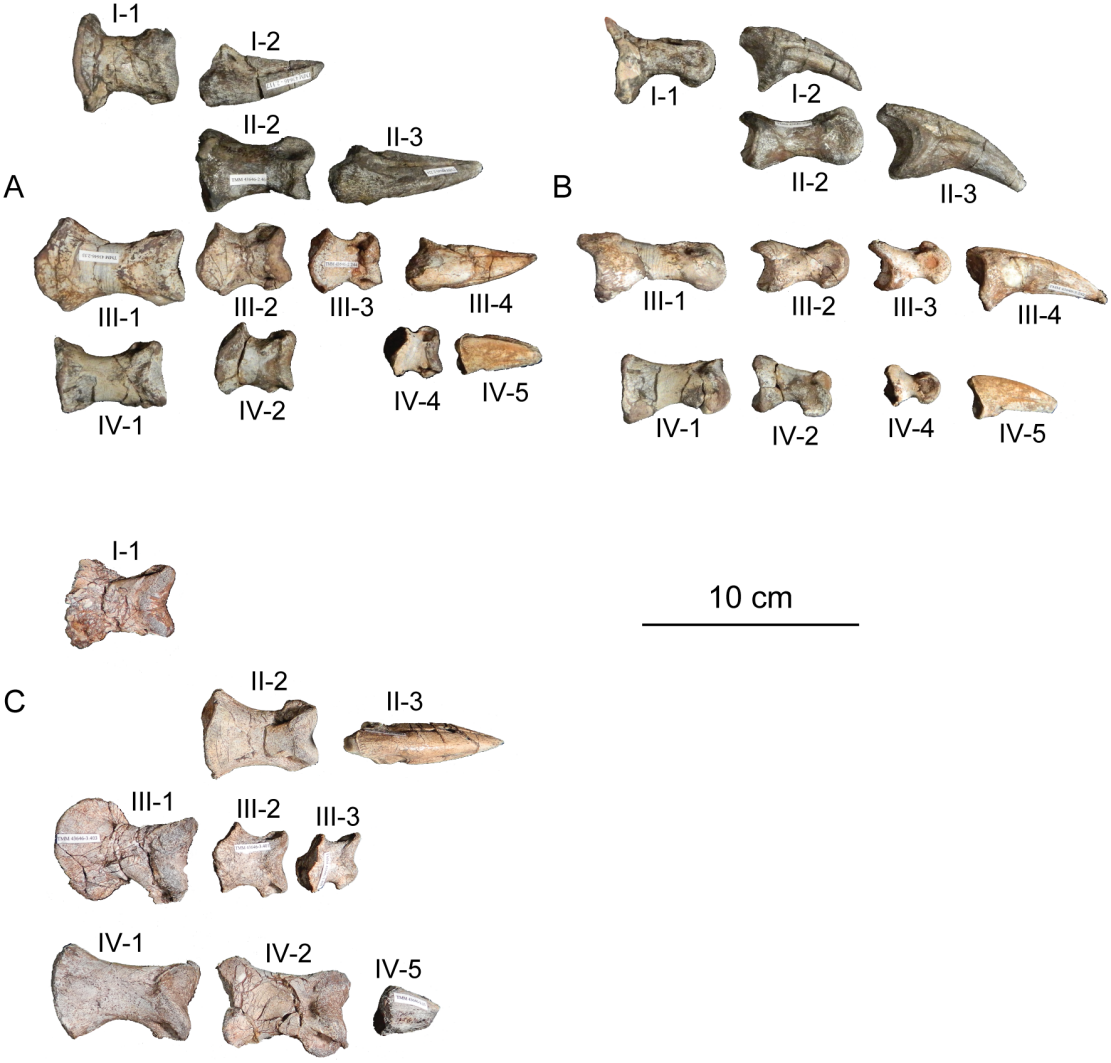
Holotype right (A and B) and paratype right (C) pedal phalanges of *Sarahsaurus aurifontanalis*. A, C- dorsal view; B- lateral view. Abbreviations: digits one through five (I-V).

Phalanx 1 of pedal digit I is slightly shorter than the curved ungual that follows it (Figs 44 and 45). The bone is semielliptical in proximal outline and thins halfway down the shaft. The mediolateral expansion of the proximal end is slightly longer than that of the distal end. The distal condyles are well-developed and are subequal in size. Those condyles are separated dorsally and ventrally by a groove. Large, well-developed collateral ligament fossae can be seen in dorsal view and are reniform in lateral and medial views.

The ungual of digit I (I-2) is long and slender, but it is not longer than the first metatarsal (Figs 44 and 45). Proximally, the outline resembles a triangle with a tall, laterally-directed dorsal process that makes the articular surface taller than wide, unlike the rounder proximal outline present in later-divererging sauropodomorphs such as *Antetonitrus ingenipes* [39] and *Mussaurus patagonicus* [41]. This surface is separated into distinct medial and lateral concavities that articulate with the distal condyles of I-1. In dorsal view, the ungual is subtriangular and long, tapering to a sharp point distally along convex side margins. The medial and lateral margins are formed into sharp edges by the concave flat ventral surface. A longitudinal groove extends parallel to the ventral margin of the ungual along both sides, and at least three small foramina can be seen in the medial of the two grooves. The medial groove terminates just before the sharp tip of the claw, but the lateral groove stops at the two-third mark along the bone.

Phalanx 1 of digit II is subequal in length to its counterpart on the first digit (Figs 44 and 45). The proximal articular surface is cup-shaped and lacks a dorsal process. The bone is thinnest at midshaft. The distal end is almost identical to that of I-1, except that the ventral margin of the distal condyles underneath the ligament fossae is slightly thicker. Phalanx II-2 is slightly shorter than the preceding phalanx. The bone is thinnest just proximal to the distal end. Additionally, the proximal surface of this bone is divided into two concave surfaces by a dorsoventral ridge extending up to a dorsal process that points laterally. In dorsal view, the distal condyles are symmetrical, and when viewed distally, the condyles lean laterally at the same angle as the proximal dorsal process. Shallower collateral ligament fossae are present, and these are rounder than those of the previous phalanx. The ungual of digit II is subequal in length to its first phalanx. It resembles ungual I-2 in overall shape, but is shorter and has a more rounded proximal outline. The dorsal proximal process also leans laterally, and the lateral proximal articular surface is smaller than the medial articular surface.

The first phalanx of digit III is only slightly shorter than ungual I-1 and is the longest non-terminal phalanx in the pes (Figs 44 and 45). The element is constricted at midshaft and strongly resembles I-1 in overall form. Unlike I-1 and II-1, the proximal and distal mediolateral expansions are subequal in width. Phalanges III-2 and III-3 strongly resemble one another in shape, but the third phalanx is sequentially shorter than the second. In III-2 and III-3, the subtriangular proximal outline is more rounded, and the dorsal process only hints towards a lateral lean. Viewed dorsally, the dorsal process is round proximally and does not entirely cover the proximal articular surface, which is divided again into subequal medial and lateral halves. These bones are thinnest below the mid-shaft, just proximal to the distal condyles. The medial condyles are slightly taller, but the lateral condyles project more laterally and distally. Those condyles are separated by a groove and house collateral ligament fossae. The distal condyles of III-2 lean laterally in distal view, but those of III-3 lean medially. The medial incline of the distal condyles of the penultimate phalanx of digit III corresponds to a medial incline of the proximal dorsal process of ungual III-4. That claw is subequal in length with phalanx III-2, shorter than the ungual of digit II, and is identical in shape to the previous unguals except that the lateral longitudinal groove reaches the same distal extent as the medial groove (Figs 44 and 45).

Phalanx IV-1 is subequal in length to I-1 (Figs 44 and 45). Its proximal articular surface is subelliptical and smooth. The shaft thins at midlength before expanding into asymmetrical distal condyles. The medial condyle is larger, but both house collateral ligament fossae, the lateral of which is very shallow. No dorsal groove separates the distal condyles, but they are separated by a small distal concavity. These condyles are directed medially. Phalanges 2, 3 and 4 of digit IV are sequentially shorter, but share the same morphology. Each has a proximal articular surface divided into lateral and medial surfaces subequal in area. The dorsal processes of them all point medially and the thinnest cross-section of the shaft can be found immediately proximal to the distal end. The distal condyles are subsymmetrical and spool-shaped and contain round collateral ligament fossae. The ungual tipping this digit is the shortest claw in the pes, and its proximal dorsal process leans medially.

The only phalanx found distal to metatarsal V is a subtriangular wedge of bone that is thickened proximally and tapers to a point (Fig 44). Its lateral margin is slightly more pronounced than the medial margin, but that region has been significantly compressed in the only pes that preserves V-1.

## Description of MCZ 8893

### Skull and mandible

**Fig 46:**
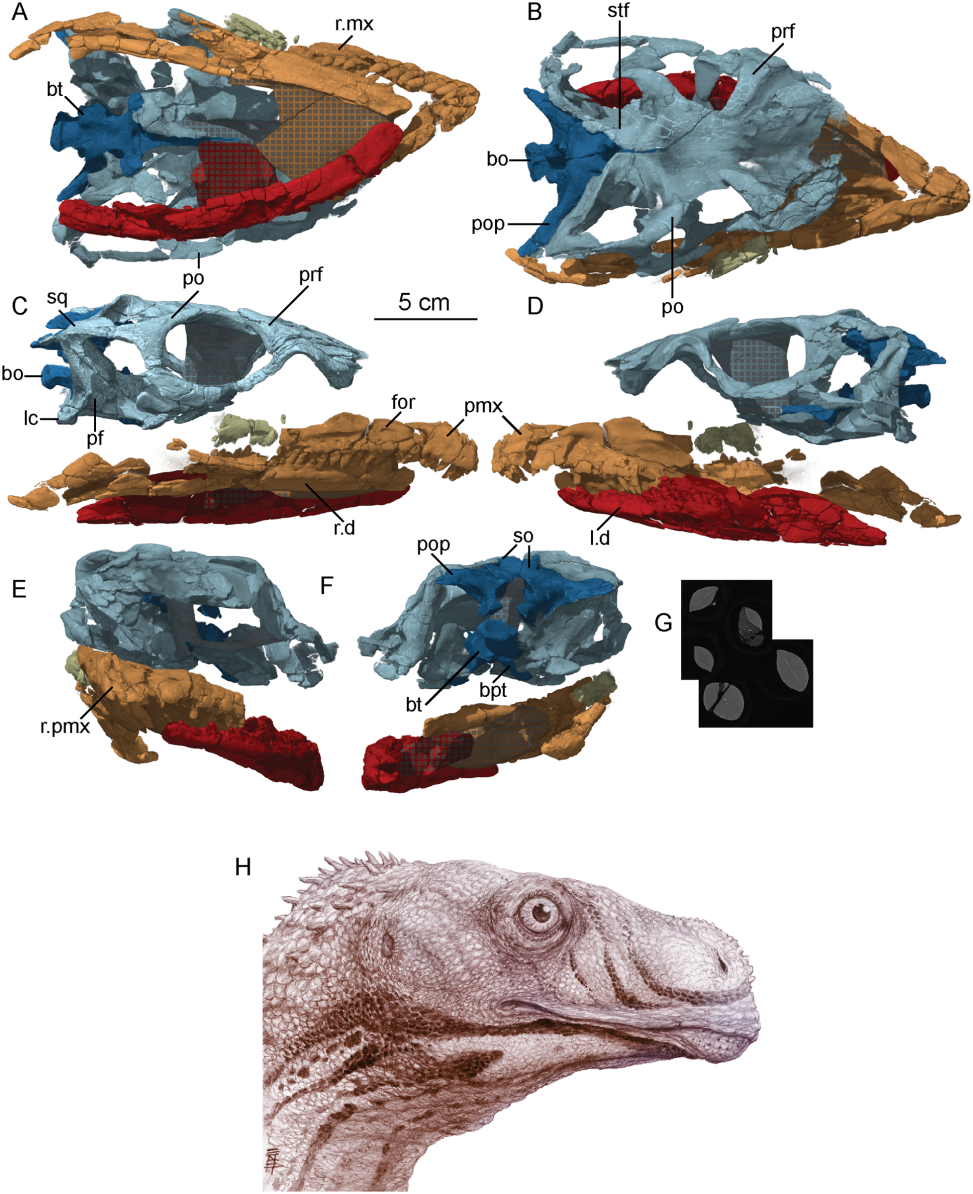
Skull (MCZ 8893) provisionally referred to *Sarahsaurus aurifontanalis*, reconstructed from CT data. A- ventral view; B- dorsal view; C- right lateral view; D- left lateral view; E- anterior view; F- posterior view; G- CT cross-sections of palatal teeth associated with the skull; H- Life reconstruction of the head of *Sarahsaurus* by Brian Engh, used with permission (http://dontmesswithdinosaurs.com/). Covered areas represent matrix on the specimen. Light and dark blue represent the skull roof and basicranium, respectively. The orange bones comprise the right mandible, maxilla, and premaxilla. Red areas indicate the left mandible. Abbreviations: basioccipital (bo), basal tuber (bt), basipterygoid process (bpt), dentary (d), foramen (for), lateral condyle (lc), maxilla (mx), pterygoid flange (pf), premaxilla (pmx), postorbital (po), paroccipital process (pop), prefrontal (prf), supraoccipital (so), squamosal (sq), supratemporal fossa (stf).

This skull is mostly complete and is only moderately distorted. As a result of cyclic swelling and shrinkage of clay in the enclosing sediment it was split horizontally at the time of discovery, separating the skull roof from the palate with commensurate damage. The specimen was briefly described and was first referred to *Massospondylus* sp., and this identification was advanced as evidence of a uniform cosmopolitan dinosaur fauna across much of Pangaea [15]. It was later provisionally referred to *Sarahsaurus aurifontanalis* [25]. The referred skull is from an individual that was less mature at time of death than the holotype (above). We provide digital animations based on high-resolution X-ray CT imagery of the skull (Fig 46; Appendix B, S2 Text).

The palatal shelves of the premaxillae are narrow. A large subnarial foramen penetrates the descending segment of the premaxillary-maxillary suture above the tooth row and a small neurovascular foramen lies above the alveolar margin behind the first tooth on the right premaxilla. The diameter of the naris is less than 50% the maximum diameter of the orbit. The external narial margin is largely formed by the premaxilla and nasal, with only a small contribution from the maxilla. Above the alveolar ramus of the maxilla projects a short facial process that tapers dorsally, inserting for a short distance between the premaxilla and nasal at the posteroventral margin of the naris. The posterior rim of the naris lies above and behind the first maxillary alveolus, and rostral to the rostral margin of the antorbital fenestra. A line of five to six large foramina of equal size open posteriorly on the lateral surface of the maxilla. The medial shelves of the maxillae were probably in contact anteriorly, but the palate is too damaged to confirm this. The jugal process of the ectopterygoid is strongly recurved and hooked. The medial process of pterygoid is flat and blunt.

The maxilla bears a concave, sharp-rimmed antorbital fossa. The fossa fails to extend onto the lacrimal, and there is evidence of a neurovascular canal opening in the fossa. The lateral margin of the nasal overhangs the antorbital fossa and forms its dorsal margin. The maxilla partially surrounds a short antorbital fenestra that is shorter than the length of the orbit. Beneath the antorbital fenestra is a long lateral maxillary lamina that is twice as long as it is high. The contact between the maxilla and lacrimal above the antorbital fenestra is visible laterally, and the lacrimal is exposed on the dorsal surface of the skull. The length of the anterior process of the lacrimal is less than half the length of its ventral process. There is no ridge on the dorsolateral surface of the lacrimal and no associated knob on the lateral aspect of the prefrontal. The jugal fails to contribute to the margin of the antorbital fenestra. The prefrontal is more extensively exposed on the skull roof than the lacrimal.

There appears to be a shallow median nasal depression, but this may be the result of postmortem damage to the rostrum. The nasal has a posterior process lying between the frontal and prefrontal. The nasal contributes to the lateral edge of the antorbital fossa dorsally but does not form a deep recess over the dorsal apex of the fossa.

The prefrontal is less than 75% the length of the frontal, but it has a long ventral process that extends down the medial side of the lacrimal. In forming the anterior border of the subcircular orbit, the lacrimal slopes anterodorsally and the jugal overlaps the lacrimal laterally. The frontal is longer than it is wide and makes a broad contribution to the orbital rim. It is gently concave in the interorbital region, which is constricted at mid-length. The frontal does not enter into the anterior margin of the supratemporal fenestra, but the caudolateral corner of the frontal contributes slightly to the supratemporal fossa just behind the articulation with the postorbital. The anterior process of the postorbital is forked at its medial contact with the frontal, and the supratemporal fossa extends onto its posterodorsal surface (this part of the frontal is not preserved in the holotype). The supratemporal fenestra is longer than it is wide. The rostral margin of the infratemporal fenestra lies behind to the orbit, but level with its dorsal margin. A suture remains widely open between the parietals, which probably reflects relative skeletal immaturity at time of death.

The ventral process of the postorbital overlaps the dorsal process of the jugal anterolaterally, whereas its posterior process overlaps the anterior process of the squamosal posterolaterally. The posterolateral process of the parietal is deflected ventrolaterally and contacts the medial process of the squamosal slightly below the level of the dorsal surface of the skull roof. The ventral process of the squamosal is strap-like and four times longer than its distal width. The quadrate foramen lies on the quadrate-quadratojugal suture. The angle between the rostral and dorsal rami of the quadratojugal is acute (~ 60°).

The braincase corresponds in detail to the holotype. There is a large postparietal fenestra between the parietal and supraoccipital. The supraoccipital is diamond-shaped and inclined at 45° so that its rostral tip lies above the basipterygoid process. The basipterygoid processes are distinct, and connected by only a narrow transverse ridge. The floor of the braincase is relatively straight with the basal tubera, basipterygoid processes, and parasphenoid rostrum aligned horizontally. A ridge is formed along the junction of the parabasisphenoid and the basioccipital, between the basal tubera and has a smooth rostral face. Co-ossification at the extremity of the basal tubera is complete such that the basioccipital and parabasisphenoid form a single rugose ridge, as in the holotype. The basal tubera are knob-like, with the basisphenoid component protruding anterior to the lateral basioccipital components, and the transverse wall between the basipterygoid processes has an indentation on its front.

The dentary has a ventral curve towards its anterior tip, typical of sauropodomorphs. The dentition is moderately heterodont, with the upper tooth row extending beyond the back of the dentary teeth. There are four premaxillary teeth, sixteen maxillary teeth, and twenty dentary teeth. The first dentary tooth is inset a short distance from the rostral tip of the dentary and is slightly procumbent. Individual tooth crowns are labiolingually compressed, taller apicobasally than wide, and convex to varying degrees mesiodistally, straight rather than recurved, and subsymmetrical in labial view. The tooth crown and root are separated by a slight constriction. The mesial and distal carinae are coarsely serrated with denticles that project apically at an angle of about 45° relative to the carina, as in other early sauropodomorphs. There are up to 20 denticles per tooth crown. The crowns are angled posteriorly relative to the long axis of the jaw and imbricate slightly, such that each tooth has its mesial margin lying lingual to the distal margin of the crown immediately in front.

MCZ 8893 contains pieces of siltstone matrix associated with the palate of the skull that supposedly include ‘palatal teeth’ that were proposed to have been embedded in the epithelium of the roof of the mouth [15]. The palatal elements of the skull lack alveoli or broken tooth bases. In cross-section, CT images indicate that these may in fact be teeth that have an extremely thin layer of exterior enamel (Fig 46). If this is the case, these elements represent palatal teeth like those found on the pterygoids of *Eoraptor lunensis* [70] and *Pampadromaeus barberenai* [72].

#### Postcrania

A few fragments of the postcranial skeleton of MCZ 8893 were also recovered as surface float, including possible atlantal neural arches, a partial cervical neural arch with small epipophyses that extend back, but not beyond, the articular faces of the postzygapophyses. There are three partial caudal centra, a partial distal humerus, an incomplete femoral shaft, and fragments of gastralia. They compare favorably in both size and anatomy with the holotype, but little more can be said of them owing to their highly fragmentary nature.

## Phylogenetic analyses

### Nomenclature

Phylogenetic definitions of relevant taxonomic names are based on the following citations: Sauropodomorpha [43], Plateosauria [152], Plateosauridae [152], Massopoda [63], Massospondylidae [153], Anchisauria [152], Sauropoda [63], Eusauropoda [154]. We prefer to use the definition of Sauropoda given by Yates [63] over that of Sereno [64] because the latter specifies *Mussaurus*, a taxon not included in one of our three data matrices. We also note that *Melanorosaurus readi* and *Riojasaurus incertus* are scored on multiple specimens of more than one taxon in the analyses of Yates [63] and Upchurch et al. [64] as discussed by McPhee and Choiniere [38]. *Melanorosaurus* and *Riojasaurus* retain retain historical character scorings in those two modified analyses in our study in order to facilitate direct comparability to the similar analyses of Rowe et al. [25] and because we have not viewed those specimens personally, but not in the modified McPhee and Choiniere matrix described below.

### Results

The initial phylogenetic study of *Sarahsaurus aurifontanalis* [25] was conducted with revised data matrices for Sauropodomorpha based on those compiled by Yates [63] and Upchurch et al. [64]. Its two primary goals were to test whether *Sarahsaurus aurifontanalis* was diagnosable as a new taxon, and to test whether Early Jurassic sauropodomorphs of North America formed their own exclusive clade. Parsimony analyses included all the taxa in each data matrix and generated strict consensus, Adams consensus, and 50% majority rules consensus trees for the revised Yates and Upchurch et al. matrices.

Our present analysis further revised character scores, added new characters, and filled in missing data thanks to more complete preparation of the paratype skeleton and CT scanning of additional material (see above). In addition, we tested the phylogenetic effects of assuming that the holotype specimen and the referred MCZ skull belong to a single taxon; we also tested the phylogenetic effects of the contrary assumption, which is that they represent separate taxa. Regardless of which assumption one makes, or which matrix one employs, our current analsysis corroborates the main point of the initial analysis [25] which is that the Early Jurassic sauropodomorphs of North America do not form a clade unto themselves.

#### Yates, 2007 [63] (Fig 47)

**Fig 47:**
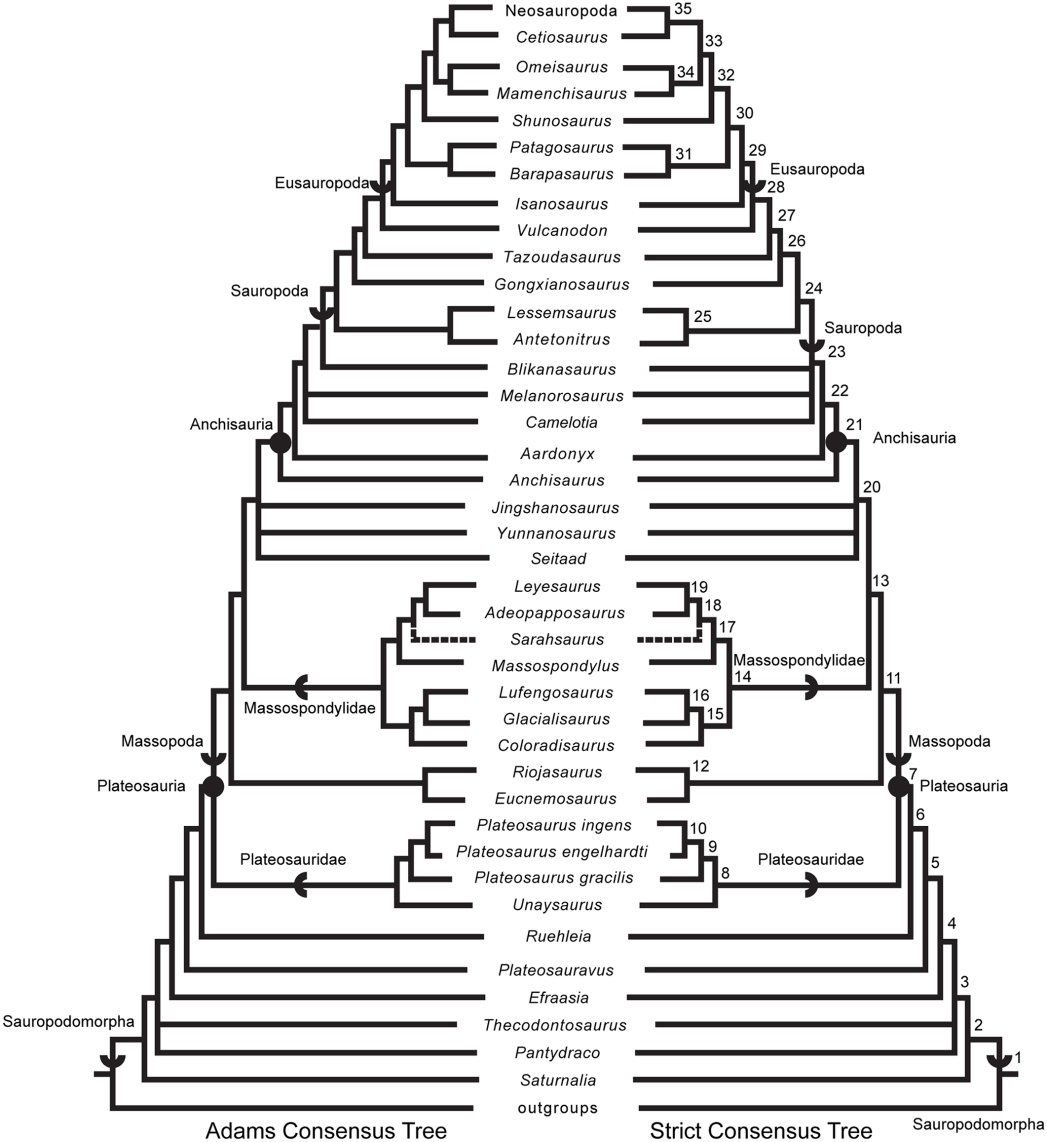
Phylogenetic hypotheses based on the revised data set of Yates [63] recovered in this study as Adams and strict consensus trees estimated from the most parsimonious trees produced by the analysis. Only the holotype of *Sarahsaurus aurifontanalis* (TMM 43646–2) was scored in this analysis. Node numbers are used in Table 1.

The heuristic search recovered 24 most parsimonious trees (MPTs) of 1212 steps (CI = 0.351, RI = 0.670). Even though our updated matrix only has two additional characters and one more taxon [25], we found 106 fewer MPTs with lengths shorter by 22 steps. Our findings on relationships among the early sauropodomorphs (*Saturnalia tupiniquim*, *Pantydraco caducus*, *Thecodontosaurus antiquus*, *Efraasia minor*, and *Plateosauravus cullingworthi*) are identical to those recovered in the initial analysis [25]. *Ruehelia bedheimensis* was excluded from Plateosauria (Fig 47). *Riojasaurus incertus* + *Eucnemosaurus* form the sister clade to all other massopodans, a sister taxon relationship found previously that was poorly resolved with respect to other plateosaurs. We also recovered a large Massospondylidae clade that includes two primary branches (found next to nodes 15 and 17 in Fig 47). In one, *Lufengosaurus hueni* and *Glacialisaurus* form the sister clade to *Coloradisaurus brevis* (including node 15). The second main branch of Massospondylidae (including node 17) includes the South American sister taxa *Adeopapposaurus mognai* and *Leyesaurus marayensis*, with *Sarahsaurus aurifontanalis* as its sister taxon. *Massospondylus carinatus* is the sister taxon to the clade including *Adeopapposaurus mognai*, *Leyesaurus marayensis*, and *Sarahsaurus aurifontanalis*. The inclusion of *Leyesaurus marayensis* helped to resolve the position of Massospondylidae within Plateosauria and pulled *Seitaad ruessi* up the tree to lie just outside of Anchisauria. The taxic composition of Anchisauria is identical in our analysis to that of the initial study [25]. The Adams consensus tree recovered the same topology as the strict consensus tree (Fig 47).

Bremer support values for the internal nodes within Sauropodomorpha are either 1 or 2, with the exception of *Riojasaurus incertus* + *Eucnemosaurus*, which is 3 (Table 1). Bootstrap resampling shows variable node support (Appendix F, S1 Figures). The bootstrap value of unnamed node 4 is 0.61, but most of the bootstrap values along the backbone of the tree are below 0.50. *Leyesaurus marayensis* + *Adeopapposaurus mognai* (node 19) had a bootstrap value of 0.42 and node 19 + *Sarahsaurus aurifontanalis* (node 18) has a bootstrap value of 0.19. Despite these low scores, however, Massosponylidae is supported by five unambiguous synapomorphies (characters 20, 99, 131, 294, 318) and one ambiguous synapomorphy (character 264).

**Table 1:** Group support for results of phylogenetic analyses from the modified Yates matrix [63].

Node	Bremer/GC bootstrap %	Node	Bremer/GC bootstrap %
2 Sauropodomorpha	1/22	7 Plateosauria	1/-
8 Plateosauridae	1/16	11 Massopoda	1/-
14 Massospondylidae	1/-	21 Anchisauria	1/5
23 Sauropoda	1/22	29 Eusauropoda	1/12

Node numbers are taken from strict consensus tree in Fig 47. Values marked by a ‘-‘ are found at nodes that were not recovered in the Bremer or bootstrap analyses. Appendix F (S1 Figures) includes the full results of the statistical analyses.

#### Upchurch et al., 2007 [64] (Fig 48)

**Fig 48:**
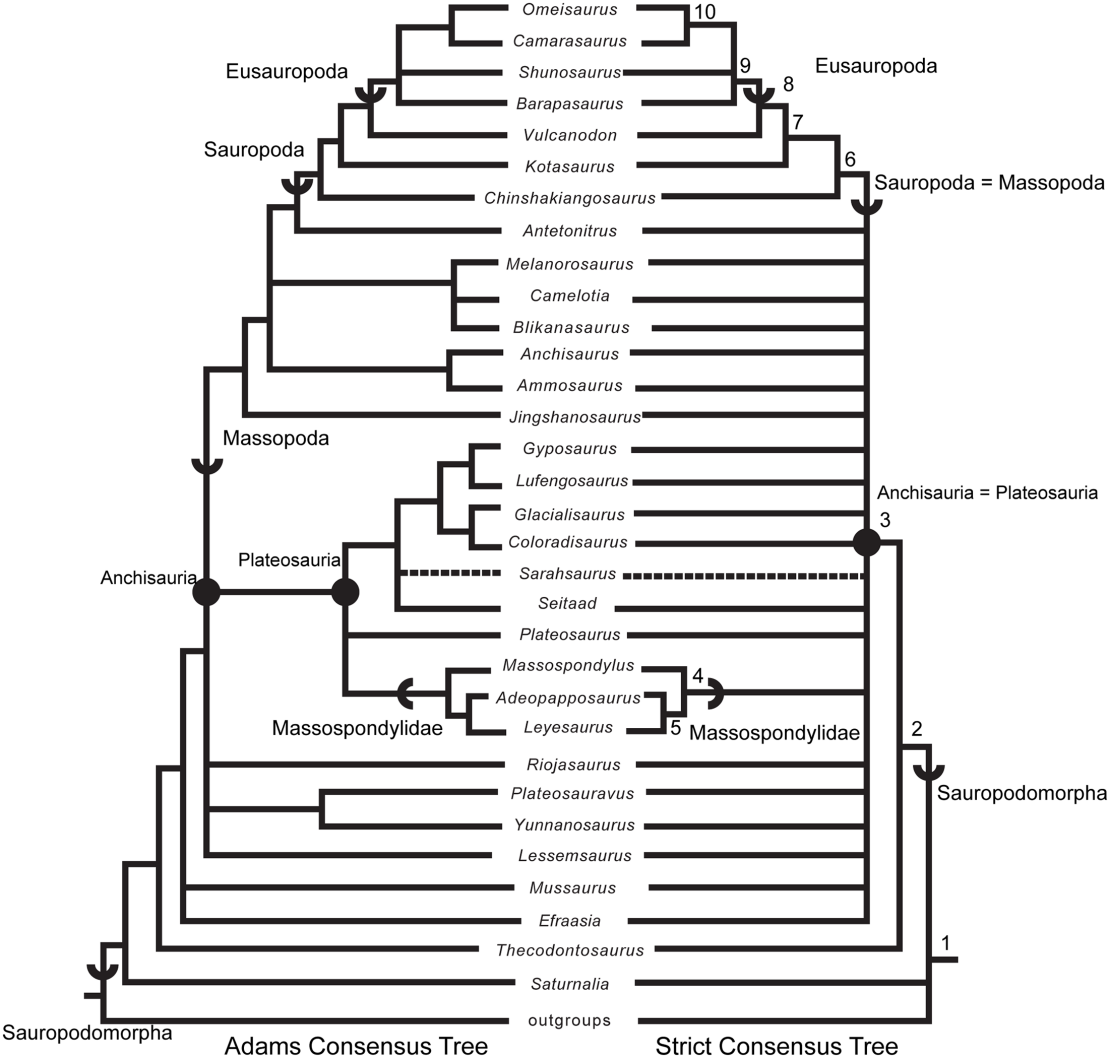
Phylogenetic hypotheses based on the revised data set of Upchurch et al. [64] recovered in this study as Adams and strict consensus trees estimated from the most parsimonious trees produced by the analysis. Only the holotype of *Sarahsaurus aurifontanalis* (TMM 43646–2) was scored in this analysis. Node numbers are used in Table 2.

We added an additional taxon and ten additional characters to the version used in the initial analysis [25]. Analysis of the revised matrix recovered 487 MPTs of 817 steps (CI = 0.398, RI = 0.651), and it yielded 3,098 fewer MPTs that are shorter by three steps. Compared to the initial results [25], *Saturnalia tupiniquim* is not unambiguously part of the ingroup (Sauropodomorpha) in the strict consensus tree (Fig 48). Much of the large polytomy at the node Plateosauria was unresolved, although we recovered a monophyletic Massospondylidae with *Adeopapposaurus mognai* + *Leyesaurus marayensis* as the sister to *Massospondylus carinatus*. *Sarahsaurus aurifontanalis* was not included in Massosponylidae in this result, and it fell in the polytomy at node 3 (Fig 48). *Melanorosaurus readi* (scored from multiple taxa in this analysis) fell within the polytomy at Plateosauria, forcing ambiguity in positions of the stem-based groups Massopoda and Sauropoda. The relationships of Eusauropoda and stem-eusauropods were the same as those recovered in the initial analysis of this matrix [25] (Fig 48).

The Adams consensus tree recovered more fully resovled relationships than the strict consensus tree (Fig 48). Most notably, it recovered *Saturnalia tupiniquim* as the sister taxon to all other sauropodomorphs, and a monophyletic Plateosauria that includes Massospondylidae plus an unnamed clade that includes *Sarahsaurus*. In effect, *Sarahsaurus aurifontanalis* acted as a wildcard taxon with *Seitaad ruessi* and a clade that includes the groups *Gyposaurus* + *Lufengosaurus hueni* and *Glacialisaurus* + *Coloradisaurus brevis* as sister taxa. *Anchisaurus polyzelus* and *Ammosaurus major* are sister taxa in the Adams consensus tree, and more resolution was recovered among non-sauropod massopods.

Because much of the strict consensus tree is unresolved, Bremer support values are 1 along the backbone nodes, with the exception of nodes 7 and higher (Bremer scores between 2 and 4; Table 2; Appendix F, S1 Figures). As expected, bootstrap support values were also low in the nodes close to Eusauropoda. The clade *Leyesaurus marayensis* + *Adeopapposaurus mognai* had a bootstrap score of 0.48. One unambiguous synapomorphy (character 218) and ten ambiguous synapomorphies (characters 14, 20, 29, 79, 94, 126, 157, 205, 215, and 257) supported a monophyletic Massospondylidae.

**Table 2:** Group support for results of phylogenetic analyses from the modified Upchurch et al. matrix [6,4].

Node	Bremer/GC bootstrap %	Node	Bremer/GC bootstrap %
2Sauropodomorpha	1/6	3 Anchisauria/Plateosauria	-/6
4 Massospondylidae	1/5	6 Sauropoda/Massopoda	-/1
8 Eusauropoda	3/77		

Node numbers are taken from strict consensus tree in Fig 48. Values marked by a ‘-‘ are found at nodes that were not recovered in the Bremer or bootstrap analyses. Appendix F (S1 Figures) includes the full results of the statistical analyses.

#### McPhee and Choiniere [38] (Fig 49)

**Fig 49:**
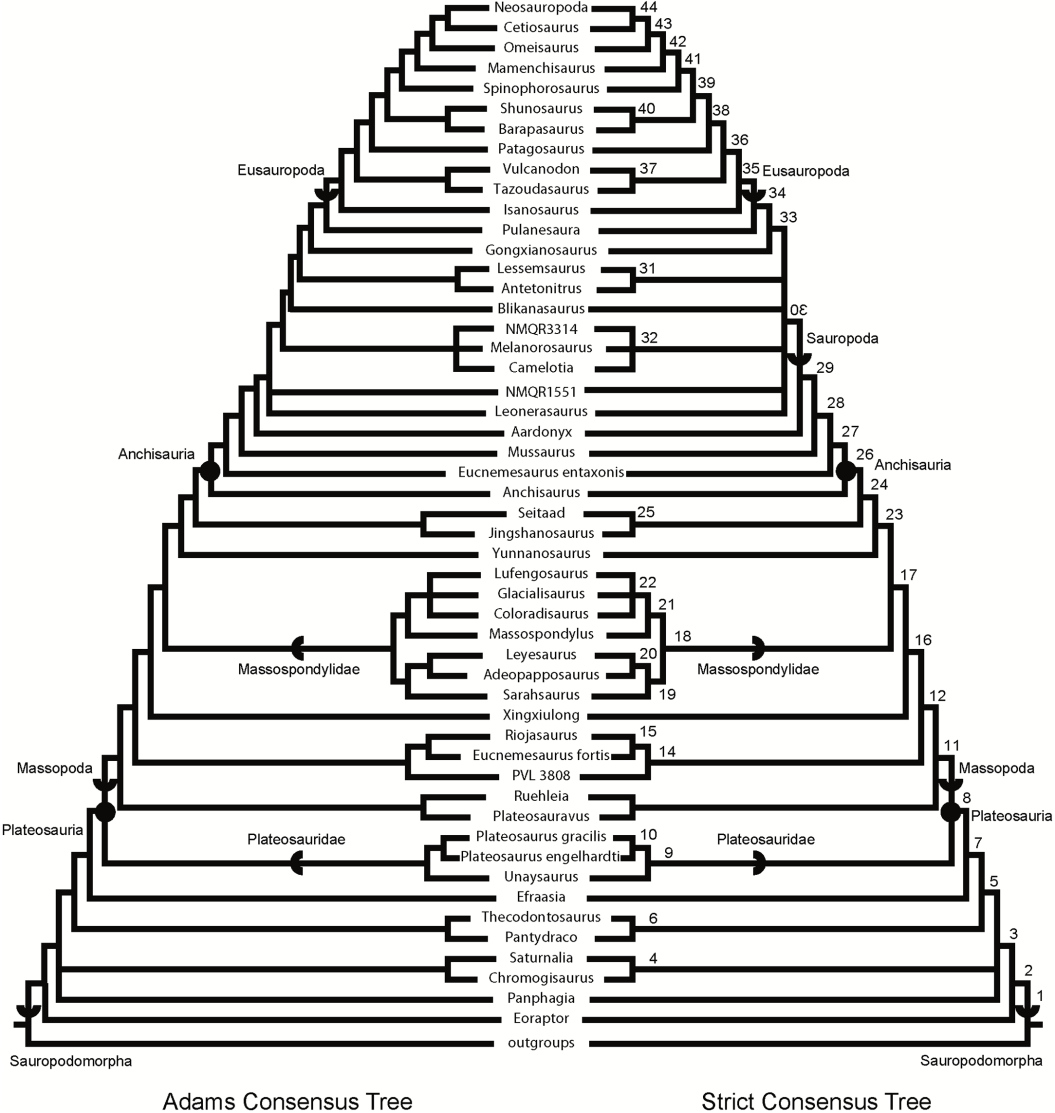
Phylogenetic hypotheses based on the revised data set of McPhee and Choiniere [38] recovered in this study as a strict consensus tree estimated from the most parsimonious trees of this analysis. Only the holotype of *Sarahsaurus aurifontanalis* (TMM 43646–2) was scored in this analysis. Node numbers are used in Table 3.

Our heuristic search recovered four MPTs of 1318 steps (CI = 0.330, RI = 0.696). Our analysis recovered the same relationships found by McPhee and Choiniere among non-plateosaur sauropodomorph. These included the *Saturnalia tupiniqium* + *Chromogisaurus novasi* clade, the *Pantydraco caducus* + *Thecodontosaurus antiquus* clade, and the *Plateosaurus* + *Unaysaurus tolentinoi* clade. In the original analysis [25], *Xingxiulong chengi* and *Sarahsaurus aurifontanalis* fell into a polytomy with all other massopods. In this analysis, *Sarahsaurus aurifontanalis* was sister taxon to the clade *Leyesaurus marayensis* + *Adeopapposaurus mognai* within a monophyletic Massospondylidae (Fig 49). Unlike the modified Yates analysis above, *Massospondylus carinatus* was more closely related to *Coloradisaurus brevis*, *Glacialisaurus hammeri*, and *Lufengosaurus hueni* (node 21) than *Adeopapposaurus mognai*, *Leyesaurus marayensis*, and *Sarahsaurus aurifontanalis* (node 19). *Seitaad ruessi* and *Jingshanosaurus xinwaensis* formed a clade with *Yunnanosaurus huangi* which collectively are the most derived non-anchisaur massopods. In contrast to the initial analysis, the two species of *Eucnemosaurus* were not recovered as sister taxa. Instead *E*. *fortis* formed a basal branch within Massopoda, while *E*. *entaxonis* was a basal branch within Anchisauria. The Adams consensus tree recovered the same topology as the strict consensus tree with the polytomy in basal anchisaurs being more resolved.

Bremer support values for the internal nodes within Sauropodomorpha fell between 1 and 3, with the exception of higher values for *Efraasia minor* plus all other Sauropodomorpha (9) and Plateosauria (7; Table 3; Appendix F, S1 Figures). Bootstrap resampling showdemonstrated low support for non-sauropod massopods (Appendix F, S1 Figures). Within Sauropodomorpha, the highest bootstrap values were found for *Efraasia minor* +all other sauropodomorphs (0.79) and for Plateosauria (0.61). *Leyesaurus marayensis* + *Adeopapposaurus mognai* (node 20) had a bootstrap value of 0.37 and node 20 + *Sarahsaurus aurifontanalis* (node 19) a bootstrap value of 0.31. Despite these low scores, Massosponylidae was supported by seven unambiguous synapomorphies (characters 67, 73, 99, 131, 139, 322, and 337).

**Table 3:** Group support for results of phylogenetic analyses from the modified McPhee and Choiniere [38].

Node	Bremer/GC bootstrap	Node	Bremer/GC bootstrap
2 Sauropodomorpha	3/37	9 Plateosauridae	4/47
8 Plateosauria	7/61	11 Massopoda	1/8
18 Massospondylidae	3/6	26 Anchisauria	2/-
30 Sauropoda	1/-	35 Eusauropoda	1/42

Node numbers are taken from strict consensus tree in Fig 49. Values marked by a ‘-‘ are found at nodes that were not recovered in the Bremer or bootstrap analyses. Appendix F (S1 Figures) includes the full results of the statistical analyses.

### Sensitivity analyses

#### Yates, 2007 [63] (Fig 50)

**Fig 50:**
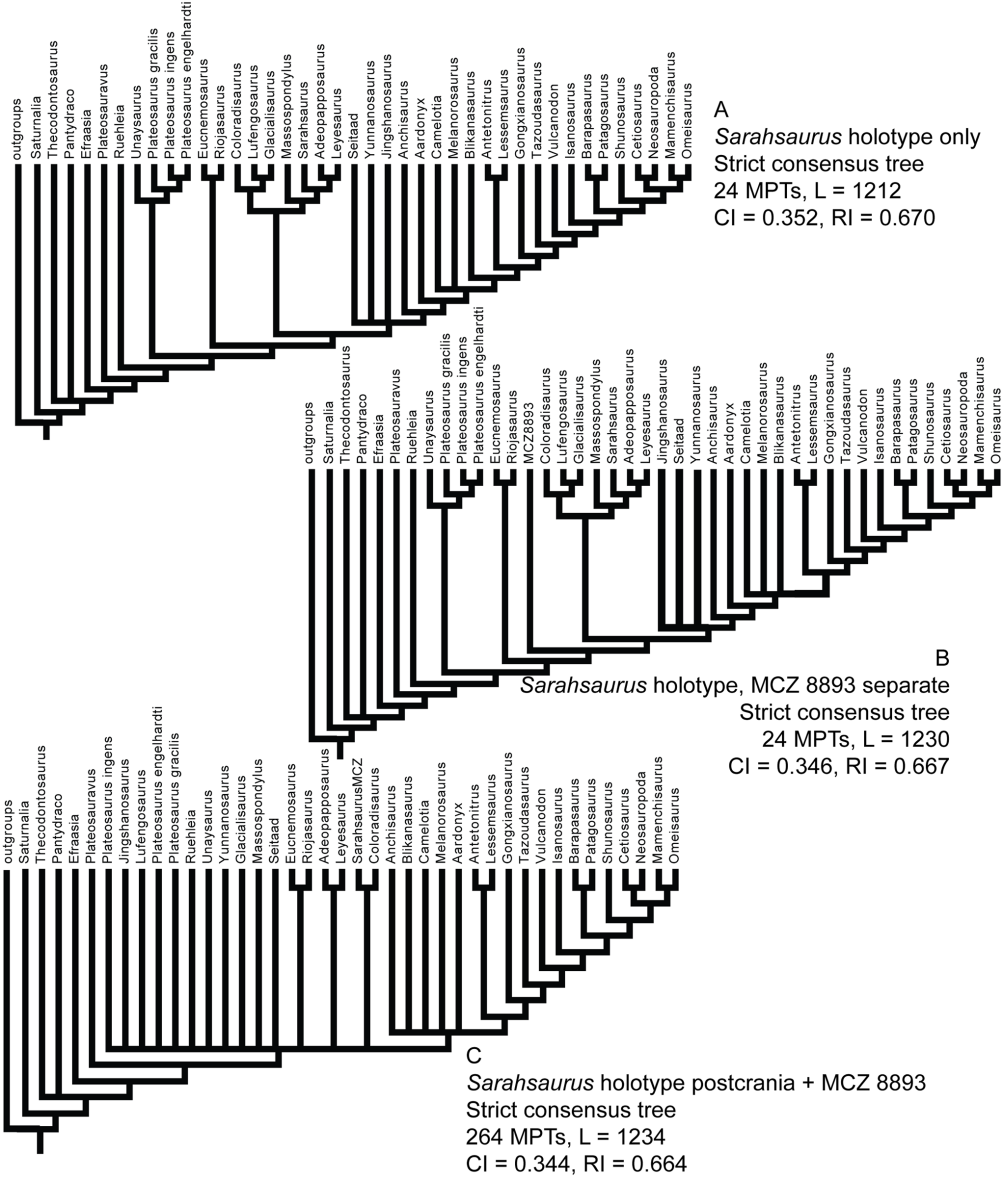
Results of the sensitivity analyses using the Yates, 2007 [63] data set. A- using only character scores from the holotype specimen of *Sarahsaurus aurifontanalis* (TMM 43646–2); B- scoring TMM 43646–2 and MCZ 8893 as separate OTUs; C- combining the character scores of TMM 43646–2 and MCZ 8893 as a single OTU.

Scored as separate OTUs, the Rock Head skull (MCZ 8893) and the holotype of *Sarahsaurus aurifontanalis* occupied different positions on the strict consensus tree. The holotype of *Sarahsaurus* remained nested within Massospondylidae as the sister taxon of *Leyesaurus marayensis* + *Adeopapposaurus mognai*. However, the Rock Head skull fell out as the sister taxon to Massospondylidae.

When *Sarahsaurus aurifontanalis* and the Rock Head skull were scored as a single OTU (the ‘combined analysis’ Fig 50C), overall resolution decreased and the number of MPTs increased. In the combined analysis, *Sarahsaurus aurifontanalis* was recovered in a strict consensus tree as the sister taxon to *Coloradisaurus brevis*. Most of the surrounding clades, including Massospondylidae collapsed in this analysis. Our analysis of the holotype alone (Fig 50A) returned same number of MPTs as the combined analysis (Fig 50B), but it was 18 steps longer.

#### Upchurch et al., 2007 [64] (Fig 51)

**Fig 51:**
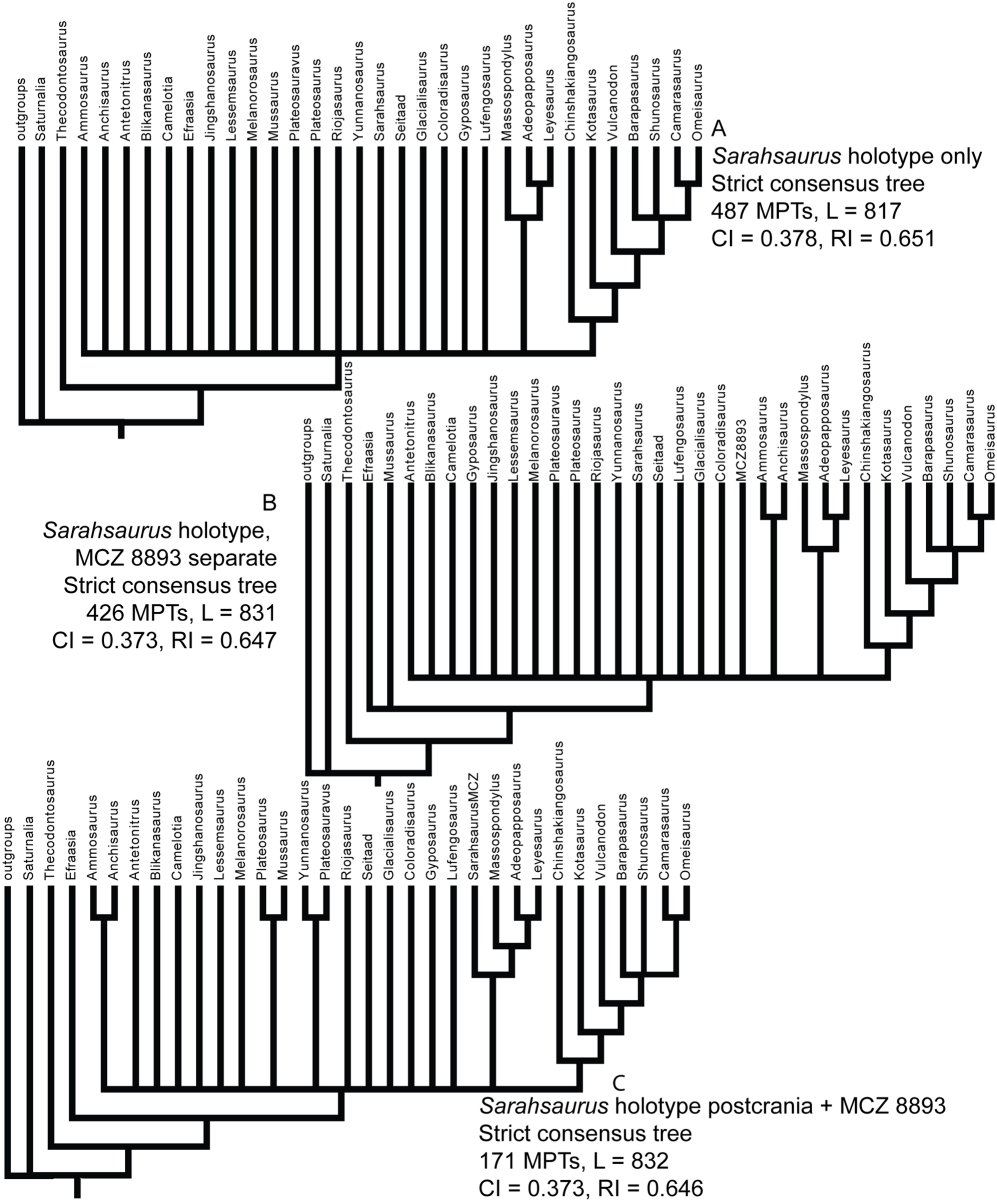
Results of the sensitivity analyses using the Upchurch et al., 2007 [64] data set. A- using only character scores from the holotype specimen of *Sarahsaurus aurifontanalis* (TMM 43646–2); B- scoring TMM 43646–2 and MCZ 8893 as separate OTUs; C- combining the character scores of TMM 43646–2 and MCZ 8893 as a single OTU.

Scoring the Rock Head and holotype specimens as separate OTUs (Fig 51B) as opposed to combining them as a single OTU (Fig 51C) decreased the number of MPTs by 61 while it increased tree length by 14 steps. Massospondylidae was recovered in both analyses but it excluded both the holotype of *Sarahsaurus aurifontanalis* and Rock Head specimens. When the holotype and Rock Head skull were scored as a single taxon, *Sarahsaurus aurifontanalis* (Fig 51C), became the sister taxon to Massospondylidae. This analysis resulted in the fewest number of MPTs.

#### McPhee and Choiniere, 2017 [38] (Fig 52)

**Fig 52:**
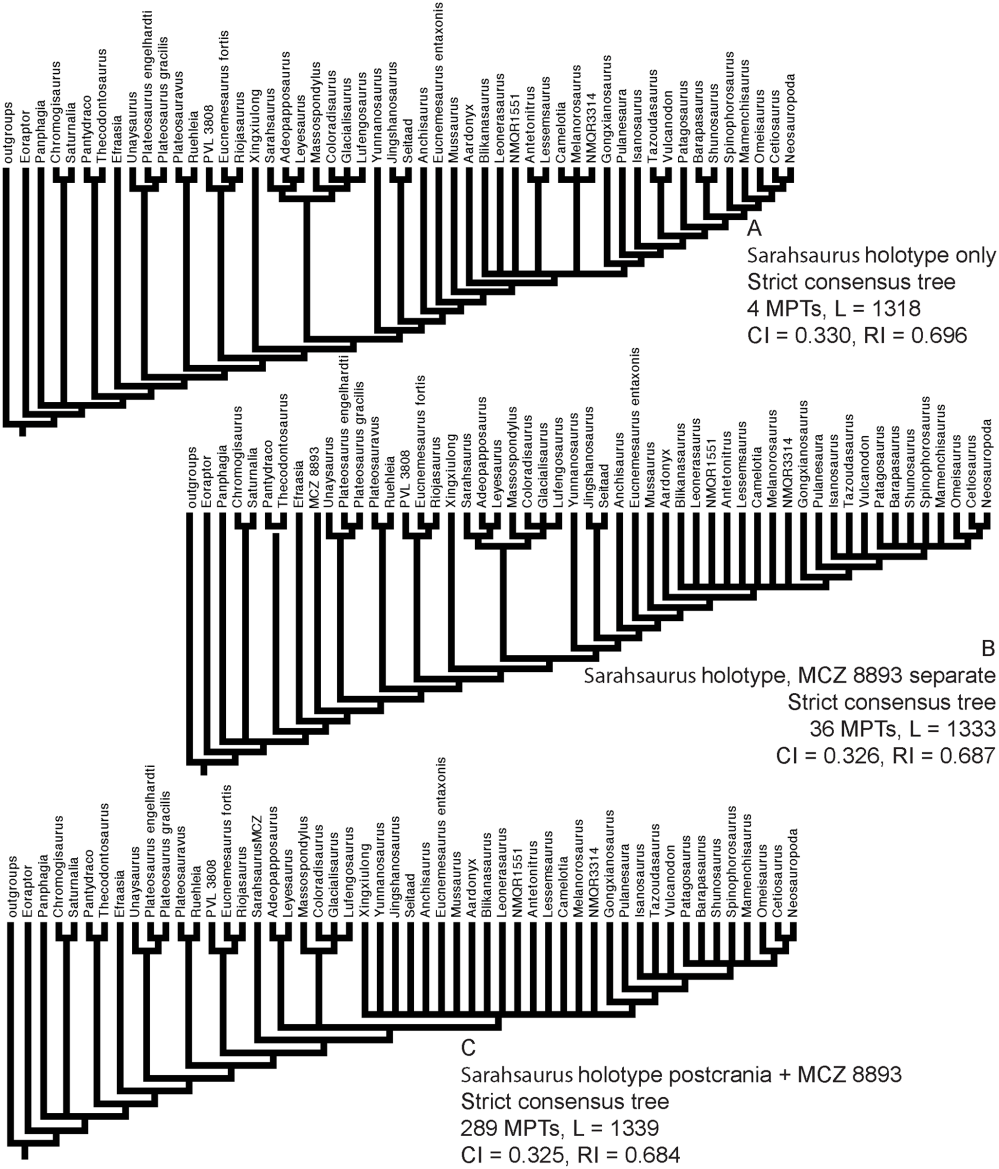
Results of the sensitivity analyses using the McPhee and Choiniere, 2017 [38] data set. A- using only character scores from the holotype specimen of *Sarahsaurus aurifontanalis* (TMM 43646–2); B- scoring TMM 43646–2 and MCZ 8893 as separate OTUs; C- combining the character scores of TMM 43646–2 and MCZ 8893 as a single OTU.

When the holotype specimen and MCZ 8893 were scored as a combined OTU (Fig 52C) as opposed to separate OTUs (Fig 52B) the analyses resulted in eight times as many MPTs and an increase in length from 1333 to 1339 (15 to 21 steps longer than if only the holotype was included; Fig 52A). As separate OTUs in the same analysis (Fig 52B) the holotype specimen was found in the same position as the holotype-only analysis (sister taxon to *Adeopapposaurus mogna*i + *Leyesaurus marayensis* within Massospondylidae) and MCZ 8893 was found in a basal position as the sister taxon to Plateosauridae + all other sauropodomorphs. The relationships of anchisaurians are more unresolved in that analysis. In the combined analysis (Fig 52C), *Sarahsaurus aurifontalis* is found outside of Massospondylidae and Massopoda is largely unresolved.

#### Results of the sensitivity analyses

Under the three matrices analyzed above, the phylogenetic placement of *Sarahsaurus aurifontanalis* was sensitive to whether or not the Rock Head specimen was included in scoring it. The sensitivity analysis using the Yates matrix raised the possibility that Rock Head specimen represents a taxon lying just outside of Massospondylidae, one that is separate from *Sarahsaurus aurifontanalis*. However, we note that it has no unique diagnostic apomorphies that would justify naming it as a separate taxon, nor is its phylogenetic position far from that of the holotype.

Under the Upchurch et al. matrix, the results of the sensitivity analysis were equivocal. With the holotype and Rock Head skull falling together into a larger polytomy, the possibility remains open that they represent a single (if unresolved) taxon, and that combining them into a single OTU can be neither accepted or rejected out of hand.

The sensitivity analyses conducted using the McPhee and Choiniere matrix supports the hypothesis that the holotype specimen of *Sarahsaurus aurifontanalis* is that of a massospondylid, even if the MCZ skull is included as a separate OTU. The more basal position of *Sarahsaurus aurifontalis* using the combined scoring in the McPhee and Choiniere matrix suggests that many of the characters found in the Rock Head skull are plesiomorphic and drag the taxon down the tree.

In light of the high degree of homolasy that was recovered in these analyses matrix the effects of differential incompleteness may explain the separation of the two specimens on the given trees. The holotype preserves realively few cranial characters but a relatively complete postcranium, while the Rock Head specimen preserves a far more complete skull but virtually no postcranial characters. Thus, differential incompleteness in the face of abundant homoplasy can equally account for the separation of the two specimens on the tree when they are included as separate OTUs. This is not surprising because those character states that are shared by the holotype and the Rock Head specimen are plesiomorphic.

### Diagnosis of *Sarahsaurus aurifontanalis*

*Sarahsaurus aurifontanalis* is represented by an exceptionally complete and semi-articulated holotype specimen that preserves autapomorphies, a combination of plesiomorphic and apomorphic features, and anatomical associations that previously were unknown or uncertain for early sauropodomorphs. An extended diagnosis for *Sarahsaurus aurifontanalis* was difficult to ascertain previously owing to the disparate topologies and character optimizations from the two data sets used in previous analyses [25] (Figs 47 and 48). Whereas many of the relationships among early sauropodomorphs remain unclear (especially those estimated from the Upchurch et al. matrix [64]), our results suggest that the position of *Sarahsaurus aurifontanalis* within Massospondylidae is supported between two different matrices. These results also offer data for an amended diagnosis for *Sarahsaurus aurifontanalis*.

There are 23 unambiguous apomorphies that diagnose *Sarahsaurus* in the modified Yates [63] matrix. Seven unambiguous and thirteen ambiguous apomorphies diagnose the taxon in the modified Upchurch et al. [64] matrix, and 20 unambiguous apomorphies diagnose the taxon in the modified McPhee and Choiniere matrix [38] (Appendix D, S4 Text). In order to not favor one phylogenetic hypothesis over the other in our analyses, we diagnose *Sarahsaurus aurifontanalis* only on the autapomorphies found in common between the three matrices. *Sarahsaurus aurifontanalis* is the only sauropodomorph dinosaur to have two foramina in the proximal region of the pubis (Fig 33). It is the only sauropodomorph dinosaur to have transversely-expanded, elaborate dorsal processes on the neural spines of the posterior cervical and anterior dorsal vertebrae (= ‘spine tables’; Figs 13–15), and a metacarpal I that is flush with the rest of the metacarpals proximally (Fig 27). In addition to these unambiguous autapomorphies, *Sarahsaurus aurifontanalis* is also diagnosed by a combination of characters; the proximal width of metacarpal I is between 65–80% of the length of the bone (Fig 28), and metacarpals II and III have deep distal extensor pits (Figs 28 and 29). While it was not borne out in the analyses, the 2-3-4-2-2 manual phalangeal count of *Sarahsaurus aurifontanalis*, specifically the two phalanges on digit V, also seems unique among sauropodomorphs (Fig 24).

## Discussion

Our analyses of *Sarahsaurus aurifontanalis* highlight some of the many systemic problems that are confronted in differing ways by the large community now working to map the phylogenetic relationships of early sauropodomorphs and other early dinosaurs. Selecting the OTUs for an analysis is among the most basic operations, and yet it is also among the most problematic operations in those cases where multiple specimens are candidates for inclusion in a composite OTU. Because the holotype and paratype of *Sarahsaurus aurifontanalis* were intermingled in the same quarry, they shared apomorphic resemblances in the cervical vertebrae and pubis, and the absence of character conflict gave us no reason to doubt their conspecificity and to treat them as as single OTU

Referral of the Rock Head specimen, found a few kilometers away from the type quarry, was more problematic. Differences between it and the holotype in maturity at time of death complicated comparisons. More tenacious is the problem of incompleteness. The Rock Head specimen consists of a fairly complete skull that was found with almost none of the postcranium, whereas the holotype and paratype consist mostly of postcranial elements. All of the features comparable favorably between the two specimens proved to be plesiomorphic in the three data matrices used in our analyses (25).

Combining the two specimens into a single OTU served to increase the matrix completeness of *Sarahsaurus aurifontanalis* from 70.5% to 92.6% in the Yates matrix [63], from 68.8% to 91.4% in the Upchurch et al. matrix [64], and 69.6% to 92.3% in the McPhee and Choiniere matrix [38]. The effects of greater completeness on tree resolution and support are well-known [68, 155], and this is the source of motivation in assembling the most complete OTUs that can be justified, ideally, by the criterion of apomorphy.

The effects of incompleteness on both tree topology and taxon diagnoses were compounded by the high degree of homoplasy found in all three matrices in which we analysed *Sarahsaurus aurifontanalis*. Our analysis found some of the homoplasy to be a result of differnt views on character conceptualization, different conclusions on the polarity of character transformations, conflicting views on the ordering of multi-state characters, and differential attention to maturity at time of death of the specimens scored.

Whether or not one adheres strictly to the criterion of apomorphy, the rationale for combining or for separating the holotype and Rock Head specimens appear to be equally balanced. Whereas ccurrent knowledges of these specimens offers no unique apomorphies that link them, the two specimens do share many corresponding, albeit plesiomorphic features that offer some measure of justification in combining them. Moreover, there are no character conflicts to separate them that are not reflections of differential maturity at time of death. And finally, there is the circumstantial evidence of their temporal and geographic correspondence that paleontologists have scrutinized for generations while weighing the identitites of different specimens found in close proxiomity.

**Fig 53:**
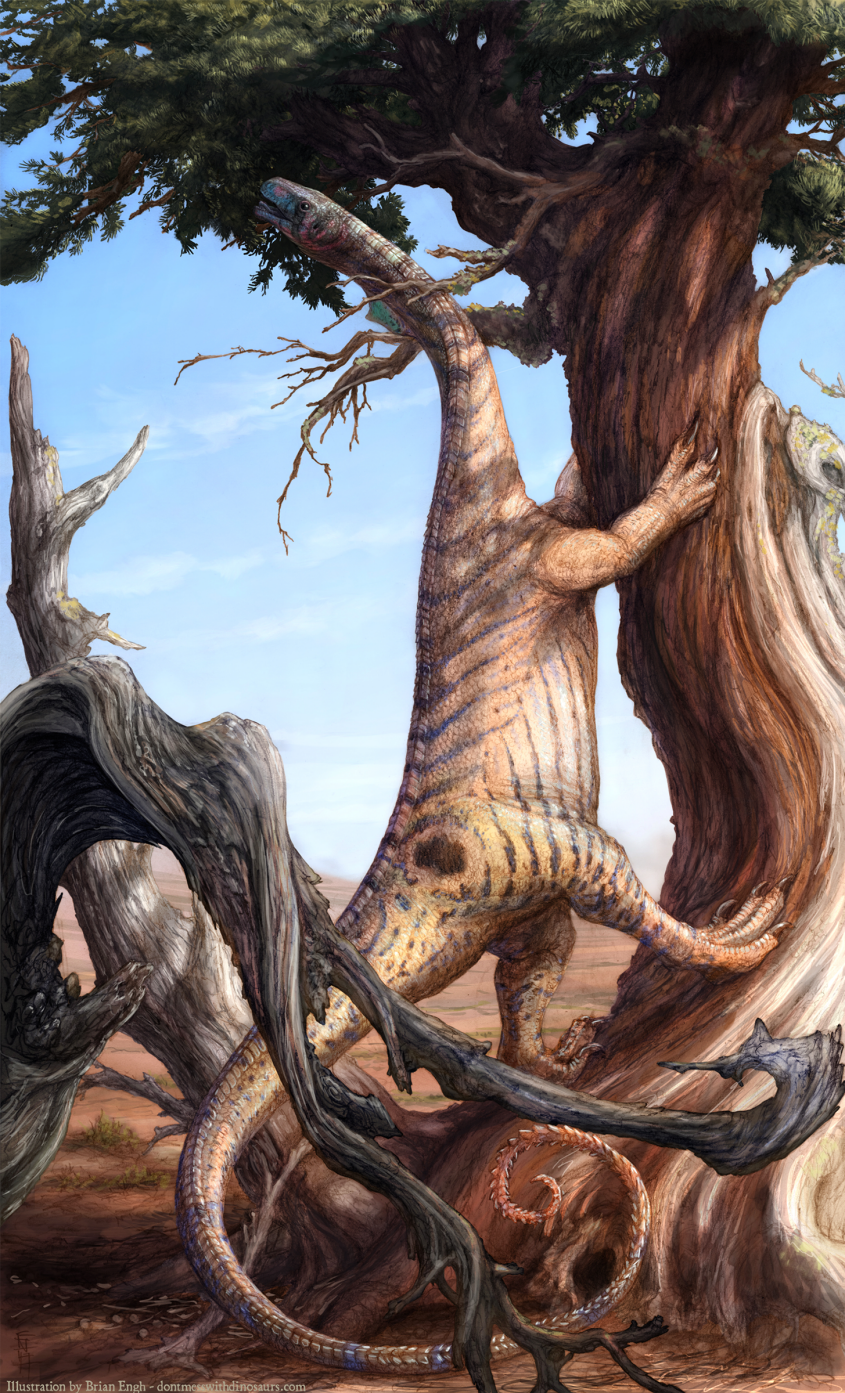
Life restoration of *Sarahsaurus aurifontanalis* showing the bipedal bauplan shared by *Sarahsaurus* and other massospondylid sauropodomorphs. Art by Brian Engh, used with permission (http://dontmesswithdinosaurs.com/).

The phylogenetic analyses conducted here suggest that *Sarahsaurus aurifontanalis* is a member of Massospondylidae, a largely Gondwanan clade of Late Triassic and Early Jurassic bipedal plateosaurian sauropodomorphs (Fig 53). Other members of this group include *Massospondylus carinatus* from southern Africa, *Adeopapposaurus mognai*, *Leyesaurus marayensis*, and *Coloradisaurus brevis* from South America, *Glacialisaurus hammeri* from Antarctica, and *Lufengosaurus huenei* from China.

### Biogeographic implications

One robust result of this study corroborates the initial analysis of *Sarahsaurus aurifontanalis* in finding that the Early Jurassic sauropodomorphs of North America do not constitiute a unique clade unto themesleves. Despite the many other uncertainties facing our understanding of early sauropodomorph evolution, this unequivocal phylogenetic result offeres a basis to infer certain local biogeographic pattens that affected the diversity of North American sauropodomorphs, and a basis to critique proposed driving mechanisms behind their early evolution. A lack of resolution in early sauropodomorph phylogeny presents impediments to charting their precisee center of origin, and many details of their subsequent dispersal routes during the Late Triassic and Jurassic remain uncertain. However, the new information on the age and relationships among the earliest North American sauropodomorphs offers a few new details about these events as well as insights into broad evolutionary mechanisms that have been hypothesized as driving early dinosaur diversification.

The initial recognition of the supercontinent Pangaea and the of plate tectonic theory in the 1970s led to a popular view in which there were two broad episodes in Mesozoic dinosaur evolution [16]. The first episode played out in the Late Triassic, as dinosaurs originated and diversified into their three major clades, Ornithischia, Sauropodomorpha, and Theropoda. All three major clades were believed to have rapidly spread across Pangaea during the Late Triassic to establish a uniform, low-diversity, and cosmopolitan community. Ease of terrestrial dispersal across Pangaea was postulated to have limited faunal differentiation that might otherwise have arisen in response to geographical isolation, explaining the broad range and uniformity of this community [9, 10, 17, 18].

Under this model, a second episode in dinosaur evolution occured as Pangaea fragmented and drifted apart during the Middle to Late Jurassic and the Cretaceous. Vicariance was hypothesized to have accelerated diversification through increased faunal isolation and provincialism, by regional extinction, and with episodic intercontinental ‘sweepstakes’ arrivals. Thus, three processes were believed to govern the early pattern of Mesozoic dinosaur diversification: vicariance, regional extinction, and dispersal. Vicariance and regional extinction were believed to generally enhance diversity while dispersal served to reduce it [9, 10, 17, 18].

The proposition of an apparent uniformity in Late Triassic terrestrial vertebrate faunas soon faced two important challenges. The first was the age the Glen Canyon Group and its global correlation. For many years, the Glen Canyon Group and its vertebrate fauna were thought to be Late Triassic in age (e.g., [47, 52, 55]). However, radiometric dating [46] recently confirmed widely-held suspicions based on biostratigraphy that the Kayenta and Navajo formations, if not the entire Glen Canyon Group, are Early Jurassic in age [56, 156, 157].

The refined dating of the Glen Canyon Group also suggests that neither ornithischian nor sauropodomorph dinosaurs were present in North America before the end-Triassic extinction event [158–161]. If there was ever a wide-spread cosmopolitan Late Triassic dinosaur community, current evidence suggests that it did not extend into North America. While this hypothesis is based on negative evidence, it is also based on careful inspection of large collections assembled from vast exposures of Late Triassic sediments over more than a century of study by many different paleontologists. To date, not one Triassic specimen has been found that preserves unequivocal apomorphies diagnostic of either Ornithischia or Sauropodomorpha, although theropod dinosaurs were indeed present in the North American Late Triassic (e.g., [161–163]).

A second challenge to the early global model of dinosaur evolution emerged from the referral of the fragmentary Glen Canyon Group vertebrates to bettter-known Gondwanan taxa. In particular, referral of the fragementary sauropodomorph specimens to *Massospondylus* sp. became questionable in the light of phylogenetic systematic methods and application of the criterion of apomorphy. The phenetic taxonomic methods used up until that that time tended to lump taxa, whereas the increased systematic rigor introduced by cladistic methods has considerably increased the taxonomic diversity now recognized among early dinosaurs [75].

If it is true that sauropodomorphs were primitively absent in the Late Triassic of North America, then *Anchisaurus polyzelus*, *Seitaad ruessi*, and *Sarahsaurus aurifontanalis* represent the earliest evidence yet discovered of the arrival of this clade in North America. It follows that the interrelationships of the North American sauropodomorphs offer a test of competing patterns of diversification. Our analysis supports the conclusion [25] that the North American sauropodomorphs do not form an exclusive clade, and it points to the likelihood of their independent dispersal into North America from different parts of Pangaea following the end-Triassic event [25]. In this case, the diversity of Early Jurassic North American sauropodomorphs is associated with dispersal, and with a process that has been referred to as ‘adaptive endmism’ [164] in which new taxa originate in response to the new local environments into which they (or their immediate ancestors) dispersed. Hence, in the case of North American sauropodomorphs, diversity was not constrained by dispersal, but rather dispersal seems more likely to have driven endemism and diversification, at least during this interval of time in which they first entered North America. With *Sarahsaurus aurifontanalis* recovered as a massospondylid, its lineage most likely originated and dispersed from Gondwana between the end of the Norian and the Pliensbachian.

Our phylogenetic analyses corroborate earlier findings [25] that the pattern of ‘foreign-relationships’ among Early Jurassic North American sauropodomorphs is not predicted by vicariance models (nor was tectonic vicariance an active phenomena in North America at this time [165]). Dispersal patterns in the face of relative tectonic stability are more difficult to predict and test. However, the multiple independent arrivals of sauropodomorphs in North America are consistent with an ‘area coalescence’ model in which taxa from separate geographic areas come together by dispersing into a newly accessible area [164, 166]. Like vicariance, this allows a large number of taxonomically diverse groups to all achieve similar changes in range [166–168]. The ‘coalescence event’ in this case was not a colliding tectonic plate. Perhaps it was the elimination of some barrier related to the end-Triassic event, or it may simply have been a matter of time in which the occupation of Pangaea by dinosaurs took longer than previously believed. In any event, current phylogenetic resolution suggests that the Early Jurassic sauropodomorphs dispersed into North America from the adjoining lands of South America and Africa, where their more ancient phylogenetic affinities lie [169].

It is worth noting that a similar pattern is seen in other Early Jurassic North American vertebrates. Current evidence suggests that ornithischians were also primitively absent in the Late Triassic in North American [158, 160], and that their oldest representatives appear simultaneously with *Sarahsaurus aurifontanalis* in the Kayenta Formation and include *Scutellosaurus lawleri* [55], ‘*Scelidosaurus’* sp. [157] and an undescribed ‘heterodontosaurid’ [15]. Based on some recent ornithischian phylogenies [123, 170] all three taxa from the Kayenta Formation are related more closely to non-North American ornithischians than to one another. Taken collectively, it appears that all the earliest North American sauropodomorphs and ornithischians originated elsewhere in Europe, Africa, or South America and dispersed onto the continent in separate events during the Early Jurassic. The tritylodontids [171, 172] and the one known goniopholid [60] from the Kayenta Formation also repeat this pattern.

The Central Atlantic Magmatic Province (CAMP) is strongly implicated in shaping these events based on its timing and geographic position [173]. CAMP activity is represented by tholeiitic dikes, sills, and lava flows in eastern North America, northern South America, northwestern Africa, and western Europe in a broad band separating the North American interior from most of the rest of Pangaea [174]. High-precision geochronology indicates CAMP activity as manifesting in a single brief magmatic episode all along the pre-Atlantic rift zone approximately 201.5 million years ago, and is temporally coincident with end-Triassic extinctions in marine faunas [173]. CAMP volcanism may have surpassed even the end-Permian Siberian flood basalts in volume and extent, profoundly altering climate, and disrupting Pangaea by opening the proto-Atlantic Ocean [172–178].

The timing of events suggests that, at least in North America, the end-Triassic mass extinction on land was not driven by competitive invasion of foreign taxa, nor is there faunal evidence of such an invasion. Theropods were present during most if not all of the Late Triassic, but only as rare faunal elements. It was not until after the end-Triassic extinctions, cessation of CAMP volcanism, and following an early Hettangian ‘recovery period’ of up to two million years [175], that sauropodomorphs and larger theropods dispersed into North America into a void left by earlier events [169]. This local snapshot is consistent with the broader picture of dinosaurs as opportunistically occupying niches left vacant by prior extinction [9, 10, 17, 179–181].

Finally, this common biogeographic pattern contradicts the assertion that dispersal reduces diversity [9, 10, 17] and was less influential than vicariance in shaping early dinosaur diversity. High-resolution phylogenetic analyses of all three clades of early dinosaurs reflect high degrees of endemism that contest the notion of a uniform cosmopolitan dinosaur community in the Late Triassic and Early Jurassic. Moreover, it suggests that the notion of a ‘cosmopolitan dinosaur fauna’ is merely an artifact of poor taxonomic resolution, and a result of confusing the evolutionary process of divergence with the historical result of accumulated morphological novelty. Late Jurassic and Cretaceous dinosaur faunas are sharply differentiated in the fragmented landmasses they occupy and are easily recognizable by more than 100 million years of accumulated novelty and divergence [25]. The Triassic and Early Jurassic patterns are subtler but are present nevertheless. For example, *Sarahsaurus aurifontanalis* is almost 40 million years removed from the earliest sauropodomorphs it is distinguishable from all other non-sauropod sauropodomorphs in its autapomorphies and a unique suite comprising dozens of character states.

The earliest North American sauropodomorphs support the view that early dinosaur diversification was driven by dispersal and adaptation over the vast and ecologically heterogeneous environs of Pangaea, and opportunistically amplified by the end-Triassic extinctions. Only later in the Jurassic, as Pangaea fragmented, was vicariance introduced as a secondary diversification factor. In light of local adaptive radiations throughout the Mesozoic, such as the Cretaceous diversification of ceratopsians in North America, dispersal across the ecologically heterogeneous continents continued to shape dinosaur diversity [164].

The relative importance of competition, vicariance, extinction, and dispersal typically is an essentialist debate seeking a single dominant cause throughout dinosaur history. However, it seems clear that these factors did not operate uniformly over time or under uniform conditions, and only in narrowed time slices and bounded regions can their roles be assessed accurately.

## Conclusion

Given its completeness, *Sarahsaurus aurifontanalis* represents the most significant increase in our knowledge of the earliest North American sauropodomorphs in a century. To estimate its phylogenetic position, we undertook phylogenetic analyses that used modified versions of matrices published by Yates [63], by Upchurch et al. [64], and by McPhee and Choiniere [38]. Given that the Yates and Upchurch et al. datasets are 42% different in character scoring [24], it was not surprising that they recovered rather different hypotheses of early sauropodomorph phylogeny. Nevertheless, all three matrices recovered a monophyletic Massospondylidae that included, minimally, (*Massospondylus carinatus* + (*Adeopapposaurus mognai* + *Leyesaurus marayensis*)).

*Sarahsaurus aurifontanalis* was nested within Plateosauria by all three matrices. Using the matrices published by Yates [63] and by McPhee and Choiniere [38], *Sarahsaurus aurifontanalis* was nested within Massospondylidae. Owing to character conflict the resolution was lower in the Upchurch el al. [64] matrix, where a large basal polytomy among plateosaurians reflected greater uncrtainty in early sauropodomorph relationships. *Sarahsaurus aurifontanalis* fell into that polytomy, and was not nested within Massospondylidae. However, when the holotype and Rock Head skull were scored as a single taxon, *Sarahsaurus aurifontanalis* became the sister taxon to Massospondylidae. Collectively, these results support the hypothesis that the holotype specimen of *Sarahsaurus aurifontanalis* is most likely a member of Massospondylidae. Whether the taxon is considered to be a massospondylid or the sister taxon of that group is sensitive to whether or not one assumes the holotype and the Rock Head skull represent a single taxon. However, one important result was that in every case North American Early Jurassic sauropodomorphs failed to form a unique clade unto themselves. This result agrees with the initial phylogenetic conclusions [25] that three different dispersals brought sauropodomorphs into North America after the end-Triassic mass extinction.

## Supporting information

S1 Text Appendix A. List of TMM specimen numbers figured in description.
(DOCX)

S2 Text Appendix B. Details of computed tomographic scans and lists of animations for TMM 43646–2 and MCZ 8893 in S1 to S21 Animations.
(DOCX)

S3 Text Appendix C. Linear measurements from undistorted holotype and paratype elements of *Sarahsaurus aurifontanalis*.
(DOCX)

S4 Text Appendix D. Character descriptions from modified Yates, Upchurch et al., and McPhee and Choiniere matrices, including a discussion on the changed character scores of *Sarahsaurus aurifontanalis* in this analysis.
(DOCX)

S1 Files Appendix E. TNT files for the phylogenetic and sensitivity analyses in this paper for the modified Yates, Upchurch et al. matrices, and McPhee and Choiniere matrices.
(ZIP)

S1 Figures Appendix F. Bremer support and GC bootstrap scores for the phylogenetic analyses in this paper.
(ZIP)

S1 Animation TMM 43646–2 braincasewithinnerear.mov. (MOV)

S2 Animation TMM 43646–2 braincase_x_spin.mov. (MOV)

S3 Animation TMM 43646–2 braincase_y_spin.mov. (MOV)

S4 Animation TMM 43646–2 hand_cutaway.mov. (MOV)

S5 Animation TMM 43646–2 hand_segmentation.mov. (MOV)

S6 Animation TMM 43646–2 hand_x_spin.mov. (MOV)

S7 Animation TMM 43646–2 hand_y_spin.mov. (MOV)

S8 Animation TMM 43646–2 segmented_hand_x_spin.mov. (MOV)

S9 Animation TMM 43646–2 segmented_hand_y_spin.mov. (MOV)

S10 Animation TMM 43646–2 carpals_x_spin.mov. (MOV)

S11 Animation MCZ 8893 skull_x_spin.mov. (MOV)

S12 Animation MCZ 8893 skull_y_spin.mov. (MOV)

S13 Animation MCZ 8893 skull_z_spin.mov. (MOV)

S14 Animation MCZ 8893 jaws_x_spin.mov. (MOV)

S15 Animation MCZ 8893 jaws_y_spin.mov. (MOV)

S16 Animation MCZ 8893 tooth1_x_spin.mov. (MOV)

S17 Animation MCZ 8893 tooth1_y_spin.mov. (MOV)

S18 Animation MCZ 8893 tooth2_x_spin.mov. (MOV)

S19 Animation MCZ 8893 tooth2_y_spin.mov. (MOV)

S20 Animation MCZ 8893 tooth3_x_spin.mov. (MOV)

S21 Animation MCZ 8893 tooth3_y_spin.mov. (MOV)

## Abbreviations

BP: Evolutionary Studies Institute (formerly the Bernard Price Institiute for Paleontological Research), Johannesburg, South Africa
BSP: Bayerische Staatssammlung für Paläontologie und Historische Geologie, München, Germany
LV: Lufeng Dinosaur Museum, Jingshan, Yunnan Province, China
MCP: Museu de Ciêncian e Tecnologia PUCS, Porto Alegre, Brazil
MCZ: Museum of Comparative Zoology, Harvard University, Cambridge, Massachusetts
SMNS: Staatliches Museum für Naturkunde, Stuttgart, Germany
TMM: Vertebrate Paleontology Laboratory, University of Texas at Austin, Austin, Texas
