# Supplementary material for: Anatomy and systematics of the sauropodomorph Sarahsaurus aurifontanalis from the Early Jurassic Kayenta Formation
Source: PLoS One. 2018 Oct 10;13(10):e0204007. doi: 10.1371/journal.pone.0204007 (PMC6179219; doi:10.1371/journal.pone.0204007)
Supplement: S1 Text — List of TMM specimen numbers figured in description. (DOCX) [file pone.0204007.s001.docx]

**Appendix A: List of TMM specimen numbers figured in description**

| **TMM 43646** | **Side** | **Element** |
| --- | --- | --- |
| **Vertebrae** |  |  |
| 2.82 | - | Pre-sacral 2-24, sacral 1-3, two anterior caudal, and 31 distal caudal vertebrae |
| 2.180 | - | Anterior caudal vertebra |
| 2.240 | - | Fused 2 anterior caudal vertebra |
| 2.16 | - | Vertebra from the middle third of the tail |
| 2.22 | - | Vertebra from the middle third of the tail |
| 2.32 | - | Vertebra from the middle third of the tail |
| 2.83 | - | Vertebra from the middle third of the tail |
| **Ribs** |  |  |
| 2.14 | L | 21, 22 |
| 2.82 | L | 5, 6, 7, 9, 12, 13, 14, 15, 17, 18, 19, 20 |
| 2.82 | R | 6, 7, 8, 12, 13, 14, 15, 16, 17, 18, 19, 20 |
| 2.287 | L | 9 |
| **Haemal arches** |  |  |
| 2.82 | - | - |
| 2.101 | - | - |
| 2.126 | - | - |
| 2.211 | - | - |
| 2.216 | - | - |
| 2.233 | - | - |
| **Skull** |  |  |
| 2.27 | L | Frontal |
| 2.35 | - | Braincase (supraoccipital, opisthotics, exoccipitals, basioccipital, prootics, parasphenoid, and basisphenoid) |
| 2.82 | R | Jugal |
| 2.88 | L | Quadrate |
| 2.230 | L | Atlantal neural arch |
| 2.291 | L | Premaxilla |
| 2.460 | L | Prefrontal |

**Appendix A: List of TMM specimen numbers figured in description, continued**

| **Pectoral girdle** |  |  |
| --- | --- | --- |
| 2.56 | L | Scapula |
| 2.57 | L | Coracoid |
| 2.82 | L | Sternal plate |
| 2.82 | R | Scapula (thin-sectioned) |
| 2.118 | L | Clavicle |
| 3.181 | L | Clavicle |
| 3.272 | R | Coracoid |
| 3.374 | R | Scapula |
| 3.399 | L | Scapula |
| **Forelimb** |  |  |
| 2.12 | R | Metacarpal IV, phalanges IV-1 and 2 |
| 2.29 | R | Radius |
| 2.58 | R | Humerus |
| 2.82 | L | Humerus and articulated forearm (radius, ulna, distal carpals, metacarpals, all phalanges) |
| 2.114 | R | Metacarpal V, phalanges V-1 and V-2 |
| 2.151 | R | Phalanx II-1 |
| 2.220 | R | Metacarpal II |
| 2.223 | R | Phalanx III-1 |
| 2.480 | R | Metacarpal III |
| 3.49 | R | Radius |
| **Pelvic girdle** |  |  |
| 2.44 | L | Ilium |
| 2.45 | L | Ischium |
| 2.50 | R | Pubis |
| 2.51 | L | Pubis |
| 2.82 | R | Ilium |
| 2.320 | R | Ischium |
| 3.271 | R | Ischium |
| 3.320 | L | Ilium |
| 3.375 | R | Pubis |
| 3.412 | L | Pubis |

**Appendix A: List of TMM specimen numbers figured in description, continued**

| **Hindlimb** |  |  |
| --- | --- | --- |
| 3.456 | R | Astragalus |
| 2.305 | R | Astragalus |
| 2.47 | L | Calcaneum |
| 2.82 | L | Articulated tibia, fibula, astragalus, calcaneum, distal tarsals II and III, metatarsals I-V, all phalanges) |
| 2.18 | R | Metatarsal II |
| 2.43 | L | Femur |
| 2.46 | L | Phalanx IV-1 |
| 2.48 | R | Metatarsal IV |
| 2.53 | R | Phalanx III-1 |
| 2.82 | L | Femur |
| 2.82 | R | Fibula |
| 2.87 | L | Phalanx I-2 |
| 2.117 | R | Phalanges I-1 and I-2 |
| 2.173 | R | Tibia |
| 2.218 | L | Metatarsal III |
| 2.224 | R | Phalanx I-2 |
| 2.243 | R | Phalanx IV-4 |
| 2.244 | R | Phalanx III-3 |
| 2.245 | R | Phalanx III-2 |
| 2.247 | R | Phalanx |
| 2.266 | R | Phalanx |
| 2.277 | R | Phalanx |
| 3.241 | R | Metatarsal II |
| 3.242 | R | Phalanx III-4 |
| 3.248 | R | Metatarsal V |
| 3.274 | R | Fibula |
| 3.366 | R | Femur |
| 3.400 | L | Tibia |
| 3.405 | R | Metatarsal III |
| 3.407 | R | Metatarsal IV |
| 3.409 | R | Metatarsal I |
| 3.420 | R | Phalanx I-1 |
| 3.457 | R | Phalanx |
| 3.485 | R | Phalanx |
|  |  |  |

**Appendix A: List of TMM specimen numbers figured in description, continued**

| **Thin-section slides and casts**   \| 1.34.1 \| *Dilophosaurus* ischium slide 1 (D1) \| \| --- \| --- \| \| 1.34.2 \| Slide 2 (D2), ischium of *Dilophosaurus* \| \| 1.34.3 \| Slide 3 (D3), ischium of *Dilophosaurus* \| \| 1.34.4 \| Slide 4 (D4), ischium of *Dilophosaurus* \| \| 1.34.5 \| Slide 5 (D5), ischium of *Dilophosaurus* \| \| 1.34.6 \| Slide 6 (D6), ischium of *Dilophosaurus* \| \| 1.34.7 \| Cast of ischium of *Dilophosaurus* \| \| 2.82.1 \| Slide 1 (S1), scapula of *Sarahsaurus* \| \| 2.82.2 \| Slide 2 (S2), scapula of *Sarahsaurus* \| \| 2.82.3 \| Slide 3 (S3), scapula of *Sarahsaurus* \| \| 2.82.4 \| Slide 4 (S4), scapula of *Sarahsaurus* \| \| 2.82.5 \| Slide 5 (S5), scapula of *Sarahsaurus* \| \| 2.82.6 \| Slide 6 (S6), scapula of *Sarahsaurus* \| \| 2.82.7 \| Slide 7 (S7), scapula of *Sarahsaurus* \| \| 2.82.8 \| Cast of scapula of *Sarahsaurus* \| |  |  |
| --- | --- | --- | --- | --- | --- | --- | --- | --- | --- | --- | --- | --- | --- | --- | --- | --- | --- | --- | --- | --- | --- | --- | --- | --- | --- | --- | --- | --- | --- | --- | --- | --- |
|  |  |  |
|  |  |  |
|  |  |  |
|  |  |  |
|  |  |  |
|  |  |  |
|  |  |  |
|  |  |  |
|  |  |  |
|  |  |  |
|  |  |  |
|  |  |  |
|  |  |  |
|  |  |  |
|  |  |  |
|  |  |  |
|  |  |  |
