# Supplementary material for: Anatomy and systematics of the sauropodomorph Sarahsaurus aurifontanalis from the Early Jurassic Kayenta Formation
Source: PLoS One. 2018 Oct 10;13(10):e0204007. doi: 10.1371/journal.pone.0204007 (PMC6179219; doi:10.1371/journal.pone.0204007)
Supplement: S2 Text — Details of computed tomographic scans and lists of animations for TMM 43646–2 and MCZ 8893 in S1 to S21 Animations. (DOCX) [file pone.0204007.s002.docx]

**Appendix B: CT datasets & Movies**

1) ***Sarahsaurus aurifontanalis* Holotype Braincase**: Scans of a fossil braincase of holotype braincase of *Sarahsaurus* *aurifontanalis* (TMM 43646-2.35) Lower Jurassic Kayenta Formation, Dilophosaurus Quarry, Navajo Nation; coll. T. Rowe and party) for Timothy Rowe of the University of Texas, Department of Geological Sciences. Specimen scanned by Matthew Colbert 4 April 2007. 16bit: 1024x1024 16-bit TIFF images. II, 200 kV, 0.16 mA, intensity control on, aluminum filter, air wedge, no offset, slice thickness 2 lines (= 0.1668 mm), S.O.D. 240 mm, 1400 views, 3 samples per view, inter-slice spacing 2 lines (= 0.1668 mm), field of reconstruction 79 mm (maximum field of view 79.57 mm), reconstruction offset 5000, reconstruction scale 1000. Acquired with 19 slices per rotation and 15 slices per set. Drift and ring-removal processing done by Alison Mote based on correction of raw sinogram data using IDL routines “RK_SinoDeDrift” with parameter “goodfile=135” and “RK_SinoRingProcSimul” with default parameters. Reconstructed with beam hardening coefficients [0, 0.8, 0.1, 0.05] and an angle of 5 degrees. Deleted the last four duplicate slices of each rotation and blank slices 1-10. Total final slices = 425. 8-bitjpg: 8bit jpg version of the above images.

Animations:

S7_Animation Braincasewithinnerear.mov 6.817 Mb

S8_Animation Sarahsaurus Braincase X spin.mov 2.816 Mb

S9 Animation Sarahsaurus Braincase Y spin.mov 3.374 Mb

2) ***Sarahsaurus aurifontanalis* Holotype left hand**: Scans of the left wrist and hand of the holotype forearm (TMM 43646-2.35) for Timothy Rowe of The University of Texas at Austin. Specimen scanned by Matthew Colbert 23 October 2009. 16bit: 1024x1024 16-bit TIFF images. P250D, 450 kV, 1.3 mA, small spot, two brass filters, air wedge, 130% offset, 64 ms integration time, slice thickness = 0.25 mm, S.O.D. 681 mm, 1600 views, 1 ray averaged per view, 1 sample per view, inter-slice spacing = 0.22 mm, field of reconstruction 180.4519 mm (maximum field of view 181.1768 mm. This scan was originally archived with an incorrectly reported field of view, which was corrected in August, 2010.), reconstruction offset 4500, reconstruction scale 4400. Streak- and ring-removal processing done by Jessie Maisano based on correction of raw sinogram data using IDL routines “RK_SinoDeStreak” with default parameters and “RK_SinoRingProcSimul” with parameters “bestof5=11, nuke_inds=[225-228,420-421].” Deleted first blank slice. Total slices = 196. 8bitjpg: 8bit jpg version of the above images.

Animations:

S10_Animation Hand cutaway.mov 0.694 Mb

S11_Animation Hand Segmentation.mov 1.920 Mb

S12_Animation Sarahsaurus Hand X Spin.mov 3.457 Mb

S13_Animation Sarahsaurus Hand Y Spin.mov 3.434 Mb

S14_Animation Segmented Hand X spin.mov 1.833 Mb

S15_Animation Segmented Hand Y spin.mov 4.451 Mb

S16_Animation Carpals X spin,mov 1.297 Mb

# 3) *Sarahsaurus aurifontanalis –* referred Rock Head skull (MCZ 8893:

CT Dataset File Name: 8893skull_jaws. Scans of parts of the skull of *Sarahsaurus* (MCZ 8893) for Timothy Rowe of The University of Texas at Austin. Specimen scanned by Matthew Colbert 3 March, 2014. 16bit: Scan parameters: NSI scanner. Scanning parameters are as follows: Feinfocus X-ray source, high-power mode, 170 kV, 0.19 mA, no filter, Perkin Elmer detector, 0.5 pF gain, 5 fps (199.982 ms integration time), 2x2 binning, no flip, source to object 220 mm, source to detector -100 mm, helical continuous CT scan, vertical extent 257 mm, pitch 40 mm, 2 revolutions, 7 sets, no frames averaged, 0 skip frames, 16010 projections, 6 gain calibrations, 15 mm calibration phantom, data range [-19.892128, 183.132431]. Total slices = 1459. Voxel size = 0.1382 mm.

Animations:

S17_Animation Skull X Spin.mov 5.447 Mb

S18_Animation Skull Y spin small.mov 3.359 Mb

S19_Animation Skull Z spin.mov 3.341 Mb

S20_Animation Jaws X spin small.mov 3.260 Mb

S21_Animation Jaws Y spin small.mov 2.812 Mb

# *4) Sarahsaurus aurifontanalis –* possible teeth recovered with the referred Rock Head skull (MCZ 8893)

CT Dataset File Name: 8893teethA. Scans of three individual teeth of *Sarahsaurus* (MCZ 8893) for Tim Rowe of UT DOGS. All three teeth were scanned together and then digitally separated for the individual movies included here as supplemental data. Scanning parameters are as follows: Specimens scanned by Jessie Maisano 26 March 2014. Scan parameters: Xradia. LFOV objective, 90kV, 10W, 1.5s acquisition time, detector 137 mm, source -37 mm, XYZ [-958, 11379, 1495], camera bin 2, angles ±180, 1261 views, 2.1 mm SiO2 filter, dithering. End reference (45 frames, each for 1.5s). Reconstructed with center shift -5, beam hardening 0.17, theta 0, byte scaling [-12, 400], binning 1, recon filter smooth (kernel size = 0.5). Total slices = 686. 16bit: 16bit TIFF images reconstructed by Xradia Reconstructor. Voxels are 14.30 microns. 8bitjpg: 8bit jpg version of the reconstructed images.

Animations:

S22_Animation Tooth 1 X spin.mov 3.534 Mb

S23_Animation Tooth 1 Y spin.mov 4.279 Mb

S24_Animation Tooth 2 X spin.mov 3.590 Mb

S25_Animation Tooth 2 Y spin.mov 4.278 Mb

S26_Animation Tooth 3 X spin.mov 4.057 Mb

S27_Animation Tooth 3 Y spin.mov 4.279 Mb
