# Supplementary material for: Anatomy and systematics of the sauropodomorph Sarahsaurus aurifontanalis from the Early Jurassic Kayenta Formation
Source: PLoS One. 2018 Oct 10;13(10):e0204007. doi: 10.1371/journal.pone.0204007 (PMC6179219; doi:10.1371/journal.pone.0204007)
Supplement: S3 Text — Linear measurements from undistorted holotype and paratype elements of Sarahsaurus aurifontanalis. (DOCX) [file pone.0204007.s003.docx]

**Appendix C: Linear measurements from undistorted holotype and paratype elements of *Sarahsaurus***

| Vertebra | Centrum length | Inter-zygapophyseal distance | Total height | Neural spine height | Neural spine length |
| --- | --- | --- | --- | --- | --- |
| 2 (cv2) | 55.79 | 53.40 | 38.87 | 9.84 | 51.36 |
| 3 (cv3) | 78.02 | 83.89 | 38.56 | 9.04 | 38.83 |
| 4 (cv4) | 88.93 | 108.97 | 40.25 | 8.94 | 50.70 |
| 5 (cv5) | 98.43 | 113.11 | 42.90 | 9.32 | 63.84 |
| 6 (cv6) | 99.47 | 117.09 | 48.81 | 11.87 | 57.81 |
| 7 (cv7) | 91.98 | 111.84 | 60.09 | 12.42 | 50.79 |
| 8 (cv8) | 86.25 | 110.46 | 67.81 | 13.82 | 46.16 |
| 9 (cv9) | 79.21 | 112.34 | 80.31 | 17.79 | 38.71 |
| 10 (cv10) | 77.56 | 104.73 | 94.67 | 18.61 | 29.17 |
| 11 (dv1) | 64.74 | 86.96 | 101.28 | 18.98 | 26.59 |
| 12 (dv2) | 59.09 | 76.21 | 99.43 | 17.13 | 23.22 |
| 13 (dv3) | 57.87 | 68.23 | 96.77 | 17.99 | 26.42 |
| 14 (dv4) | 51.14 | 73.68 | 90.91 | 24.55 | 31.51 |
| 15 (dv5) | 53.30 | 75.64 | 91.35 | 25.59 | 32.42 |
| 16 (dv6) | 56.20 | 79.39 | 94.27 | 27.77 | 39.05 |
| 17 (dv7) | 58.65 | 81.50 | 92.51 | 30.63 | 45.94 |
| 18 (dv8) | 60.17 | 88.18 | 97.39 | 34.96 | 47.08ǂ |
| 19 (dv9) | 57.79 | 86.83 | 101.90 | 35.40 | 46.13 |
| 20 (dv10) | 60.85 | 87.35 | 111.98 | 36.29 | 44.80 |
| 21 (dv11) | 59.20 | 88.78 | 120.84 | 41.49 | 46.76 |
| 22 (dv12) | 59.36ǂ | 89.00 | 127.99 | 40.07 | 41.11 |
| 23 (dv13) | 59.5ǂ | 89.30 | 121.56 | 39.97 | 39.50 |
| 24 (dv14) | 47.17 | 73.58 | 123.84 | 44.50 | 34.56ǂ |
| all measurements in mm |  |  |  |  |  |
| ǂ broken or pathological |  |  |  |  |  |

**Appendix C: Linear measurements from undistorted holotype and paratype elements of *Sarahsaurus*, continued**

|  | Scapula | Coracoid | Ilium | Ischium | Pubis |
| --- | --- | --- | --- | --- | --- |
|  | L, 2.56 | L, 2.57 | L, 2.44 | L, 2.45 | L, 2.51 |
| Total length | 250.72 | 141.03 | 230.57 | 257.3 | 296.26 |
| Max proximal width | 119.36 | - | - | - | - |
| Max midshaft width | 37.84 | - | - | - | - |
| Min midshaft width | 17.33 | - | - | - | - |
| Max distal width | 79.64 | - | - | - | - |
| Acromion length | 61.15 | - | - | - | - |
| Preacetabluar process length | - | - | 35.53 | - | - |
| Ischial peduncle length | - | - | 44.49 | - | - |
| Pubic peduncle length | - | - | 99.69 | - | - |
| Max ischial pedicle width | - | - | - | - | 86.26 |
| Min ischial pedicle width | - | - | - | - | 9.28 |
| Max pubic pedicle width | - | - | - | 56.91 | - |
| Min pubic peduncle width | - | - | - | 9.49 | - |
| Max iliac pedicle width | - | - | - | 65.63 | 87.16 |
| Min iliac pedicle width | - | - | - | 30.2 | 30.76 |
| all measurements in mm |  |  |  |  |  |
| - not applicable |  |  |  |  |  |
| * distorted |  |  |  |  |  |
| ǂ broken |  |  |  |  |  |

**Appendix C: Linear measurements from undistorted holotype and paratype elements of *Sarahsaurus*, continued**

|  | Humeri | | Radii |  |  | Ulna |
| --- | --- | --- | --- | --- | --- | --- |
|  | R, 2.58 | L, 2.82 | R, 2.29 | L, 2.82 | R, 3.49 | L, 2.82 |
| Total length | 232.50 | 258.30 | 154.40 | 147.30 | 140.77 | 161.38 |
| Max proximal width | 87.43* | 70.18 | 35.10 | 37.08 | 40.07 | 49.78 |
| Min proximal width | 28.73* | 23.78 | 19.16 | 12.19 | 19.15 | 13.40 |
| Max proximal height | - | - | - | - | - | - |
| Max midshaft width | 39.25 | 32.09 | 19.15 | 20.02 | 19.88 | 23.84 |
| Min midshaft width | 20.86* | 15.42* | 11.72* | 8.84* | 9.69 | 9.90 |
| Max distal width | 72.97 | 77.36* | 26.48 | 27.95 | 28.93 | 20.94 |
| Min distal width | - | - | 17.12* | 18.33* | 20.01* | 19.34* |
| Max distal height | - | - | - | - | - | - |
| Deltopectoral crest length | 108.98 | 119.07 | - | - | - | - |
| Max width ulnar condyle | 21.48 | 13.02* | - | - | - | - |
| Max width radial condyle | 22.48 | 14.16* | - | - | - | - |
| all measurements in mm |  |  |  |  |  |  |
| - not applicable |  |  |  |  |  |  |
| * distorted |  |  |  |  |  |  |
| ǂ broken |  |  |  |  |  |  |

**Appendix C: Linear measurements from undistorted holotype and paratype elements of *Sarahsaurus*, continued**

|  | Metacarpals | |  |  |  |  |  |  |  |
| --- | --- | --- | --- | --- | --- | --- | --- | --- | --- |
|  | R II, 2.220 | R III, 2.480 | R IV, 2.12 | R V, 2.114 | L I, 2.82 | L II, 2.82 | L III, 2.82 | L IV, 2.82 | L V, 2.82 |
| Total length | 64.60 | 59.94 | 46.79 | 32.46 | 51.95 | 63.42 | 59.61 | 51.29 | 32.28 |
| Max proximal width | 26.72 | 23.00 | 11.42 | 21.87 | 36.88 | 20.47 | 12.23* | 19.13* | 13.08* |
| Min proximal width | - | - | - | - | - | - | - | - | - |
| Max proximal height | 16.81 | 10.87* | 16.70 | 9.83 | 23.04 | 11.43* | 12.92 | 8.21* | 6.37* |
| Max midshaft width | - | - | - | - | - | - | - | - | - |
| Min midshaft width | - | - | - | - | - | - | - | - | - |
| Max distal width | 21.29 | 20.30 | 10.72 | 15.90 | 38.33 | 15.21* | 15.58* | 15.62* | 12.91* |
| Min distal width | - | - | - | - | - | - | - | - | - |
| Max distal height | 17.85 | 9.14* | 13.17 | 8.84 | 12.22* | 16.81 | 9.46* | 7.79* | 5.98* |
| all measurements in mm |  |  |  |  |  |  |  |  |  |
| - not applicable |  |  |  |  |  |  |  |  |  |
| * distorted |  |  |  |  |  |  |  |  |  |
| ǂ broken |  |  |  |  |  |  |  |  |  |

**Appendix C: Linear measurements from undistorted holotype and paratype elements of *Sarahsaurus*, continued**

|  | Metatarsals | |  |  |  |  |  |  |
| --- | --- | --- | --- | --- | --- | --- | --- | --- |
|  | R II, 2.18 | R III, 2.218 | R IV, 2.48 | L I, 3.409 | L II, 3.241 | L III, 3.405 | L IV, 3.407 | L V, 3.248 |
| Total length | 139.46 | 147.99 | 137.02 | 103.31 | 141.76 | 158.65* | 146.78 | 83.56 |
| Max proximal width | 39.22 | 29.51 | 55.88 | 45.06 | 24.34ǂ | 44.34* | 52.23 | 48.06 |
| Min proximal width | - | - | - | - | - | - | - | - |
| Max proximal height | 41.81* | 38.45 | 16.99 | 12.53* | 47.63 | 17.47ǂ | 15.25* | 18.23 |
| Max midshaft width | - | - | - | - | - | - | - | - |
| Min midshaft width | - | - | - | - | - | - | - | - |
| Max distal width | 40.75 | 41.53 | 41.20 | 40.63 | 31.76 | 41.07 | 36.77 | 15.73 |
| Min distal width | - | - | - | - | - | - | - | - |
| Max distal height | 22.79 | 26.79 | 22.72 | 15.11* | 22.03 | 20.50 | 17.18 | 11.91 |
| all measurements in mm |  |  |  |  |  |  |  |  |
| - not applicable |  |  |  |  |  |  |  |  |
| * distorted |  |  |  |  |  |  |  |  |
| ǂ broken |  |  |  |  |  |  |  |  |

**Appendix C: Linear measurements from undistorted holotype and paratype elements of *Sarahsaurus*, continued**

|  | Manual unguals | |  |  |  | Pedal unguals | |  |  |
| --- | --- | --- | --- | --- | --- | --- | --- | --- | --- |
|  | L I-2, 2.82 | L II-3, 2.82 | L III-4, 2.82 | L IV-2, 2.82 | L V-2, 2.82 | L I-2, 2.82 | L II-3, 2.82 | L III-4, 2.82 | L IV-5, 2.82 |
| Total length | 75.46 | 40.13 | 16.05 | 10.54 | 8.35 | 70.18 | 57.67 | 50.19 | 36.14ǂ |
| Max proximal width | 14.71* | 11.92 | 11.65 | 10.98* | 14.47* | 29.15 | 24.55 | 20.73 | 23.82* |
| Min proximal width | - | - | - | - | - | - | - | - | - |
| Max proximal height | 39.84 | 18.69 | 15.36 | 12.13* | 6.90* | 28.15 | 20.80 | 17.51 | 14.39* |
| all measurements in mm |  |  |  |  |  |  |  |  |  |
| - not applicable |  |  |  |  |  |  |  |  |  |
| * distorted |  |  |  |  |  |  |  |  |  |
| ǂ broken |  |  |  |  |  |  |  |  |  |
