# Supplementary material for: Anatomy and systematics of the sauropodomorph Sarahsaurus aurifontanalis from the Early Jurassic Kayenta Formation
Source: PLoS One. 2018 Oct 10;13(10):e0204007. doi: 10.1371/journal.pone.0204007 (PMC6179219; doi:10.1371/journal.pone.0204007)
Supplement: S4 Text — Character descriptions from modified Yates, Upchurch et al., and McPhee and Choiniere matrices, including a discussion on the changed character scores of Sarahsaurus aurifontanalis in this analysis. (DOCX) [file pone.0204007.s004.docx]

**Appendix D: Character descriptions**

**A) Modified matrix of Yates (2007) used for phylogenetic analyses**

**Characters 1-353 from Yates (2007)**

**Character 1**. Skull to femur ratio

0: greater than

1: less than 0.6

**Character 2**. Lateral plates appressed to the labial side of the premaxillary, maxillary and dentary teeth:

0: absent

1: present

**Character 3**. Relative height of the rostrum at the posterior margin of the naris:

0: more than

1: less than 0.6 of the height of the skull at the middle of the orbit

**Character 4**. Foramen on the lateral surface of the premaxillary body:

0: absent

1: present

**Character 5**. Distal end of the dorsal premaxillary process:

0: tapered

1: transversely expanded

**Character 6**. Profile of premaxilla:

0: convex

1: with an inflection at the base of the dorsal process

**Character 7**. Size and position of the posterolateral process of premaxilla:

0: large and lateral to the anterior process of the maxilla

1: small and medial to the anterior process of the maxilla

**Character 8**. Relationship between posterolateral process of the premaxilla and the anteroventral process of the nasal:

0: broad sutured contact

1: point contact

2: separated by maxilla

**Character 9**. Posteromedial process of the premaxilla:

0: absent

1: present

**Character 10**. Shape of the anteromedial process of the maxilla:

0: narrow, elongated and projecting anterior to lateral premaxilla-maxilla suture

1: short, broad and level with lateral premaxilla-maxilla suture

**Character 11**. Development of external narial fossa:

0: absent to weak

1: well developed with sharp posterior and anteroventral rims

**Character 12**. Development of narial fossa on the anterior ramus of the maxilla:

0: weak and orientated laterally to dorsolaterally

1: well developed and forming a horizontal shelf

**Character 13**. Size and position of subnarial foramen :

0: absent

1: small (no larger than adjacent maxillary neurovascular foramina) and positioned outside of narial fossa

2: large and on the rim of, or inside, the narial fossa

**Character 14**. Shape of subnarial foramen:

0: rounded

1: slot-shaped

**Character 15**. Maxillary contribution to the margin of the narial fossa:

0: absent

1: present

**Character 16**. Diameter of external naris:

0: less than

1: greater than 0.5 of the orbital diameter

**Character 17**. Shape of the external naris (in adults):

0: rounded

1: subtriangular with an acute posteroventral corner

**Character 18**. Level of the anterior margin of the external naris:

0: anterior

1: posterior to the midlength of the premaxillary body

**Character 19**. Level of the posterior margin of external naris :

0: anterior to, or level with the premaxilla-maxilla suture

1: posterior to the first maxillary alveolus

2: posterior to the midlength of the maxillary tooth row and the anterior margin of the antorbital fenestra.

**Character 20**. Dorsal profile of the snout:

0: straight to gently convex

1: with a depression behind the naris

**Character 21**. Elongate median nasal depression:

0: absent

1: present

**Character 22**. Width of anteroventral process of nasal at its base:

0: less than

1: greater than width of anterodorsal process at its base

**Character 23**. Nasal relationship with dorsal margin of antorbital fossa:

0: not contributing to the margin of the antorbital fossa

1: lateral margin overhangs the antorbital fossa and forms its dorsal margin

2: overhang extensive, obscuring the dorsal lachrymal maxilla contact in lateral view

**Character 24**. Pointed caudolateral process of the nasal overlapping the lachrymal:

0: absent

1: present

**Character 25**. Anterior profile of the maxilla:

0: slopes continuously towards the rostral tip

1: with a strong inflection at the base of the ascending ramus, creating a rostral ramus with parallel dorsal and ventral margins

**Character 26**. Length of rostral ramus of the maxilla:

0: less than

1: greater than its dorsoventral depth

**Character 27**. Shape of the main body of the maxilla:

0: tapering posteriorly

1: dorsal and ventral margins parallel for most of their length

**Character 28**. Shape of the ascending ramus of the maxilla in lateral view:

0: tapering dorsally

1: with an anteroposterior expansion at the dorsal end

**Character 29**. Rostrocaudal length of the antorbital fossa:

0: greater

1: less than that of the orbit

**Character 30**. Posteroventral extent of medial wall of antorbital fossa:

0: reaching

1: terminating anterior to the anterior tip of the jugal

**Character 31**. Development of the antorbital fossa on the ascending ramus of the maxilla:

0: deeply impressed and delimited by a sharp, scarp-like rim

1: weakly impressed and delimited by a rounded rim or a change in slope

**Character 32**. Shape of the antorbital fossa:

0: crescentic with a strongly concave posterior margin that is roughly parallel to the rostral margin of the antorbital fossa

1: subtriangular with a straight to gently concave posterior margin

2: or antorbital fossa absent

**Character 33**. Size of the neurovascular foramen at the caudal end of the lateral maxillary row (Yates 2003b):

0: not larger than the others

1: or distinctly larger than the others in the row

**Character 34**. Direction that the neurovascular foramen at the caudal end of the lateral maxillary row opens:

0: caudally

1: or rostrally, ventrally or laterally

**Character 35**. Arrangement of lateral maxillary neurovascular foramina:

0: linear

1: or irregular

**Character 36**. Longitudinal ridge on the posterior lateral surface of the maxilla:

0: absent

1: present

**Character 37**. Dorsal exposure of the lachrymal:

0: present

1: absent

**Character 38**. Shape of the lachrymal:

0: dorsoventrally short and blockshaped

1: dorsoventrally elongate and shaped like an inverted L

**Character 39**. Orientation of the lachrymal orbital margin:

0: strongly sloping anterodorsally

1: or erect and close to vertical

**Character 40**. Length of the anterior ramus of the lachrymal :

0: greater than

1: or less than half the length of the ventral ramus,

2: or absent altogether

**Character 41**. Web of bone spanning junction between anterior and ventral rami of lachrymal:

0: absent and antorbital fossa laterally exposed

1: or present, obscuring posterodorsal corner of antorbital fossa

**Character 42**. Extension of the antorbital fossa onto the ventral end of the lachrymal:

0: present

1: or absent

**Character 43**. Length of the caudal process of the prefrontal:

0: short

**Character 43**. Length of the caudal process of the prefrontal:

0: short

1: or elongated, so that total prefrontal length is equal to the rostrocaudal diameter of the orbit

**Character 44**. Ventral process of prefrontal extending down the posteromedial side of the lachrymal:

0: present

1: absent

**Character 45**. Maximum transverse width of the prefrontal:

0: less than

1: more than 0.25 of the skull width at that level

**Character 46**. Shape of the orbit:

0: subcircular

1: or ventrally constricted making the orbit subtriangular

**Character 47**. Slender anterior process of the frontal intruding between the prefrontal and the nasal:

0: absent

1: present

**Character 48**. Jugal-lachrymal relationship:

0: lachrymal overlapping lateral surface of jugal or abutting it dorsally

1: jugal overlapping lachrymal laterally

**Character 49**. Shape of the suborbital region of the jugal:

0: an anteroposteriorly elongate bar

1: or an anteroposteriorly shortened plate

**Character 50**. Jugal contribution to the antorbital fenestra:

0: absent

1: or present

**Character 51**. Dorsal process of the anterior jugal:

0: present

1: absent

**Character 52**. Ratio of the minimum depth of the jugal below the orbit to the distance between the rostral end of the jugal and the rostroventral corner of the infratemporal fenestra:

0: less than

1: or greater than 0.2

**Character 53**. Transverse width of the ventral ramus of the postorbital:

0: less than

1: or greater than, its rostrocaudal width at midshaft

**Character 54**. Shape of the dorsal margin of postorbital in lateral view:

0: straight to gently curved

1: or with a distinct embayment between the anterior and posterior dorsal processes

**Character 55**. Height of the postorbital rim of the orbit:

0: flush with the posterior lateral process of the postorbital

1: or raised so that it projects laterally to the posterior dorsal process

**Character 56**. Postfrontal bone:

0: present

1: absent

**Character 57**. Position of the rostral margin of the infratemporal fenestra :

0: behind the orbit

1: extends under the rear half of the orbit

2: or extends as far forward as the midlength of the orbit

**Character 58**. Frontal contribution to the supratemporal fenestra:

0: present

1: absent

**Character 59**. Orientation of the long axis of the supratemporal fenestra:

0: longitudinal

1: transverse

**Character 60**. Medial margin of supratemporal fossa:

0: simple smooth curve

1: or with a projection at the frontal ⁄ postorbital-parietal suture producing a scalloped margin

**Character 61**. Length of the quadratojugal ramus of the squamosal relative to the width at its base:

0: less than

1: greater than four times its width

**Character 62**. Proportion of infratemporal fenestra bordered by squamosal:

0: more than

1: or less than 0.5 of the depth of the infratemporal fenestra

**Character 63**. Squamosal-quadratojugal contact:

0: present

1: absent

**Character 64**. Angle of divergence between jugal and squamosal rami of quadratojugal:

0: close to 90 degrees

1: close to parallel

**Character 65**. Length of jugal ramus of quadratojugal:

0: no longer than

1: or longer than the squamosal ramus

**Character 66**. Shape of the rostral end of the jugal ramus of the quadratojugal:

0: tapered

1: dorsoventrally expanded

**Character 67**. Relationship of quadratojugal to jugal:

0: jugal overlaps the lateral surface of the quadratojugal

1: quadratojugal overlaps the lateral surface of the jugal

2: or quadratojugal sutures along the ventrolateral margin of the jugal

**Character 68**. Position of the quadrate foramen:

0: on the quadrate-quadratojugal suture

1: deeply incised into, and partly encircled by, the quadrate

2: or on the quadrate-squamosal suture, just below the quadrate head

**Character 69**. Shape of posterolateral margin of quadrate:

0: sloping anterolaterally from posteromedial ridge

1: everted posteriorly creating a posteriorly facing fossa

2: posterior fossa deeply excavated, invading quadrate body

**Character 70**. Exposure of the lateral surface of the quadrate head:

0: absent, covered by lateral sheet of the squamosal

1: or present

**Character 71**. Proportion of the length of the quadrate that is occupied by the pterygoid wing:

0: at least 70 per cent

1: or greater than 70 per cent

**Character 72**. Depth of the occipital wing of the parietal:

0: less than

1: more than 1.5 times the depth of the foramen magnum

**Character 73**. Position of foramina for mid-cerebral vein on occiput:

0: between supraoccipital and parietal

1: or on the supraoccipital

**Character 74**. Postparietal fenestra between supraoccipital and parietals:

0: absent

1: present

**Character 75**. Shape of the supraoccipital:

0: diamond-shaped, at least as high as wide

1: or semilunate and wider than high

**Character 76**. Orientation of the supraoccipital plate:

0: erect to gently sloping

1: or strongly sloping forward so that the dorsal tip lies level with the basipterygoid processes

**Character 77**. Orientation of the paroccipital processes in occipital view:

0: slightly dorsolaterally directed to horizontal

1: or ventrolaterally directed

**Character 78**. Orientation of the paroccipital processes in dorsal view:

0: posterolateral forming a V-shaped occiput

1: or lateral forming a flat occiput

**Character 79**. Size of the post-temporal fenestra:

0: large fenestra

1: a small hole that is much less than half the depth of the paroccipital process

**Character 80**. Exit of the mid-cerebral vein:

0: through trigeminal foramen

1: or through a separate foramen anterodorsal to trigeminal foramen

**Character 81**. Shape of the floor of the braincase in lateral view:

0: relatively straight with the basal tuberae, basipterygoid processes and parasphenoid rostrum roughly aligned

1: bent with the basipterygoid processes and the parasphenoid rostrum below the level of the basioccipital condyle and the basal tuberae

2: or bent with the basal tuberae lowered below the level of the basioccipital and the parasphenoid rostrum raised above it

**Character 82**. Shape of basal tuberae:

0: knob-like, with basisphenoidal component rostral to asioccipital component

1: or forming a transverse ridge with the basisphenoidal component lateral to the basioccipital component

**Character 83**. Length of the basipterygoid processes (from the top of the parasphenoid to the tip of the process):

0: less than

1: or greater than, the height of the braincase (from the top of the parasphenoid to the top of the supraoccipital)

**Character 84**. Ridge formed along the junction of the parabasisphenoid and the basioccipital, between the basal tuberae:

0: present with a smooth rostral face

1: present with a median fossa on the rostral face

2: or absent with the basal tuberae being separated by a deep caudally opening U-shaped fossa

**Character 85**. Deep septum spanning the interbasipterygoid space:

0: absent

1: present

**Character 86**. Dorsoventral depth of the parasphenoid rostrum:

0: much less than

1: or about equal to the transverse width

**Character 87**. Shape of jugal process of ectopterygoid:

0: gently curved

1: or strongly recurved and hook-like

**Character 88**. Pneumatic fossa on the ventral surface of the ectopterygoid:

0: present

1: absent

**Character 89**. Relationship of the ectopterygoid to the pterygoid (Sereno et al. 1993):

0: ectopterygoid overlapping the ventral

1: or dorsal surface of the pterygoid

**Character 90**. Position of the maxillary articular surface of the palatine:

0: along the lateral margin of the bone

1: or at the end of a narrow anterolateral process due to the absence of the posterolateral process

**Character 91**. Centrally located tubercle on the ventral surface of palatine:

0: absent

1: present

**Character 92**. Medial process of the pterygoid forming a hook around the basipterygoid process :

0: absent

1: flat and blunt-ended

2: or bent upward and pointed

**Character 93**. Length of the vomers:

0: less than

1: or more than 0.25 of the total skull length

**Character 94**. Position of jaw joint:

0: no lower than the level of the dorsal margin of the dentary

1: or depressed well below this level

**Character 95**. Shape of upper jaws in ventral view:

0: narrow with an acute rostral apex

1: or broad and U-shaped

**Character 96**. Length of the external mandibular fenestra:

0: more than

1: or less than 0.1 of the length of the mandible

**Character 97**. Caudal end of dentary tooth row medially inset with a thick lateral ridge on the dentary forming a buccal emargination:

0: absent

1: present

**Character 98**. Height : length ratio of the dentary:

0: less than

1: or greater than 0.2

**Character 99**. Orientation of the symphyseal end of the dentary:

0: in line with the long axis of the dentary

1: or strongly curved ventrally

**Character 100**. Position of first dentary tooth:

0: adjacent to symphysis

1: or inset one tooth’s width from the symphysis

**Character 101**. Dorsoventral expansion at the symphyseal end of the dentary:

0: absent

1: present

**Character 102**. Splenial foramen :

0: absent

1: present and enclosed

2: or present and open anteriorly

**Character 103**. Splenial-angular joint:

0: flattened sutured contact

1: synovial joint surface between tongue-like process of angular fitting in groove of the splenial

**Character 104**. A stout, triangular, medial process of the articular, behind the glenoid:

0: present

1: absent

**Character 105**. Length of the retroarticular process:

0: less than

1: or greater than than the depth of the mandible below the glenoid

**Character 106**. Strong medial embayment behind glenoid of the articular in dorsal view:

0: absent

1: or present

**Character 107**. Number of premaxillary teeth (Galton 1990):

0: four

1: or more than four

**Character 108**. Number of dentary teeth (in adults):

0: less than 18

1: or 18 or more

**Character 109**. Arrangement of teeth within the jaws:

0: linearly placed, crowns not overlapping

1: or imbricated with distal side of tooth overlapping mesial side of the succeeding tooth

**Character 110**. Orientation of the maxillary tooth crowns:

0: erect

1: or procumbent

**Character 111**. Orientation of the dentary tooth crowns:

0: erect

1: or procumbent

**Character 112**. Teeth with basally constricted crowns:

0: absent

1: present

**Character 113**. Tooth–tooth occlusal wear facets:

0: absent

1: present

**Character 114**. Mesial and distal serrations of the teeth:

0: fine and set at right angles to the margin of the tooth

1: coarse and angled upwards at an angle of 45 degrees to the margin of the tooth

**Character 115**. Distribution of serrations on the maxillary and dentary teeth:

0: present on both the mesial and distal carinae

1: absent on the posterior carinae

2: absent on both carinae

**Character 116**. Long axis of the tooth crowns distally recurved:

0: present

1: absent

**Character 117**. Texture of the enamel surface:

0: entirely smooth

1: finely wrinkled in some patches

2: extensively and coarsely wrinkled

**Character 118**. Lingual concavities of the teeth:

0: absent

1: present

**Character 119**. Longitudinal labial grooves on the teeth:

0: absent

1: present

**Character 120**. Distribution of the serrations along the mesial and distal carinae of the tooth:

0: extend along most of the length of the crown

1: are restricted to the upper half of the crown

**Character 121**. Number of cervical vertebrae:

0: eight or fewer

1: 9–10

2: 12–13

3: more than 13

**Character 122**. Shallow, dorsally facing fossa on the atlantal neurapophysis bordered by a dorsally everted lateral margin:

0: absent

1: present

**Character 123**. Width of axial intercentrum:

0: less than

1: greater than width of axial centrum.

**Character 124**. Position of axial prezygapophyses:

0: on the anterolateral surface of the neural arch

1: mounted on anteriorly projecting pedicels

**Character 125**. Posterior margin of the axial postzygapophyses:

0: Overhang the axial centrum

1: are flush with the caudal face of the axial centrum

**Character 126**. Length of the axial centrum:

0: less than

1: at least three times the height of the centrum

**Character 127**. Length of the anterior cervical centra (cervicals 3–5):

0: No more than

1: greater than the length of the axial centrum

**Character 128**. Length of middle to posterior cervical centra (cervicals 6–8):

0: no more than

1: greater than the length of the axial centrum

**Character 129**. Dorsal excavation of the cervical parapophyses:

0: absent

1: present

**Character 130**. Lateral compression of the anterior cervical vertebrae:

0: centra are no higher than they are wide

1: are approximately 1.25 times higher than wide

**Character 131**. Relative elongation of the anterior cervical centra (cervicals 3–5) :

0: lengths of the centra are less than 2.5 times the height of their anterior faces

1: lengths are 2.5–4 times the height of their anterior faces

2: the length of at least cervical 4 or 5 exceeds 4 times the anterior centrum height

**Character 132**. Ventral keels on cranial cervical centra:

0: present

1: absent

**Character 133**. 133. Height of the mid cervical neural arches:

0: no more than

1: greater than height of the posterior centrum face

**Character 134**. Cervical epipophyses on the dorsal surface of the postzygapophyses:

0: absent

1: present on at least some cervical vertebrae

**Character** 135. Caudal ends of cranial, postaxial epipophyses:

0: with a free pointed tip

1: joined to the postzygapophysis along their entire length

**Character** 136. Shape of the epipophyses:

0: tall ridges

1: flattened, horizontal plates

**Caracter 137**. Epipophyses overhanging the rear margin of the postzygapophyses:

0: absent

1: present in at least some postaxial cervical vertebrae

**Character 138**. Anterior spur-like projections on mid-cervical neural spines:

0: ausente

1: presente

**Character 139**. Shape of mid-cervical neural spines:

0: less than

1: at least twice as long as high.

**Character 140**. Shape of cervical rib shafts (Sereno 1999):

0: short and posteroventrally directed

1: longer than the length of their centra and extending parallel to cervical column

**Character 141**. Position of the base of the cervical rib shaft (Wilson and Sereno 1998):

0: level with, or higher than the ventral margin of the cervical centrum

1: or located below the ventral margin due to a ventrally extended parapophysis

**Character 142**. Postzygodiapophyseal lamina in cervical neural arches 4–8 (Yates 2003b):

0: present

1: absent

**Character 143**. Laminae of the cervical neural arches 4–8 (Wilson and Sereno 1998):

0: well-developed tall laminae

1: or weakly developed low ridges

**Character 144**. Shape of anterior centrum face in cervical centra (modified from Gauthier 1986. Ordenado):

0: concave

1: flat

2: convex

**Character 145**. Ventral surface of the centra in the cervicodorsal transition (Rauhut 2003a):

0: transversely rounded

1: with longitudinal keels

**Character 146**. Number of vertebrae between cervicodorsal transition and primordial sacral vertebrae:

0: 15–16

1: no more than 14

**Character 147**. Lateral surfaces of the dorsal centra :

0: with at most vague, shallow depressions

1: with deep fossae that approach the midline

2: or with invasive, sharp-rimmed pleurocoels

**Character 148**. Oblique ridge dividing pleural fossa of cervical vertebrae:

0: absent

1: present

**Character 149**. Laterally expanded tables at the midlength of the dorsal surface of the neural spines :

0: absent in all vertebrae

1: present on the pectoral vertebrae

2: present on the pectoral and cervical vertebrae

3: present and greatly expanded on the pectoral and cervical vertebrae (NEW STATE)

**Character 150**. Dorsal centra :

0: entirely amphicoelous to amphiplatyan

1: first two dorsals are opisthocoelous

2: cranial half of dorsal column is opisthocoelous

**Character 151**. Shape of the posterior dorsal centra:

0: relatively elongated for their size

1: strongly axially compressed for their size

**Character 152**. Laminae bounding triangular infradiapophyseal fossae (chonae) on dorsal neural arches:

0: absent

1: present

**Character 153**. Location of parapophysis in first two dorsals:

0: at the anterior end of the centrum

1: or located at the mid-length of the centrum, within the middle chonos

**Character 154**. Parapophyses of the dorsal column completely shift from the centrum to the neural arch:

0: anterior

1: posterior to the thirteenth presacral vertebra

**Character 155**. Orientation of the transverse processes of the dorsal vertebrae:

0: most horizontally directed

1: all upwardly directed

**Character 156**. Contribution of the paradiapophyseal lamina to the margin of the anterior chonos in mid-dorsal vertebrae:

0: present

1: prevented by high placement of parapophysis

**Character 157**. Hyposphenes in the dorsal vertebrae :

0: absent

1: present but less than the height of the neural canal

2: present and equal to the height of the neural canal

**Character 158**. Prezygodiapophyseal lamina and associated anterior triangular fossa (chonos):

0: present on all dorsals

1: absent in mid-dorsals

**Character 159**. Anterior centroparapophyseal lamina in dorsal vertebrae:

0: absent

1: present

**Character 160**. Prezygoparapophyseal lamina in dorsal vertebrae:

0: absent

1: present

**Character 161**. Accessory lamina dividing posterior chonos from postzygapophysis:

0: absent

1: present

**Character 162**. Lateral pneumatic fenestra in middle chonos of middle and posterior dorsal vertebrae opening into neural cavity:

0: absent

1: present

**Character 163**. Separation of lateral surfaces of anterior dorsal neural arches under transverse processes:

0: widely spaced

1: only separated by a thin midline septum

**Character 164**. Height of dorsal neural arches, from neurocentral suture to level of zygapophyseal facets:

0: much less than

1: subequal to or greater than height of centrum

**Character 165**. Form of anterior surface of neural arch (Bonaparte 1999):

0: simple centroprezygopophyseal ridge

1: broad anteriorly facing surface bounded laterally by centroprezygopophyseal lamina

**Character 166**. Shape of posterior dorsal neural canal (Wilson and Sereno 1998):

0: subcircular

1: slit-shaped

**Character 167**. Height of middle dorsal neural spines :

0: less than the length of the base

1: higher than the length of the base but less than 1.5 times the length of the base

2: or greater than 1.5 times the length of the base

**Character 168**. Shape of anterior dorsal neural spines:

0: lateral margins parallel in anterior view

1: transversely expanding towards dorsal end

2: greatly expanded dorsally (NEW STATE)

**Character 169**. Cross-sectional shape of dorsal neural spines:

0: transversely compressed

1: broad and triangular

2: square-shaped in posterior vertebrae

**Character 170**. Spinodiapophyseal lamina on dorsal vertebrae:

0: absent

1: present and separated from spinopostzygapophyseal lamina

2: present and joining spinopostzygapophyseal lamina to create a composite posterolateral spinal lamina

**Character 171**. Well-developed, sheet-like suprapostzygapophyseal laminae:

0: absent

1: present on at least the caudal dorsal vertebrae

**Character 172**. Shape of the spinopostzygapophyseal lamina in middle and posterior dorsal vertebrae:

0: singular

1: bifurcated at its distal end

**Character 173**. Shape of posterior margin of middle dorsal neural spines in lateral view:

0: approximately straight

1: concave with a projecting posterodorsal corner

**Character 174**. Transversely expanded plate-like summits of posterior dorsal neural spines:

0: absent

1: present

**Character 175**. Last presacral rib:

0: free

1: fused to vertebra

**Character 176**. Sacral rib much narrower than the transverse process of the first primordial sacral vertebra (and dorsosacral if present) in dorsal view:

0: absent

1: present

**Character 177**. Number of dorsosacral vertebrae (modified from Gauthier 1986). Ordered:

0: none

1: one

2: two

**Character 178**. Caudosacral vertebra:

0: absent

1: present

**Character 179**. Shape of the iliac articular facets of the first primordial sacral rib:

0: singular

1: divided into dorsal and ventral facets separated by a non-articulating gap

**Character 180**. Depth of the iliac articular surface of the primordial sacrals:

0: less than

1: greater than 0.75 of the depth of the ilium

**Character 181**. Sacral ribs contributing to the rim of the acetabulum:

0: absent

1: present

**Character 182**. Posterior and anterior expansion of the transverse processes of the first and second primordial sacral vertebrae, respectively, partly roofing the intercostal space:

0: absent

1: present

**Character 183**. Length of first caudal centrum:

0: greater than

1: less than its height

**Character 184**. Length of base of the proximal caudal neural spines:

0: less than

1: greater than half the length of the neural arch

**Character 185.** Position of postzygapophyses in proximal caudal vertebrae:

0: protruding with an interpostzygapophyseal notch visible in dorsal view

1: placed on either side of the caudal end of the base of the neural spine without any interpostzygapophyseal notch.

**Character 186**. A hyposphenal ridge on caudal vertebrae:

0: absent

1: present

**Character 187**. Depth of the bases of the proximal caudal transverse processes:

0: shallow, restricted to the neural arches

1: deep, extending from the centrum to the neural arch

**Character 188**. Position of last caudal vertebra with a protruding transverse process

0: distal

1: proximal to caudal 16

**Character 189**. Orientation of posterior margin of proximal caudal neural spines:

0: sloping posterodorsally

1: vertical

**Character 190**. Longitudinal ventral sulcus on proximal and middle caudal vertebrae:

0: present

1: absent

**Character 191**. Length of midcaudal centra:

0: greater than

1: less than twice the height of their anterior faces

**Character 192**. Cross-sectional shape of the distal caudal centra:

0: oval with rounded lateral and ventral sides

1: square-shaped with flattened lateral and ventral sides

**Character 193**. Length of distal caudal prezygapophysis:

0: short, not overlapping the preceding centrum by more than a quarter

1: long and overlapping the preceding the centrum by more than a quarter

**Character 194**. Shape of the terminal caudal vertebrae:

0: unfused, size decreasing toward tip

1: expanded and fused to form a club-shaped tail

**Character 195**. Length of the longest chevron:

0: is less than

1: greater than twice the length of the preceding centrum

**Character 196**. Anteroventral process on distal chevrons:

0: absent

1: present

**Character 197**. Mid-caudal chevrons with a ventral slit:

0: absent

1: present

**Character 198**. Longitudinal ridge on the dorsal surface of the sterna plate:

0: absent

1: present

**Character 199**. Craniocaudal length of the acromion process of the scapula:

0: less than

1: greater than 1.5 times the minimum width of the scapula blade

**Character 200**. Minimum width of the scapula (Gauthier 1986):

0: less than

1: greater than 20 per cent of its length

**Character 201**. Caudal margin of the acromion process of the scapula (modified from Novas 1992):

0: rises from the blade at angle that is less than

1: greater than 65 degrees from the long axis of the scapula, at its steepest point

**Character 202**. Width of dorsal expansion of the scapula (Pol and Powell 2007):

0: less than

1: equal to the width of the ventral end of the scapula

**Character 203**. Flat caudoventrally facing surface on the coracoid between glenoid and coracoid tubercle (Yates and Kitching 2003):

0: absent

1: present

**Character 204**. Coracoid tubercle (modified from Pérez-Moreno et al. 1994):

0: present

1: absent

**Character 205**. Length of the humerus (modified from Gauthier 1986) :

0: less than 55 per cent

1: 55–65 per cent

2: 65–70 per cent

3: more than 70 per cent of the length of the femur

**Character 206**. Shape of the deltopectoral crest (Gauthier 1986):

0: subtriangular

1: subrectangular

**Character 207**. Length of the deltopectoral crest of the humerus (modified from Sereno

et al. 1993). :

0: less than 30 per cent

1: 30–50 per cent

2: greater than 50 per cent of the length of the humerus

**Character 208**. Shape of the anterolateral margin of the deltopectoral crest of the humerus (Yates 2003b):

0: straight

1: strongly sinuous

**Character 209**. Rugose pit centrally located on the lateral surface of the deltopectoral crest:

0: absent

1: present

**Character 210**. Well-defined fossa on the distal flexor surface of the humerus (Yates and Kitching 2003):

0: present

1: absent

**Character 211**. Transverse width of the distal humerus (Langer 2004):

0: is less than

1: greater than 33 per cent of the length of the humerus

**Character 212**. Shape of the entepicondyle of the distal humerus:

0: rounded process

1: with a flat distomedially facing surface bounded by a sharp proximal margin

**Character 213**. Length of the radius (Langer 2004):

0: greater than

1: less than 80 per cent of the humerus

**Character 214**. Deep radial fossa, bounded by an anterolateral process, on proximal ulna (Wilson and Sereno 1998):

0: absent

1: present

**Character 215**. Olecranon process on proximal ulna (Wilson and Sereno 1998):

0: present

1: absent

**Character 216**. Maximum linear dimensions of the ulnare and radiale (Yates 2003b):

0: exceed that of at least one of the first three distal carpals

1: are less than any of the distal carpals

**Character 217**. Transverse width of the first distal carpal (Sereno 1999):

0: less than

1: greater than 120 per cent of the transverse width of the second distal carpal

**Character 218**. Sulcus across the medial end of the first distal carpal:

0: absent

1: present

**Character 219**. Lateral end of first distal carpal (Yates 2003b):

0: abuts

1: overlaps second distal carpal

**Character 220**. Second distal carpal (Yates and Kitching 2003):

0: does

1: does not completely cover the proximal end of the second metacarpal

**Character 221**. Ossification of the fifth distal carpal:

0: present

1: absent

**Character 222**. Length of the manus (modified from Sereno et al., 1993) :

0: less than 38 per cent

1: 38–45 per cent

2: greater than 45 per cent of the humerus + radius

**Character 223**. Shape of metacarpus (Wilson and Sereno 1998):

0: flattened to gently curved and spreading

1: a colonnade of subparallel metacarpals tightly curved into a U-shape

**Character 224**. Proximal width of first metacarpal (modified from Gauthier 1986):

0: less than

1: greater than the proximal width of the second metacarpal

**Character 225**. Minimum transverse shaft width of first metacarpal:

0: less than

1: greater than twice the minimum transverse shaft width of second metacarpal

**Character 226**. Proximal end of first metacarpal (Sereno 1999):

0: flush with other metacarpals

1: inset into the carpus

**Character 227**. Shape of the first metacarpal (modified from Sereno 1999). :

0: proximal width less than 65 per cent

1: 65–80 per cent

2: 80–100 per cent

3: greater than 100 per cent of its length

**Character 228**. Strong asymetry in the lateral and medial distal condyles of the first metacarpal (Gauthier 1986):

0: absent

1: present

**Character 229**. Deep distal extensor pits on the second and third metacarpals (Novas 1993):

0: absent

1: present

**Character 230**. Shape of the distal ends of second and third metacarpals:

0: subrectangular in distal view

1: trapezoidal with flexor rims of distal collateral ligament pits flaring beyond extensor rims

**Character 231**. Shape of the fifth metacarpal (Yates 2003b):

0: longer than wide at the proximal end with a flat proximal surface

1: close to as wide as it is long with a strongly convex proximal articulation surface

**Character 232**. Length of the fifth metacarpal (Upchurch 1998):

0: less than

1: greater than 75 per cent of the length of the third metacarpal

**Character 233**. Length of manual digit one (Yates 2003b):

0: less than

1: greater than the length of manual digit two

**Character 234**. Ventrolateral twisting of the transverse axis of the distal end of the first phalanx of manual digit one relative to its proximal end (Sereno 1999). Ordered:

0: absent

1: present but much less than 60 degrees or 60 degrees

**Character 235**. Length of the first phalanx of manual digit one (Gauthier 1986):

0: less than

1: greater than the length of the first metacarpal

**Character 236**. Shape of the proximal articular surface of the first phalanx of manual digit one (modified from Sereno 1999):

0: rounded

1: with an embayment on the medial side

**Character 237**. Shape of the first phalanx of manual digit one (Wilson 2002):

0: elongate and subcylindrical

1: strongly proximodistally compressed and wedge-shaped

**Character 238**. Length of the penultimate phalanx of manual digit two (Rauhut 2003a):

0: less than

1: greater than the length of the second metacarpal

**Character 239**. Length of the penultimate phalanx of manual digit three (Rauhut 2003a):

0: less than

1: greater than the length of the third metacarpal

**Character 240**. Shape of non-terminal phalanges of manual digits two and three (Yates 2003b):

0: longer than wide

1: as long as wide

**Character 241**. Shape of the unguals of manual digits two and three (Sereno et al. 1993):

0: straight

1: strongly curved with tips projecting well below flexor margin of proximal articular surface

**Character 242**. Length of the ungual of manual digit two (modified from Gauthier 1986). :

0: greater than the length of the ungual of manual digit one

1: 75–100 per cent of the ungual of manual digit one

2: less than 75 per cent of the ungual of manual digit one

3: the ungual of manual digit two is absent

**Characters 243**. Phalangeal formula of manual digits two and three (modified from Wilson and Sereno 1998):

0: three and four, respectively

1: with at least one phalanx missing from each digit

**Character 244**. Phalangeal formula of manual digits four and five (Gauthier 1986):

0: greater than

1: less than 2–0, respectively

**Character 245**. Strongly convex dorsal margin of the ilium (Gauthier 1986):

0: absent

1: present

**Character 246**. Cranial extent of preacetabular process of ilium (Yates 2003b):

0: does not

1: does project further forward than cranial end of the pubic peduncle

**Character 247**. Shape of the preacetabular process (modified from Sereno 1999):

0: blunt and rectangular

1: with a pointed, projecting cranioventral corner and a rounded dorsum

**Character 248**. Depth of the preacetabular process of the ilium (modified from Gauthier 1986):

0: much less than

1: subequal to the depth of the ilium above the acetabulum

**Character 249**. Length of preacetabular process of the ilium:

0: less than

1: greater than twice its depth

**Character 250**. Buttress between preacetabular process and the supraacetabular crest of the ilium (Gauthier 1986):

0: present

1: absent

**Character 251**. Medial wall of acetabulum (modified from Gauthier 1986). Ordered:

0: fully closing acetabulum with a triangular ventral process between the pubic and ischial peduncles

1: partially open acetabulum with a straight ventral margin between the peduncles

2: partially open acetabulum with a concave ventral margin between the peduncles

3: fully open acetabulum with medial ventral margin closely approximating lateral rim of acetabulum

**Character 252**. Length of the pubic peduncle of the ilium (Sereno 1999):

0: less than

1: greater than twice the craniocaudal width of its distal end

**Character 253**. Caudally projecting ‘heel’ at the distal end of the ischial peduncle:

0: absent

1: present

**Character 254**. Length of the ischial peduncle of the ilium (Upchurch et al. 2004) :

0: similar to pubic peduncle

1: much shorter than pubic peduncle

2: virtually absent so that the chord connecting the distal end of the pubic peduncle with the ischial articular surface contacts the postacetabular process

**Character 255**. Length of the postacetabular process of the ilium:

0: between 40 and 100 per cent of the distance between the pubic and ischial peduncles

1: less than 40 per cent of this distance

2: more than 100 per cent of this distance

**Character 256**. Well developed brevis fossa with sharp margins on the ventral surface of the postacetabular process of the ilium (Gauthier 1986):

0: absent

1: present

**Character 257**. Anterior end of ventrolateral ridge bounding brevis fossa:

0: not connected

1: joining to the supracetabular crest

**Character 258**. Shape of the caudal margin of the postacetabular process of the ilium:

0: rounded to bluntly pointed

1: square ended

2: with a pointed ventral corner and a rounded caudodorsal margin

**Character 259**. Width of the conjoined pubes:

0: less than

1: greater than 75 per cent of their length

**Character 260**. Pubic tubercle on the lateral surface of the proximal pubis (Yates 2003b):

0: present

1: absent

**Character 261**. Proximal anterior profile of pubis:

0: anterior margin of pubic apron smoothly confluent with anterior margin of iliac pedicel

1: iliac pedicel set anterior to the pubic apron creating a prominent inflection in the proximal anterior profile of the pubis

**Character 262**. Minimum transverse width of the pubic apron:

0: much more than

1: less than 40 per cent of the width across the iliac peduncles of the ilium

**Character 263**. Position of the obturator foramen of the pubis (Galton and Upchurch 2004):

0: at least partially occluded by the iliac pedicel

1: completely visible in anterior view

**Character 264**. Lateral margins of the pubic apron in anterior view (Yates and Kitching 2003):

0: straight

1: concave

**Character 265**. Orientation of distal third of the blades of the pubic apron (Langer 2004):

0: confluent with the proximal part of the pubic apron

1: twisted posterolaterally relative to proximal section so that the anterior surface turns to face laterally

**Character 266**. Orientation of the entire blades of the pubic apron:

0: transverse

1: twisted posteromedially

**Character 267**. Craniocaudal expansion of the distal pubis :

0: absent

1: less than 15 per cent

2: greater than 15 per cent of the length of the pubis

**Character 268**. Notch separating posteroventral end of the ischial obturator plate from the ischial shaft:

0: present

1: absent

**Character 269**. Elongate interischial fenestra:

0: absent

1: present

**Character 270**. Longitudinal dorsolateral sulcus on proximal ischium (Yates 2003b):

0: absent

1: present

**Character 271**. Shape of distal ischium:

0: broad and plate-like, not distinct from obturator region

1: with a discrete rod-like distal shaft

**Character 272**. Length of ischium:

0: less than

1: greater than that of the pubis

**Character 273**. Ischial component of acetabular rim (Galton and Upchurch 2004):

0: larger than

1: equal to the pubic component

**Character 274**. Shape of the transverse section of the ischial shaft (Sereno 1999):

0: ovoid to subrectangular

1: triangular

**Character 275**. Orientation of the long axes of the transverse section of the distal Ischia:

0: meet at an angle

1: are coplanar

**Character 276**. Depth of the transverse section of the ischial shaft:

0: much less than

1: at least as great as the transverse width of the section

**Character 277**. Distal ischial expansion:

0: absent

1: present

**Character 278**. Transverse width of the conjoined distal ischial expansions (Yates 2003b):

0: greater than

1: less than their sagittal depth

**Character 279**. Length of the hindlimb (Gauthier 1986):

0: greater than

1: less than the length of the trunk

**Character 280**. Longitudinal axis of the femur in lateral view :

0: strongly bent with an offset between the proximal and distal axes greater

than 15 degrees

1: weakly bent with an offset of less than 10 degrees

2: straight

**Character 281**. Shape of the cross-section of the mid-shaft of the femur:

0: subcircular

1: strongly elliptical with the long axis orientated mediolaterally

**Character 282**. Angle between the long axis of the femoral head and the transverse axis of the distal femur:

0: about 30 degrees

1: close to 0 degrees

**Character 283**. Shape of femoral head:

0: roughly rectangular in profile with a sharp medial distal corner

1: roughly hemispherical with no sharp medial distal corner

**Character 284**. Posterior proximal tubercle on femur:

0: well developed

1: indistinct to absent

**Character 285**. Shape of the lesser trochanter:

0: small rounded tubercle

1: proximodistally orientated, elongate ridge

2: absent

**Character 286**. Position of proximal tip of lesser trochanter (Galton and Upchurch 2004):

0: level with

1: distal to the femoral head

**Character 287**. Projection of the lesser trochanter:

0: just a scar upon the femoral surface

1: a raised process

**Character 288**. Transverse ridge extending laterally from the lesser trochanter:

0: absent

1: present

**Character 289**. Height of the lesser trochanter in cross section:

0: less than

1: at least as high as basal width

**Character 290**. Position of the lesser trochanter:

0: near the centre of the anterior face

1: close to the lateral margin of the femoral shaft in anterior view

**Character 291**. Visibility of the lesser trochanter in posterior view:

0: not visible

1: visible

**Character 292**. Height of the fourth trochanter:

0: tall crest

1: a low rugose ridge

**Character 293**. Position of the fourth trochanter along the length of the femur:

0: in the proximal half

1: straddling the midpoint

**Character 294**. Symmetry of the profile of the fourth trochanter of the femur (Langer 2004):

0: subsymmetrical without a sharp distal corner

1: asymmetrical with a steeper distal slope than the proximal slope and a distinct distal corner

2: symmetrical, with sharp proximal and distal corner

**Character 295**. Shape of the profile of the fourth trochanter of the femur:

0: rounded

1: subrectangular

**Character 296**. Position of fourth trochanter along the mediolateral axis of the femur:

0: centrally located

1: on the medial margin

**Character 297**. Extensor depression on anterior surface of the distal end of the femur:

0: absent

1: present

**Character 298**. Size of the medial condyle of the distal femur:

0: subequal

1: larger than the fibular + lateral condyles

**Character 299**. Tibia:femur length ratio :

0: greater than 1.0

1: between 0.6 and 1.0

2: less than 0.6

**Character 300**. Orientation of cnemial crest:

0: projects anteriorly to anterolaterally

1: projecting laterally

**Character 301**. Paramarginal ridge on lateral surface of cnemial crest:

0: absent

1: present

**Character 302**. Position of the tallest point of the cnemial crest:

0: close to the proximal end of the crest

1: about half-way along the length of the crest, creating an anterodorsally sloping proximal margin of the crest

**Character 303**. Proximal end of tibia with a flange of bone that contacts the fibula:

0: absent

1: present

**Character 304**. Position of the posterior end of the fibular condyle on the proximal articular surface tibia:

0: anterior to

1: level with the posterior margin of proximal articular surface

**Character 305**. Shape of the proximal articular surface of the tibia

0: ovoid, anteroposteriorly longer than transversely wide

1: subcircular and as wide transversely as anteroposteriorly long

**Character 306**. Transverse width of the distal tibia (Gauthier 1986):

0: subequal to

1: greater than its craniocaudal length

**Character 307**. Anteroposterior width of the lateral side of the distal articular surface of the tibia:

0: as wide

1: narrower than the anteroposterior width of the medial side

**Character 308**. Relationship of the posterolateral process of the distal end of the tibia with the fibula:

0: not flaring laterally and not making significant contact with the fibula

1: flaring laterally and backing the fibula

**Character 309**. Shape of the distal articular end of the tibia in distal view:

0: ovoid

1: subrectangular

**Character 310**. Shape of the anteromedial corner of the distal articular surface of the tibia:

0: forming a right angle

1: forming an acute angle

**Character 311**. Position of the lateral margin of descending caudoventral process of the distal end of the tibia:

0: protrudes laterally at least as far as

1: set well back from the craniolateral corner of the distal tibia

**Character 312**. A triangular rugose area on the medial side of the fibula:

0: absent

1: present

**Character 313**. Transverse width of the midshaft of the fibula :

0: greater than 0.75

1: between 0.5 and 0.75

2: less than 0.5 of the transverse width of the midshaft of the tibia

**Character 314**. Position of fibula trochanter:

0: on anterior surface of fibula

1: laterally facing

2: anteriorly facing but with strong lateral bulge

**Character 315**. Depth of the medial end of the astragalar body in cranial view:

0: roughly equal to the lateral end

1: or much shallower creating a wedge-shaped astragalar body

**Character 316.** Shape of the posteromedial margin of the astragalus in dorsal view:

0: forming a moderately sharp corner of a subrectangular astragalus

1: evenly rounded without formation of a caudomedial corner

**Character 317**. Dorsally facing horizontal shelf forming part of the fibular facet of the astragalus:

0: present

1: absent with a largely vertical fibular facet

**Character 318**. Pyramidal dorsal process on the posteromedial corner of the astragalus:

0: absent

1: present

**Character 319**. Shape of the ascending process of the astragalus:

0: anteroposteriorly deeper than transversely wide

1: transversely wider than anteroposteriorly deep

**Character 320**. Posterior extent of ascending process of the astragalus:

0: well anterior to

1: close to the posterior margin of the astragalus

**Character 321**. Sharp medial margin around the depression posterior to the ascending process of the astragalus:

0: absent

1: present

**Character 322**. Buttress dividing posterior fossa of astragalus and supporting ascending process:

0: absent

1: present

**Character 323**. Vascular foramina set in a fossa at the base of the ascending process of the astragalus:

0: present

1: absent

**Character 324**. Transverse width of the calcaneum (Yates and Kitching 2003):

0: greater than

1: less than 30 per cent of the transverse width of the astragalus

**Character 325**. Lateral surface of calcaneum:

0: simple

1: with a fossa

**Character 326**. Medial peg of calcaneum fitting into astragalus:

0: present, even if rudimentary

1: absent

**Character 327**. Calcaneal tuber:

0: large and well developed

1: highly reduced to absent

**Character 328**. Shape of posteromedial heel of distal tarsal four (lateral distal tarsal):

0: proximodistally deepest part of the bone

1: no deeper than the rest of the bone

**Character 329**. Shape of posteromedial process of distal tarsal four in proximal view:

0: rounded

1: pointed

**Character 330**. Ossified distal tarsals (Gauthier 1986):

0: present

1: absent

**Character 331**. Proximal width of the first metatarsal:

0: is less than

1: at least as great as the proximal width of the second metatarsal

**Character 332**. Orientation of proximal articular surface of metatarsal one:

0: horizontal

1: sloping proximolaterally relative to the long axis of the bone

**Character 333**. Orientation of the transverse axis of the distal end of metatarsal one:

0: horizontal

1: angled proximomedially

**Character 334**. Shape of the medial margin of the proximal surface of the second metatarsal:

0: straight

1: concave

**Character 335**. Shape of the lateral margin of the proximal surface of the second metatarsal:

0: straight

1: concave

**Character 336**. Length of the third metatarsal (Gauthier 1986):

0: greater than

1: less than 40 per cent of the length of the tibia

**Character 337**. Minimum transverse shaft diameters of third and fourth metatarsals:

0: greater than

1: less than 60 per cent of the minimum tansverse shaft diameter of the second metatarsal

**Character 338**. Transverse width of the proximal end of the fourth metatarsal:

0: less than

1: at least twice the anteroposterior depth of the proximal end

**Character 339**. Transverse width of the proximal end of the fifth metatarsal :

0: less than 25 per cent

1: between 30 and 49 per cent

2: greater than 50 per cent of the length of the fifth metatarsal

**Character 340**. Transverse width of distal articular surface of metatarsal four in distal view:

0: greater

1: less than anteroposterior depth

**Character 341**. Pedal digit five:

0: reduced, non-weight bearing

1: large (fifth metatarsal at least 70 per cent of fourth metatarsal), robust and weight bearing

**Character 342**. Length of non-terminal pedal phalanges :

0: all longer than wide

1: proximalmost phalanges longer than wide while more distal phalanges are as wide as long

2: all non-terminal phalanges are as wide, if not wider, than long

**Character 343**. Length of the first phalanx of pedal digit one (Yates and Kitching 2003):

0: greater than

1: less than the length of the ungual of pedal digit one

**Character 344**. Length of the ungual of pedal digit one:

0: less than at least some non-terminal phalanges

1: longer than all non-terminal phalanges

**Character 345**. Shape of the ungual of pedal digit one:

0: shallow, pointed, with convex sides and a broad ventral surface

1: deep, abruptly tapering, with flattened sides and a narrow ventral surface

**Character 346**. Shape of proximal articular surface of pedal unguals:

0: proximally facing, visible on medial and lateral sides

1: proximomedially facing and visible only in medial view, causing medial deflection of pedal unguals in articulation

**Character 347**. Penultimate phalanges of pedal digits two and three:

0: well-developed

1: reduced disc-shaped elements if they are ossified at all

**Character 348**. Shape of the unguals of pedal digits two and three:

0: dorsoventrally deep with a proximal articulating surface that is at least as deep as it is wide

1: dorsoventrally flattened with a proximal articulating surface that is wider than deep

**Character 349**. Length of the ungual of pedal digit two :

0: greater than

1: between 90 and 100 per cent of

2: less than 90 per cent of the length of the ungual of pedal digit one

**Character 350**. Size of the ungual of pedal digit three:

0: greater than

1: less than 85 per cent of the ungual of pedal digit two in all linear dimensions

**Character 351**. Number of phalanges in pedal digit four:

0: four

1: fewer than four

**Character 352**. Phalanges of pedal digit five (Gauthier 1986):

0: present

1: absent

**Character 353**. Femoral length :

0: less than 200 mm

1: between 200 and 399 mm

2: between 400 and 599 mm

3: between 600 and 799 mm

4: between 800 and 1000 mm (4),

5: greater than 1000 mm

**Characters 354-361 from Smith and Pol (2007)**

**Character 354**. Lateral extent of ventrolateral flange on plantar surface of MT II in proximal aspect:

0: similar in development to ventromedial flange

1: well−developed, extending further

**Character 355**. Distal articular surface of astragalus:

0: relatively flat or weakly convex

1: extremely convex and “roller−shaped”

**Character 356**. Distal surface of tibiofibular crest:

0: as deep anteroposteriorly as wide mediolaterally or deeper

1: wider mediolaterally than deep anteroposteriorly

**Character 357**. Well−developed facet on proximolateral corner of plantar ventrolateral flange ofMT II for articulation with medial distal tarsal:

0: absent

1: present

**Character 358**. Proximal outline of metatarsal III:

0: subtriangular with acute or rounded posterior border

1: subtrapezoidal, with posterior border broadly exposed in plantar view

**Character 359**. Angle formed by the anterior and anteromedial borders of metatarsal IV:

0: obtuse

1: right angle, or acute

**Character 360**. Well−developed tibiofibular crest on distal femur:

0: absent

1: present

**Character 361**. Shaft of metatarsal I:

0: closely appressed to metatarsal II throughout its length

1: only closely appressed proximally, with a space between metatarsals I and II distally

**Characters 362-363 from Apladetti et al. (2012)**

**Character 362.** Anterior fossa on the proximal region of the pubic apron:

0: absent

1: present

**Character 363**. Proximal end of the tibia with a transverse/anteroposterior length ratio:

0: narrow (ratio less than 0.7)

1: broad (more than 0.7)

**Character 364 new**

**Character 364.** Number of foramina in proximal portion of pubis:

0: one

1: two

**B) Modified matrix of Upchurch et al. (2007) used for phylogenetic analyses**

**Characters 1-292 from Upchurch et al. (2007**)

**Character 1**. Skull length:

0: > 50 per cent of femur length

1: < 50 per cent of femur length

**Character 2**. Caudal rim of external naris:

0: lies rostral to the rostral margin of the antorbital fenestra

1: lies caudal to the rostral margin of the antorbital fenestra

**Character 3**. External naris is:

0: < 50 per cent of maximum diameter of orbit

1: > 50 per cent of orbit diameter (1)

**Character 4**. External narial shape:

0: oval ⁄ elliptical

1: (0); subtriangular with right-angle at caudoventral corner

**Character 5**. External narial margin:

0: formed by the premaxilla and nasal alone, with a broad sutured contact between these processes

1: there is a point contact, or a gap between these elements filled by the maxilla

**Character 6**. Contact between premaxilla and nasal at the caudoventral margin of the external nares:

0: is a broad sutured contact or reduced to a point contact

1: is lost and replaced by a portion of the maxilla contributing to the margin

**Character 7**. Internarial bar:

0: wide (transverse width equals or exceeds rostrocaudal width)

1: laterally compressed (transverse width is less than rostrocaudal width)

**Character 8**. Dorsal (ascending) process of premaxilla:

0: curves dorsocaudally throughout its length

1: becomes horizontal in its caudal half

**Character 9**. Distal end of premaxillary dorsal (ascending) process:

0: tapers to a slender point

1: maintains or even increases its transverse width

**Character 10**. Caudolateral process of the premaxilla:

0: present

1: absent

**Character 11**. Profile of the rostrodorsal margin of the premaxilla in lateral view:

0: convex

1: has an inflexion at the base of the dorsal process

**Character 12**. Lateral plate on premaxilla, maxilla and dentary (supporting the bases of the tooth crowns labially):

0: absent

1: present

**Character 13**. Profile of the rostral end of the maxilla in lateral view:

0: slopes continuously towards the rostral tip

1: has an inflexion at the base of the ascending process which creates a rostral process with parallel dorsal and ventral margins

**Character 14**. Length of the rostral process of the maxilla:

0: less than its dorsoventral height

1: greater than its dorsoventral height

**Character 15**. Neurovascular foramina on the lateral surface of the maxilla:

0: numerous

1: reduced to 5–6 large foramina

**Character 16**. Size of neurovascular foramen at the caudal end of the lateral maxillary row:

0: not larger than the others

1: distinctly larger than the others

**Character 17**. Direction of the neurovascular foramen at the caudal end of the lateral maxillary row:

0: opens ventrally, laterally and rostrally

1: opens caudally

**Character 18**. Arrangement of lateral maxillary neurovascular foramina:

0: linear

1: irregular

**Character 19**. Lamina from the back edge of the maxillary ascending process creates an antorbital fossa:

0: present

1: absent

**Character 20**. Shape of the rostral margin of the antorbital fenestra in lateral view:

0: strongly concave, creating a narrow antorbital fossa

1: straight or slightly concave, creating a broad subtriangular antorbital fossa

**Character 21**. Lateral lamina (extending caudally from the ascending process of the maxilla, along the ventral rim of the antorbital fenestra):

0: absent

1: present

**Character 22**. Maxillary lateral lamina:

0: short (its rostrocaudal length is less than twice its height)

1: long (rostrocaudal length is more than twice its height)

**Character 23**. Dorsally open neurovascular canal on the floor of the antorbital fossa:

0: absent

1: present

**Character 24**. Maxilla–lachrymal contact above antorbital fenestra:

0: visible in lateral view

1: obscured in lateral view by an overhanging portion of the nasal

**Character 25**. Rostrocaudal length of the antorbital fossa:

0: greater than that of the orbit

1: less than that of the orbit

**Character 26**. ‘Shelf’-like area lateral to the external naris, extending onto the rostral end of the maxilla:

0: absent

1: present

**Character 27**. Rostrolateral process of the nasal:

0: has a basal width equal to that of the rostromedial process

1: is 50 per cent wider than the rostromedial process

**Character 28**. Median nasal depression:

0: absent

1: present

**Character 29**. Pointed caudolateral process of the nasal overlapping the lachrymal:

0: absent

1: present

**Character 30**. Dorsal exposure of the lachrymal:

0: present

1: absent

**Character 31**. Length of the dorsal process of the lachrymal compared to its ventral process:

0: > 0.5

1: < 0.5

**Character 32**. Extension of the antorbital fossa onto the ventral end of the lachrymal:

0: present

1: absent

**Character 33**. Prefrontal ventral process:

0: short, lies on the caudal surface of the lachrymal

1: long and extends down the medial side of the lachrymal

**Character 34**. Prefrontal : frontal length ratio:

0: < 0.75

1: > 0.75

**Character 35**. Jugal contribution to the antorbital fenestra:

0: absent

1: present

**Character 36**. Profile of the rostral end of the jugal in lateral view:

0: blunt

1: sharply pointed

**Character 37**. Ratio of the minimum depth of the jugal beneath the orbit to the length of the jugal from its rostral tip to the rostroventral corner of the infratemporal fenestra:

0: < 0.2

1: > 0.2

**Character 38**. Transverse width of the ventral process of the postorbital:

0: is less than its rostrocaudal width at mid-shaft

1: greater than its rostrocaudal width at mid-shaft

**Character 39**. Angle between the rostral and dorsal rami of the quadratojugal:

0: 90 degrees or more

1: 60 degrees or less

**Character 40**. Length of the rostral process of the quadratojugal:

0: less than or equal to that of the dorsal process

1: longer than the dorsal process

**Character 41**. Distal end of the quadratojugal rostral process:

0: tapers to a point

1: is dorsoventrally expanded

**Character 42**. Rounded ‘heel’-like caudoventral process of the quadratojugal:

0: present

1: absent

**Character 43**. Rostral margin of infratemporal fenestra:

0: lies caudal to the orbit

1: lies below the orbit

**Character 44**. Rostral margin of infratemporal fenestra:

0: lies below the midpoint of the orbit or more caudally

1: lies level with the rostral margin of the orbit

**Character 45**. Frontal contribution to dorsal margin of the orbit:

0: substantial

1: small or absent (as a result of the prefrontal and postfrontal approaching close to each other)

**Character 46**. Frontal exposure between prefrontal and nasal:

0: absent

1: present

**Character 47**. Frontal contribution to supratemporal fossa:

0: present

1: absent (excluded by a parietal-postorbital contact)

**Character 48**. Supratemporal fenestra:

0: obscured laterally by the postorbital bar

1: visible in lateral view because the postorbital bar lies substantially below the dorsal rim of the orbit

**Character 49**. Supratemporal fenestra:

0: longer rostrocaudally than wide transversely

1: wider transversely than rostrocaudally

**Character 50**. Parietals:

0: paired, sutured on the midline

1: fused on the midline

**Character 51**. Ventral process of the squamosal:

0: is tab-like

1: is straplike

**Character 52**.Quadratojugal process of the squamosal is:

0: less than four times as long as its basal width

1: more than four times as long as its basal width

**Character 53**. Squamosal-quadratojugal contact:

0: present

1: absent

**Character 54**. Position of the quadrate foramen:

0: deeply incised into, and partly encircled by, the quadrate

1: lies on the quadratequadratojugal suture

**Character 55**. Proportion of the length of the quadrate that is occupied by the pterygoid wing:

0: 0.7 or more

1: < 0.7

**Character 56**. Location of the foramen for the vena capitis media:

0: between the parietal, supraoccipital and exoccipital-opisthotic complex

1: fully enclosed by the supraoccipital

**Character 57**. Postparietal fenestra between the parietal and supraoccipital:

0: absent

1: present

**Character 58**. Supraoccipital:

0: is inclined at 75 degrees to the vertical so its rostral tip lies caudal to the basipterygoid processes

1: is inclined at 45 degrees so that its rostral tip lies above the basipterygoid processes

**Character 59**. Shape of the supraoccipital in caudal view:

0: diamond shaped, at least as high as wide

1: semilunate and wider than high

**Character 60**. Notch in prootic, above the opening for cranial nerve V, for a separate exit of the vena cerebralis medialis:

0: absent

1: present

**Character 61**. Deep transverse wall of bone between basipterygoid processes:

0: absent

1: present

**Character 62**. Shape of the floor of the braincase in lateral view:

0: relatively straight with the basal tubera, basipterygoid processes and parasphenoid rostrum roughly aligned

1: bent with the basipterygoid processes and parasphenoid rostrum below the level of the basioccipital condyle and the basal tubera

2: bent with the basal tubera below the basioccipital condyle and the parasphenoid rostrum above it

**Character 63**. Ridge formed along the junction of the parabasisphenoid and the basioccipital, between the basal tubera:

0: present with a smooth rostral face

1: present with a median fossa on the rostral face

2: absent, with the basal tubera separated by a deep fossa that opens caudally into a U-shaped fossa

**Character 64**. Ossification of the extremity of the basal tubera:

0: complete, so that the basioccipital and parabasisphenoid form a single rugose tuber

1: unossified, with the basioccipital forming a ventrally facing platform of unfinished bone that abuts a similarly unfinished caudally facing wall of the parabasisphenoid

**Character 65**. Shape of the basal tubera:

0: knob-like, with the basisphenoidal component rostral to the basioccipital component

1: a transverse ridge with the basisphenoidal component lying lateral to the basioccipital component

**Character 66**. Dorsoventral depth of the parasphenoid rostrum:

0: much less than its transverse width

1: approximately equal to its transverse width

**Character 67**. Length of the basipterygoid processes (from the top of the parabasisphenoid to the tip of the process):

0: less than the height of the braincase (from the top of the parabasisphenoid to the top of the supraoccipital)

1: greater than the height of the braincase

**Character 68**. Shape of the jugal process of the ectopterygoid:

0: gently curved

1: strongly recurved and ‘hook-like’

**Character 69**. Pneumatic fossa on the ventral surface of the ectopterygoid:

0: present

1: absent

**Character 70**. Position of the maxillary articulating surface of the palatine:

0: on the lateral margin of the bone

1: at the end of a narrow rostrolateral process

**Character 71**. Medial process of the pterygoid forming a ‘hook’ around the basipterygoid process:

0: absent

1: flat and blunt ended

2: pointed and bent upward

**Character 72**. Rostral end of dentary:

0: is narrower dorsoventrally than the caudal portion

1: is wider dorsoventrally, and more robust, than the caudal portion

**Character 73**. Dentary in lateral view:

0: is essentially straight or curves slightly upwards towards its rostral tip

1: curves ventrally towards its rostral tip

**Character 74**. Ridge on lateral surface of the dentary (possibly associated with a fleshy cheek in life):

0: absent

1: present

**Character 75**. Outline of lower jaw in dorsal view:

0: the rami meet each other at an acute angle

1: the rostral ends of the dentaries curve toward each other, creating a U-shaped outline

**Character 76**. Height : length ratio of the dentary:

0: < 0.2

1: > 0.2

**Character 77**. Long diameter of external mandibular fenestra:

0: is 10–15 per cent of mandible length

1: is 5 per cent of mandible length, or less

**Character 78**. External mandibular fenestra:

0: present (albeit in a reduced form)

1: closed

**Character 79**. Jaw articulation:

0: lies above the dorsal margin of the dentary

1: lies well below the dorsal margin of the dentary (at or close to the level of the ventral margin of the dentary)

**Character 80**. Retroarticular process length divided by its height at its base:

0: < 1.0

1: > 1.0 (approaching 2.0)

**Character 81**. Stout triangular medial process of the articular behind the glenoid:

0: present

1: absent

**Character 82**. Strong medial embayment behind the glenoid of the articular in dorsal view:

0: absent

1: present

**Character 83**. Number of teeth in the premaxilla:

0: 4

1: 5 or more

**Character 84**. Adjacent tooth crowns:

0: are aligned so they do not overlap in lateral view

1: are angled relative to the long axis of the jaw so tooth crowns appear to overlap in lateral view (each tooth has its mesial margin lying lingual to the distal margin of the crown immediately in front)

**Character 85**. Adjacent tooth crowns:

0: not in contact

1: in contact

**Character 86**. Tooth crown serrations:

0: project approximately perpendicular to the long axis of the crown

1: project at approximately 45 degrees to the long axis of the crown

2: serrations absent

**Character 87**. First dentary tooth:

0: lies at the extreme rostral end of the dentary

1: is inset a short distance from the rostral tip of the dentary

**Character 88**. Lingual surfaces of tooth crowns:

0: are convex or flat mesiodistally

1: have a concave area (either mildly concave or strongly concave)

**Character 89**. Lingual surfaces of tooth crowns:

0: are convex, nearly flat, or slightly concave mesiodistally

1: are deeply concave mesiodistally

**Character 90**. Prominent grooves near the distal margin of the labial surface of each tooth crown:

0: absent

1: present

**Character 91**. Prominent grooves near the mesial margin of the labial surface of each tooth crown:

0: absent

1: present

**Character 92**. Tooth crowns:

0: are all recurved

1: are lanceolate in at least the middle and caudal part of the tooth row

**Character 93**. Number of dentary teeth:

0: 18 or more

1: 17 or fewer

**Character 94**. Orientation of the dentary tooth crowns:

0: erect

1: procumbent

**Character 95**. Orientation of maxillary tooth crowns:

0: erect

1: procumbent

**Character 96**. Teeth with basally constricted crowns:

0: absent

1: present

**Character 97**. Tooth-tooth occlusion:

0: absent

1: present

**Character 98**. Tooth crown enamel:

0: smooth

1: wrinkled

**Character 99**. Tooth crown serrations:

0: distributed along the mesial and distal margins of the crown

1: restricted to the apical half of the crown

**Character 100**. Number of cervical vertebrae:

0: nine or fewer

1: ten or more

**Character 101**. Number of cervical vertebrae:

0: ten or fewer

1: 12 or more

**Character 102**. Shallow, dorsally facing fossa on the atlantal neurapophysis:

0: absent

1: present

**Character 103**. Axial postzygapophyses:

0: project caudally beyond end of centrum

1: are flush with end of centrum

**Character 104**. Length : height ratio of axis centrum:

0: < 3.0

1: 3.0 or more

**Character 105**. Length : height ratio of longest postaxial cervical centrum:

0: < 3.0

1: 3.0 or more

**Character 106**. Articulations between cervical centra:

0: are amphicoelous ⁄ amphiplatyan

1: are opisthocoelous (i.e. a cranial hemispherical convexity articulates with a corresponding concavity on the caudal surface of the preceding cervical)

**Character 107**. Dorsal excavation of the cervical parapophyses:

0: absent

1: present

**Character 108**. Strong lateral compression of cranial cervical vertebrae:

0: absent

1: present

**Character 109**. Ventral keels on caudal cervical centra:

0: present

1: absent

**Character 110**. Height of neural arches of mid-cervicals:

0: is less than centrum diameter

1: is equal to or greater than centrum diameter

**Character 111**. Height of mid-cervical neural arches:

0: is equal to, or less than, centrum diameter

1: is greater than centrum diameter

**Character 112**. Centrodiapophyseal lamina system:

0: is restricted to the dorsal vertebrae and caudal cervicals

1: is found on all presacral vertebrae

**Character 113**. Short cranially projected pedicels bearing axial prezygapophyses:

0: absent

1: present

**Character 114**. Epipophyses overhanging the rear margin of the postzygapophyses:

0: present

1: absent

**Character 115**. Caudal ends of cranial postaxial epipophyses:

0: with a free pointed tip

1: joined to the postzygapophyses along their entire length

**Character 116**. Length : height ratios for caudal dorsal centra:

0: < 1.0

1: > 1.0

**Character 117**. Lateral surfaces of dorsal centra:

0: with at most a shallow depression

1: strongly excavated (either deep fossae or true pleurocoels)

**Character 118**. Lateral surfaces of dorsal centra:

0: have a shallow or deep depression

1: have a deep pleurocoel that is sharp-edged and ramifies within the centrum

**Character 119**. Height of dorsal neural arches (i.e. from top of centrum to the level of the zygapophyses):

0: low (i.e. less than that of the centrum)

1: high (i.e. subequal to, or greater than, the height of the centrum)

**Character 120**. Cranial face of dorsal neural arch:

0: is flat or shallowly excavated

1: is deeply excavated, forming a large cavity above the neural canal

**Character 121**. Cranial dorsal transverse processes are directed:

0: laterally or slightly upwards

1: strongly dorsolaterally

**Character 122**. Laminae (prezygodiapophyseal) linking the prezygapophyses to the transverse processes on caudal dorsal vertebrae:

0: present

1: absent

**Character 123**. Prezygodiapophyseal lamina on cranial dorsals:

0: present

1: absent

**Character 124**. Spinodiapophyseal lamina on middle and caudal dorsal vertebrae:

0: absent

1: present

**Character 125**. Laterally expanded tables at the mid-length of the distal surface of the neural spines:

0: absent in all vertebrae

1: present on the cervical vertebrae

**Character 126**. Laterally expanded tables at mid-length on the distal surface of the neural spines:

0: absent, or present on cranial dorsal vertebrae alone

1: present on the cervical and cranial dorsal vertebrae

2: present and greatly expanded on the cervical and cranial dorsal vertebrae (NEW STATE)

**Character 127**. Dorsoventral height of the hyposphene:

0: much less than the dorsoventral height of the neural canal

1: equal to the dorsoventral height of the neural canal

**Character 128**. Ratio of the height of the neural spine to its craniocaudal basal width:

0: > 1.5

1: < 1.5

**Character 129**. Cross-sectional shape of dorsal neural spines:

0: narrow and elliptical

1: broad and triangular

**Character 130**. Composite lateral laminae on dorsal neural spines:

0: absent

1: present

**Character 131**. Spinoprezygapophyseal laminae:

0: absent

1: present on caudal or all dorsal vertebrae

**Character 132**. Spinoprezygapophyseal laminae:

0: absent, or present as low ridges on caudal dorsal vertebrae only

1: present on all dorsals as thin laminae

**Character 133**. Well-developed spinopostzygapophyseal laminae:

0: absent

1: present on at least the caudal dorsal vertebrae

**Character 134**. Well-developed spinopostzygapophyseal laminae:

0: absent or restricted to the caudal dorsal vertebrae

1: present on all dorsals

**Character 135**. Accessory infrapostzygapophyseal laminae on dorsal vertebrae:

0: present

1: absent

**Character 136**. Hindlimb : trunk length ratio:

0: is 1.0 lower

1: > 1.0

**Character 137**. Last presacral rib:

0: free

1: fused to vertebra

**Character 138**. Sacral number is:

0: two

1: three (via the addition of a caudosacral)

**Character 139**. Sacral number is:

0: two

1: three (via the addition of a dorsosacral)

**Character 140**. Sacral number:

0: four or fewer

1: five or more

**Character 141**. Sacricostal yoke (distal ends of sacral ribs fuse together):

0: absent

1: present

**Character 142**. Sacricostal yoke:

0: does not contribute to the dorsal rim of the acetabulum

1: contributes to the dorsal margin of the acetabulum

**Character 143**. Strong constriction between the sacral rib and transverse process of the first primordial sacral (and dorsosacral if present) in dorsal view:

0: absent

1: present

**Character 144**. Centrum length : height ratio :

0: > 1.0

1: 1.0–0.7

2: <0.7

**Character 145**. Length of mid-caudal centra compared with height of the cranial articular face:

0: > 2.0

1: < 2.0

**Character 146**. Longitudinal sulcus on the ventral surface of caudal centra:

0: absent

1: present

**Character 147**. Caudal ‘hyposphenal’ ridge:

0: absent

1: present

**Character 148**. Length of base of the proximal caudal neural spines:

0: greater than

1: less than half the length of the neural arch

**Character 149**. Position of postzygapophyses in proximal caudal vertebrae:

0: protruding with an interpostzygapophyseal notch visible in dorsal view

1: placed on either side of the base of the neural spine, without an interpostzygapophyseal notch

**Character 150**. Disappearance of caudal ribs occurs:

0: on caudal 20 or more distally

1: on caudals 14–16 or more cranially

**Character 151**. ‘Forked’ or ‘skid’-like middle and distal chevrons:

0: absent

1: present

**Character 152**. Mid-caudal chevrons with ventral midline slit:

0: absent

1: present

**Character 153**. Length of the longest chevron divided by the length of the centrum preceding it:

0: < 1.0

1: > 1.0

**Character 154**. Longitudinal ridge along the dorsal surface of the sternal plate:

0: absent

1: present

**Character 155**. Craniocaudal length of the acromion process of the scapula:

0: < 1.5

1: > 1.5 times the minimum width of the scapula blade

**Character 156**. Minimum width of the scapula divided by scapular length:

0: < 0.2

1: > 0.2

**Character 157**. Scapular blade in lateral view:

0: with strap-shaped midsection that has straight, subparallel margins

1: waisted with curved margins

**Character 158**. Caudal margin of the acromion process of the scapula rises at an angle to the blade that, at its steepest point is:

0: < 65 degrees

1: > 65 degrees

**Character 159**. Flat caudoventrally facing surface on the coracoid between the glenoid and the coracoid tubercle:

0: absent

1: present

**Character 160**. Coracoid tubercle:

0: present

1: absent

**Character 161**. Forelimb : hindlimb length ratio is:

0: < 0.60

1: 0.60 or more

**Character 132**. Forelimb : hindlimb length ratio is:

0: < 0.75

1: 0.75 or more

**Character 163**. Deltopectoral crest:

0: slants at 45–60 degrees to the transverse axis of the distal condyles

1: is perpendicular to the transverse axis of the distal condyles

**Character 164**. Deltopectoral crest:

0: terminates less than 50 per cent of humerus length from its proximal end

1: terminates at least 50 per cent of humerus length from the proximal end

**Character 165**. Deltopectoral crest is:

0: visible in caudal view (because the crest projects laterally beyond the rest of the shaft)

1: not visible in caudal view

**Character 166**. Deltopectoral crest:

0: prominent

1: reduced to a low ridge

**Character 167**. Craniolateral margin of the deltopectoral crest in cranial view:

0: straight

1: sigmoid

**Character 168**. Humerus : femur length ratio:

0: < 0.55

1: > 0.55

**Character 169**. Humerus : femur length ratio:

0: < 0.65

1: > 0.65

**Character 170**. Humerus : femur length ratio:

0: < 0.8

1: > 0.8

**Character 171**. Well-defined semicircular fossa on the distal flexor surface of the humerus:

0: present

1: absent

**Character 172**. Ratio of the transverse width of the distal end to total humerus length:

0: < 0.33

1: > 0.33

**Character 173**. Proximal end of ulna is:

0: subtriangular in outline and lacks a groove for the radius

1: triradiate because of a deep groove for reception of the radius

**Character 174**. Olecranon:

0: present as a prominent projection

1: almost completely absent

**Character 175**. Ratio of the lengths of the craniomedial and craniolateral processes of the proximal end of the ulna:

0: 1.0

1: > 1.0

**Character 176**. Radius : humerus length ratio:

0: < 0.80

1: 0.80 or more

**Character 177**. Distal condyle of radius:

0: is subcircular or oval in outline

1: is subrectangular with a flattened caudal margin for articulation with the ulna

**Character 178**. Proximal carpals:

0: present as ossifications

1: absent or fail to ossify

**Character 179**. Maximum linear dimensions of ulnare and radiale:

0: exceed those of at least one of the first three distal carpals

1: are less than any of the distal carpals

**Character 180**. First distal carpal:

0: is narrower transversely than metacarpal I

1: is subequal to, or greater than, the transverse width of metacarpal I

**Character 181**. Lateral end of first distal carpal:

0: abuts the second distal carpal

1: overlaps the second distal carpal

**Character 182**. Proximal end of first metacarpal:

0: flush with the proximal ends of other metacarpals

1: inset into the wrist

**Character 183**. Second distal carpal:

0: completely covers the proximal end of the second metacarpal

1: does not cover this surface completely

**Character 184**. Length of the manus divided by humerus + radius length:

0: > 0.45

1: < 0.45

**Character 185**. Length of manus divided by humerus + radius length:

0: >0.40

1: < 0.40

**Character 186**. Proximal width of metacarpal I divided by proximal width of metacarpal II:

0: < 1.0

1: > 1.0

**Character 187**. Proximal width of metacarpal I divided by metacarpal length:

0: < 0.65

1: > 0.65

**Character 188**. Proximal width of metacarpal I divided by metacarpal length:

0: < 0.8

1: > 0.8

**Character 189**. Proximal width of metacarpal I divided by metacarpal length:

0: < 1.0

1: > 1.0

**Character 190**. Strong asymmetry in the lateral and medial condyles of the first metacarpal:

0: absent

1: present

**Character 191**. Deep distal extensor pits on the distal end of metacarpals II and III:

0: present

1: absent

**Character 192**. Shape of metacarpal V:

0: longer than wide at the proximal end, with a flat proximal surface

1: nearly as wide as long with a strongly convex proximal surface

**Character 193**. Metacarpal V:

0: is reduced or absent

1: is large, robust and approximately 90 per cent of the length of the longest metacarpal

**Character 194**. Proximal ‘heel’ on first phalanx of manual digit I:

0: absent

1: present

**Character 195**. First phalanx of manual digit I:

0: has its proximal and distal articular surfaces with their axes in the same plane

1: has proximal and distal articular surfaces with their axes twisted so that they are at approximately 45 degrees to each other

**Character 196**. First phalanx of manual digit I:

0: has its proximal and distal articular surfaces with their axes in the same plane or twisted by no more than 45 degrees

1: has proximal and distal articular surfaces with their axes twisted so that they are at approximately 60 degrees to each other

**Character 197**. Length of manual digit I divided by length of manual digit II:

0: < 1.0

1: > 1.0

**Character 198**. Length of ungual on manual digit II divided by length of ungual on manual digit I:

0: > 1.0

1: < 1.0

**Character 199**. Length of ungual on manual digit II divided by length of ungual on manual digit I:

0: > 0.75

1: < 0.75

**Character 200**. Length of ungual on manual digit II divided by length of ungual on manual digit I:

0: > 0.75

1: ungual on manual digit II absent

**Character 201**. Shape of non-terminal manual phalanges:

0: longer than wide

1: as wide as long

**Character 202**. Phalangeal formula of manual digits IV and V:

0: less than 2–0

1: at least equal to or greater than 2–0, respectively

**Character 203**. Cranial process of the ilium:

0: lacks a scar

1: scar present

**Character 204**. Cranial process of the ilium:

0: terminates behind the level of the distal end of the pubic process

1: projects further cranially than the distal end of the pubic process

**Character 205**. Cranial process of the ilium:

0: is long and slender or very short

1: is relatively large and has a broad triangular outline in lateral view

**Character 206**. Depth of the cranial process of the ilium:

0: much less than the depth of the ilium above the acetabulum

1: is approximately the same depth as the ilium immediately above the acetabulum

**Character 207**. Length of cranial process of the ilium divided by its maximum depth:

0: < 2.0

1: > 2.0

**Character 208**. Area between the cranial process of the ilium and the pubic peduncle:

0: is gently curved in lateral view

1: is acute in lateral view

**Character 209**. Iliac portion of acetabulum:

0: partially backed by a wall of bone

1: almost completely open

**Character 210**. Concave area on the lateral surface of the ilium:

0: extends ventrally to a point close to the acetabular margin

1: is restricted to the dorsal half of the blade

**Character 211**. Dorsal margin of ilium:

0: smoothly convex (mildly or strongly) in lateral view

1: has a ‘step-like’ sigmoid profile in lateral view

**Character 212**. Lateral profile of the dorsal margin of the ilium:

0: is straight or sinusoidal

1: is strongly convex

**Character 213**. Length of the pubic peduncle of the ilium divided by the craniocaudal width of the peduncle:

0: < 2.0

1: > 2.0

**Character 214**. Ischial peduncle of the ilium:

0: is subequal in length to the pubic peduncle, giving the long axis of the iliac blade a nearly horizontal orientation

1: is reduced so that the long axis of the iliac blade slopes strongly craniodorsally in lateral view

**Character 215**. Caudally projecting ‘heel’ at the distal end of the ischial peduncle of the ilium:

0: absent

1: present

**Character 216**. Length of the postacetabular process of the ilium divided by total length of the ilium:

0: > 0.30

1: < 0.30

**Character 217**. Well-developed brevis fossa with sharp margins on the ventral surface of the postacetabular process of the ilium:

0: absent

1: present

**Character 218**. Shape of the caudal margin of the postacetabular process of the ilium:

0: rounded and bluntly pointed

1: square ended

**Character 219**. Pubic acetabular margin:

0: is approximately subequal in length to the ischial acetabular margin

1: is approximately half the length of the ischial acetabular margin

**Character 220**. Pubis in cranial view:

0: lateral margin of the ‘apron’ is straight or bows laterally

1: has a concave profile

**Character 221**. Ischium : pubis length ratio:

0: 0.90

1: 0.90 or more

**Character 222**. Pubic obturator foramen:

0: is absent or very small

1: is large, at least 50 per cent of acetabulum diameter

**Character 223**. Pubic obturator foramen:

0: partially obscured in cranial view of the pubis

1: completely visible in cranial view of the pubis

**Character 224**. Middle and distal portions of the pubis:

0: form a transverse sheet of bone that is twisted with respect to the proximal end

1: lies in approximately the same plane as the proximal end

**Character 225**. Pubic tubercle on the lateral surface of the proximal pubis:

0: present

1: absent

**Character 226**. Width of the conjoined pubes divided by their length:

0: <0.7

1: > 0.7

**Character 227**. Minimum transverse width of the pubic ‘apron’ divided by the width across the pubic peduncles of the ilium:

0: > 0.4

1: <0.4

**Character 228**. Craniocaudal length of the distal pubic expansion divided by pubis length:

0: < 0.15

1: > 0.15

**Character 229**. Notch separating caudoventral end of the ischial obturator plate from the ischial shaft:

0: present

1: absent

**Character 230**. Elongate interischial fenestra:

0: present

1: absent

**Character 231**. Long-axes of the distal ends of the ischia:

0: are set at an angle to each other

1: are co-planar

**Character 232**. Distal end of ischium:

0: is only slightly expanded relative to the rest of the shaft

1: is strongly expanded dorsoventrally (so that the thickness of the shaft appears to have doubled at the distal end in lateral view)

**Character 233**. Distal end of ischium:

0: maximum thickness is less than three times the minimum thickness

1: maximum thickness is at least three times minimum thickness

**Character 234**. Outline of the distal end of ischium:

0: rounded or flattened

1: subtriangular

**Character 235**. Proximal end of the lesser trochanter:

0: terminates below the femoral head

1: terminates level with the femoral head

**Character 236**. Lesser trochanter:

0: all of lateral edge lies medial to the lateral edge of femur

1: projects beyond the lateral edge of the femur so that it is visible in caudal view

**Character 237**. Lesser trochanter on the femur:

0: is well developed

1: is absent or greatly reduced

**Character 238**. Lesser trochanter:

0: is well developed or reduced to a still visible ridge

1: is completely absent

**Character 239**. Lesser trochanter:

0: is a ridge-like structure or reduced

1: is developed into a prominent sheet-like structure

**Character 240**. Fourth trochanter:

0: is a prominent plate-like structure

1: is reduced to a low ridge

**Character 241**. Fourth trochanter on the femoral shaft:

0: lies in the proximal half

1: lies over the mid-point

**Character 242**. Profile of the fourth trochanter in lateral ⁄ medial view:

0: rounded and symmetrical

1: asymmetrical with the distal margin steeper than the rounded dorsal margin

**Character 243**. Fourth trochanter of the femur:

0: lies centrally on the caudal surface

1: lies near or on the caudomedial margin

**Character 244**. Femoral head:

0: projects medially or ventromedially

1: projects dorsomedially

**Character 245**. Proximal end of femur in cranial or caudal view:

0: merges smoothly with the lateral margin of the shaft

1: meets the lateral margin at an abrupt angle (approximately 90 degrees)

**Character 246**. Femoral shaft:

0: has a sigmoid curve

1: is straight (in cranial or caudal view)

**Character 247**. The cranial face of the femur in lateral view:

0: is convex

1: is straight

**Character 248**. Horizontal cross-section through the femoral shaft:

0: is subcircular

1: is elliptical or subrectangular (with the transverse diameter wider than the craniocaudal diameter)

**Character 249**. Angle between the long-axis of the femoral head and the transverse axis of the distal end:

0: close to 30 degrees

1: close to 0 degrees

**Character 250**. Tibia : femur length ratio:

0: 1.0 or more

1: < 1.0

**Character 251**. Tibia : femur length ratio:

0: approximately 0.65 or higher

1: < 0.65

**Character 252**. Extensor depression on the distal end of the femur:

0: absent

1: present

**Character 253**. Cnemial crest on the tibia:

0: is directed cranially

1: is directed laterally

**Character 254**. Medial malleolus of the tibia:

0: extends caudoventrally to cover the astragalus in caudal view

1: is reduced, exposing the posterior fossa of the astragalus in caudal view

**Character 255**. Transverse width of the distal tibia:

0: subequal to its craniocaudal width

1: greater than its craniocaudal width

**Character 256**. Trigonal striated articular crest on medial surface of the proximal end of the fibula:

0: absent

1: present

**Character 257**. Muscle scar ⁄ trochanter on the lateral surface of fibula (at mid-length):

0: absent

1: present

**Character 258**. Ascending process of the astragalus:

0: extends dorsally in front of the distal end of the tibia

1: keys into the distal end surface of the tibia

**Character 259**. Depression and vascular foramina in front of the base of the astragalar ascending process:

0: present

1: absent

**Character 260**. Caudal fossa of the astragalus:

0: is undivided

1: is divided into lateral and medial portions by the presence of a ridge or crest that descends ventromedially from the apex of the ascending process

**Character 261**. Depth of the medial end of the astragalus in cranial view:

0: equal to the depth of the lateral end

1: much less than the lateral end, making the astragalus wedge-shaped

**Character 262**. Shape of the caudomedial margin of the astragalus in dorsal view:

0: forms a moderately sharp corner of a subrectangular outline

1: smoothly rounded without a caudomedial corner

**Character 263**. Dorsally facing horizontal shelf forming part of the fibular facet of the astragalus:

0: present

1: absent, so that the fibular facet faces laterally and is vertical

**Character 264**. A lateral horizontal groove on the calcaneum:

0: absent

1: present

**Character 265**. Transverse width of the calcaneum divided by the transverse width of the astragalus:

0: > 0.3

1: < 0.3

**Character 266**. Number of ossified distal tarsals:

0: two or more

1: none

**Character 267**. Metatarsal III length : tibia length ratio:

0: 0.4 or higher

1: < 0.4

**Character 268**. Metatarsal III length : tibia length:

0: > 0.3

1: < 0.3

**Character 269**. Metatarsal I length : width ratio:

0: > 1.5

1: < 1.5

**Character 270**. Proximal ends of metatarsals I and V:

0: are smaller in area than those of metatarsals II and IV

1: have areas equal to or larger than metatarsals II and IV

**Character 271**. Medial margin of the proximal end of metatarsal II:

0: straight or convex

1: concave

**Character 272**. Lateral margin of the proximal end of metatarsal II:

0: straight or convex

1: concave

**Character 273**. Metatarsals II and III have proximal width : length ratios of:

0: < 0.25

1: 0.25 or higher

**Character 274**. Metatarsal IV proximal end transverse width : dorsoventral height ratio:

0: < 2.0

1: > 2.0

**Character 275**. Metatarsal IV proximal end transverse width : dorsoventral height ratio:

0: < 3.0

1: approximately 3.0

**Character 276**. Minimum shaft widths of metatarsals III and IV divided by minimum shaft width of metatarsal II:

0: < 0.6

1: > 0.6

**Character 277**. Metatarsal V in dorsal (cranial) view:

0: has proximal and distal ends approximately subequal in width

1: has a transversely widened proximal end and narrowed distal end so that the metatarsal is ‘funnel’ or ‘paddle’-shaped

**Character 278**. Metatarsal V length:

0: < 50 per cent of metatarsal III length

1: > 50 per cent of metatarsal III length

**Character 279**. Proximal width : length ratio of metatarsal V:

0: < 0.25

1: > 0.25

**Character 280**. Proximal width : length ratio of metatarsal V:

0: < 0.5

1: >0.5

**Character 281**. At least some pedal phalanges, apart from unguals:

0: are longer proximodistally than their transverse widths

1: are wider transversely than their proximodistal lengths

**Character 282**. Number of phalanges on pedal digit IV:

0: four

1: fewer than four

**Character 283**. Phalanges of pedal digit V:

0: absent

1: present

**Character 284**. Ungual on pedal digit I:

0: is shorter than other pedal unguals

1: is longer than other pedal unguals

**Character 285**. Ungual on pedal digit I:

0: is shorter than other pedal phalanges

1: is subequal to, or longer than, all other pedal phalanges

**Character 286**. Ungual on pedal digit I:

0: shorter than metatarsal I

1: longer than metatarsal I

**Character 287**. Shape of ungual on pedal digit I:

0: shallow, pointed, with convex sides and broad ventral surface

1: deep, abruptly tapering, with flat sides and a narrow ventral surface

**Character 288**. Proximal ends of unguals on pedal digits II and III:

0: have subequal transverse and dorsoventral diameters (or are dorsoventrally compressed)

1: are transversely compressed

**Character 289**. Length of ungual on pedal digit II divided by length of ungual on pedal digit I:

0: > 1.0

1: < 1.0

**Character 290**. Length of ungual on pedal digit II divided by length of ungual on pedal digit I:

0: > 0.9

1: < 0.9

**Character 291**. Length of ungual on pedal digit III divided by length of ungual on pedal digit II:

0: > 0.85

1: < 0.85

**Character 292**. Pedal unguals:

0: directed forwards

1: deflected forwards and laterally, so that the articular surfaces of the unguals are only visible in lateral view

**Characters 293-300 from Smith and Pol (2007)**

**Character 293**. Lateral extent of ventrolateral flange on plantar surface of MT II in proximal aspect:

0: similar in development to ventromedial flange

1: well−developed, extending further

**Character 294**. Distal articular surface of astragalus:

0: relatively flat or weakly convex

1: extremely convex and “roller−shaped”

**Character 295**. Distal surface of tibiofibular crest:

0: as deep anteroposteriorly as wide mediolaterally or deeper

1: wider mediolaterally than deep anteroposteriorly

**Character 296**. Well−developed facet on proximolateral corner of plantar ventrolateral flange ofMT II for articulation with medial distal tarsal:

0: absent

1: present

**Character 297**. Proximal outline of metatarsal III:

0: subtriangular with acute or rounded posterior border

1: subtrapezoidal, with posterior border broadly exposed in plantar view

**Character 298**. Angle formed by the anterior and anteromedial borders of metatarsal IV:

0: obtuse

1: right angle, or acute

**Character 299**. Well−developed tibiofibular crest on distal femur:

0: absent

1: present

**Character 300**. Shaft of metatarsal I:

0: closely appressed to metatarsal II throughout its length

1: only closely appressed proximally, with a space between metatarsals I and II distally

**Characters 301-302 from Apaldetti et al. (2012)**

**Character 301.** Anterior fossa on the proximal end of the pubic apron:

0: absent

1: presence

**Character 302.** Proximal en of the tibia with a transverse/anteroposterior length ratio:

0: narrow (ratio less than 0.7)

1: broad (ratio more than 0.7

**Character 303 new**

**Character 303.** Number of foramina in proximal portion of pubis:

0: one

1: two

**C) Discussion on the changed character scores of Sarahsaurus in this analysis.**

*Sarahsaurus* was recovered as a basal sauropodomorph dinosaur in most of the phylogenetic analyses conducted by Rowe et al. (1), it was positioned within Sauropoda as the sister taxon to all other sauropods in the pruned analysis of the revised matrix from Upchurch et al (3). In the pruned analysis of the revised data set from Yates (2), *Sarahsaurus* was recovered as basal to all other sauropodomorphs except *Efraasia*, *Pantydraco*, *Thecodontosaurus*, and *Saturnalia*. In that tree, the branch length of *Sarahsaurus* was long and diagnosed by numerous homoplasies and reversals (1), which is often symptomatic of erroneous topology (Donoghue et al., 1989). Those authors concluded their study by mentioning that *Sarahsaurus* shared features with sauropods like “columnar hindlimbs,” suggesting that it may be a more derived sauropodomorph (1: 1050). However, in light of this redescription and its affects on our phylogenetic analyses, we conclude that *Sarahsaurus* is not a sauropod and is instead a massospondylid sauropodomorph. A non-comprehensive list of character state changes attributed to this study is found in Table 1.

Table 1- Select changes to character scores discussed above, taken from the revised data matrices used by the initial study [1].

|  | Yates [2] | | Upchurch et al. [3] | |
| --- | --- | --- | --- | --- |
| Skeletal focus of character | Character | Change | Character | Change |
| Contribution of frontal to supratemporal fenestra | 58 | 0→1 | - | - |
| Position of quadrate foramen | 68 | 0→1 | 54 | 1→0 |
| Shape of floor of braincase | 81 | 1→0 | 62 | n.a. |
| Presence of anterior centroparapophyseal lamina | 159 | 1→0 | - | - |
| Presence of prezygapophyseal lamina | 160 | 1→0 | - | - |
| Presence of accessory lamina | 161 | 1→0 | - | - |
| Shape of suprapostzygapophyseal lamina | 172 | 0→? | - | - |
| Ridge on sternal plate | 198 | 1→0 | 154 | 1→0 |
| Presence of radiale and ulnare | - | - | 178 | 0→1 |
| Maximum dimensions of radiale and ulnare | 216 | 1→? | 179 | 1→? |
| Shape of dorsal margin of ilium | 245 | 1→0 | - | - |
| Orientation of long axis of ilium | - | - | 214 | 1→0 |
| Buttress on preacetabular process of ilium | 250 | 0→1 | - | - |
| Presence of pubic tubercle | 260 | 0→1 | 225 | 0→1 |
| Presence of interischial fenestra | 269 | 1→0 | 230 | n.a. |
| Curvature of femur in lateral view | 280 | 2→0&1* | 247 | 1→0 |
| Curvature of femur in anterior view | - | - | 246 | 1→0 |
| Offset between femoral head and distal condyles | 282 | 1→0 | 249 | 1→0 |
| Size of anterior trochanter | 287 | 0→1 | 237 | 1→0 |
| Height of anterior trochanter | 289 | 1→0 | 238 | n.a. |
| Orientation of pedal unguals | - | - | 292 | ?→0 |
| Many of the characters in the pelvic girdle and hindlimb may have biased the results of the previous phylogenetic analysis in which *Sarahsaurus* was recovered as a basal sauropod dinosaur instead of an early sauropodomorph. *The holotype femora are crushed and display a different degree of curvature than the paratype femur (Fig. 36). | | | | |

Some of the character score changes for the skull are provided above in the discussion of MCZ 8893. Those changes include the lack of contribution of the frontal to the supratemporal fenestra, the position of the quadrate foramen through the body of the quadrate, and the horizontal alignment of the floor of the braincase in *Sarahsaurus*.

The anterior dorsal vertebrae of *Sarahsaurus* have laterally-expanded tables on the dorsal margin of the neural spine. Those tables are lost in sauropods and closely related taxa like *Melanorosaurus* and *Aardonyx*. In many sauropods, the transverse processes of the dorsal vertebrae point dorsolaterally, but they jut laterally in *Sarahsaurus*. The pre-sacral vertebrae of *Sarahsaurus* do not have the anterior centroparapophyseal, prezygaparapophyseal, and accessory laminae that are found in sauropods, even though they were reported previously [1]. Because *Sarahsaurus* also does not have the suprapostzygapophyseal lamina, the character describing the shape of that lamina should be changed from ‘0’ to ‘?’ for *Sarahsaurus*, because the original ‘0’ implies that that lamina is present (Table 4). The anterior and posterior centrodiapophyseal laminae are present in the dorsal vertebrae of *Sarahsaurus*, but they also are found on the last cervical vertebrae.

The number of sacral vertebrae needs to be recounted for all sauropodomorphs using consistent definitions of terms like ‘dorsosacral,’ ‘caudosacral,’ ‘primordial,’ and ‘insertion’ [100, 107-109]. However, *Sarahsaurus* and other early sauropodomorphs only have three sacral vertebrae, but skeletally mature sauropods tend to incorporate more than three vertebrae into the sacrum. Caudal vertebrae of sauropods lose their transverse processes between caudal vertebrae 14 to 16, but the transverse processes of *Sarahsaurus* disappear behind those vertebrae.

Unlike the basal sauropods *Cetiosaurus*, *Mamenchisaurus*, *Omeisaurus*, and *Shunosaurus*, *Sarahsaurus* does not have a longitudinal ridge on the exterior surface of the sternal plate. The radiale and ulnare are absent in *Sarahsaurus*, so the character describing the maximum linear dimensions of those bones should be scored as ‘?’ instead of ‘0’ (Table 2). Again, a ‘0’ represents an implied presence that is unjustifiable based upon the CT data from the articulated left manus of *Sarahsaurus* (Fig. 24).

Many of the characters that were found by Rowe et al. [1] to compare favorably to sauropods pertain to the pelvic girdle and hindlimb (Table 4). The revised matrix from Upchurch et al. [3] that was used in their pruned analysis was sensitive to the character scores in those anatomical regions, and once incomplete taxa were removed from the data set, *Sarahsaurus* was pulled up the tree and placed within Sauropoda.

First, the dorsal margin of the ilium is not strongly convex in *Sarahsaurus* like it is in sauropods, and its long axis is not inclined anterodorsally. Instead, the top of the ilium is only slightly convex, and its long axis points anteriorly. The shape and orientation of the ilium of *Sarahsaurus* is more similar to that of the early sauropodomorphs *Massospondylus*, *Plateosaurus*, *Adeopapposaurus*, and *Coloradisaurus*. Second, the pubic tubercle found in sauropods and many early sauropodomorphs is not present on the proximolateral margin of the pubis of *Sarahsaurus*. Third, the pubic blades of *Sarahsaurus* are not twisted posteromedially like they are in sauropods, and instead, the pubic aprons directed medially and meet along the midline. Fourth, the holotype femora of *Sarahsaurus* are crushed, but close inspection and referall to the uncrushed paratype femur confirms that the femur of *Sarahsaurus* was not straight like that of sauropods, but was instead slightly sigmoid in lateral profile. There is also a degree of offset between the long axes of the head of the femur and the distal condyles in *Sarahsaurus* that is not present in sauropods. Fifth, the anterior trochanter is well developed in *Sarahsaurus*. This feature is diminished and is not as prominent in sauropods. Sixth, the unguals of *Sarahsaurus*, and early sauropodomorphs in general, point anteriorly, unlike the unguals of sauropods that are inclind anteromedially. Recent reconstructions of the pedes of early sauropodomorph dinosaurs suggest that the metatarsals were not splayed mediolaterally [117-118].

**D) Modified matrix of McPhee and Choiniere (2017) with *Sarahsaurus aurifontanalis* rescored for this study. See McPhee and Choiniere (2017) for the derivations of the characters for this matrix.**

**Character 1**. Skull to femur ratio:

0: greater than 0.6

1: less than 0.6

**Character 2.** Lateral plates appressed to the labial side of the premaxillary:

0: absent

1: present

**Character 3.** Relative height of the rostrum at the posterior margin of the external naris:

0: more than 0.6 the height of the skull at the middle of the orbit

1: less than 0.6 the height of the skull at the middle of the orbit

**Character 4**. Foramen on the lateral surface of the premaxillary body:

0: absent

1: present

**Character 5**. Distal end of the dorsal premaxillary process:

0: tapered

1: transversely expanded

**Character 6.** Profile of premaxilla:

0: convex

1: with an inflection at the base of the dorsal process:

**Character 7**. Size and position of the posterolateral process of premaxilla:

0: large and lateral to the anterior process of the maxilla

1: small and medial to the anterior process of the maxilla

**Character 8**. Relationship between posterolateral process of the premaxilla and the anteroventral process of the nasal:

0: broad sutured contact

1: point contact

2: separated by maxilla

**Character 9**. Posteromedial process of the premaxilla:

0: absent

1: present

**Character 10**. Shape of the anteromedial process of the maxilla:

0: narrow, elongated and projecting anterior to lateral premaxilla-maxilla suture

1: short, broad and level with lateral premaxilla-maxilla suture

**Character 11**. Development of external narial fossa:

0: absent to weak

1: well developed with sharp posterior and anteroventral rims

**Character 12**. Development of narial fossa on the anterior ramus of the maxilla:

0: weak and orientated laterally to dorsolaterally

1: well developed and forming a horizontal shelf

**Character 13**. Size and position of subnarial foramen:

0: absent

1: small (no larger than adjacent maxillary neurovascular foramina) and positioned outside of narial fossa

2: large and on the rim of, or inside, the narial fossa

**Character 14**. Shape of subnarial foramen:

0: rounded

1: slot-shaped

**Character 15**. Maxillary contribution to the margin of the narial fossa:

0: absent

1: present

**Character 16**. Diameter of external naris:

0: less than 0.5 of the orbital diameter

1: greater than 0.5 of the orbital diameter

**Character 17**. Shape of the external naris (in adults):

0: rounded

1: subtriangular with an acute posteroventral corner

**Character 18**. Level of the anterior margin of the external naris:

0: anterior to the midlength of the premaxillary body

1: posterior to the midlength of the premaxillary body

**Character 19**. Level of the posterior margin of external naris:

0: anterior to, or level with the premaxilla-maxilla suture

1: posterior to the first maxillary alveolus

2: posterior to the midlength of the maxillary tooth row and the anterior margin of the antorbital fenestra

**Character 20**. Dorsal profile of the snout:

0: straight to gently convex

1: with a depression behind the naris

**Character 21**. Elongate median nasal depression:

0: absent

1: present

**Character 22**. Width of anteroventral process of nasal at its base:

0: less than the width of the anterodorsal process at its base

1: greater than the width of the anterodorsal process at its base

**Character 23**. Nasal relationship with dorsal margin of antorbital fossa:

0: not contributing to the margin of the antorbital fossa

1: lateral margin overhangs the antorbital fossa and forms its dorsal margin

2: overhang extensive, obscuring the dorsal lacrimal-maxilla contact in lateral view

**Character 24**. Pointed caudolateral process of the nasal overlapping the lacrimal:

0: absent

1: present

**Character 25**. Anterior profile of the maxilla:

0: slopes continuously towards the rostral tip

1: with a strong inflection at the base of the ascending ramus, creating a rostral ramus with parallel dorsal and ventral margins

**Character 26**. Length of rostral ramus of the maxilla:

0: less than its dorsoventral depth

1: greater than its dorsoventral depth

**Character 27**. Shape of the main body of the maxilla:

0: tapering posteriorly

1: dorsal and ventral margins parallel for most of their length

**Character 28**. Shape of the ascending ramus of the maxilla in lateral view:

0: tapering dorsally

1: with an anteroposterior expansion at the dorsal end

**Character 29**. Rostrocaudal length of the antorbital fossa

0: greater than that of the orbit

1: less than that of the orbit

**Character 30**. Posteroventral extent of medial wall of antorbital fossa:

0: reaching the anterior tip of the jugal

1: terminating anterior to the anterior tip of the jugal

**Character 31**. Development of the antorbital fossa on the ascending ramus of the maxilla:

0: deeply impressed and delimited by a sharp, scarp-like rim

1: weakly impressed and delimited by arounded rim or a change in slope

**Character 32**. Shape of the antorbital fossa

0: crescentic with a strongly concave posterior margin that is roughly parallel to the rostral margin of the antorbital fossa

1: subtriangular with a straight to gently concave posterior margin

2: antorbital fossa absent

**Character 33**. Size of the neurovascular foramen at the caudal end of the lateral maxillary row

0: not larger than the others

1: distinctly larger than the others in the row

**Character 34**. Direction that the neurovascular foramen at the caudal end of the lateral maxillary row opens

0: caudally

1: rostrally, ventrally or laterally

**Character 35**. Arrangement of lateral maxillary neurovascular foramina

0: linear

1: irregular

**Character 36**. Longitudinal ridge on the posterior lateral surface of the maxilla

0: absent

1: present

**Character 37**. Dorsal exposure of the lacrimal

0: present

1: absent

**Character 38**. Shape of the lacrimal

0: dorsoventrally short and blockshaped

1: dorsoventrally elongate and shaped like an inverted L

**Character 39**. Orientation of the lacrimal orbital margin

0: strongly sloping anterodorsally

1: erect and close to vertical

**Character 40**. Length of the anterior ramus of the lacrimal

0: greater than half the length of the ventral ramus

1: less than half the length of the ventral ramus

2: absent altogether

**Character 41**. Web/flange of bone spanning junction between anterior and ventral rami of lacrimal

0: absent and antorbital fossa laterally exposed

1: present, obscuring posterodorsal corner of antorbitalfossa

**Character 42**. Extension of the antorbital fossa onto the ventral end of the lacrimal

0: present

1: absent

**Character 43**. Length of the caudal process of the prefrontal

0: short

1: elongated, so that total prefrontal length is equal to the rostrocaudal diameter of the orbit

**Character 44**. Ventral process of prefrontal extending down the posteromedial side of the lachrymal

0: present

1: absent

**Character 45**. Maximum transverse width of the prefrontal

0: less than 0.25 of the skull width at that level

1: more than 0.25 of the skull width at that level

**Character 46**. Shape of the orbit

0: subcircular

1: ventrally constricted making the orbit subtriangular

**Character 47**. Slender anterior process of the frontal intruding between the prefrontal and the nasal

0: absent

1: present

**Character 48**. Jugal-lachrymal relationship

0: lachrymal overlapping lateral surface of jugal or abutting it dorsally

1: jugal overlapping lachrymal laterally

**Character 49**. Shape of the suborbital region of the jugal

0: an anteroposteriorly elongate bar

1: an anteroposteriorly shortened plate

**Character 50**. Jugal contribution to the antorbital fenestra

0: present

1: absent

**Character 51**. Dorsal process of the anterior jugal

0: present

1: absent

**Character 52**. Ratio of the minimum depth of the jugal below the orbit to the distance between the anterior end of the jugal and the anteroventral corner of the infratemporal fenestra

0: less than 0.2:

1: greater than 0.2:

**Character 53**. Transverse width of the ventral ramus of the postorbital

0: less than its anteroposterior width at midshaft

1: greater than its anteroposterior width at midshaft

**Character 54**. Shape of the dorsal margin of postorbital in lateral view

0: straight to gently curved

1: with a distinct embayment (that interrupts the dorsal continuation of the two processes more so than a simple "notch") between the anterior and posterior dorsal processes

**Character 55**. Height of the postorbital rim of the orbit

0: flush with the posterior lateral process of the postorbital

1: raised so that it projects laterally to the posterior dorsal process

**Character 56**. Postfrontal bone

0: present

1: absent

**Character 57**. Position of the anterior margin of the infratemporal fenestra

0: behind the orbit

1: extends under the rear half of the orbit

2: extends as far forward as the midlength of the orbit

**Character 58**. Frontal contribution to the supratemporal fenestra

0: present

1: absent

**Character 59**. Orientation of the long axis of the supratemporal fenestra

0: longitudinal

1: transverse

**Character 60**. Medial margin of supratemporal fossa

0: simple smooth curve

1: with a projection at the frontal/postorbital-parietal suture producing a scalloped margin

**Character 61**. Length of the quadratojugal ramus of the squamosal relative to the width at its base

0: less than four times its width

1: greater than four times its width

**Character 62**. Proportion of infratemporal fenestra bordered by squamosal

0: more than 0.5 of the depth of the infratemporal fenestra

1: less than 0.5 of the depth of the infratemporal fenestra

**Character 63**. Squamosal-quadratojugal contact

0: present

1: absent

**Character 64**. Angle of divergence between jugal and squamosal rami of quadratojugal

0: close to 90: degrees

1: close to parallel

**Character 65**. Length of jugal ramus of quadratojugal

0: no longer than the squamosal ramus

1: longer than the squamosal ramus

**Character 66**. Shape of the rostral end of the jugal ramus of the quadratojugal

0: tapered

1: dorsoventrally expanded

**Character 67**. Relationship of quadratojugal to jugal

0: jugal overlaps the lateral surface of the quadratojugal

1: quadratojugal overlaps the lateral surface of the jugal

2: quadratojugal sutures along the ventrolateral margin of the jugal

**Character 68**. Position of the quadrate foramen

0: on the quadrate-quadratojugal suture

1: deeply incised into, and partly encircled by, the quadrate

2: on the quadrate-squamosal suture, just below the quadrate head

**Character 69**. Shape of posterolateral margin of quadrate

0: sloping anterolaterally from posteromedial ridge

1: everted posteriorly creating a posteriorly facing fossa

2: posterior fossa deeply excavated, invading quadrate body

**Character 70**. Exposure of the lateral surface of the quadrate head

0: absent, covered by lateral sheet of the squamosal

1: present

**Character 71**. Proportion of the length of the quadrate that is occupied by the pterygoid wing

0: at least 70 per cent

1: greater than 70 per cent

**Character 72**. Depth of the occipital wing of the parietal

0: less than 1.5 times the depth of the foramen magnum

1: more than 1.5 times the depth of the foramen magnum

**Character 73**. Position of foramina for mid-cerebral vein on occiput

0: between supraoccipital and parietal

1: on the supraoccipital

**Character 74**. Postparietal fenestra between supraoccipital and parietals

0: absent

1: present

**Character 75**. Shape of the supraoccipital

0: diamond-shaped, at least as high as wide

1: semilunate and wider than high

**Character 76**. Orientation of the supraoccipital plate

0: erect to gently sloping

1: strongly sloping forward so that the dorsal tip lies level with the basipterygoid processes

**Character 77**. Orientation of the paroccipital processes in occipital view

0: slightly dorsolaterally directed to horizontal

1: ventrolaterally directed

**Character 78**. Orientation of the paroccipital processes in dorsal view

0: posterolateral forming a V-shaped occiput

1: lateral forming a flat occiput

**Character 79**. Size of the post-temporal fenestra

0: large fenestra

1: a small hole that is much less than half the depth of the paroccipital process

**Character 80**. Exit of the mid-cerebral vein

0: through trigeminal foramen

1: through a separate foramen anterodorsal to trigeminal foramen

**Character 81**. Shape of the floor of the braincase in lateral view

0: relatively straight with the basal tuberae, basipterygoid processes and parasphenoid rostrum roughly aligned

1: bent with the basipterygoid processes and the parasphenoid rostrum below the level of the basioccipital condyle and the basal tuberae

2: bent with the basal tuberae lowered below the level of the basioccipital and the parasphenoid rostrum raised above it

**Character 82**. Shape of basal tuberae

0: knob-like, with basisphenoidal component rostral to basioccipital component

1: forming a transverse ridge with the basisphenoidal component lateral to

the basioccipital component

**Character 83**. Length of the basipterygoid processes (from the top of the parasphenoid to the tip of the process)

0: less than the height of the braincase (from the top of the parasphenoid to the top of the supraoccipital)

1: greater than the height of the braincase (from the top of the parasphenoid to the top of the supraoccipital)

**Character 84**. Ridge formed along the junction of the parabasisphenoid and the basioccipital, between the basal tuberae

0: present with a smooth rostral face

1: present with a median fossa on the rostral face

2: absent with the basal tuberae being separated by a deep caudally opening U-shaped fossa

**Character 85**. Deep septum spanning the interbasipterygoid space

0: absent

1: present

**Character 86**. Dorsoventral depth of the parasphenoid rostrum

0: much less than the transverse width

1: about equal to the transverse width

**Character 87**. Shape of jugal process of ectopterygoid

0: gently curved

1: strongly recurved and hook-like

**Character 88**. Pneumatic fossa on the ventral surface of the ectopterygoid

0: present

1: absent

**Character 89**. Relationship of the ectopterygoid to the pterygoid

0: ectopterygoid overlapping the ventral surface of the pterygoid

1: ectopterygoid overlapping the dorsal surface of the pterygoid

**Character 90**. Position of the maxillary articular surface of the palatine

0: along the lateral margin of the bone

1: at the end of a narrow anterolateral process due to the absence of the posterolateral process

**Character 91**. Centrally located tubercle on the ventral surface of palatine

0: absent

1: present

**Character 92**. Medial process of the pterygoid forming a hook around the basipterygoid process

0: absent

1: flat and blunt-ended

2: bent upward and pointed

**Character 93**. Length of the vomers

0: less than 0.25 of the total skull length

1: more than 0.25 of the total skull length

**Character 94**. Position of jaw joint

0: no lower than the level of the dorsal margin of the dentary

1: depressed well below this level

**Character 95**. Shape of upper jaws in ventral view

0: narrow with an acute rostral apex

1: broad and U-shaped

**Character 96**. Length of the external mandibular fenestra

0: more than 0.1: of the length of the mandible

1: less than 0.1: of the length of the mandible

**Character 97**. Caudal end of dentary tooth row medially inset with a thick lateral ridge on the dentary forming a buccal emargination

0: absent

1: present

**Character 98**. Height : length ratio of the dentary

0: less than 0.2:

1: greater than 0.2:

**Character 99**. Orientation of the symphyseal end of the dentary

0: in line with the long axis of the dentary

1: strongly curved ventrally

**Character 100**. Position of first dentary tooth

0: adjacent to symphysis

1: inset one tooth's width from the symphysis

**Character 101**. Dorsoventral expansion at the symphyseal end of the dentary

0: absent

1: present

**Character 102**. Splenial foramen

0: absent

1: present and enclosed

2: present and open anteriorly

**Character 103**. Splenial-angular joint

0: flattened sutured contact

1: synovial joint surface between tongue-like process of angular fitting in groove of the splenial

**Character 104**. A stout, triangular, medial process of the articular, behind the glenoid

0: present

1: absent

**Character 105**. Length of the retroarticular process

0: less than the depth of the mandible below the glenoid

1: greater than the depth of the mandible below the glenoid

**Character 106**. Strong medial embayment behind glenoid of the articular in dorsal view

0: absent

1: present

**Character 107**. Number of premaxillary teeth

0: four

1: more than four

**Character 108**. Number of dentary teeth (in adults)

0: less than 18

1: 18 or more

**Character 109**. Arrangement of teeth within the jaws

0: linearly placed, crowns not overlapping

1: imbricated with distal side of tooth overlapping mesial side of the succeeding tooth

**Character 110**. Orientation of the maxillary tooth crowns

0: erect

1: procumbent

**Character 111**. Orientation of the dentary tooth crowns

0: erect

1: procumbent

**Character 112**. Teeth with basally constricted crowns

0: absent

1: present

**Character 113**. Tooth-tooth occlusal wear facets

0: absent

1: present

**Character 114**. Mesial and distal serrations of the teeth

0: fine and set at right angles to the margin of the tooth

1: coarse and angled upwards at an angle of 45 degrees to the margin of the tooth

**Character 115**. Distribution of serrations on the maxillary and dentary teeth

0: present on both the mesial and distal carinae

1: absent on the posterior carinae

2: absent on both carinae

**Character 116**. Long axis of the tooth crowns distally recurved

0: present

1: absent

**Character 117**. Texture of the enamel surface

0: entirely smooth

1: finely wrinkled in some patches

2: extensively and coarsely wrinkled

**Character 118**. Lingual concavities of the teeth

0: absent

1: present

**Character 119**. Longitudinal labial grooves on the teeth

0: absent

1: present

**Character 120**. Distribution of the serrations along the mesial and distal carinae of the tooth

0: extend along most of the length of the crown

1: restricted to the upper half of the crown

**Character 121**. Number of cervical vertebrae

0: eight or fewer

1: 9-10

2: 12-13:

3: more than 13:

**Character 122**. Shallow, dorsally facing fossa on the atlantal neurapophysis bordered by a dorsally everted lateral margin

0: absent

1: present

**Character 123**. Width of axial intercentrum

0: less than width of axial centrum

1: greater than width of axial centrum

**Character 124**. Position of axial prezygapophyses

0: on the anterolateral surface of the neural arch

1: mounted on anteriorly projecting pedicels

**Character 125**. Posterior margin of the axial postzygapophyses

0: extend well beyond the posterior margin of the axial centrum

1: flush with (or only marginally posterior to) the posterior margin of the axial centrum

**Character 126**. Length of the axial centrum

0: less than three times the height of the centrum

1: at least three times the height of the centrum

**Character 127**. Length of the anterior cervical centra (cervicals 3-5)

0: no more than the length of the axial centrum

1: greater than the length of the axial centrum

**Character 128**. Length of middle to posterior cervical centra (cervical 6-8)

0: no more than the length of the axial centrum

1: greater than the length of the axial centrum

**Character 129**. Dorsal excavation of the cervical parapophyses

0: absent

1: present

**Character 130**. Lateral compression of the anterior cervical vertebrae

0: centra are no higher than they are wide

1: are approximately 1.25 times higher than wide

**Character 131**. Relative elongation of the anterior cervical centra (cervical 3-5)

0: lengths of the centra are less than 2.5 times the height of their anterior faces

1: lengths are 2.5-4: times the height of their anterior faces

2: the length of at least cervical 4: or 5 exceeds 4: times the anterior centrum height

**Character 132**. Ventral keels on cranial cervical centra

0: present

1: absent

**Character 133**. Height of the mid cervical neural arches

0: no more than the height of the posterior centrum face

1: greater than the height of the posterior centrum face

**Character 134**. Orientation of the anterior-to-middle cervical postzygapophyses

0: Planar (minimally offset) with respect to the prezygapophyses

1: Dorsally raised roughly 20 degrees relative to the coronal plane

2: Dorsally raised at least 30 degrees or more relative to the coronal plane

**Character 135**. Cervical epipophyses on the dorsal surface of the postzygapophyses

0: absent

1: present on at least some cervical vertebrae

**Character 136**. Caudal ends of cranial, postaxial epipophyses

0: with a free pointed tip

1: joined to the postzygapophysis along their entire length

**Character 137**. Shape of the epipophyses

0: tall ridges

1: flattened, horizontal plates

**Character 138**. Epipophyses overhanging the rear margin of the postzygapophyses

0: present in at least some postaxial cervical vertebrae

1: absent

**Character 139**. Anterior spur-like projections on mid-cervical neural spines

0: absent

1: present

**Character 140**. Shape of mid-cervical neural spines

0: less than twice as long as high

1: at least twice as long as high

**Character 141**. Shape of cervical rib shafts

0: short and posteroventrally directed

1: longer than the length of their centra and extending parallel to cervical column

**Character 142**. Position of the base of the cervical rib shaft

0: level with, or higher than the ventral margin of the cervical centrum

1: located below the ventral margin due to a ventrally extended parapophysis

**Character 143**. Postzygodiapophyseal lamina in cervical neural arches 4-8

0: present

1: absent

**Character 144**. Laminae of the cervical neural arches 4-8

0: well-developed tall laminae

1: weakly developed low ridges

**Character 145**. Shape of anterior centrum face in cervical centra

0: concave

1: flat

2: convex

**Character 146**. Ventral surface of the centra in the cervicodorsal transition

0: transversely rounded

1: with longitudinal keels

**Character 147**. Number of vertebrae between cervicodorsal transition and primordial sacral vertebrae

0: 15-16

1: no more than 14:

**Character 148**. Lateral surfaces of the dorsal centra

0: with at most vague, shallow depressions

1: with deep fossae that approach the midline

2: with invasive, sharp-rimmed pleurocoels

**Character 149**. Oblique ridge dividing pleural fossa of cervical vertebrae

0: absent

1: present

**Character 150**. Laterally expanded tables at the midlength of the dorsal surface of the neural spines

0: absent in all vertebrae

1: present on the pectoral vertebrae

2: present on the pectoral and cervical vertebrae

3: present and greatly expanded on the pectoral and cervical vertebrae (NEW STATE)

**Character 151**. Dorsal centra

0: entirely amphicoelous to amphiplatyan

1: first two dorsals are opisthocoelous

2: cranial half of dorsal column is opisthocoelous

**Character 152**. Shape of the posterior dorsal centra

0: relatively elongated for their size

1: strongly axially compressed for their size

**Character 153**. Laminae bounding triangular infradiapophyseal fossae (chonae) on dorsal neural arches

0: absent

1: present

**Character 154**. Location of parapophysis in first two dorsals

0: at the anterior end of the centrum

1: Set back from the anterior margin, within lateral surface of centrum

**Character 155**. Parapophyses of the dorsal column completely shift from the centrum to the neural arch

0: anterior to the thirteenth presacral vertebra

1: posterior to the thirteenth presacral vertebra

**Character 156**. Orientation of the transverse processes of the dorsal vertebrae

0: most horizontally directed

1: all upwardly directed

**Character 157**. Contribution of the paradiapophyseal lamina to the margin of the anterior chonos in mid-dorsal vertebrae

0: present

1: prevented by high placement of parapophysis

**Character 158**. Hyposphenes in the dorsal vertebrae

0: absent

1: present but less than the height of the neural canal

2: present and equal to the height of the neural canal

**Character 159**. Prezygodiapophyseal lamina and associated anterior triangular fossa (chonos)

0: present on all dorsals

1: absent in mid-dorsals

**Character 160**. Anterior centroparapophyseal lamina in dorsal vertebrae

0: absent

1: present

**Character 161**. Prezygoparapophyseal lamina in dorsal vertebrae

0: absent

1: present

**Character 162**. Accessory lamina dividing posterior chonos from postzygapophysis

0: absent

1: present

**Character 163**. Pneumatic excavation of the dorsal neural arches

0: absent

1: equivocal (e.g., no more than depressions within the infradiapophyseal chambers)

2: sharp-rimmed fossae or foramina clearly invading bone surface

**Character 164**. Separation of lateral surfaces of anterior dorsal neural arches under transverse processes

0: widely spaced

1: only separated by a thin midline septum

**Character 165**. Height of dorsal neural arches, from neurocentral suture to level of zygapophyseal facets

0: much less than height of centrum

1: subequal to or greater than height of centrum

**Character 166**. Form of anterior surface of neural arch

0: simple centroprezygopophyseal ridge

1: broad anteriorly facing surface bounded laterally by centroprezygopophyseal lamina

**Character 167**. Shape of posterior dorsal neural canal

0: subcircular

1: slit-shaped

**Character 168**. Height of middle dorsal neural spines

0: less than the length of the base

1: higher than the length of the base but less than 1.5 times the length of the base

2: greater than 1.5 times the length of the base

**Character 169**. Shape of anterior dorsal neural spines

0: lateral margins subparallel in anterior view

1: transversely expanding towards dorsal end

2: greatly expanded dorsally (NEW STATE)

**Character 170**. Cross-sectional shape of dorsal neural spines

0: transversely compressed

1: broad and triangular

2: square-shaped in posterior vertebrae

**Character 171**. Spinodiapophyseal lamina on dorsal vertebrae

0: absent

1: present and separated from spinopostzygapophyseal lamina

2: present and joining spinopostzygapophyseal lamina to create a composite posterolateral spinal lamina

**Character 172**. Well-developed, sheet-like suprapostzygapophyseal laminae

0: absent

1: present on at least the caudal dorsal vertebrae

**Character 173**. Shape of the spinopostzygapophyseal lamina in middle and posterior dorsal vertebrae

0: singular

1: bifurcated at its distal end

**Character 174**. Shape of posterior margin of middle dorsal neural spines in lateral view

0: approximately straight

1: concave with a projecting posterodorsal corner

**Character 175**. Transversely expanded plate-like summits of posterior dorsal neural spines

0: absent

1: present

**Character 176**. Last presacral rib

0: free

1: fused to vertebra

**Character 177**. Sacral rib much narrower than the transverse process of the first primordial sacral vertebra (and dorsosacral if present) in dorsal view

0: absent

1: present

**Character 178**. Number of dorsosacral vertebrae

0: none

1: one

2: two

**Character 179**. Caudosacral vertebra

0: absent

1: present

**Character 180**. Shape of the iliac articular facets of the first primordial sacral rib

0: singular

1: divided into dorsal and ventral facets separated by a non-articulating gap

**Character 181**. Deep, medially-directed pit excavating the surface of the non-articulating gap of the first primordial sacral rib

0: absent

1: present

**Character 182**. Depth of the iliac articular surface of the primordial sacrals

0: less than 0.75 of the depth of the ilium

1: greater than 0.75 of the depth of the ilium

**Character 183**. Sacral ribs contributing to the rim of the acetabulum

0: absent

1: present

**Character 184**. Posterior and anterior expansion of the transverse processes of the first and second primordial sacral vertebrae, respectively, partly roofing the intercostal space

0: absent

1: present

**Character 185**. Length of first caudal centrum

0: longer anteroposteriorly than dorsoventrally tall

1: taller than long

2: highly compressed (dorsoventral height at least twice anteroposterior length)

**Character 186**. Position of postzygapophyses in proximal caudal vertebrae

0: protruding with an interpostzygapophyseal notch visible in dorsal view

1: placed on either side of the caudal end of the base of the neural spine without any interpostzygapophyseal notch

**Character 187**. A hyposphenal ridge on caudal vertebrae

0: absent

1: present

**Character 188**. Prezygadiapophyseal laminae on anterior caudals

0: absent

1: present

**Character 189**. Depth of the bases of the anterior caudal transverse processes

0: dorsoventrally shallow and directed mainly anteroposteriorly, with only a minimal incursion onto the centrum

1: dorsoventrally deep, with a strong ventral cant extending from the neural arch to the centrum

**Character 190**. Position of last caudal vertebra with a protruding transverse process

0: distal to caudal 16

1: proximal to caudal 16

**Character 191**. Orientation of posterior margin of proximal caudal neural spines

0: sloping posterodorsally

1: vertical

**Character 192**. Longitudinal ventral sulcus on proximal and middle caudal vertebrae

0: present

1: absent

**Character 193**. Length of midcaudal centra

0: greater than twice the height of their anterior faces

1: less than twice the height of their anterior faces

**Character 194**. Cross-sectional shape of the distal caudal centra

0: oval with rounded lateral and ventral sides

1: square-shaped with flattened lateral and ventral sides

**Character 195**. Length of distal caudal prezygapophyses

0: short, not overlapping the preceding centrum by more than a quarter

1: long and overlapping the preceding the centrum by more than a quarter

**Character 196**. Shape of the terminal caudal vertebrae

0: unfused, size decreasing toward tip

1: expanded and fused to form a club-shaped tail

**Character 197**. 'Weaponized' dermal spikes on tail

0: absent

1: present

**Character 198**. Length of the longest chevron

0: less than twice the length of the preceding centrum

1: greater than twice the length of the preceding centrum

**Character 199**. Anteroventral process on distal chevrons

0: absent

1: present

**Character 200**. Mid-caudal chevrons with a ventral slit

0: absent

1: present

**Character 201**. Longitudinal ridge on the dorsal surface of the sternal plate

0: absent

1: present

**Character 202**. Dorsoventral length of the acromion process of the scapula (when long axis of scapula is oriented vertically)

0: less than 1.5 times the minimum width of the scapula blade

1: greater than 1.5 times the minimum width of the scapula blade

**Character 203**. Minimum width of the scapula

0: greater than 20 per cent of its length

1: less than 20 per cent of its length

**Character 204**. Dorsal margin of the acromion process of the scapula

0: rises from the blade at angle that is less than 65 degrees from the long axis of the scapula, at its steepest point

1: rises from the blade at angle that is greater than 65 degrees from the long axis of the scapula, at its steepest point

**Character 205**. Width of dorsal expansion of the scapula

0: less than the width of the ventral end of the scapula

1: equal to the width of the ventral end of the scapula

**Character 206**. Flat caudoventrally facing surface on the coracoids between glenoid and coracoid tubercle

0: absent

1: present

**Character 207**. Coracoid tubercle

0: present

1: absent

**Character 208**. Length of the humerus

0: less than 55 per cent of the length of the femur

1: 55-65 per cent of the length of the femur

2: 65-70 per cent of the length of the femur

3: more than 70 per cent of the length of the femur

**Character 209**. Shape of the humeral head

0: Slightly-developed, rounded in anteroposterior view

1: Flat in anteroposterior view with a minimally expanded lateral component

2: Domed, being convex/hemispherical in anteroposterior view with a strong lateral incursion onto the humeral shaft

**Character 210**. Shape of the deltopectoral crest

0: subtriangular

1: subrectangular

2: reduced to low ridge

**Character 211**. Length of the deltopectoral crest of the humerus

0: less than 30 per cent of the length of the humerus

1: 30-50 per cent of the length of the humerus

2: greater than 50 per cent of the length of the humerus

**Character 212**. Shape of the anterolateral margin of the deltopectoral crest of the humerus

0: straight

1: strongly sinuous

**Character 213**. Rugose pit centrally located on the lateral surface of the deltopectoral crest

0: absent

1: present

**Character 214**. Well-defined fossa on the distal flexor surface of the humerus

0: present

1: absent

**Character 215**. Transverse width of the distal humerus

0: is less than 33: per cent of the length of the humerus

1: greater than 33: per cent of the length of the humerus

**Character 216**. Shape of the entepicondyle of the distal humerus

0: rounded process

1: with a flat distomedially facing surface bounded by a sharp proximal margin

**Character 217**. Length of the radius

0: greater than 80 per cent of the humerus

1: less than 80 per cent of the humerus

**Character 218**. Radial fossa on anterolateral corner of proximal ulna

0: absent

1: present, but only shallowly defined

2: a well-defined recess, deeper than the transverse width of the anterior end of the anterior process

**Character 219**. Olecranon process on proximal ulna

0: present

1: absent

2: greatly enlarged olecranon

**Character 220**. Maximum linear dimensions of the ulnare and radiale

0: exceed that of at least one of the first three distal carpals

1: less than any of the distal carpals

**Character 221**. Transverse width of the first distal carpal

0: less than 120 per cent of the transverse width of the second distal carpal

1: greater than 120 per cent of the transverse width of the second distal carpal

**Character 222**. Sulcus across the medial end of the first distal carpal

0: absent

1: present

**Character 223**. Lateral end of first distal carpal

0: abuts second distal carpal

1: overlaps second distal carpal

**Character 224**. Second distal carpal

0: completely covers the proximal end of the second metacarpal

1: does not completely cover the proximal end of the second metacarpal

**Character 225**. Ossification of the fifth distal carpal

0: present

1: absent

**Character 226**. Length of the manus

0: less than 38 per cent of the humerus + radius

1: 38-45 per cent of the humerus + radius

2: greater than 45 per cent of the humerus + radius

**Character 227**. Shape of metacarpus

0: flattened to gently curved and spreading

1: a colonnade of subparallel metacarpals tightly curved into a U-shape

**Character 228**. Proximal width of first metacarpal

0: less than the proximal width of the second metacarpal

1: greater than the proximal width of the second metacarpal

**Character 229**. Minimum transverse shaft width of first metacarpal

0: less than twice the minimum transverse shaft width of second metacarpal

1: greater than twice the minimum transverse shaft width of second metacarpal

**Character 230**. Proximal end of first metacarpal

0: flush with other metacarpals

1: inset into the carpus

**Character 231**. Shape of the first metacarpal

0: proximal width less than 65 per cent of its length

1: proximal width 65-80 per cent of its length

2: proximal width 80-100 per cent of it length

3: greater than 100 per cent of its length

**Character 232**. Strong asymmetry in the lateral and medial distal condyles of the first metacarpal

0: absent

1: present

**Character 233**. Deep distal extensor pits on the second and third metacarpals

0: absent

1: present

**Character 234**. Shape of the distal ends of second and third metacarpals

0: subrectangular in distal view

1: trapezoidal with flexor rims of distal collateral ligament pits flaring beyond extensor rims

**Character 235**. Shape of the fifth metacarpal

0: longer than wide at the proximal end with a flat proximal surface

1: almost as wide as it is long with a strongly convex proximal articulation surface

**Character 236**. Length of the fifth metacarpal

0: less than 75 per cent of the length of the third metacarpal

1: greater than 75 per cent of the length of the third metacarpal

**Character 237**. Length of manual digit one

0: less than the length of manual digit two

1: greater than the length of manual digit two

**Character 238**. Ventrolateral twisting of the transverse axis of the distal end of the first phalanx of manual digit one relative to its proximal end

0: absent

1: present but much less than 60 degrees

2: 60 degrees

**Character 239**. Length of the first phalanx of manual digit one

0: less than the length of the first metacarpal

1: greater than the length of the first metacarpal

**Character 240**. Shape of the proximal articular surface of the first phalanx of manual digit one

0: rounded

1: with an embayment on the medial side

**Character 241**. Shape of the first phalanx of manual digit one

0: elongate and subcylindrical

1: strongly proximodistally compressed and wedge-shaped

**Character 242**. Length of the penultimate phalanx of manual digit two

0: less than the length of the second metacarpal

1: greater than the length of the second metacarpal

**Character 243**. Length of the penultimate phalanx of manual digit three

0: less than the length of the third metacarpal

1: greater than the length of the third metacarpal

**Character 244**. Shape of non-terminal phalanges of manual digits two and three

0: longer than wide

1: as long as wide

**Character 245**. Shape of the unguals of manual digits two and three

0: straight

1: strongly curved with tips projecting well below flexor margin of proximal articular surface

**Character 246**. Length of the ungual of manual digit two

0: greater than the length of the ungual of manual digit one

1: 75-100 per cent of the ungual of manual digit one

2: less than 75 per cent of the ungual of manual digit one

3: the ungual of manual digit two is absent

**Character 247**. Phalangeal formula of manual digits two and three

0: three and four, respectively

1: with at least one phalanx missing from each digit

**Character 248**. Phalangeal formula of manual digits four and five

0: greater than 2-0, respectively

1: less than 2-0, respectively

**Character 249**. Strongly convex dorsal margin of the ilium

0: absent

1: present

**Character 250**. Cranial extent of preacetabular process of ilium

0: does not project further anterior than the cranial margin of the pubic peduncle

1: projects anterior to the cranial margin of the pubic peduncle

**Character 251**. Shape of the preacetabular process

0: blunt and rectangular

1: with a pointed, projecting cranioventral corner and a rounded dorsum

**Character 252**. Depth of the preacetabular process of the ilium

0: much less than the depth of the ilium above the acetabulum

1: subequal to the depth of the ilium above the acetabulum

**Character 253**. Length of preacetabular process of the ilium

0: less than twice its depth

1: greater than twice its depth

**Character 254**. Medial wall of acetabulum

0: fully closing acetabulum with a triangular ventral process between the pubic and ischial peduncles

1: partially open acetabulum with a straight ventral margin between the peduncles

2: partially open acetabulum with a concave ventral margin between the peduncles

3: fully open acetabulum with medial ventral margin closely approximating lateral rim of acetabulum

**Character 255**. Length of the pubic peduncle of the ilium

0: less than twice the craniocaudal width of its distal end

1: greater than twice the craniocaudal width of its distal end

**Character 256**. Caudally projecting 'heel' at the distal end of the ischial peduncle

0: absent

1: present

**Character 257**. Length of the ischial peduncle of the ilium

0: similar to pubic peduncle

1: much shorter than pubic peduncle

2: virtually absent so that the chord connecting the distal end of the pubic peduncle with the ischial articular surface contacts the postacetabular process

**Character 258**. Length of the postacetabular process of the ilium

0: between 40 and 100 per cent of the distance between the pubic and ischial peduncles

1: less than 40 per cent of the distance between the pubic and ischial peduncles

2: more than 100 per cent of the distance between the pubic and ischial peduncles

**Character 259**. Well-developed brevis fossa with sharp margins on the ventral surface of the postacetabular process of the ilium

0: absent

1: present, ventrally facing

2: present, lateroventrally facing

**Character 260**. Anterior end of ventrolateral ridge bounding brevis fossa

0: not connected to supracetabular crest

1: joining supracetabular crest

**Character 261**. Shape of the caudal margin of the postacetabular process of the ilium

0: rounded to bluntly pointed

1: square ended

2: with a pointed ventral corner and a rounded caudodorsal margin

**Character 262**. Width of the conjoined pubes

0: less than 75 per cent of their length

1: greater than 75 per cent of their length

**Character 263**. Pubic tubercle on the lateral surface of the proximal pubis

0: present

1: absent

**Character 264**. Proximal anterior profile of pubis

0: anterior margin of pubic apron smoothly confluent with anterior margin of iliac pedicel

1: iliac pedicel set anterior to the pubic apron creating a prominent inflection in the proximal anterior profile of the pubis

**Character 265**. Minimum transverse width of the pubic apron

0: much more than 40 per cent of the width across the iliac peduncles of the ilium

1: less than 40 per cent of the width across the iliac peduncles of the ilium

**Character 266**. Position of the obturator foramen of the pubis

0: at least partially occluded by the iliac pedicel in anterior view

1: completely visible in anterior view

**Character 267**. Lateral margins of the pubic apron in anterior view

0: straight

1: concave

**Character 268**. Orientation of distal third of the blades of the pubic apron

0: confluent with the proximal part of the pubic apron

1: twisted posterolaterally relative to proximal section so that the anterior surface turns to face laterally

**Character 269**. Orientation of the entire blades of the pubic apron

0: transverse

1: twisted posteromedially

**Character 270**. Craniocaudal expansion of the distal pubis

0: absent

1: less than 15 per cent of the length of the pubis

2: greater than 15 per cent of the length of the pubis

**Character 271**. Elongate interischial fenestra

0: absent

1: present

**Character 272**. Longitudinal dorsolateral sulcus extending along proximal half of the shaft of the ischium

0: absent

1: present

**Character 273**. Shape of distal ischium

0: broad and plate-like, not distinct from obturator region

1: with a discrete rod-like distal shaft

**Character 274**. Length of ischium

0: less than that of the pubis

1: greater than that of the pubis

**Character 275**. Ischial component of acetabular rim

0: larger than the pubic component

1: equal to the pubic component

**Character 276**. Shape of the transverse section of the ischial shaft

0: ovoid to subrectangular

1: triangular

**Character 277**. Orientation of the long axes of the transverse section of the distal ischia

0: meet at an angle

1: are coplanar

**Character 278**. Depth of the transverse section of the ischial shaft

0: much less than the transverse width of the section

1: at least as great as the transverse width of the section

**Character 279**. Distal ischial expansion

0: absent

1: present

**Character 280**. Transverse width of the conjoined distal ischial expansions

0: greater than their sagittal depth

1: less than their sagittal depth

**Character 281**. Length of the hindlimb

0: greater than the length of the trunk

1: less than the length of the trunk

**Character 282**. Longitudinal axis of the femur in lateral view

0: strongly bent with an offset between the proximal and distal axes greater than 20 degrees

1: weakly bent with an offset of around 20 degrees or less

2: straight

**Character 283**. Shape of the cross-section of the mid-shaft of the femur

0: subcircular

1: strongly elliptical with the long axis orientated mediolaterally

**Character 284**. Angle between the long axis of the femoral head and the transverse axis of the distal femur

0: about 30 degrees

1: close to 0 degrees

**Character 285**. Shape of femoral head

0: roughly rectangular in profile with a sharp medial distal corner

1: roughly hemispherical with no sharp medial distal corner

**Character 286**. Posterior proximal tubercle on femur

0: well developed, producing a pronounced swelling on the posterior margin of the femoral head

1: reduced to absent

**Character 287**. Shape of the lesser trochanter

0: small rounded tubercle

1: proximodistally orientated, elongate ridge

2: absent

**Character 288**. Position of proximal tip of lesser trochanter

0: level with the femoral head

1: distal to the femoral head

**Character 289**. Projection of the lesser trochanter

0: just a scar upon the femoral surface

1: a raised process

**Character 290**. Transverse ridge extending laterally from the lesser trochanter

0: absent

1: present

**Character 291**. Height of the lesser trochanter in cross section

0: less than its basal width

1: at least as high as its basal width

**Character 292**. Position of the lesser trochanter in anterior view

0: near the centre of the anterior face of the femoral shaft

1: close to the lateral margin of the femoral shaft

**Character 293**. Visibility of the lesser trochanter in posterior view

0: not visible

1: visible

**Character 294**. Height of the fourth trochanter

0: a low rugose ridge

1: a tall crest

**Character 295**. Position of the fourth trochanter along the length of the femur

0: in the proximal half

1: straddling the midpoint

**Character 296**. Symmetry of the profile of the fourth trochanter of the femur

0: subsymmetrical without a sharp distal corner

1: asymmetrical with a steeper distal slope than the proximal slope and a distinct distal corner

2: symmetrical, almost rectangular in lateral view with proximal and distal corners approaching an angle of 90: degrees

**Character 297**. Shape of the profile of the fourth trochanter of the femur

0: rounded

1: subrectangular

**Character 298**. Position of fourth trochanter along the mediolateral axis of the femur

0: centrally located

1: on the medial margin

**Character 299**. Extensor depression on anterior surface of the distal end of the femur

0: absent

1: present

**Character 300**. Size of the medial condyle of the distal femur

0: subequal to the fibular + lateral condyles

1: larger than the fibular + lateral condyles

**Character 301**. Well-developed tibiofibular crest on distal femur

0: absent

1: present

**Character 302**. Distal surface of tibiofibular crest

0: as deep anteroposteriorly as wide mediolaterally or deeper

1: wider mediolaterally than deep anteroposteriorly

**Character 303**. Tibia : femur length ratio

0: tibia subequal or longer than the femur

1: between 0.6 and 0.9

2: less than 0.6

**Character 304**. Orientation of cnemial crest

0: projects anteriorly to anterolaterally

1: projecting laterally

**Character 305**. Paramarginal ridge on lateral surface of cnemial crest

0: absent

1: present

**Character 306**. Position of the tallest point of the cnemial crest

0: close to the proximal end of the crest

1: about half-way along the length of the crest, creating an anterodorsally sloping proximal margin of the crest

**Character 307**. Proximal end of tibia with a flange of bone that contacts the fibula

0: absent

1: present

**Character 308**. Position of the posterior end of the fibular condyle on the proximal articular surface tibia

0: anterior to the posterior margin of the proximal articular surface

1: level with the posterior margin of the proximal articular surface

**Character 309**. Shape of the proximal articular surface of the tibia

0: transverse width subequal to anteroposterior length

1: transverse width between 0.6 and 0.9 times anteroposterior length

2: anteroposterior length twice the transverse width or higher

**Character 310**. Transverse width of the distal tibia

0: subequal to its craniocaudal length

1: greater than its craniocaudal length

**Character 311**. Anteroposterior width of the lateral side of the distal articular surface of the tibia

0: as wide as the anteroposterior width of the medial side

1: narrower than the anteroposterior width of the medial side

**Character 312**. Relationship of the posterolateral process of the distal end of the tibia with the fibula

0: not flaring laterally and not making significant contact with the fibula

1: flaring laterally and backing the fibula

**Character 313**. Shape of the distal articular end of the tibia in distal view

0: ovoid

1: subrectangular

**Character 314**. Shape of the anteromedial corner of the distal articular surface of the tibia

0: forming a right angle

1: forming an acute angle

**Character 315**. Position of the lateral margin of descending caudoventral process of the distal end of the tibia

0: protrudes laterally at least as far as the craniolateral corner of the distal tibia

1: set well back from the craniolateral corner of the distal tibia

**Character 316**. A triangular rugose area on the medial side of the fibula

0: absent

1: present

**Character 317**. Transverse width of the midshaft of the fibula

0: greater than 0.75 of the transverse width of the midshaft of the tibia

1: between 0.5 and 0.75 of the transverse width of the midshaft of the tibia

2: less than 0.5 of the transverse width of the midshaft of the tibia

**Character 318**. Position of fibula trochanter

0: on anterior surface of fibula

1: laterally facing

2: anteriorly facing but with strong lateral bulge

**Character 319**. Depth of the medial end of the astragalar body in cranial view

0: roughly equal to the lateral end

1: much shallower creating a wedge-shaped astragalar body

**Character 320**. Shape of the posteromedial (or is supposed to be anteromedial!?) margin of the astragalus in dorsal view

0: evenly rounded without formation of a caudomedial corner

1: forming a moderately sharp corner of a subrectangular astragalus

**Character 321**. Dorsally facing horizontal shelf forming part of the fibular facet of the astragalus

0: present

1: absent with a largely vertical fibular facet

**Character 322**. Pyramidal dorsal process on the posteromedial corner of the astragalus

0: absent

1: present

**Character 323**. Shape of the ascending process of the astragalus

0: anteroposteriorly deeper than transversely wide

1: transversely wider than anteroposteriorly deep

**Character 324**. Posterior extent of ascending process of the astragalus

0: well anterior to the posterior margin of the astragalus

1: close to the posterior margin of the astragalus

**Character 325**. Sharp medial margin around the depression posterior to the ascending process of the astragalus

0: absent

1: present

**Character 326**. Buttress dividing posterior fossa of astragalus and supporting ascending process

0: absent

1: present

**Character 327**. Vascular foramina set in a fossa at the base of the ascending process of the astragalus

0: present

1: absent

**Character 328**. Distal articular surface of astragalus

0: relatively flat or weakly convex

1: extremely convex and roller-shaped

**Character 329**. Transverse width of the calcaneum

0: greater than 30 per cent of the transverse width of the astragalus

1: less than 30 per cent of the transverse width of the astragalus

**Character 330**. Lateral surface of calcaneum

0: simple

1: with a fossa

**Character 331**. Medial peg of calcaneum fitting into astragalus

0: present, even if rudimentary

1: absent

**Character 332**. Calcaneal tuber

0: large and well developed

1: highly reduced to absent

**Character 333**. Shape of posteromedial heel of distal tarsal four (lateral distal tarsal)

0: proximodistally deepest part of the bone

1: no deeper than the rest of the bone

**Character 334**. Shape of posteromedial process of distal tarsal four in proximal view

0: rounded

1: pointed

**Character 335**. Ossified distal tarsals

0: present

1: absent

**Character 336**. Proximal width of the first metatarsal

0: is less than the proximal width of the second metatarsal

1: at least as great as the proximal width of the second metatarsal

**Character 337**. Size of first metatarsal

0: maximum proximal breadth 0.4: times its proximodistal length or less

1: maximum proximal breadth between 0.4: and 0.7 times its proximodistal length

2: maximum proximal breadth greater than 0.7 times its proximodistal length

**Character 338**. Orientation of proximal articular surface of metatarsal one

0: horizontal

1: sloping proximolaterally relative to the long axis of the bone

**Character 339**. Shaft of metatarsal I

0: closely appressed to metatarsal II throughout its length

1: only closely appressed proximally, with a space between metatarsals I and II distally

**Character 340**. Orientation of the transverse axis of the distal end of metatarsal one

0: horizontal

1: angled dorsomedially

**Character 341**. Shape of the medial margin of the proximal surface of the second metatarsal

0: straight

1: concave

**Character 342**. Shape of the lateral margin of the proximal surface of the second metatarsal

0: straight

1: concave

**Character 343**. Projection of ventral flange on proximal surface of second metatarsal

0: neither corner appreciably more developed than the other

1: laterally flaring

2: medially flaring

**Character 344**. Well-developed facet on proximolateral corner of plantar ventrolateral flange of MT II for articulation with medial distal tarsal

0: absent

1: present

**Character 345**. Length of the third metatarsal

0: greater than 40 per cent of the length of the tibia

1: less than 40 per cent of the length of the tibia

**Character 346**. Proximal outline of metatarsal III

0: subtriangular with acute or rounded posterior border

1: subtrapezoidal, with posterior border broadly exposed in plantar view

**Character 347**. Minimum transverse shaft diameters of third and fourth metatarsals

0: greater than 60 per cent of the minimum tansverse shaft diameter of the second metatarsal

1: less than 60 per cent of the minimum tansverse shaft diameter of the second metatarsal

**Character 348**. Transverse width of the proximal end of the fourth metatarsal

0: less than twice the anteroposterior depth of the proximal end

1: at least twice the anteroposterior depth of the proximal end

**Character 349**. Angle formed by the anterior and anteromedial borders of metatarsal IV

0: obtuse

1: right angle, or acute

**Character 350**. Transverse width of the proximal end of the fifth metatarsal

0: less than 25 per cent of the length of the fifth metatarsal

1: between 30 and 49 per centof the length of the fifth metatarsal

2: greater than 50 per cent of the length of the fifth metatarsal

**Character 351**. Transverse width of distal articular surface of metatarsal four in distal view

0: greater than the anteroposterior depth

1: less than the anteroposterior depth

**Character 352**. Pedal digit five

0: reduced, non-weight bearing

1: large (fifth metatarsal at least 70: per cent of fourth metatarsal), robust and weight bearing

**Character 353**. Length of non-terminal pedal phalanges

0: all longer than wide

1: proximalmost phalanges longer than wide while more distal phalanges are as wide as long

2: all non-terminal phalanges are as wide, if not wider, than long

**Character 354**. Length of the first phalanx of pedal digit one

0: greater than the length of the ungual of pedal digit one

1: less than the length of the ungual of pedal digit one

**Character 355**. Length of the ungual of pedal digit one

0: less than at least some non-terminal phalanges

1: longer than all non-terminal phalanges but shorter than first metatarsal

2: longer than the first metatarsal

**Character 356**. Shape of the ungual of pedal digit one

0: shallow, pointed, with convex sides and a broad ventral surface

1: deep, abruptly tapering, with flattened sides and a narrow ventral surface

**Character 357**. Shape of proximal articular surface of pedal unguals

0: proximally facing, visible on medial and lateral sides

1: proximomedially facing and visible only in medial view, causing medial deflection of pedal unguals in articulation

**Character 358**. Penultimate phalanges of pedal digits two and three

0: well-developed

1: reduced disc-shaped elements if they are ossified at all

**Character 359**. Shape of the unguals of pedal digits two and three

0: dorsoventrally deep with a proximal articulating surface that is at least as deep as it is wide

1: dorsoventrally flattened with a proximal articulating surface that is wider tan deep

**Character 360**. Length of the ungual of pedal digit two

0: greater than the length of the ungual of pedal digit one

1: between 90 and 100 per cent of the length of the ungual of pedal digit one

2: less than 90 per cent of the length of the ungual of pedal digit one

**Character 361**. Size of the ungual of pedal digit three

0: greater than 85 per cent of the ungual of pedal digit two in all linear dimensions

1: less than 85 per cent of the ungual of pedal digit two in all linear dimensions

**Character 362**. Number of phalanges in pedal digit four

0: four

1: fewer than four

**Character 363**. Phalanges of pedal digit five.

0: present

1: absent

**Character 364**. Femoral length:

0: less than 200 mm

1: between 200 and 399 mm

2: between 400 and 599 mm

3: between 600 and 799 mm

4: between 800 and 1000 mm

5: greater than 1000 mm

**Character 365.** Number of foramina in proximal portion of pubis:

0: one

1: two
