# Supplementary figures and images for: Anatomy and systematics of the sauropodomorph Sarahsaurus aurifontanalis from the Early Jurassic Kayenta Formation
Source: PLoS One. 2018 Oct 10;13(10):e0204007. doi: 10.1371/journal.pone.0204007 (PMC6179219; doi:10.1371/journal.pone.0204007)

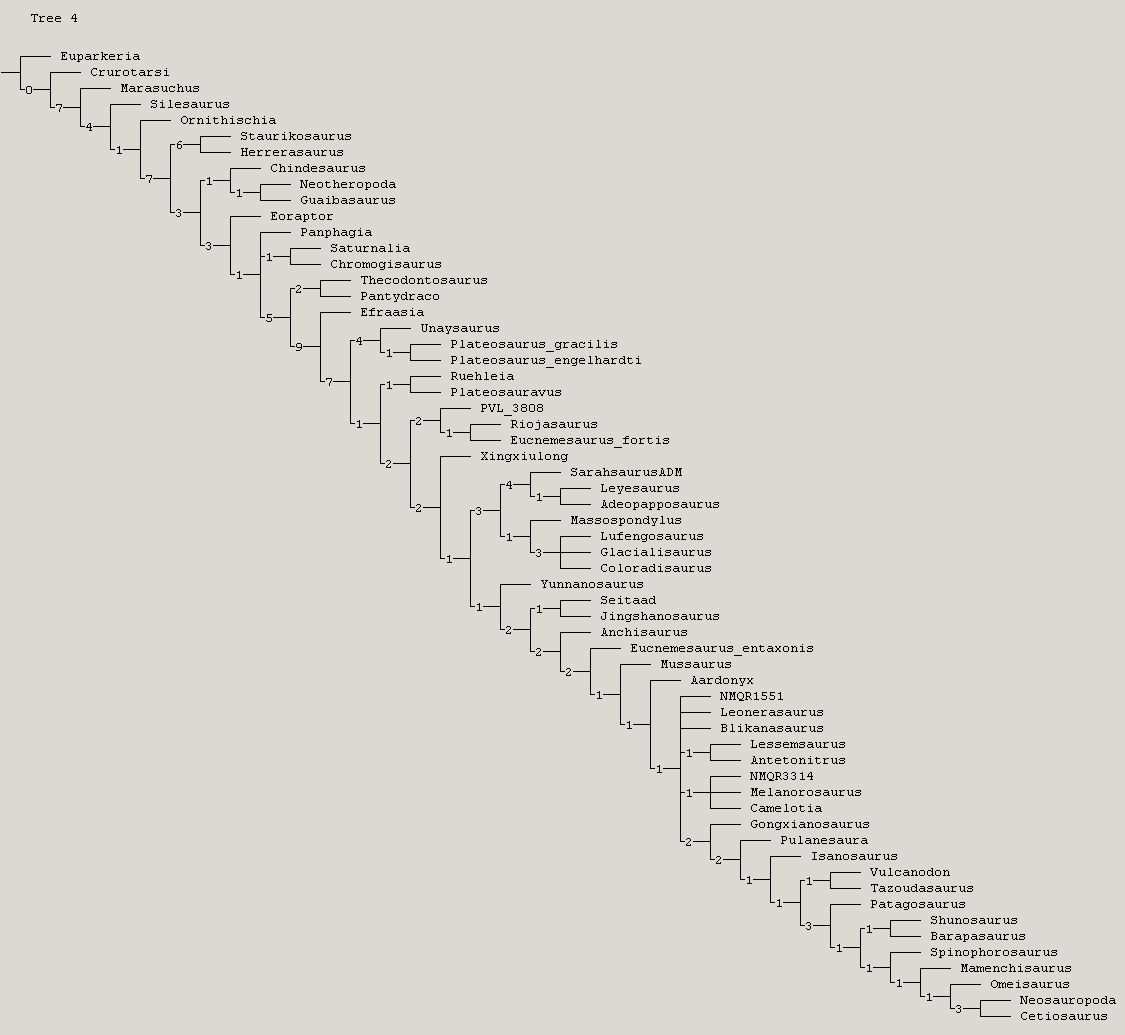

Supplement: S1 Figures — Bremer support and GC bootstrap scores for the phylogenetic analyses in this paper. (ZIP) [file pone.0204007.s006.zip › S6_Figures/McPheeChoiniere bremer.tif]

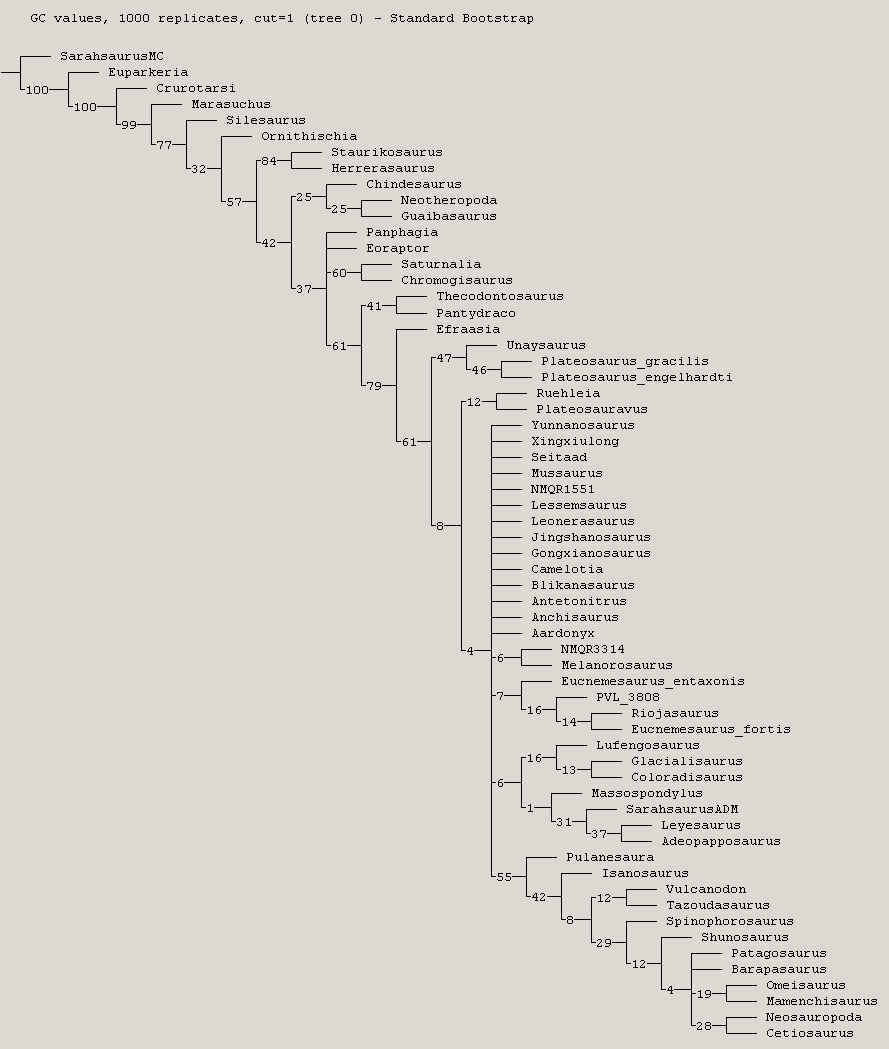

Supplement: S1 Figures — Bremer support and GC bootstrap scores for the phylogenetic analyses in this paper. (ZIP) [file pone.0204007.s006.zip › S6_Figures/McPheeChoiniere GC bootstrap.tif]

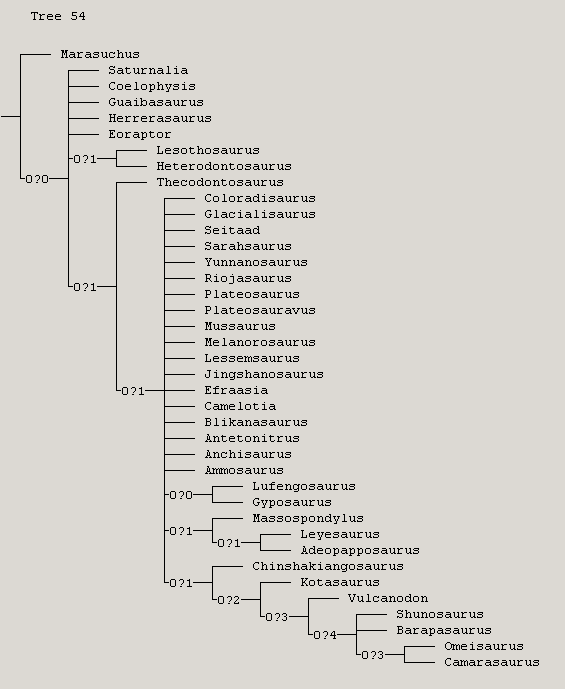

Supplement: S1 Figures — Bremer support and GC bootstrap scores for the phylogenetic analyses in this paper. (ZIP) [file pone.0204007.s006.zip › S6_Figures/Upchurch bremer.png]

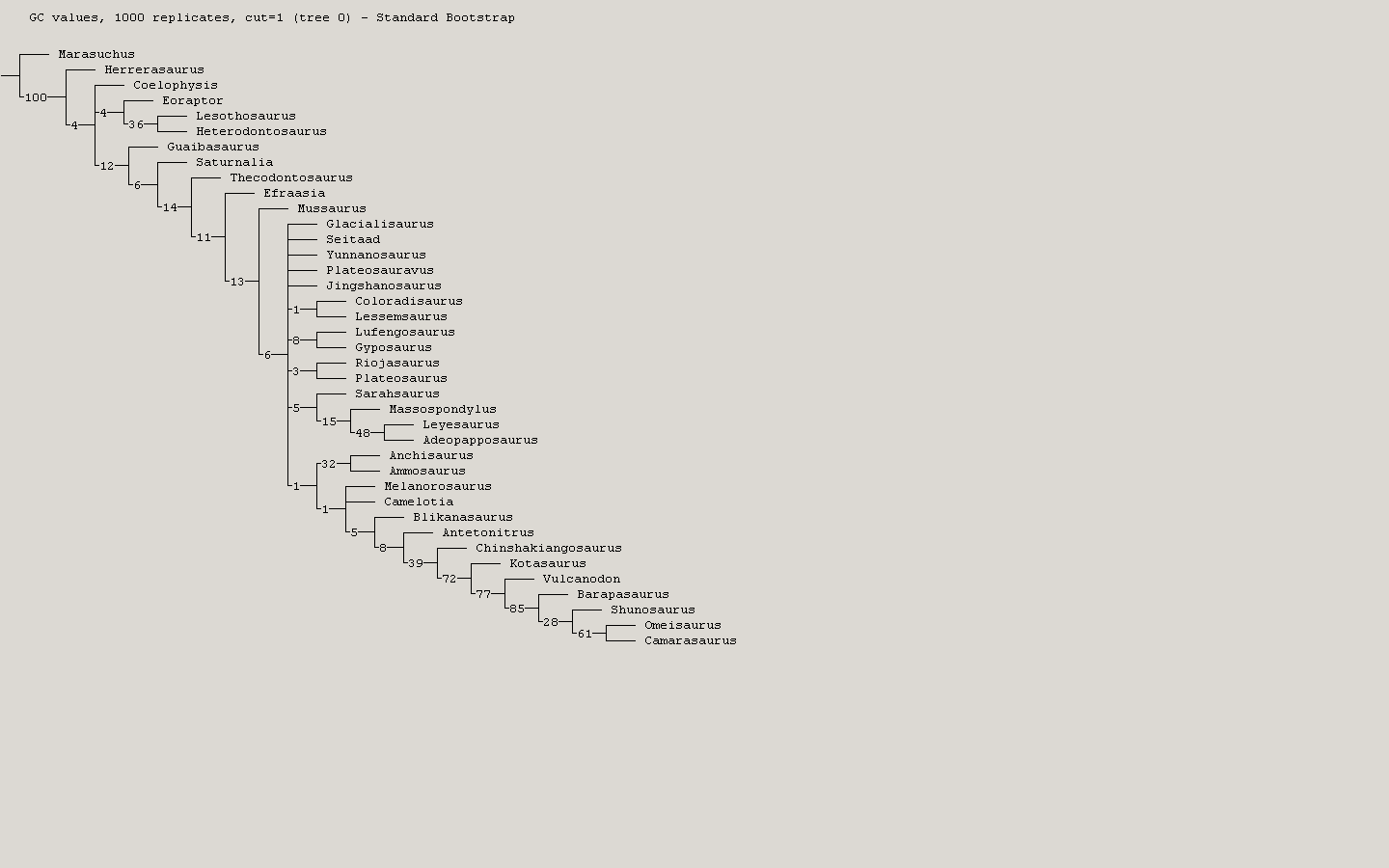

Supplement: S1 Figures — Bremer support and GC bootstrap scores for the phylogenetic analyses in this paper. (ZIP) [file pone.0204007.s006.zip › S6_Figures/Upchurch GC bootstrap.png]

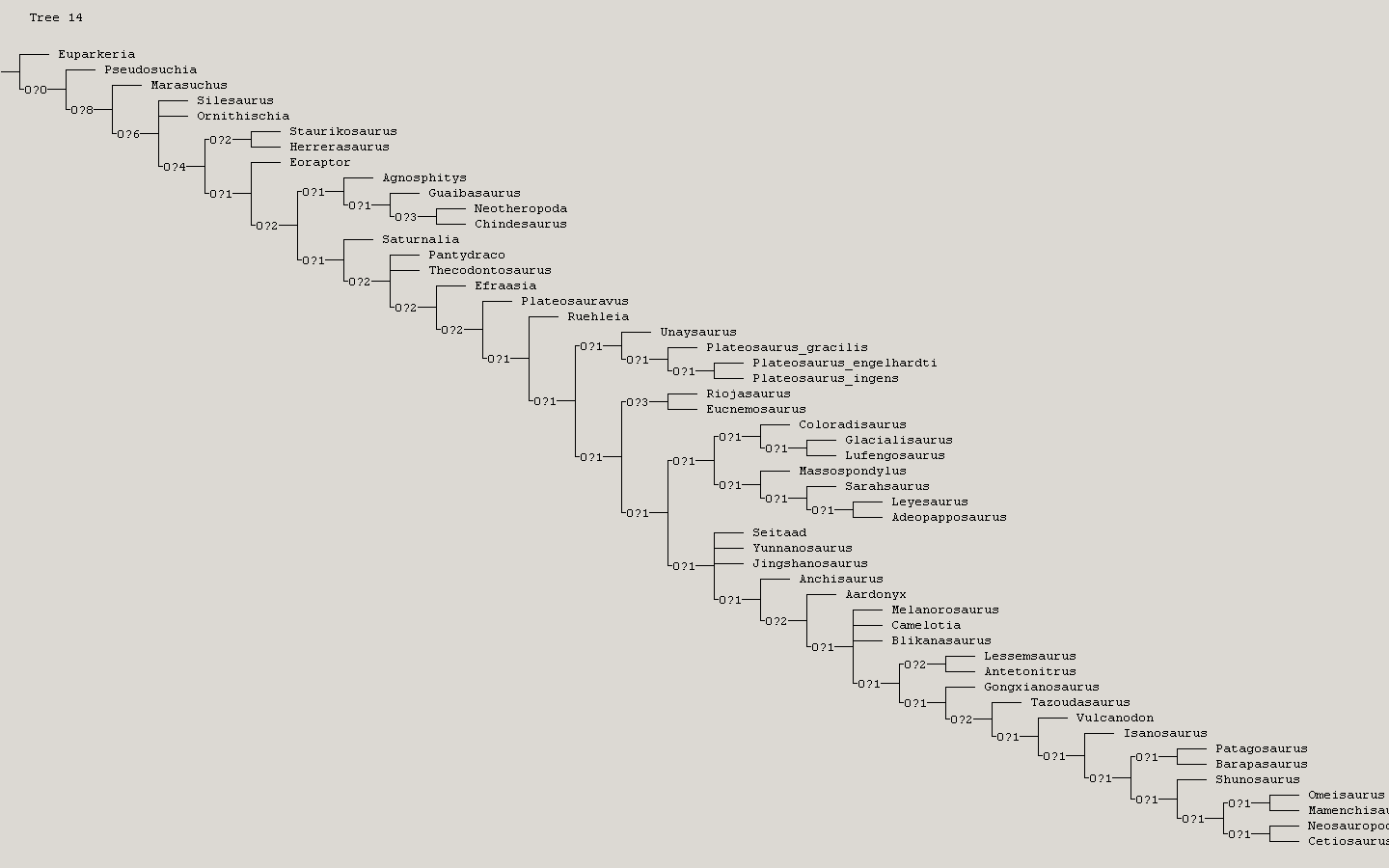

Supplement: S1 Figures — Bremer support and GC bootstrap scores for the phylogenetic analyses in this paper. (ZIP) [file pone.0204007.s006.zip › S6_Figures/Yates Bremer.png]

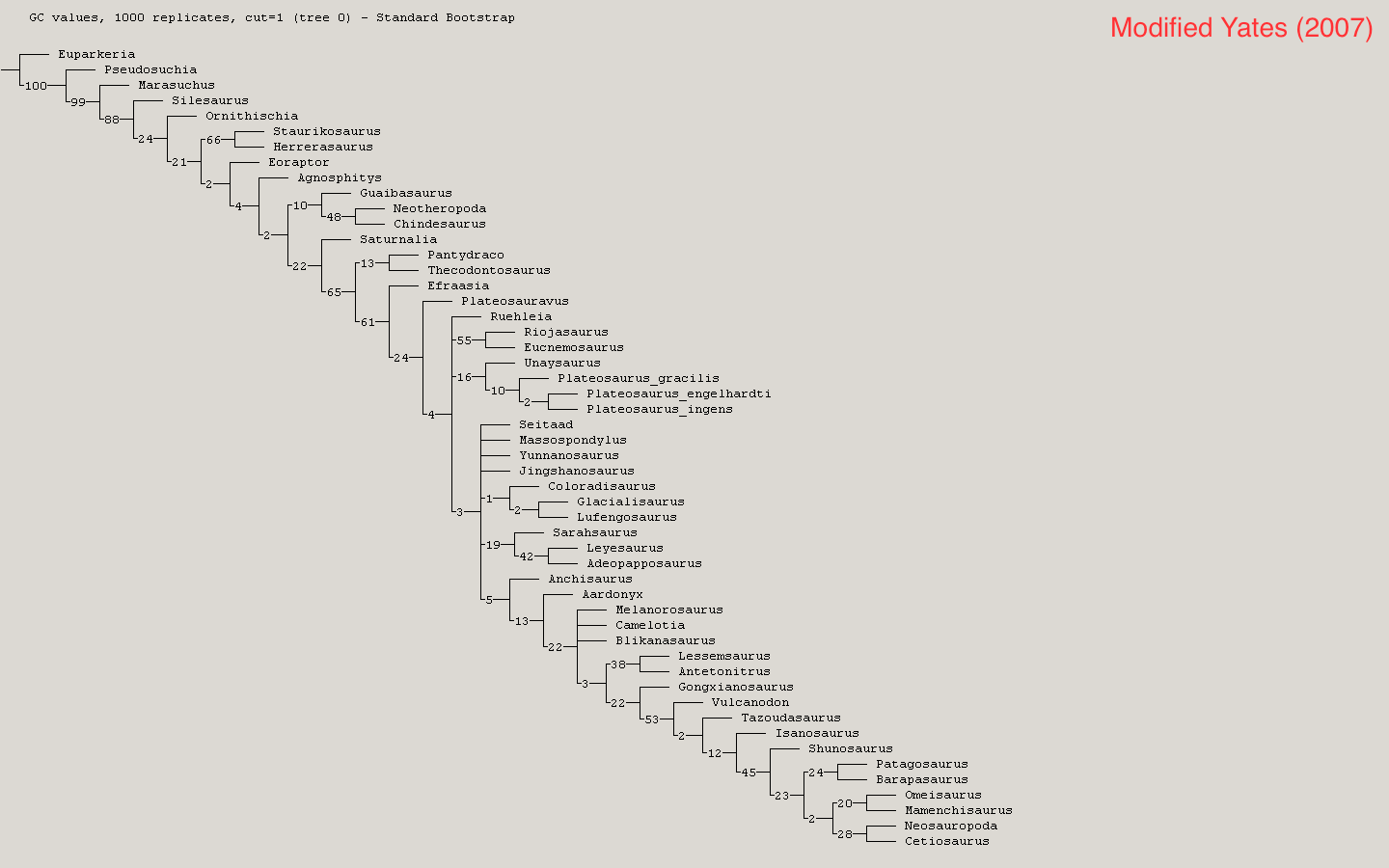

Supplement: S1 Figures — Bremer support and GC bootstrap scores for the phylogenetic analyses in this paper. (ZIP) [file pone.0204007.s006.zip › S6_Figures/Yates GC bootstrap.png]
